# Functionalized cationic [4]helicenes with unique tuning of absorption, fluorescence and chiroptical properties up to the far-red range†

**DOI:** 10.1039/c6sc00614k

**Published:** 2016-04-08

**Authors:** I. Hernández Delgado, S. Pascal, A. Wallabregue, R. Duwald, C. Besnard, L. Guénée, C. Nançoz, E. Vauthey, R. C. Tovar, J. L. Lunkley, G. Muller, J. Lacour

**Affiliations:** a Department of Organic Chemistry, University of Geneva Quai Ernest Ansermet 30 CH-1211 Geneva 4 Switzerland; b Laboratory of Crystallography, University of Geneva Quai Ernest Ansermet 24 CH-1211 Geneva 4 Switzerland; c Department of Physical Chemistry, University of Geneva Quai Ernest Ansermet 24 CH-1211 Geneva 4 Switzerland; d Department of Chemistry, San José State University 1 Washington Square San José CA 95192-0101 USA

## Abstract

Unprecedented regioselective post-functionalization of racemic and enantiopure cationic diaza [4]helicenes is afforded. The peripheral auxochrome substituents allow a general tuning of the electrochemical, photophysical and chiroptical properties of the helical dyes (26 examples). For instance, electronic absorption and circular dichroism are modulated from the orange to near-infrared spectral range (575–750 nm), fluorescence quantum efficiency is enhanced up to 0.55 (631 nm) and circularly polarized luminescence is recorded in the red (|*g*_lum_| ∼ 10^−3^).

## Abstract 2

Unprecedented regioselective post-functionalization of racemic and enantiopure cationic diaza [4]helicenes is afforded.

## Graphical abstract

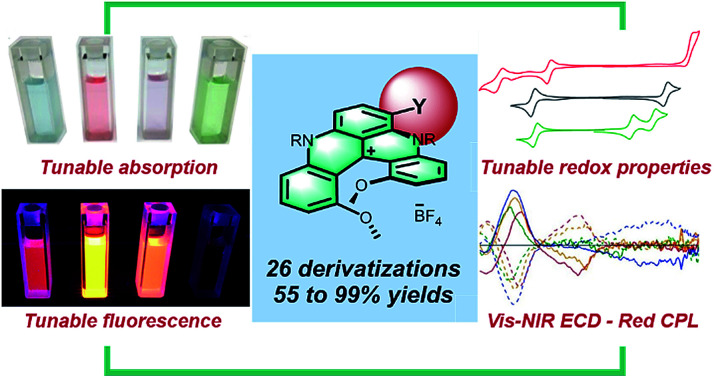

## Introduction

**Fig. 1:**
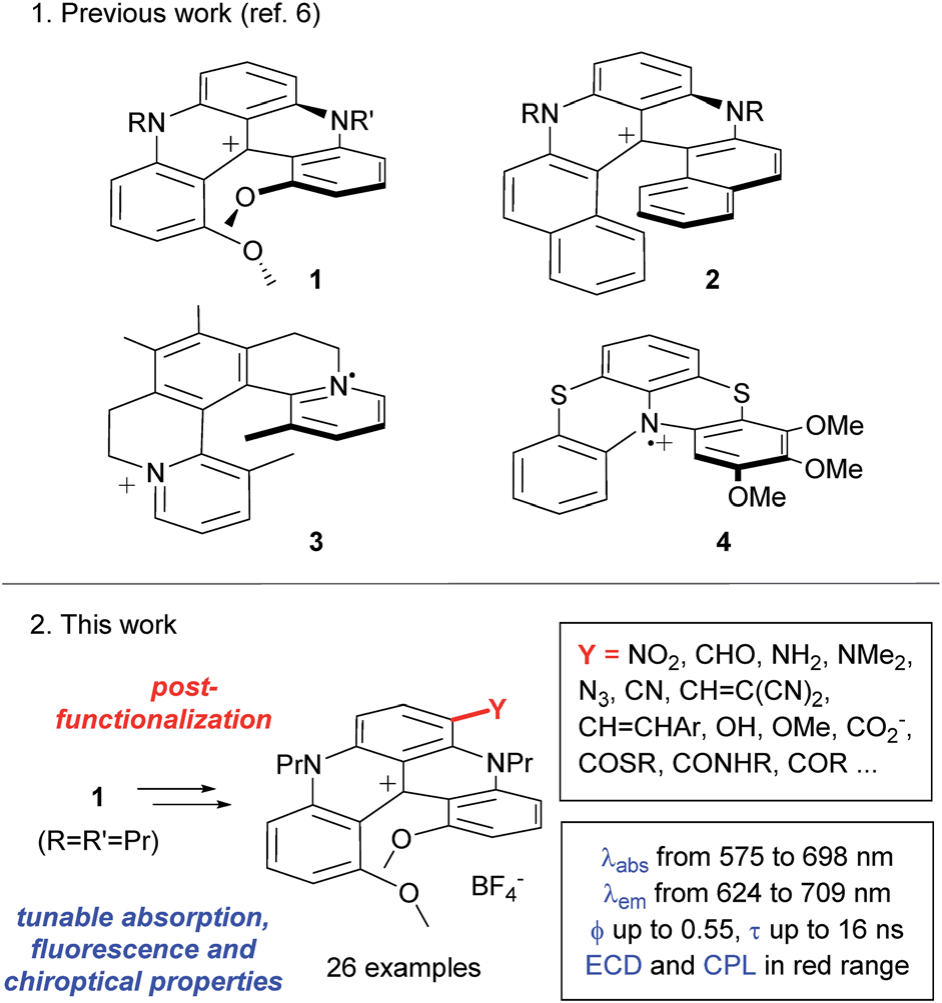
Cationic aza helical chromophores and the scope of this work.

Organic helicenes, which are defined as helical derivatives made of *ortho*-fused aromatic rings,^1^ commonly feature (chir)optical properties, *i.e.* absorption, fluorescence, electronic circular dichroism (ECD) and circularly polarized luminescence (CPL) in the blue range of the visible spectrum.^2^ Such photophysical characteristics have triggered applications in (polarized) blue light emitting diodes for instance^3^ and to a lesser extent in the field of bio-imaging.^4^ Accessing the red spectral region (620–750 nm) is essential for applications in microscopy and chemical biology in particular, and this remains a challenge for organic helicenes.^5^ In fact, only a few cationic aza derivatives, *e.g.*1–4, exhibit optical properties in this range; these moieties therefore offer limited access to specific low energy absorptions and emissions (Fig. 1, top).^6,7^ Among these species, cationic helicenes of type 1, sometimes named DMQA (DiMethoxyQuinAcridinium), are of particular interest due to their remarkable chemical (p*K*_R+_ ≈ 19)^8^ and configurational (Δ*G*^‡^ of racemization ∼ 42 kcal mol^−1^) stabilities.^9^ These derivatives are prepared on the gram-scale in no more than two steps. Starting from a tris(2,6-dimethoxyphenyl)methylium cation and using primary amines as nucleophiles, the protocol involves two consecutive aza ring closures using nucleophilic substitutions of four *ortho*-OMe groups.^9*b*,*d*,10^ Unsymmetrical derivatives with two different nitrogen substituents can also be afforded using a stepwise process.^11^ Furthermore a highly reliable resolution procedure is available using (i) addition of (+)-(*R*)-methyl-*p*-tolylsulfoxide to the central carbon, (ii) a facile chromatographic separation of the diastereomeric adducts (Δ*R*_f_ ∼ 0.3 on TLC (SiO_2_)) and (iii) a final Pummerer fragmentation.^12^ Applications in the fields of supramolecular chemistry, selective DNA binding or material science have been developed.^13^ Of importance for the current study, nitrogen substituents (H, alkyl, aryl) have only a negligible impact on the optical properties of cation 1,^14^ which are remarkably conserved with absorption and emission maxima centered at 616 and 667 nm, respectively. To modulate the photophysics, it was deemed necessary to introduce substituents at positions other than the nitrogen atoms. In fact, in the related triangulenium series, oxa or aza functional groups positioned *para* to the central cationic charge provoke efficient optical shifts (Δ*λ*_abs_ up to 50 nm).^9*c*,15^ Still, the introduction of such groups requires the preparation of specific cationic triarylmethylium precursors for each substitution pattern. Herein, the capacity to broadly tune the electrochemical, absorption, fluorescence and chiroptical properties of purely organic cationic helicenes is reported for the first time. Thanks to an unprecedented post-functionalization strategy, a large variety of substituents were introduced regioselectively at the periphery of racemic and enantiopure diaza [4]helicenes 1, in no more than four synthetic steps (Fig. 1, bottom). The functional groups induce major changes in the (photo)physical properties of the chromophores as demonstrated using electrochemical, absorption, and fluorescence studies. This allows a large tuning of the absorption and circular dichroism properties from the orange to near-infrared spectral range (575–750 nm) and an enhancement of fluorescence can be achieved with quantum yields up to 0.55 and cut-off emissions up to the far-red range. CPL activities (|*g*_lum_| ∼ 10^−3^) in the red domain were also monitored for some of these dyes.

## Results and discussion

As already mentioned, species of types 1–4 offer only limited access to specific low energy absorptions and emissions. Care was thus taken to study the viability of a post-functionalization strategy which would give access to a large variety of substituted helicenes using a single (racemic or enantiopure) [4]helicene precursor of type 1.

### Synthesis

**Scheme 1:**
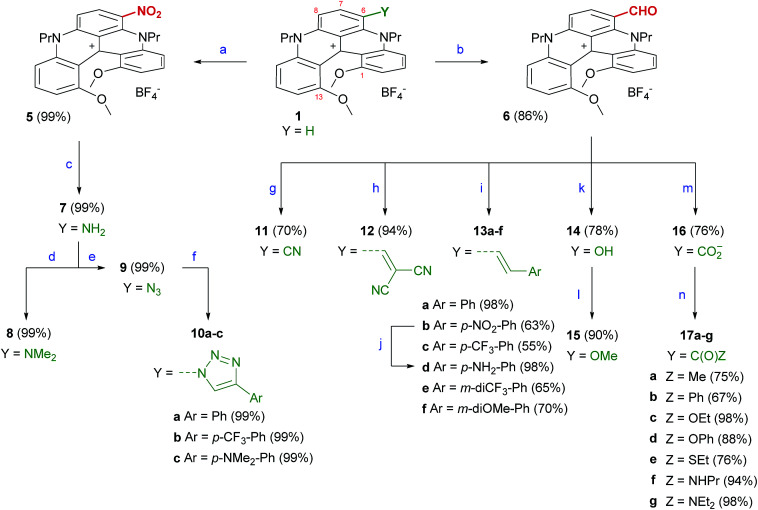
Synthesis of functionalized [4]helicenes. Reagents and conditions: (a) HNO_3_ (60% aq.), CH_2_Cl_2_, 25 °C, 15 min. (b) POCl_3_, DMF, 90 °C, 8 h. (c) H_2_, Pd/C (0.2 equiv.), CH_2_Cl_2_/MeOH, 25 °C, 1 h. (d) (i) HCHO (12 equiv.), NaBH_3_CN (7 equiv.), THF, 25 °C, 30 min; (ii) AcOH, 3 h; (iii) NaOH; (iv) 1 M aq. HBF_4_. (e) *t*BuONO (1.5 equiv.), TMSN_3_ (1.2 equiv.), MeCN, 0 to 25 °C, 3 h. (f) ArC≡CH, CuSO_4_·5H_2_O (0.2 equiv.), ascorbic acid (0.3 equiv.), NaHCO_3_ (0.3 equiv.), MeCN/H_2_O, 25 °C, 12 h. (g) NaN_3_ (1.5 equiv.), TfOH (3 equiv.), MeCN, 25 °C, 10 min. (h) NCCH_2_CN (3 equiv.), Ph_3_P (20 mol%), MeCN, 130 °C (MW), 25 °C, 1 h. (i) (i) ArCH_2_X (1.2 equiv.), Ph_3_P (1.3 equiv.), CH_2_Cl_2_; (ii) NaH (2 equiv.), DCM, 25 °C, 30 min; (iii) 1 M aq. HBF_4_. (j) AcOH (15 equiv.), Zn (5 equiv.), CH_2_Cl_2_, 25 °C, 3 h. (k) *m*CPBA (5 equiv.), CH_2_Cl_2_, 25 °C, 2 h. (l) MeI (3 equiv.), K_2_CO_3_ (5 equiv.), MeCN, reflux, 1 h. (m) NaH_2_PO_4_ (1 equiv.), NaClO_2_ (2 equiv.), H_2_O_2_, MeCN, 60 °C, 1 h. (n) (i) SOCl_2_ (6 equiv.), CH_2_Cl_2_, 25 °C, 10 min; (ii) for 17a,b: MeMgI or PhMgI (1.5 equiv.), −5 °C, 30 min, then 1 M aq. HBF_4_; for 17c,e: EtOH, PhOH or EtSH (5 or 15 equiv.), 25 °C, 10 min or 2 h; for 17f,g: PrNH_2_ or Et_2_NH (15 equiv.), 0 °C then 25 °C, 15 min.

Interestingly and somewhat surprisingly, initial halogenation attempts using classical *N*-chlorosuccinimide or *N*-bromosuccinimide reagents (1 equiv.)^6*b*^ demonstrated a strong nucleophilic reactivity for compound 1 (Fig. 1, R = R′ = ^*n*^Pr). In fact, reactions with the electrophilic agents yielded not only monohalogenated derivatives but also several polyhalogenated adducts.^16^ Complex mixtures were obtained which could not be purified. Attempts to tame the reactivity after the first halogenation were unsuccessful. To prevent polysubstitution, it became clear that the first introduced substituent should deactivate the nucleophilic character of the central aromatic core. The introduction of strong electron-withdrawing groups such NO_2_ or CHO was thus envisaged. Accordingly, conditions were found for the selective mononitration of 1 using mild biphasic conditions (HNO_3_/CH_2_Cl_2_, 25 °C, 15 min). Compound 5 was isolated in an excellent yield after a simple filtration (99%, Scheme 1). Satisfactorily, an exclusive regioselectivity was observed with only one of three formally activated positions being nitrated (atom C6 or C8, *vide infra*). For the formylation, 1 was engaged in a Vilsmeier–Haack reaction using a large excess of phosphorus oxychloride in DMF at 90 °C. Compound 6 was isolated in a good yield (86%), again as a single regioisomer. With 5 and 6 in hand, it was then possible to generate the other functionalized derivatives. Hydrogenation of 5 under heterogeneous catalysis (Pd/C, H_2_ 1 atm) led to the formation of amino derivative 7 (1 h, 99%); the formation was characterized by a visible color change from red to light green. Using modified Eschweiler–Clarke conditions (HCHO, NaBH_3_CN, acetic acid), 7 was efficiently converted into tertiary bismethylated amine 8 (99%). Primary amino 7 was transformed into azido 9 using the reaction with *tert*-butyl nitrite and azidotrimethylsilane (99%).^17^ With this compound in hand, Cu(i)-catalyzed azide–alkyne cycloadditions were performed to yield triazolo derivatives 10a, 10b and 10c (Ar = Ph, *p*-CF_3_Ph, *p*-NMe_2_Ph, 87–99%).

Aldehyde 6 was also easily derivatized. Cyano 11 was obtained through a Schmidt reaction in 70% yield.^18,19^ Olefination reactions were achieved either *via* a Knoevenagel condensation with malononitrile (12, 94%) or by Wittig reactions. For these latter transformations, a series of phosphonium salts was prepared with benzylic halides carrying electron-withdrawing and donating substituents. After formation of the ylides and condensation with 6, the resulting alkenes 13a, 13b, 13c, 13e and 13f were isolated with a perfect *E* selectivity in moderate to excellent yields (55–98%). Reduction of 13b was achieved with Zn in the presence of AcOH to form 13d (98%).^20^ Furthermore, it was possible to convert the formyl group of 6 into an hydroxyl OH group *via* a modified Dakin protocol using 3-chloroperbenzoic acid (*m*CPBA) as the oxidant (14, 78%).^21^ Compound 14 was alkylated (MeI, K_2_CO_3_) to afford 15 in 95% yield.

Finally, a series of carboxyl and carbonyl adducts was generated. Zwitterionic carboxylate 16 was obtained under mild Pinnick–Kraus conditions (76%).^22,23^ The transformation of green 16 into a corresponding deep purple acyl chloride intermediate (with SOCl_2_) led to ketones (17a and 17b, 67–75%), esters (17c and 17d, 88–98%), thioester 17e (76%) and amides (17f and 17g, 94–98%) by reactions with Grignard reagents, alcohols, a thiol and amines, respectively. These newly-substituted [4]helicenes, with the exception of 16, were prepared as tetrafluoroborate salts in racemic form and characterized using ^1^H, ^13^C, ^19^F NMR, IR and high-resolution mass spectrometry (see ESI†). Specific compounds were prepared as single enantiomers and details will be given in the following paragraphs. When possible, further structural characterization was achieved using X-ray diffraction analysis.

### Solid state structural analysis

**Fig. 2:**
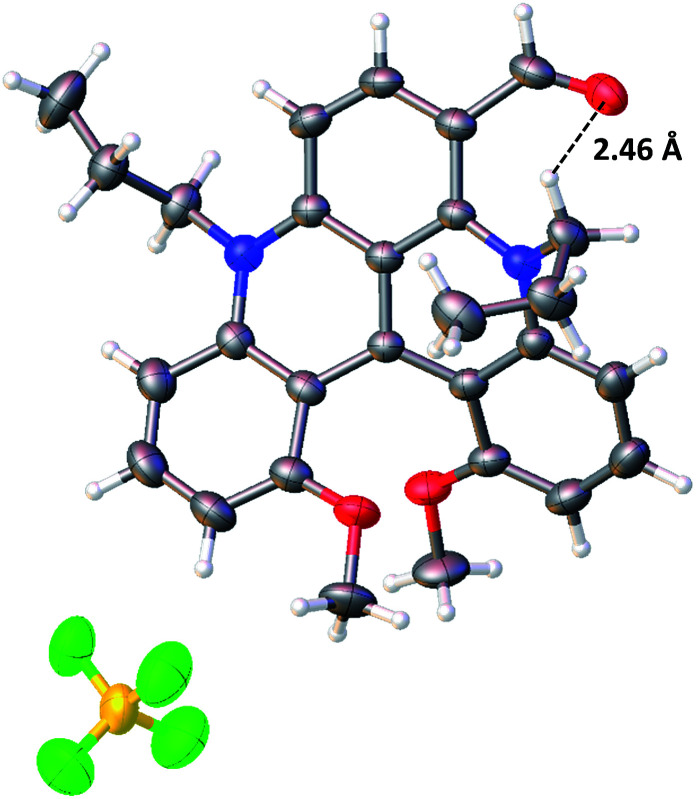
Anisotropic displacement ellipsoids plot at 50 percent probability level of the crystal structure of 6 (only the *P* enantiomer is shown).

Single crystals suitable for diffraction analysis were obtained for racemic 1, 5, 6, 7, 14, 15, 17c, 17e, 17f and 17g by the careful addition of a layer of toluene above solutions of the dyes in dichloromethane (methanol for 14) and a slow mixing of the two phases over one or two weeks (open vial usually). To crystallize 5 and 14, anion exchange metatheses of the BF_4_^−^ anion to NO_3_^−^ and Cl^−^ were necessary, respectively. The X-ray structure of 6 is presented in Fig. 2 and the relevant deduced values are reported in Table S1.† As expected for these racemates, all the space groups contain symmetry elements of the second kind. Molecules form stacks along the small unit-cell axes with typical distances between adjacent molecules of the order of 3.8–4.1 Å. Aromatic parts of the adjacent molecules in the stack are most of the time shifted to avoid strong π–π interactions.

For precursor 1, a helical angle of 41.1° formed by the planes of the terminal methoxyphenyl rings and a helical pitch of 3.19 Å between C(1) and C(13) were measured and confirmed from the previous observations.^9*b*^ The helicenes with introduced nitro (5) or carbonyl (6, 17c, 17e, 17f and 17g) functional groups possess higher helical angles and pitch (up to 45.9° and 3.26 Å for 6). In the latter case, a C–H⋯O bond between a hydrogen atom from the propyl side chain (NC*H*_2_ fragment) and the oxygen of the carbonyl function can be noticed (Fig. 2). This interaction, induced by a probably strong *δ*^+^ character of the hydrogen atoms adjacent to the nitrogen, leads to an *inwards* (*syn*) conformation of the aldehyde moiety that points towards the exocyclic methylene group.^24^ This strained geometry might be the reason for the distortion of the helical skeleton. Interestingly, this conformation was evidenced in solution using a ^1^H NOESY NMR experiment (CD_2_Cl_2_, 298 K), which shows a through-space correlation between the H of the formyl group and H(7) (see the ESI, Fig. S3†).

The bond lengths of the carbocations were also extracted from the X-ray data and analyzed (Fig. S1†). An alternation of single and double bonds within the three internal heterocycles could not be evidenced; only a slight single/double bond character of the terminal methoxybenzene rings was noticed. This suggests a strong resonance within the benzonaphthyridinylium scaffold in which the cationic charge is presumably fully delocalized.^25^ From this section, one can conclude that the substituents introduced at the C(6) position may interact with the pendant alkyl chains and influence the helical core.

### Electronic properties

**Fig. 3:**
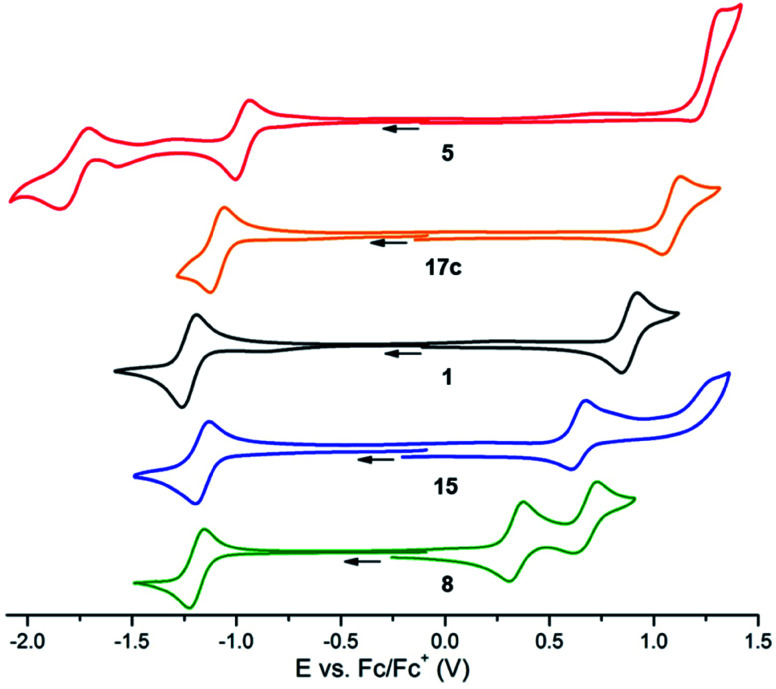
Voltammetric curves of acetonitrile ([TBA][PF_6_] 10^−1^ M) solutions of 5 (red), 17c (orange), 1 (black), 15 (blue) and 8 (green) (10^−3^ M) recorded at a Pt working electrode (*ν* = 0.1 V s^−1^).

Cyclic voltammetry (CV) experiments were performed using anhydrous acetonitrile solutions with tetrabutylammonium hexafluorophosphate as the supporting electrolyte. The voltammograms of representative dyes 1, 5, 8, 15 and 17c are presented in Fig. 3. Unsubstituted precursor 1 was used as a reference. As previously reported,^9*d*^ this compound displays a reversible reduction at −1.23 V *versus* Fc/Fc^+^ and a pseudo-reversible oxidation at 0.88 V. In comparison, the CV of nitro-substituted dye 5 reveals a reversible reduction at −0.97 V and a second reduction corresponding to the formation of the carbanion at ∼−1.78 V.^26^ Only an irreversible oxidation is recorded at high potential (1.30 V) and the increase of scan rate to 2 V s^−1^ did not allow a reversibility, suggesting a faster chemical step occurring after the oxidation. Ester 17c also displayed a reversible reduction and a pseudo-reversible oxidation, which is very similar to that of 1 except with both reduction and oxidation occurring at higher potentials (−1.09 V and 1.08 V respectively). Such an influence from electron-withdrawing NO_2_ and CO_2_Et groups was expected. Electron-rich 15 and 8 were reduced at comparable potentials to 1 but their first oxidation was, on the contrary, considerably facilitated (peaks at 0.64 V and 0.34 V, respectively). Moreover, 8 presented a reversible second oxidation at 0.67 V, marking the strong electronic donating effect of the dimethylamino group. An extension of the analysis to other derivatives was performed. Details can be found in Table 1 and in the ESI (Table S2, Fig. S3†).^27^ In other instances, complex voltammograms were obtained due to the inherent electroactivity of the substituents and these data are not presented. In a general manner, the introduced moieties have a stronger influence on the first oxidation potentials (Table 1, from 5 to 7: ΔΔ*E*(Ox_1_) = 0.96 V) than on the first reduction potentials (ΔΔ*E*(Red_1_) = 0.32 V); this modulation of the electrochemical properties bodes well for tunable optical properties.

**Table 1:** Anodic and cathodic half-wave potentials values (mV) measured using CV for selected [4]helicenes (10^−3^ M) in acetonitrile ([TBA][PF_6_] 10^−1^ M) at a Pt electrode (*Ø* = 3 mm, *ν* = 0.1 V s^−1^), *E vs.* Fc/Fc^+^. Compounds are ranked by increasing first oxidation potentials. Red_*n*_ and Ox_*n*_ represent the *n* successive reduction and oxidation processes, respectively

Molecule	Functional group (Y)	Reduction^a^			Oxidation^a^		
		Red_1_	Δ*E*(Red_1_)^b^	Red_2_	Ox_1_	Δ*E*(Ox_1_)^b^	Ox_2_
5	NO_2_	−970	−256	−1778	1304^c^	420	—
6	CHO	−1059	−167	−1269	1131	247	—
17c	CO_2_Et	−1091	−135	—	1084	200	—
17f	CONHPr	−1120	−106	−1877	1000	116	—
1	H	−1226	0	—	884	0	—
9	N_3_	−1094	−132	−1834^c^	769	−115	1275^c^
16	CO_2_^−^	−1285	59	—	711^c^	−173	1006
15	OMe	−1164	−62	—	640	−244	1258^c^
7	NH_2_	−1201	−25	—	354	−530	781^c^
8	NMe_2_	−1191	−35	—	342	−542	673

a Half-wave potentials values, otherwise noted.

b Potential difference compared to the first reduction or oxidation waves of compound 1.

c Irreversible process.

### Electronic absorption

**Fig. 4:**
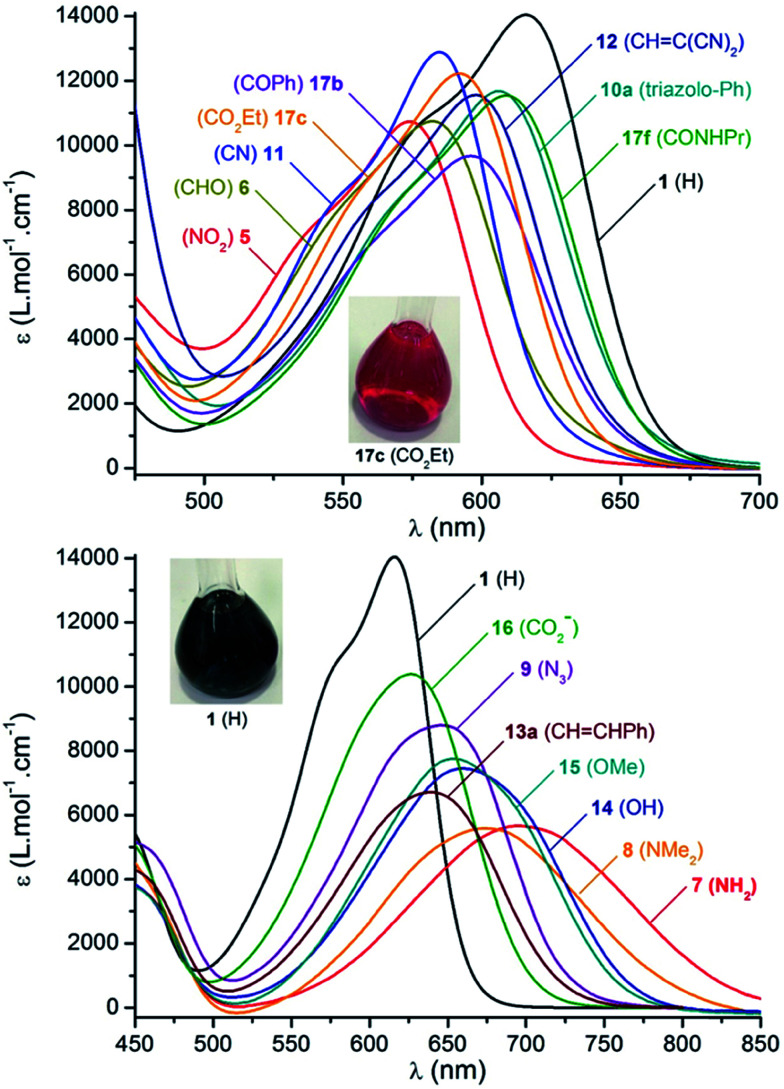
Selected absorption spectra in acetonitrile (10^−5^ M) at 293 K as a function of substituent Y. Top and bottom diagrams depict spectra presenting hypsochromic and bathochromic shifts compared to ref. 1 respectively. Insets: typical coloration of acetonitrile solutions (10^−4^ M).

Absorption spectra were recorded in acetonitrile and the results are presented in Fig. 4, data are compiled in Table 2. Unsubstituted precursor 1 was again used as a reference to evaluate the influence of the different functional groups. Dye 1 absorption is characterized using a relatively sharp and moderately intense maximum at 616 nm (full width at half maximum, fwhm = 2460 cm^−1^, *ε* = 14 000 M^−1^ cm^−1^), with a shoulder at higher energy. Upon introduction of electron-withdrawing functions, the lower energy transition undergoes a moderate hypsochromic shift. For instance the strong electron-withdrawing nitro function in 5 leads to a 40 nm blue-shift of the absorption maximum and a slight weakening of the molar extinction coefficient. Weaker aldehyde (6), cyano (11), ketone (17a,b) and ester (17c,e) substituents provoke a less pronounced hypsochromic shift of the absorption with maxima located in the yellow-orange window (∼580–600 nm). Compounds 10a–c and 17f,g display negligible shifts of absorption due to the poor electronic effect brought about by the triazolo and amido functions.

**Table 2:** Photophysical properties of [4]helicenes in acetonitrile

Molecule	Functional group (Y)	*λ*_max_/nm (*ε*_max_/L mol^−1^ cm^−1^)	fwhm/cm^−1^	*λ*_em_/nm	*Φ* a	*τ* b/ns	*k*_R_ c/10^6^ s^−1^	*k*_NR_ c/10^6^ s^−1^
1	H	616 (14 000)	2460	667	0.13	5.5	23.6	158.2
5	NO_2_	575 (12 000)	2440	624	0.35	14.5	24.1	44.8
6	CHO	582 (10 750)	2500	640	0.37	16.2	22.8	38.9
7	NH_2_	698 (6560)	3280	—	—	—	—	—
8	NMe_2_	675 (5580)	3280	—	—	—	—	—
9	N_3_	644 (8790)	2890	—	—	—	—	—
10a	Triazolo-Ph	606 (11 670)	2430	670	0.19	8.5	22.4	95.3
10b	Triazolo-*p*-CF_3_-Ph	605 (12 820)	2370	665	0.24	10.4	23.1	73.1
10c	Triazolo-*p*-NMe_2_-Ph	607 (11 520)	2420	—	—	—	—	—
11	CN	585 (12 880)	2290	631	0.55	19.1	28.8	23.6
12	CHC(CN)_2_	598 (11 550)	2540	658	0.28	15.1	18.5	47.7
13a	CH=CH-Ph	638 (6710)	2910	—	—	—	—	—
13b	CH=CH-*p*-NO_2_-Ph	638 (8090)	2810	—	—	—	—	—
13c	CH=CH-*p*-CF_3_-Ph	640 (8810)	2840	—	—	—	—	—
13d	CH=CH-*p*-NH_2_-Ph	660 (5600)	3170	—	—	—	—	—
13e	CH=CH-*m*-diCF_3_-Ph	636 (8750)	2770	—	—	—	—	—
13f	CH=CH-*m*-diOMe-Ph	644 (7960)	2860	—	—	—	—	—
14	OH	659 (7450)	3040	—	—	—	—	—
15	OMe	653 (7750)	3030	—	—	—	—	—
16	CO_2_^−^	626 (10 400)	2840	709	0.01	—	—	—
17a	COMe	593 (9080)	2430	657	0.26	12.0	21.7	61.7
17b	COPh	598 (9130)	2450	659	0.27	12.1	22.3	60.3
17c	CO_2_Et	592 (12 200)	2370	653	0.33	14.0	23.6	47.9
17d	CO_2_Ph	586 (11 700)	2380	641	0.39	16.3	23.9	37.4
17e	COSEt	596 (11 100)	2370	655	0.34	14.3	23.8	46.2
17f	CONHPr	609 (11 500)	2420	667	0.19	8.3	22.9	97.6
17g	CONEt_2_	615 (10 300)	2450	678	0.13	6.2	21.0	140.3

a Reference: cresyl violet (*Φ* = 0.54 in methanol), estimated error ± 10%.

b Excitation at 470 nm.

c With *k*_R_ = *Φ*/*τ* and *k*_NR_ = (1 − *Φ*)/*τ*.

In the series of helicenes carrying electron-withdrawing groups (Fig. 4, top), the shape of the lower energy transition remains remarkably comparable to 1, with fwhm between 2370 and 2540 cm^−1^. Furthermore, the absorption spectra of chromophores 1 and 5, were recorded in a wide range of solvents (Fig. S4†). 1 displays similar absorption bands in solvents ranging from THF to DMSO (Δ*ν̄* = 230 cm^−1^), indicating a small electronic rearrangement between the ground and excited states.^28,29^ This absence of solvatochromism was also observed for the lower energy transition of 5 (Δ*ν̄* = 120 cm^−1^). Electron-withdrawing substituents have therefore only a minor influence and the electronic delocalization is poorly perturbed, leading to limited spectroscopic changes.

In contrast and not surprisingly, absorption maxima are red-shifted with electron-donating groups (Fig. 4, bottom). The smallest effects are observed for the carboxylato 16, azido 9 and olefinic 13a derivatives that exhibit maxima centered at 626, 638 and 644 nm, respectively. Within the series of compounds 13a to 13f, it was noticed that extra substituents on the styrene fragments have little influence. Only in the case of *p*-aminophenyl 13d, is a broader transition observed (with a lower intensity and a shift of +20 nm compared to 13a). More noticeable effects are displayed with 14 and 15 that present both similar absorption properties (*λ*_abs_ = 659 and 653 nm respectively). The most dramatically red-shifted absorptions are recorded for dyes 7 and 8, with the absorption red edge extending to the near-infrared (NIR) range, around 800–850 nm. Interestingly, the primary amine function in 7 induces a stronger shift of the absorption towards lower energies compared to its tertiary amine functionalized analogue 8. This bathochromic effect brought on by electron-donating groups is further characterized by a progressive decrease of the molar extinction coefficient from *ε* = 10 400 to 5580 M^−1^ cm^−1^ in compounds 16 to 8. A marked broadening of the transition towards quasi-gaussian profiles also results from the increase in electron-donating strength of the substituents (fwhm = 2840 to 3280 cm^−1^ from 16 to 8). Such a trend is characteristic of the establishment of an internal charge transfer. As previously, the solvatochromism was studied and compounds 7, 8, 14 and 15 were selected (Fig. S4†). Products 8 and 15 show no variation of absorption maxima and fwhm (Δ*ν̄* = 196 and 209 cm^−1^, respectively), and hence a lack of solvatochromism. In contrast, non-methylated analogues 7 and 14 display an inverse solvatochromism characterized by bathochromic and hypochromic shifts upon lowering the polarity of the medium (Δ*ν̄* = 744 and 944 cm^−1^, respectively). The appearance of solvatochromism with the non-methylated 7 and 14 suggests the occurrence of H-bonding interactions.

### Fluorescence

**Fig. 5:**
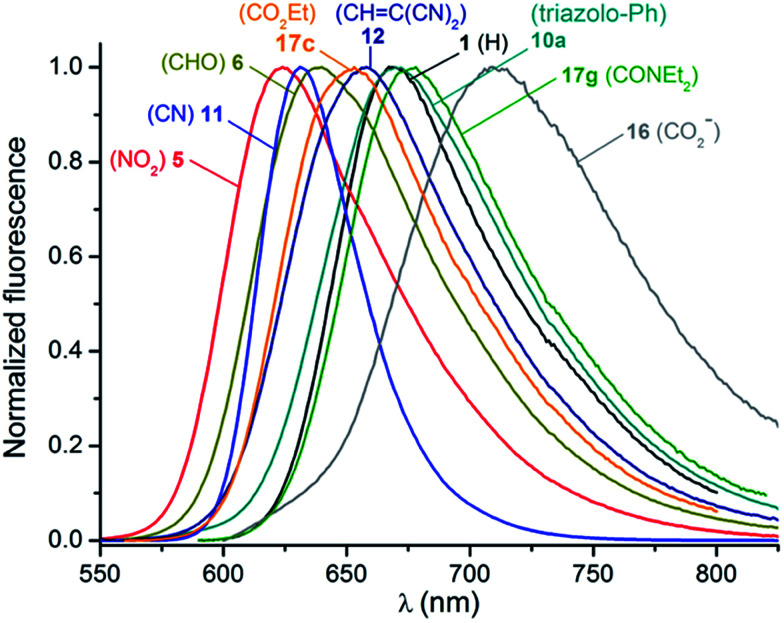
Normalized fluorescence spectra of selected [4]helicene dyes in acetonitrile.

The emission properties of the [4]helicenes were also recorded in acetonitrile and selected fluorescence spectra are displayed in Fig. 5. 1 shows an emission maximum at 667 nm characterized by a modest fluorescence quantum yield of 0.13 and a lifetime of 5.5 ns, as previously reported.^14*a*^ The majority of the chromophores functionalized with an electron-withdrawing function exhibit moderate to strong emission in the red optical region with quantum yields ranging from 0.13 (17g) to 0.55 (11). The fluorescence lifetimes are relatively high for organic chromophores in this spectral range, with values between 6.2 ns (17g) and 19.1 ns (11), as presented in Fig. S6.† In this series, the emission spectra are the mirror image of the lower energy absorption bands and show Stokes shifts values of ∼1300–1600 cm^−1^. In contrast and interestingly, fluorescence of the electron-enriched chromophores was not observed under the current experimental conditions. As displayed in Fig. S7,† the quantum efficiency declines progressively with the concomitant increase in emission wavelength, illustrating the effect of the energy gap law.^30^ Moreover, the radiative and non-radiative kinetic constants compared in Table 2 highlight the strong increase of de-excitation *via* non-radiative pathways as the fluorescence maxima are red-shifted while the radiative constants remain in the same range of values (20–30 × 10^−6^ s^−1^), assuming a negligibly small triplet yield.

### Electronic circular dichroism and circularly polarized luminescence

**Fig. 6:**
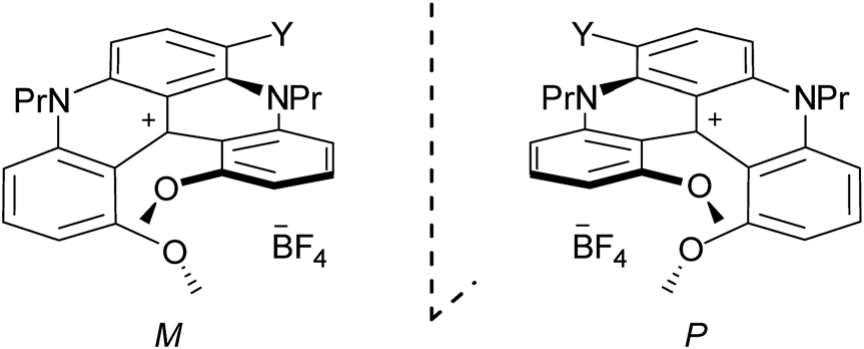
*M* and *P* enantiomers of 1, 5, 6 and 7 (Y=H, NO_2_, CHO, NH_2_).

**Fig. 7:**
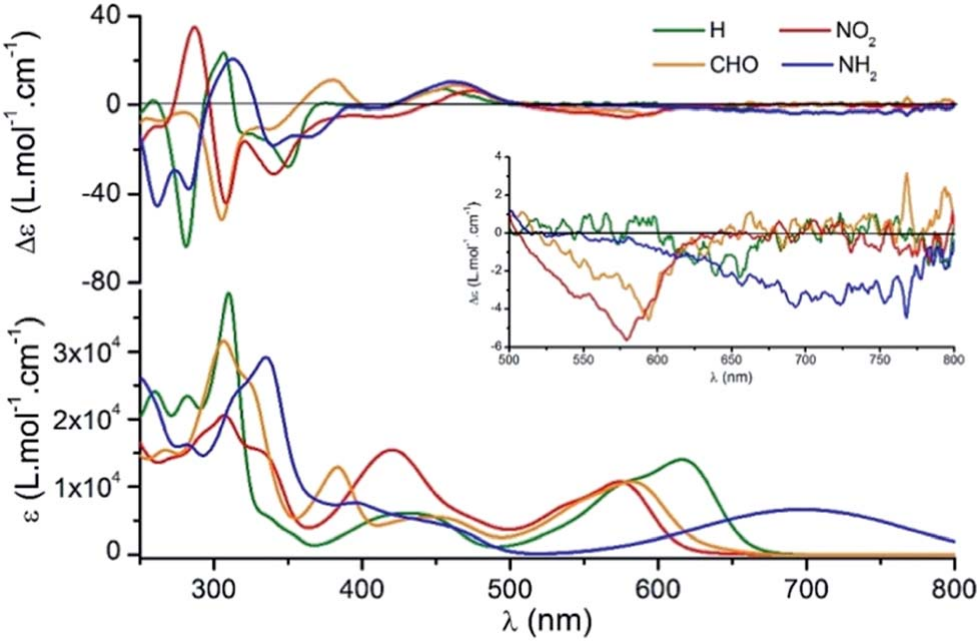
(Top) UV-Vis-NIR ECD spectra of the *M*-helices for compounds 1 (green), 5 (red), 6 (orange) and 7 (blue) in acetonitrile (10^−5^ M) at 293 K. (Bottom) UV-Vis-NIR electronic absorption spectra in acetonitrile (10^−5^ M). Inset: ECD in the vis-NIR range.

Using *M*-1 and *P*-1 as precursors instead of the racemate,^12*b*^ compounds 5, 6 and 7 were prepared as single enantiomers (Fig. 6). In Fig. 7 the UV-Vis-NIR electronic circular dichroism (ECD) spectra of the *M* enantiomers are displayed. The spectra of the *P* antipodes are reported in the ESI (Fig. S8†). The four dyes present pronounced ECD bands in the UV range with a Cotton effect near their most intense higher energy transitions. As predicted by Elm and co-workers,^31^ the functionalized helicenes feature stronger circular dichroism in the visible region than the parent 1. Chromophores *M*-5 and *M*-6 unambiguously display absorption of circularly polarized light between 400 and 600 nm, corresponding to the first and second low energy absorption transitions. Compound *M*-7 constitutes a rare example of a purely organic helicene exhibiting ECD in the far-red and NIR region of the electromagnetic spectrum.^6*c*,*d*,32^

**Fig. 8:**
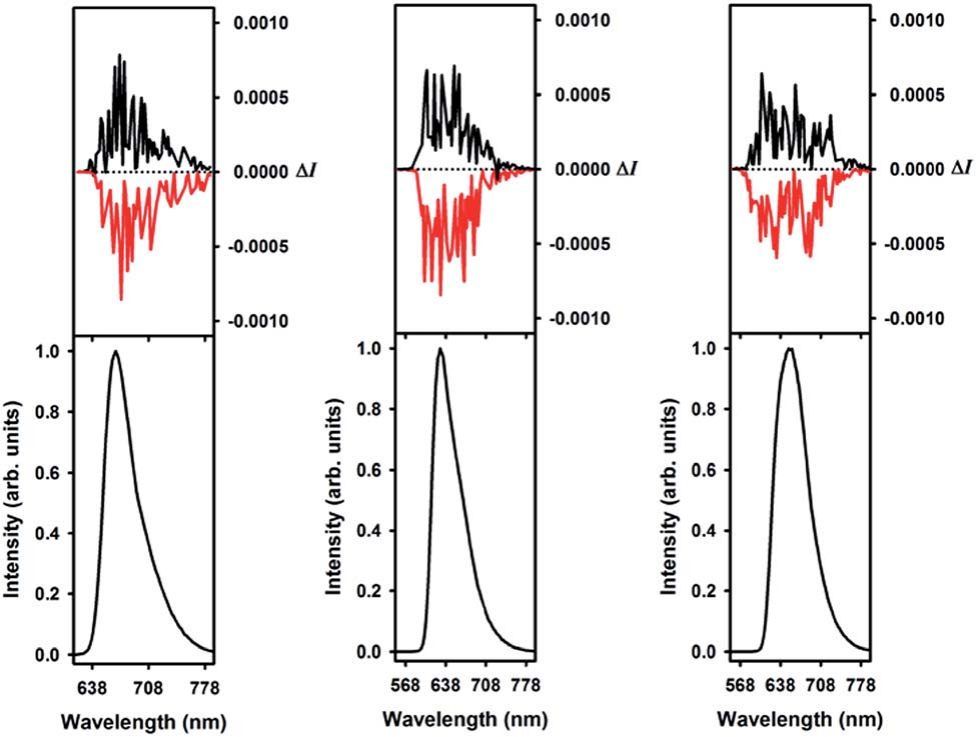
Circularly polarized luminescence (upper curves) and total luminescence (lower curves) spectra of *P*-(+) and *M*-(−)-1 (left), 5 (middle), and 6 (right) in 2 mM degassed dichloromethane solutions at 295 K, upon excitation at 497/473, 495/473, and 455/472 nm, respectively (black for *P*-(+) and red for *M*-(−)).

The circularly polarized luminescence (CPL) spectra were recorded for both enantiomers of 1, 5 and 6 and are presented in Fig. 8. Although a very weak CPL was measured, opposite trend signals were observed for the *P*-(+) and *M*-(−) enantiomers in the same wavelength as the corresponding unpolarized fluorescence. The *g*_lum_ values are +0.0013/−0.0010, +0.0016/−0.0017, and +0.0009/−0.0008 at the vicinity of the maximum emission wavelength for the *P*-(+)/*M*-(−) helices of 1, 5, and 6, respectively. These CPL intensities are in the same order as previously reported chiral organic dyes and bodipys^33^ but present the originality to be located in the red range, an unusual spectral region for helicene-like chromophores.^2^ To our knowledge, only helicene derivatives benefiting from the presence of transition metal fragments have achieved similar outputs.^34^

## Conclusions

In summary, direct post-functionalization routes to a large variety of substituted cationic diaza [4]helicenes have been achieved. More than twenty new chromophores were prepared in moderate to excellent yields (55–99%). Structural solid-state analyses have revealed higher helical angles and pitches upon introduction of the substituents (up to 45.9° and 3.26 Å). Moreover, a strongly resonant π-system was evidenced by the non-alternant bond lengths of the conjugated core. Furthermore a broad tuning of the electrochemical and optical properties can be achieved by the selection of the peripheral substituent. While electron-withdrawing functional groups led to highly fluorescent derivatives emitting in the red domain, electron-donating residues allowed an extension of the low-energy absorption band towards the far-red and NIR regions. Thanks to these properties, ECD and CPL spectra were recorded at unusual wavelengths for purely organic helicenes, in the far-red spectral range. Some of these compounds should be well-suited for high-contrast imaging applications using confocal microscopy in the transparency window of biological media.^13*b*,35^

## Supplementary Material

SC-007-C6SC00614K-s001

SC-007-C6SC00614K-s002
