# Supplementary material for: Functionalized cationic [4]helicenes with unique tuning of absorption, fluorescence and chiroptical properties up to the far-red range
Source: Chem Sci. 2016 Apr 8;7(7):4685–93. doi: 10.1039/c6sc00614k (PMC5772034; doi:10.1039/c6sc00614k)
Supplement: SC-007-C6SC00614K-s001 [file SC-007-C6SC00614K-s001.pdf]

Electronic Supplementary Material (ESI) for Chemical Science.
This journal is © The Royal Society of Chemistry 2016

Supporting Information

Functionalized Cationic [4]Helicenes with Unique Tuning of Absorption, Fluorescence and Chiroptical Properties up to the Far-Red Range

Irene Hernández Delgado, Simon Pascal, Antoine Wallabregue, Romain Duwald, Céline Besnard, Laure Guénée, Christoph Nançoz, Eric Vauthey, Roberto C. Tovar, Jamie L. Lunkley, Gilles Muller, and Jérôme Lacour

1. GENERAL REMARKS AND ANALYSIS CONDITIONS 2

2. SYNTHETIC PROTOCOLS AND CHARACTERIZATIONS 4

3. ^{1}H, ^{13}C, ^{19}F NMR AND HRMS ANALYSIS 21

4. X-RAY DIFFRACTION 73

5. ^{1}H-NOESY EXPERIMENT 85

6. ADDITIONAL CYCLIC VOLTAMMETRY DATA 86

7. ADDITIONAL OPTICAL DATA 88

1. SOLVATOCHROMISM 88
2. FLUORESCENCE 90
3. ECD AND OR DATA 91

8. REFERENCES 93

1. General remarks and analysis conditions

Reagents. Compound **1** was prepared according to reported procedures from tris(2,6-dimethoxyphenyl)methylium tetrafluoroborate.[1,2] Column chromatography were performed using Siliaflash P60 silicagel (40-63 µm, 60 Å) and Acros Brockmann I basic alumina (40-200 µm, 60 Å). Optical properties were recorded in analytical grade solvents.

Analytical methods and apparatus. NMR spectra were recorded on Brucker Advance II+ AMX-500 and AMX-400 spectrometers at room temperature (otherwise noted). NMR chemical shifts are given in ppm (δ) relative to Me_4Si with solvent resonances used as internal standards (CD_2Cl_2: 5.32 ppm for ^1H and 53.8 for ^{13}C; CD_3OD: 3.31 ppm for ^1H and 49.0 for ^{13}C; DMSO-d_6: 2.50 ppm for ^1H and 39.5 for ^{13}C). IR spectra were recorded on a Perkin-Elmer 1650 FT-IR spectrometer using a diamond ATR Golden Gate sampling. Melting points (M.P.) were measured in open capillary tubes with a Buchi B-550 melting points apparatus and are uncorrected. UV-Vis-NIR absorption spectra were recorded on a JASCO V-650 spectrophotometer at 20°C. Electrospray mass spectra were obtained on a Finnigan SSQ 7000 spectrometer QSTAR pulsar *i* (AB / MDS Sciex), ESI (TIS)/nanoESI/APCI-QqTof by the Department of Mass Spectroscopy of the University of Geneva. Optical rotation were measured on a Perkin Elmer 241 polarimeter at 20°C using a Hg lamp (365 nm). Electronic circular dichroism spectra were recorded on a JASCO J-815 spectrophotometer at 20 °C.

Crystallography. All data were collected on an Agilent supernova dual source diffractometer equipped with an Atlas detector, using Cu Kα radiation. Data reduction was carried out in the crysalis Pro Software.[3] Structure solution was made using direct methods (Shelxs[4] or sir2004[5]) or charge-flipping (Superflip[6]). Refinements were carried out in ShexlL[4] within the Olex2[7] software.

Electrochemistry. Cyclic voltammetry (CV) data were recorded using a CH instruments potentiostat. All the experiments were conducted under an argon atmosphere in a standard one-compartment, three-electrode electrochemical cell placed in a faraday cage. Tetra-n-butylammonium hexafluorophosphate ([TBA][PF_6]) in anhydrous acetonitrile was used as supporting electrolytes (10^{-1} M). All the electrodes were purchased from BAS Inc. Platinum (Ø = 3 mm) working electrodes was polished with 0.05 µm alumina paste before each recording. An Ag|$AgNO_3$ (10^{-2} M + TBA•PF_6 10^{-1} M) electrode was used as a pseudo-reference. Before experiment, the solution was degassed using argon for 5 minutes. Ferrocene was used as redox potential of reference.

Fluorescence. Steady-state fluorescence spectra were measured using a Varian Cary 50 Eclipse spectrofluorimeter. All fluorescence spectra were corrected for the wavelength-dependent sensitivity of the detection. Fluorescence quantum yields Φ were measured in diluted solutions (at least 5 different concentrations for each sample) with an optical density lower than 0.1 using the following equation:

$$\frac{\Phi_x}{\Phi_r} = \left(\frac{\mathrm{A}_r(\lambda)}{\mathrm{A}_x(\lambda)}\right)\left(\frac{n_x^2}{n_r^2}\right)\left(\frac{\mathrm{D}_x}{D_r}\right)$$

where A is the absorbance at the excitation wavelength (λ), n the refractive index and D the integrated intensity. “r” and “x” stand for reference and sample. The fluorescence quantum yields were measured relative to cresyl violet in methanol (Φ = 0.54). Excitation of reference and sample compounds was performed at the same wavelength.

Fluorescence lifetime. Fluorescence dynamics on the nanosecond timescale were measured by a time-correlated single photon counting (TCSPC) setup described in detail previously.[8,9] Excitation was performed at 470 nm using ~60 ps pulse at 10 MHz produced by a laser diode (Picoquant, LDH-P-C-470). The full width at half-maximum (fwhm) of the instrument response function (IRF) was around 200 ps. The fluorescence time profiles were analysed with the convolution of the experimental IRF and an exponential function.

Circularly polarized luminescence. The circularly polarized luminescence (CPL) and total luminescence spectra were recorded on an instrument described previously,[10] operating in a differential photon-counting mode. The light source for excitation was a continuous wave 1000 W xenon arc lamp from a Spex Fluorolog-2 spectrofluorimeter, equipped with excitation and emission monochromators with dispersion of 4 nm/mm (SPEX, 1681B). To prevent artifacts associated with the presence of linear polarization in the emission,[11] a high quality linear polarizer was placed in the sample compartment, and aligned so that the excitation beam was linearly polarized in the direction of emission detection (z-axis). The key feature of this geometry is that it ensures that the molecules that have been excited and that are subsequently emitting are isotropically distributed in the plane (x,y) perpendicular to the direction of emission detection. The optical system detection consisted of a focusing lens, long pass filter, and 0.22 m monochromator. The emitted light was detected by a cooled EMI-9558B photomultiplier tube operating in photo-counting mode. All measurements were performed with quartz cuvettes with a path length of 1.0 cm.

2. Synthetic protocols and characterizations

1,13-Dimethoxy-6-nitro-5,9-dipropyl-5,9-dihydro-13b*H*-quinolino[2,3,4-*kl*]acridin-13b-ylium tetrafluoroborate (5)

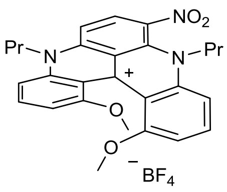

To a solution of **1** (1 g, 2 mmol) in CH_2Cl_2 (40 mL, 0.05 M) were added 40 mL of aqueous HNO_3 (60%, 0.05 M). After 15 min of stirring at 25 °C (monitored by TLC), the reaction mixture was quenched by addition of aqueous NaOH (1 M) and then, it was extracted with CH_2Cl_2 (3 x 20 mL) and washed with HBF_4 1 M in H_2O. The organic layer was dried over Na_2SO_4, filtered and evaporated under *vacuum*. The desired product was obtained as a red solid (1.08 g, 99%) after filtration.

R_f = 0.6 (SiO_2, CH_2Cl_2/MeOH, 98:2). **M. P.:** 273-275 °C (decomposition). **^{1}H NMR (400 MHz, CD_2Cl_2):** δ = 8.81 (d, *J* = 9.6 Hz, 1H, CH), 8.13 (t, *J* = 8.4 Hz, 1H, CH), 7.99 (t, *J* = 8.4 Hz, 1H, CH), 7.65 – 7.57 (m, 2H, CH), 7.49 (d, *J* = 8.6 Hz, 1H, CH), 7.10 (d, *J* = 8.1 Hz, 1H, CH), 7.01 (d, *J* = 8.2 Hz, 1H, CH), 4.97 – 4.75 (m, 2H, CH), 4.71 – 4.56 (m, 1H, CH), 3.84 (s, 3H, OCH_3), 3.79 (s, 3H, OCH_3), 3.59 – 3.44 (m, 1H, CH), 2.31 – 2.14 (m, 2H, CH_2), 1.84 – 1.69 (m, 2H, CH_2), 1.29 (t, *J* = 7.4 Hz, 3H, CH_3), 0.45 (t, *J* = 7.3 Hz, 3H, CH_3). **^{13}C{^{1}H} NMR (101 MHz, CD_2Cl_2):** δ 160.2 (C), 159.6 (C), 141.8 (C), 141.7 (C), 141.4 (C), 141.3 (C), 139.6 (CH), 138.01 (CH), 136.5 (C), 134.7 (CH), 132.1 (C), 120.5 (C), 116.9 (C), 114.5 (C), 110.4 (CH), 108.3 (CH), 106.7 (CH), 105.8 (CH), 105.4 (CH), 58.8 (CH_2), 56.7 (OCH_3), 56.5 (OCH_3), 22.6 (CH_2), 21.3 (CH_2), 11.4 (CH_3), 10.8 (CH_3). **^{19}F NMR (282 MHz, CD_2Cl_2):** δ = -152.5. **UV-Vis:** λ_{max} (CH_3CN) = 575 nm (ε = 12000 $L.mol^{-1}.cm^{-1}$). **IR (CH_2Cl_2, cm^{-1}):** ν = 3107, 2969, 2880, 1585, 1500, 1468, 1389, 1285, 1168, 1040, 817, 731, 699, 632. **HRMS (ESI+)** calculated for [M+]: 458.2074 ($C_{27}H_{28}N_3O_4^+$), Found 458.2097.

6-Formyl-1,13-dimethoxy-5,9-dipropyl-5,9-dihydro-13b*H*-quinolino[2,3,4-*kl*]acridin-13b-ylium tetrafluoroborate (6)

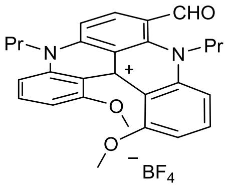

To a solution of **1** (1 g, 2.00 mmol) in DMF (1.85 mL, 24.00 mmol) at 90 °C was added $POCl_3$ (4.5 mL, 45.00 mmol). After being stirred for 2 h at this temperature (monitored by ESI-MS), H_2O (10 mL) was added to the reaction mixture at 25 °C and stirred for 30 min. Then, the mixture was washed with aqueous 5% LiCl solution and extracted with CH_2Cl_2 (3 x 20 mL). The organic layer was washed with HBF_4 1 M in H_2O, dried over Na_2SO_4, filtered and evaporated under *vacuum*. The crude material was purified by flash chromatography on silica gel (CH_2Cl_2/MeOH, 98:2). The desired product is obtained as a purple solid (908 mg, 86%).

R_f = 0.5 (SiO_2, CH_2Cl_2/MeOH, 98:2). **M. P.:** 264-266 °C (decomposition). **^{1}H NMR (400 MHz, CD_2Cl_2):** δ = 10.17 (s, 1H, CHO), 8.60 (d, *J* = 9.0 Hz, 1H, CH), 8.07 (t, *J* = 8.2 Hz, 1H, CH), 7.96 (t, *J* = 8.4 Hz, 1H, CH), 7.68 (d, *J* = 9.0 Hz, 1H, CH), 7.56 (d, *J* = 8.8 Hz, 2H, CH), 7.05 (d, *J* = 8.1 Hz, 1H, CH), 6.97 (d, *J* = 8.0 Hz, 1H, CH), 5.20 – 5.13 (m, 1H, CH), 4.82 – 4.74 (m, 1H, CH), 4.64 – 4.55 (m, 1H, CH), 4.09 – 4.01 (m, 1H, CH), 3.82 (s, 3H, OCH_3), 3.78 (s, 3H, OCH_3), 2.30 – 2.17 (m, 2H, CH_2), 1.81 – 1.67 (m, 2H, CH_2), 1.30 (t, *J* = 7.4 Hz, 3H, CH_3), 0.41 (t, *J* = 7.3 Hz, 3H, CH_3). **^{13}C{^{1}H} NMR (101 MHz, CD_2Cl_2):** δ = 186.9 (C), 160.2

(C), 159.6 (C), 142.4 (CH), 142.1 (C), 141.9 (C), 141.7 (C), 141.5 (C), 141.0 (C), 138.9 (CH), 137.4 (CH), 120.2 (C), 117.7 (C), 116.8 (C), 114.2 (C), 110.7 (CH), 108.1 (CH), 106.9 (CH), 105.2 (CH), 104.8 (CH), 60.8 (CH_2), 56.6 (OCH_3), 56.4 (OCH_3), 23.0 (CH_2), 21.1 (CH_2), 11.4 (CH_3), 10.9 (CH_3). **^{19}F NMR** (**282 MHz, CD_2Cl_2):** δ = -152.5. **UV-Vis:** λ_{max} (CH_3CN) = 582 nm (ε = 10750 L.mol^{-1}.cm^{-1}). **IR (CH_2Cl_2, cm^{-1}):** ν = 2961, 2961, 2752, 1686, 1583, 1506, 1470, 1343, 1266, 1171, 1050, 818, 784, 732, 653. **HRMS (ESI+)** calculated for [M+]: 441.2173 ($C_{28}H_{29}N_2O_3^+$), Found 441.2192.

6-amino-1,13-dimethoxy-5,9-dipropyl-5,9-dihydro-13b*H*-quinolino[2,3,4-*kl*]acridin-13b-ylium tetrafluoroborate (7)

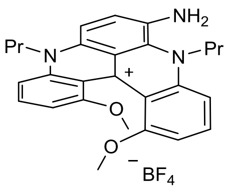

To solution of **5** (1.084 g, 2.00 mmol) in CH_2Cl_2/methanol (60:20 mL) under N_2 atmosphere was added Pd/C (108 mg, 10% wt). Then H_2 was bubbled in the reaction mixture for 5 min and stirred further for 30 min at 25 °C (monitored by ESI-MS). Then, the reaction mixture was purged with N_2 and filtered over celite®, with CH_2Cl_2. The solvent was evaporated under *vacuum*. The desired product was obtained as a light green solid (1.14 g, 99%) after evaporation of solvent.

R_f = 0.4 (SiO_2, CH_2Cl_2/MeOH, 98:2). **M. P.:** 231-233 °C (decomposition). **^{1}H NMR** (**400 MHz, CD_2Cl_2):** δ = 7.85 (t, *J* = 9.0, 7.8 Hz, 1H, CH), 7.77 (d, *J* = 9.1 Hz, 1H, CH), 7.70 (t, *J* = 8.4 Hz, 1H, CH), 7.52 (d, *J* = 9.1 Hz, 1H, CH), 7.34 (d, *J* = 9.0 Hz, 1H, CH), 7.29 (d, *J* = 8.8 Hz, 1H, CH), 6.81 (d, *J* = 8.0 Hz, 1H, CH), 6.69 (d, *J* = 8.1 Hz, 1H, CH), 4.79 – 4.71 (m, 1H, CH), 4.67 – 4.58 (m, 1H, CH), 4.49 – 4.39 (m, 2H, CH), 4.28 (s, 2H, NH_2), 3.76 (s, 3H, OCH_3), 3.69 (s, 3H, OCH_3), 2.26 – 2.05 (m, 2H, CH_2), 1.63 – 1.45 (m, 2H, CH_2), 1.24 (t, *J* = 7.4 Hz, 3H, CH_3), 0.53 (t, *J* = 7.4 Hz, 3H, CH_3). **^{13}C{^{1}H} NMR** (**101 MHz, CD_2Cl_2):** δ = 160.1 (C), 159.6 (C), 148.0 (C), 143.6 (C), 142.4 (C), 137.2 (CH), 136.4 (CH), 132.2 (C), 131.6 (C), 128.0 (C), 126.9 (CH), 123.8 (C), 117.7 (C), 114.6 (C), 110.5 (CH), 107.9 (CH), 107.8 (CH), 103.1 (CH), 103.1 (CH), 56.3 (OCH_3), 56.0 (OCH_3), 51.6 (CH_2), 22.7 (CH_2), 21.1 (CH_2), 11.5 (CH_3), 11.3 (CH_3). **^{19}F NMR** (**282 MHz, CD_2Cl_2):** δ = -152.4. **UV-Vis:** λ_{max} (CH_3CN) = 698 nm (ε = 6560 L.mol^{-1}.cm^{-1}). **IR (CH_2Cl_2, cm^{-1}):** ν = 3663, 2964, 1605, 1578, 1559, 1502, 1470, 1369, 1263, 1166, 1056, 812, 775, 730, 656. **HRMS (ESI+)** calculated for [M+]: 428.2333 ($C_{27}H_{30}N_3O_4^+$), Found 428.2325.

6-(dimethylamino)-1,13-dimethoxy-5,9-dipropyl-5,9-dihydro-13bH-quinolino[2,3,4-kl]acridin-13b-ylium tetrafluoroborate (8)

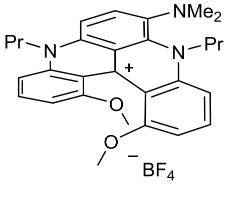

To solution of **7** (500 mg, 0.97 mmol) in THF (12.6 mL) was added aqueous formaldehyde (40%, 350 mg, 11.64 mmol) and the reaction mixture was stirred for 15 min. Then $NaBH_3CN$ (427 mg, 6.80 mmol) was added, and the reaction mixture was stirred for 15 min, followed by addition of AcOH (1.26 mL, 22.0 mmol). The resulting mixture was stirred for 1 h at 25 °C (monitored by ESI-MS). The reaction was quenched by addition of NaOH 1 N to adjust the solution to pH ≈ 7. The reaction mixture was extracted with CH_2Cl_2 (3 x 20 mL) and washed with HBF_4 1 M in H_2O. The organic layer was dried over Na_2SO_4, filtered and evaporated under *vacuum*. The crude material was dissolved in a minimum amount of CH_2Cl_2 and then

the addition of Et_2O led to the precipitation of the product, which was separated from the mother liquor by centrifugation. The desired product was further purified by flash chromatography (CH_2Cl_2/MeOH, 98:2) as a light green solid (495 mg, 94%).

R_f = 0.5 (SiO_2, CH_2Cl_2/MeOH, 98:2). **M. P.:** 122-124 °C (decomposition). **^{1}H NMR (400 MHz, CD_2Cl_2):** δ = 7.98 (d, *J* = 9.2 Hz, 1H, CH), 7.91 (t, *J* = 8.5 Hz, 1H, CH), 7.78 (t, *J* = 8.4 Hz, 1H, CH), 7.60 (d, *J* = 9.2 Hz, 1H, CH), 7.39 (d, *J* = 9.2 Hz, 2H, CH), 6.86 (d, *J* = 8.0 Hz, 1H, CH), 6.77 (d, *J* = 8.1 Hz, 1H, CH), 5.19 – 5.11 (m, 1H, CH), 4.71 – 4.62 (m, 1H, CH), 4.51 – 4.42 (m, 2H, CH), 3.78 (s, 3H, OCH_3), 3.72 (s, 3H, OCH_3), 3.2 – 2.5 (bs, 6H, NCH_3), 2.27 – 2.06 (m, 3H, CH_2), 1.48 – 1.38 (m, 2H, CH_2), 1.25 (t, *J* = 7.4 Hz, 3H, CH_3), 0.51 (t, *J* = 7.3 Hz, 3H, CH_3). **$^{13}C\{^1H\}$ NMR (101 MHz, CD_2Cl_2):** δ = 160.1 (C), 159.7 (C), 146.7 (C), 143.2 (C), 142.4 (C), 137.8 (C), 137.7 (CH), 136.8 (CH), 133.6 (C), 131.0 (C), 128.3 (CH), 123.5 (C), 116.9 (C), 114.2 (C), 109.5 (CH), 107.8 (CH), 107.2 (CH), 103.3 (CH), 103.2 (CH), 56.3 (OCH_3), 56.1 (OCH_3), 50.4 (CH_2), 42.2 (NCH_3), 22.6 (CH_2), 20.9 (CH_2), 11.4 (CH_3), 11.4 (CH_3). **^{19}F NMR (282 MHz, CD_2Cl_2):** δ = -152.8. **UV-Vis:** λ_{max} (CH_3CN) = 675 nm (ε = 5580 L.mol^{-1}.cm^{-1}). **IR (CH_2Cl_2, cm^{-1}):** ν = 2963, 2847, 1601, 1577, 1558, 1450, 1470, 1321, 1263, 1170, 1051, 921, 814, 779, 732, 620. **HRMS (ESI+)** calculated for [M+]: 456.2645 ($C_{29}H_{34}N_3O_2^+$), Found 456.2645.

6-azido-1,13-dimethoxy-5,9-dipropyl-5,9-dihydro-13bH-quinolino[2,3,4-kl]acridin-13b-ylium tetrafluoroborate (9)

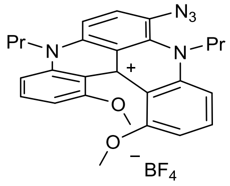

To solution of **7** (200 mg, 0.39 mmol) in MeCN (0.39 mL) at 0 °C and in the absence of light, *t*-BuONO (70 µL, 0.60 mmol) and $TMSN_3$ (102 µL, 0.78 mmol) were added dropwise. The reaction mixture was stirred for 3 h at 25 °C (monitored by ESI-MS). Then, the addition of Et_2O led to the precipitation of the product, which was separated from the mother liquor by centrifugation. The desired product was futher purified by flash chromatography (CH_2Cl_2/MeOH, 98:2) as a green solid (208 mg,99%).

R_f = 0.4 (SiO_2, CH_2Cl_2/MeOH, 98:2). **M. P.:** 190-192 °C (decomposition). **^{1}H NMR (400 MHz, CD_2Cl_2):** δ = 8.02 (d, *J* = 9.1 Hz, 1H, CH), 7.96 (t, *J* = 8.9 Hz, 1H, CH), 7.83 (t, *J* = 8.8 Hz, 1H, CH), 7.66 (d, *J* = 9.2 Hz, 1H), 7.42 (d, *J* = 9.2 Hz, 1H, CH), 7.38 (d, *J* = 9.2 Hz, 1H, CH), 6.90 (d, *J* = 8.1 Hz, 1H), 6.81 (d, *J* = 8.2 Hz, 1H), 4.95 – 4.87 (m, 1H, CH), 4.74 – 4.59 (m, 2H, CH), 4.53 – 4.44 (m, 1H, CH), 3.77 (s, 3H, OCH_3), 3.72 (s, 3H, OCH_3), 2.26 – 2.11 (m, 2H, CH_2), 1.86 – 1.76 (m, 2H, CH_2), 1.26 (t, *J* = 7.4 Hz, 4H, CH_3), 0.70 (t, *J* = 7.4 Hz, 3H, CH_3). **$^{13}C\{^1H\}$ NMR (101 MHz, CD_2Cl_2):** δ = 160.1 (C), 159.6 (C), 145.6 (C), 143.4 (C), 142.4 (C), 138.4 (CH), 137.5 (CH), 135.7 (C), 131.0 (C), 127.8 (CH), 122.3 (C), 121.1 (C), 116.4 (C), 114.4 (C), 109.6 (CH), 107.9 (CH), 107.6 (CH), 104.0 (CH), 103.8 (CH), 56.4 (OCH_3), 56.2 (OCH_3), 55.7 (CH_2), 23.2 (CH_2), 20.9 (CH_2), 11.4 (CH_3), 11.3 (CH_3). **^{19}F NMR (282 MHz, CD_2Cl_2)** δ = -152.4. **UV-Vis:** λ_{max} (CH_3CN) = 644 nm (ε = 8790 L.mol^{-1}.cm^{-1}). **IR (CH_2Cl_2, cm^{-1}):** ν = 3556, 3108, 2966, 2880, 2112, 1602, 1578, 1500, 1470, 1323, 1263, 1170, 1127, 1044, 813, 776, 732, 711, 639. **HRMS (ESI+)** calculated for [M+][-N_2]: 426.2176 ($C_{27}H_{28}N_3O_2^+$), Found 426.2188.

General procedure for the synthesis of triazolo quinacridinium tetrafluoroborate salts (10a-c).

To a solution of **9** (25 mg, 0.05 mmol) and the corresponding alkyne (0.1 – 0.25 mmol) in methanol (40 µL), was added a solution of $CuSO_4 \cdot 5H_2O$ (0.9 mg, 8 mol%) dropwise, followed by ascorbic acid (1.7 mg, 21 mol%) and $NaHCO_3$ (0.8 mg, 21 mol%) in water (40 µL). The reaction mixture was stirred for 4 - 24 h (monitored by ESI-MS). The crude product was precipitated in Et_2O. The crude precipitate was filtered and purified by dissolving it in a minimum amount of CH_2Cl_2 and then the addition of Et_2O led to the precipitation of the product, which was separated from the mother liquor by centrifugation. Desired products were obtained as light green solids.

1,13-dimethoxy-6-(4-phenyl-1H-1,2,3-triazol-1-yl)-5,9-dipropyl-5,9-dihydro-13bH-quinolino[2,3,4-kl]acridin-13b-ylium tetrafluoroborate (10a)

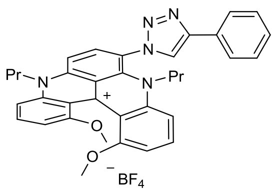

Prepared according to general procedure using ethynylbenzene (5 µL, 0.1 mmol). The reaction mixture was stirred for 4 h. Purification by selective precipitation with CH_2Cl_2 and Et_2O provided the product as a light green solid (29 mg, 99%).
R_f = 0.3 (SiO_2, CH_2Cl_2/MeOH, 98:2). **M. P.:** 267-269 °C (decomposition). **^{1}H NMR** (**400 MHz, CD_2Cl_2**)**:** δ = 8.42 (s, 1H, CH), 8.24 (d, *J* = 9.1 Hz, 1H), 8.05 (t, *J* = 8.4 Hz, 1H, CH), 8.01 (d, *J* = 8.0 Hz, 2H, CH), 7.86 (t, *J* = 8.4 Hz, 1H, CH), 7.72 (d, *J* = 9.1 Hz, 1H, CH), 7.56 – 7.47 (m, 3H, CH), 7.43 – 7.38 (m, 1H, CH), 7.29 (d, *J* = 8.6 Hz, 1H, CH), 7.00 (d, *J* = 8.1 Hz, 1H, CH), 6.90 (d, *J* = 8.1 Hz, 1H, CH), 4.82 – 4.72 (m, 1H, CH), 4.65 – 4.54 (m, 1H, CH), 4.28 – 4.19 (m, 1H, CH), 3.81 (s, 3H. OCH_3), 3.77 (s, 3H, OCH_3), 3.15 – 3.06 (m, 1H, CH), 2.34 – 2.12 (m, 2H, CH_2), 1.68 – 1.44 (m, 2H, CH_2), 1.29 (t, *J* = 7.4 Hz, 3H, CH_3), 0.38 (t, *J* = 7.3 Hz, 3H, CH_3). **^{13}C{^{1}H} NMR** (**101 MHz, CD_2Cl_2**)**:** δ = 160.2 (C), 159.6 (C), 149.4 (C), 144.0 (C), 143.2 (C), 142.4 (C), 139.0 (CH), 134.0 (C), 137.6 (CH), 135.9 (C), 135.8 (CH), 130.4 (C), 129.6 (CH), 129.3 (CH), 126.5 (CH), 122.1 (CH), 121.8 (C), 119.1 (C), 116.9 (C), 114.6 (C), 109.9 (CH), 108.1 (CH), 107.5 (CH), 104.7 (CH), 104.4 (CH), 56.5 (OCH_3), 56.3 (OCH_3), 53.8 (CH_2), 22.5 (CH_2), 21.0 (CH_2), 11.4 (CH_3), 10.9 (CH_3). **^{19}F NMR** (**282 MHz, CD_2Cl_2**)**:** δ = -152.2. **UV-Vis:** λ_{max} (CH_3CN) = 606 nm (ε = 11670 L.mol^{-1}.cm^{-1}).**IR (CH_2Cl_2, cm^{-1}):** ν = 3132, 2967, 1602, 1585, 1509, 1470, 1338, 1264, 1169, 1129, 1046, 896, 864, 816, 771, 732, 698, 640. **HRMS (ESI+)** calculated for [M+]: 556.2707 ($C_{35}H_{34}N_5O_2^+$), Found 556.2681.

1,13-dimethoxy-5,9-dipropyl-6-(4-(4-(trifluoromethyl)phenyl)-1H-1,2,3-triazol-1-yl)-5,9-dihydro-13bH-quinolino[2,3,4-kl]acridin-13b-ylium tetrafluoroborate (10b)

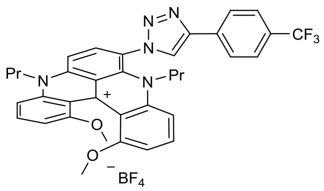

Prepared according to general procedure using 1-ethynyl-4-(trifluoromethyl)benzene (38 µL, 0.25 mmol). The reaction mixture was stirred for 24 h. Purification by selective precipitation with CH_2Cl_2 and Et_2O provided the product as a light green solid (35 mg, 99%).
R_f = 0.3 (SiO_2, CH_2Cl_2/MeOH, 98:2). **M. P.:** 257-259 °C (decomposition). **^{1}H NMR** (**400 MHz, CD_2Cl_2**)**:** δ = 8.66 (s, 1H, CH), 8.24 (d, *J* = 9.2 Hz, 1H, CH), 8.16 (d, *J* = 8.0 Hz, 2H, CH),

8.05 (t, J = 8.8 Hz, 1H, CH), 7.87 (t, J = 8.4 Hz, 1H, CH), 7.78 – 7.69 (m, 3H, CH), 7.53 (d, J = 8.9 Hz, 1H, CH), 7.30 (d, J = 8.6 Hz, 1H, CH), 6.99 (d, J = 8.1 Hz, 1H, CH), 6.91 (d, J = 8.1 Hz, 1H, CH), 4.81 – 4.71 (m, 1H, CH), 4.63 – 4.53 (m, 1H, CH), 4.27 – 4.19 (m, 1H, CH), 3.80 (s, 3H, OCH_3), 3.76 (s, 3H, OCH_3), 3.14 – 3.05 (m, 1H, CH), 2.33 – 2.15 (m, 2H, CH_2), 1.64 – 1.44 (m, 2H, CH_2), 1.29 (t, J = 7.4 Hz, 3H, CH_3), 0.38 (t, J = 7.3 Hz, 3H, CH_3). **^{13}C{^{1}H} NMR (101 MHz, CD_2Cl_2):** δ = 160.2 (C), 159.6 (C), 148.0 (C), 144.0 (C), 143.2 (C), 142.4 (C), 139.0 (C), 138.9 (CH), 137.6 (CH), 135.9 (C), 135.8 (CH), 134.1 (C), 134.1 (C), 130.6 (CF_3), 126.8 (CH), 126.5 (CH), 126.5 (CH), 123.4 (CH), 121.8 (C), 118.9 (C), 116.8 (C), 114.6 (C), 109.9 (CH), 108.1 (CH), 107.4 (CH), 104.7 (CH), 104.5 (CH), 56.5 (OCH_3), 56.3 (OCH_3), 53.9 (CH_2), 22.4 (CH_2), 21.0 (CH_2), 11.4 (CH_3), 10.8 (CH_3). **^{19}F NMR (282 MHz, CD_2Cl_2):** δ = -62.2, -152.0. **UV-Vis:** λ_{max} (CH_3CN) = 605 nm (ε = 12820 L.mol^{-1}.cm^{-1}). **IR (CH_2Cl_2, cm^{-1}):** ν = 3130, 2966, 1603, 1586, 1511, 1470, 1324, 1263, 1168, 1120, 1058, 815, 776, 736, 642. **HRMS (ESI+)** calculated for [M+]: 624.2581 ($C_{36}H_{33}F_3N_5O_2^+$), Found 624.2573.

6-(4-(4-(dimethylamino)phenyl)-1H-1,2,3-triazol-1-yl)-1,13-dimethoxy-5,9-dipropyl-5,9-dihydro-13bH-quinolino[2,3,4-kl]acridin-13b-ylium tetrafluoroborate (10c)

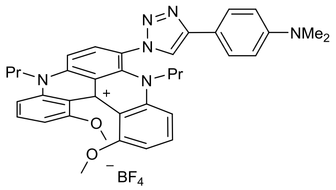

Prepared according to general procedure using 4-ethynyl-*N*,*N*-dimethylaniline (34 mg, 0.25 mmol). The reaction mixture was stirred for 24 h. Purification by selective precipitation with CH_2Cl_2 and Et_2O provided the product as a light green solid (34 mg, 99%).

R_f = 0.3 (SiO_2, CH_2Cl_2/MeOH, 98:2). **M. P.:** 208-210 °C (decomposition). **^{1}H NMR (400 MHz, CD_2Cl_2):** δ = 8.25 – 8.17 (m, 2H, CH), 8.05 (t, J = 8.5 Hz, 1H, CH), 7.85 (t, J = 8.3 Hz, 1H, CH), 7.80 (d, J = 8.5 Hz, 2H, CH), 7.70 (d, J = 9.3 Hz, 1H, CH), 7.52 (d, J = 9.0 Hz, 1H, CH), 7.28 (d, J = 8.7 Hz, 1H, CH), 7.00 (d, J = 8.1 Hz, 1H, CH), 6.90 (d, J = 8.2 Hz, 1H, CH), 6.80 (d, J = 8.4 Hz, 2H, CH), 4.80 – 4.70 (m, 1H, CH), 4.62 – 4.51 (m, 1H, CH), 4.26 – 4.18 (m, 1H, CH), 3.80 (s, 3H, OCH_3), 3.77 (s, 3H, OCH_3), 3.14 – 3.05 (m, 1H, CH), 3.00 (s, 6H, CH_3), 2.32 – 2.13 (m, 2H, CH_2), 1.65 – 1.43 (m, 2H, CH_2), 1.29 (t, J = 7.3 Hz, 3H, CH_3), 0.37 (t, J = 7.3 Hz, 3H, CH_3). **^{13}C{^{1}H} NMR (101 MHz, CD_2Cl_2):** δ = 160.2 (C), 159.6 (C), 151.4 (C), 150.0 (C), 144.0 (C), 143.2 (C), 142.4 (C), 138.9 (CH), 138.8 (C), 137.6 (CH), 135.7 (C), 135.7 (CH), 127.3 (CH), 121.8 (C), 120.2 (CH), 119.3 (C), 117.9 (C), 116.8 (C), 114.6 (C), 112.8 (CH), 109.8 (CH), 108.1 (CH), 107.4 (CH), 104.7 (CH), 104.4 (CH), 56.5 (OCH_3), 56.3 (OCH_3), 53.7 (CH_2), 40.7 (NCH_3), 22.5 (CH_2), 21.0 (CH_2), 11.4 (CH_3), 10.9 (CH_3). **^{19}F NMR (282 MHz, CD_2Cl_2):** δ = -152.3. **UV-Vis:** λ_{max} (CH_3CN) = 607 nm (ε = 11520 L.mol^{-1}.cm^{-1}). **IR (CH_2Cl_2, cm^{-1}):** ν = 3130, 2955, 1603, 1586, 1506, 1349, 1264, 1169, 1055, 946, 816, 778, 747, 644. **HRMS (ESI+)** calculated for [M+]: 599.3129 ($C_{37}H_{39}N_6O_2^+$), Found 599.3155.

6-cyano-1,13-dimethoxy-5,9-dipropyl-5,9-dihydro-13bH-quinolino[2,3,4-kl]acridin-13b-ylium tetrafluoroborate (11)

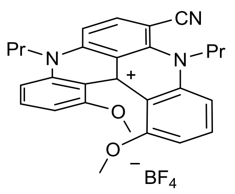

To a solution of salt **6** (25 mg, 0.05 mmol) and NaN_3 (4.6 mg, 0.07 mmol) in MeCN (95 µL, 0.05 M), was added TfOH (12.53 µL, 0.14 mmol). The resulting mixture was stirred for 5 min at 25 °C (monitored by ESI-MS). Then, the mixture was extracted with CH_2Cl_2 (3 x 10 mL) and the organic layer was washed with HBF_4 1 M in H_2O, dried over Na_2SO_4, filtered and evaporated under *vacuum*. The crude material was purified by selective precipitation with CH_2Cl_2 and Et_2O and flash chromatography on silica gel (CH_2Cl_2/MeOH, 98:2). The desired product is obtained as a purple solid (17.4 mg, 70%).

R_f = 0.3 (SiO_2, CH_2Cl_2/MeOH, 98:2). **M. P.:** 261-263 °C (decomposition). **^{1}H NMR** (**400 MHz, CD_2Cl_2**)**:** δ = 8.35 (d, *J* = 9.1 Hz, 1H, CH), 8.08 (t, *J* = 8.4, 1H, CH), 7.98 (t, *J* = 8.4, 1H, CH), 7.71 (d, *J* = 9.1 Hz, 1H, CH), 7.60 (d, *J* = 8.9 Hz, 1H, CH), 7.50 (d, *J* = 9.2 Hz, 1H), 7.04 (d, *J* = 8.1 Hz, 1H), 6.97 (d, *J* = 8.2 Hz, 1H), 5.01 – 4.95 (m, 2H, CH), 4.86 – 4.77 (m, 1H, CH), 4.65 – 4.56 (m, 1H, CH), 3.80 (s, 3H, OCH_3), 3.77 (s, 3H, OCH_3), 2.25 – 2.13 (m, 2H, CH_2), 2.10 – 1.98 (m, 2H, CH_2), 1.28 (t, *J* = 7.4 Hz, 3H, CH_3), 0.84 (t, *J* = 7.3 Hz, 3H, CH_3). **^{13}C{^{1}H} NMR** (**101 MHz, CD_2Cl_2**)**:** δ = 160.1 (C), 159.7 (C), 142.8 (C), 142.7 (C), 142.3 (C), 142.2 (CH), 141.9 (C), 141.3 (C), 139.3 (CH), 138.3 (CH), 120.0 (C), 118.5 (C), 115.9 (C), 114.5 (C), 109.3 (CH), 108.3 (CH), 107.7 (CH), 105.3 (CH), 105.0 (CH), 91.0 (CN), 56.6 (OCH_3), 56.5 (OCH_3), 55.4 (CH_2), 22.8 (CH_2), 21.0 (CH_2), 11.4 (CH_3), 10.6 (CH_3). **^{19}F NMR** (**282 MHz, CD_2Cl_2**)**:** δ = -152.0. **UV-Vis:** λ_{max} (CH_3CN) = 585 nm (ε = 12880 $L.mol^{-1}.cm^{-1}$). **IR (CH_2Cl_2, cm^{-1}):** ν = 3097, 2932, 2217, 1588, 1501, 1466, 1324, 1260, 1132, 1027, 864, 814, 781, 633. **HRMS (ESI+)** calculated for [M+]: 438.2176 ($C_{28}H_{28}N_3O_2^+$), Found 438.2170.

6-(2,2-dicyanovinyl)-1,13-dimethoxy-5,9-dipropyl-5,9-dihydro-13bH-quinolino[2,3,4-kl]acridin-13b-ylium tetrafluoroborate (12)

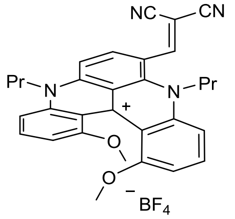

To a solution of salt **3** (25 mg, 0.05 mmol) and malononitrile (9.4 mg, 0.14 mmol) in MeCN (10 µL, 3 M), was added Ph_3P (2.5 mg, 0.01 mmol). The obtained mixture was heated in the MW at 130 °C. The crude material was purified by selective precipitation with CH_2Cl_2 and Et_2O. The desired product is obtained as a green solid (25.6 mg, 94%).

R_f = 0.3 (SiO_2, CH_2Cl_2/MeOH, 98:2). **M. P.:** 283-285 °C (decomposition). **^{1}H NMR** (**400 MHz, CD_2Cl_2**)**:** δ = 8.70 (d, *J* = 9.3 Hz, 1H, CH), 8.16 – 8.06 (m, 2H, CH), 7.97 (t, *J* = 8.4 Hz, 1H, CH), 7.68 (d, *J* = 9.4 Hz, 1H, CH), 7.60 – 7.53 (m, 2H, CH), 7.07 (d, *J* = 8.1 Hz, 1H, CH), 6.98 (d, *J* = 8.2 Hz, 1H, CH), 5.19 – 5.08 (m, 1H, CH), 4.86 – 4.73 (m, 1H, CH), 4.67 – 4.54 (m, 1H, CH), 4.21 – 4.10 (m, 1H, CH), 3.82 (s, 3H, CH_3), 3.78 (s, 3H, CH_3), 2.30 – 2.15 (m, 2H, CH_2), 1.80 – 1.69 (m, 2H, CH_2), 1.30 (t, *J* = 7.4 Hz, 3H, CH_3), 0.43 (t, *J* = 7.3 Hz, 3H, CH_3). **^{13}C{^{1}H} NMR** (**101 MHz, CD_2Cl_2**)**:** δ 160.3 (C), 159.6 (C), 156.5 (CH), 143.4 (C), 142.6 (C), 142.3 (C), 141.8 (C), 140.9 (C), 139.5 (CH), 137.9 (CH), 137.4 (CH), 120.3 (C), 117.2 (C), 114.7 (C), 114.3 (C), 113.3 (C), 112.8 (C), 111.1 (C), 108.2 (CH), 108.1 (CH), 105.6 (CH), 105.4 (CH), 82.2 (CH),

60.9 (CH_2), 56.7 (OCH_3), 56.5 (OCH_3), 23.2 (CH_2), 21.3 (CH_2), 11.4 (CH_3), 10.8 (CH_3). **^{19}F NMR** (**282 MHz, CD_2Cl_2**)**:** δ = -152.5. **UV-Vis:** λ_{max} (CH_3CN) = 598 nm (ε = 11550 $L.mol^{-1}.cm^{-1}$). **IR (CH_2Cl_2, cm^{-1}):** ν = 3120, 2968, 2227, 1583, 1503, 1469, 1340, 1287, 1265, 1235, 1117, 1125, 1050, 955, 818, 778, 709, 616. **HRMS (ESI+)** calculated for [M+]: 489.2285 ($C_{31}H_{29}N_4O_2^+$), Found 489.2285.

General procedure for the synthesis of styryl quinacridinium tetrafluoroborate salts [13a-c,e-f].

To a solution of Ph_3P (1.6 equiv) in CH_2Cl_2 (0.1 M), was added the corresponding benzyl chloride (1.6 equiv). The reaction mixture was stirred for 30 min. Full conversion to the corresponding phosponium salt was confirmed by ESI-MS. Then, **6** (25 mg, 0.05 mmol) and NaH (2 equiv) were added to the reaction mixture at 0 °C. The reaction was allowed to reach 25 °C and stirred for 30 min to 4 h (monitored by ESI-MS). Then, the mixture was extracted with CH_2Cl_2 (3 x 10 mL) and the organic layer was washed with HBF_4 1 M in H_2O, dried over Na_2SO_4, filtered and evaporated under *vacuum*. The crude product was precipitated in Et_2O. The crude precipitate was filtered and purified by dissolving it in a minimum amount of CH_2Cl_2 and then the addition of pentane led to the precipitation of the desired product, which was separated from the mother liquor by centrifugation. Desired products were further purified by flash chromatography (CH_2Cl_2/MeOH, 98:2) as light green solids.

(*E*)-1,13-dimethoxy-5,9-dipropyl-6-styryl-5,9-dihydro-13bH-quinolino[2,3,4-kl]acridin-13b-ylium tetrafluoroborate (13a)

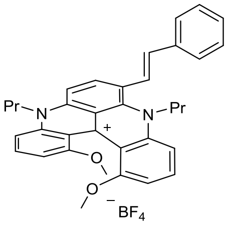

Prepared according to general procedure using benzyl bromide (13.7 mg, 0.08 mmol). The reaction mixture was stirred for 30 min after addition of the aldehyde and NaH. Purification by selective precipitation with CH_2Cl_2 and pentane and flash chromatography provided the product as a light green solid (28.0 mg, 98%).

R_f = 0.5 (SiO_2, CH_2Cl_2/MeOH, 98:2). **M. P.:** 170-172 °C (decomposition). **^{1}H NMR** (**400 MHz, CD_2Cl_2**)**:** δ = 8.37 (d, *J* = 8.0 Hz 1H, CH), 7.98 (t, *J* = 8.5 Hz, 1H, CH), 7.86 (t, *J* = 8.3 Hz, 1H, CH), 7.69 – 7.60 (m, 3H, CH), 7.47 – 7.42 (m, 4H, CH), 7.37 – 7.26 (m, 3H, CH), 6.94 (d, *J* = 8.1 Hz, 1H), 6.85 (d, *J* = 8.1 Hz, 1H), 4.85 – 4.66 (m, 2H, CH), 4.61 – 4.47 (m, 2H, CH), 3.80 (s, 3H, CH_3), 3.76 (s, 3H, CH_3), 2.32 – 2.09 (m, 2H, CH_2), 1.69 – 1.61 (m, 2H, CH_2), 1.29 (t, *J* = 7.4 Hz, 3H, CH_3), 0.39 (t, *J* = 7.3 Hz, 3H, CH_3). **^{13}C{^{1}H} NMR** (**101 MHz, CD_2Cl_2**)**:** δ = 160.2 (C), 159.7 (C), 144.9 (C), 142.8 (C), 142.2 (C), 139.4 (C), 138.1 (CH), 137.5 (C), 137.3 (CH), 136.9 (CH), 131.1 (CH), 129.5 (CH), 129.0 (CH), 127.2 (CH), 125.8 (CH), 121.9 (C), 120.5 (C), 117.1 (C), 114.4 (C), 110.4 (CH), 108.0 (CH), 107.4 (CH), 104.1 (CH), 103.8 (CH), 57.0 (CH_2), 56.4 (OCH_3), 56.2 (OCH_3), 22.8 (CH_2), 21.1 (CH_2), 11.4 (CH_3), 10.9 (CH_3). **^{19}F NMR** (**282 MHz, CD_2Cl_2**)**:** δ = -152.5. **UV-Vis:** λ_{max} (CH_3CN) = 638 nm (ε = 6710 $L.mol^{-1}.cm^{-1}$). **IR (CH_2Cl_2, cm^{-1}):** ν = 2965, 2878, 1587, 1502, 1469, 1340, 1256, 1170, 1127, 1052, 816, 779, 695. **HRMS (ESI+)** calculated for [M+]: 515.2693 ($C_{35}H_{35}N_2O_2^+$), Found 515.2693.

(*E*)-1,13-dimethoxy-6-(4-nitrostyryl)-5,9-dipropyl-5,9-dihydro-13bH-quinolino[2,3,4-kl]acridin-13b-ylium tetrafluoroborate (13b)

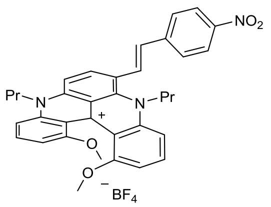

Prepared according to general procedure using *p*-NO_2-benzyl bromide (17.3 mg, 0.08 mmol). The reaction mixture was stirred for 4 h after addition of the aldehyde and NaH. Purification by selective precipitation with CH_2Cl_2 and pentane and flash chromatography provided the product as a light green solid (19.3 mg, 63%).

R_f = 0.5 (SiO_2, CH_2Cl_2/MeOH, 98:2). **M. P.:** 167-169 °C (decomposition). **^{1}H NMR (400 MHz, CD_2Cl_2):** δ = 8.41 (d, *J* = 9.1 Hz, 1H), 8.28 – 8.25 (m, 2H, CH), 7.99 (t, *J* = 8.9, 8.0 Hz, 1H, CH), 7.89 (t, *J* = 8.4 Hz, 1H, CH), 7.83 – 7.76 (m, 2H, CH), 7.67 (d, *J* = 9.1 Hz, 1H, CH), 7.57 – 7.45 (m, 3H, CH), 7.35 (d, *J* = 16.2 Hz, 1H, CH), 6.95 (d, *J* = 8.0 Hz, 1H, CH), 6.87 (d, *J* = 8.1 Hz, 1H, CH), 4.93 – 4.84 (m, 1H, CH), 4.78 – 4.67 (m, 1H, CH), 4.61 – 4.41 (m, 2H, CH), 2.33 – 2.11 (m, 2H, CH_2), 1.73 – 1.58 (m, 2H, CH_2), 1.29 (t, *J* = 7.4 Hz, 3H, CH_3), 0.38 (t, *J* = 7.3 Hz, 3H, CH_3). **^{13}C{^{1}H} NMR (101 MHz, CD_2Cl_2):** δ = 160.2 (C), 159.7 (C), 147.8 (C), 144.6 (C), 143.8 (C), 142.8 (C), 142.2 (C), 140.1 (C), 138.3 (CH), 138.0 (C), 137.4 (CH), 137.1 (CH), 130.1 (CH), 128.5 (CH), 127.8 (CH), 124.8 (CH), 121.7 (C), 119.4 (C), 117.1 (C), 114.5 (C) 110.5 (C), 108.0 (CH), 107.6 (CH), 104.3 (CH), 104.0 (CH), 57.7 (CH_2), 56.5 (OCH_3), 56.2 (OCH_3), 22.8 (CH_2), 21.1 (CH_2), 11.5 (CH_3), 10.9 (CH_3). **^{19}F NMR (282 MHz, CD_2Cl_2):** δ = -152.4. **UV-Vis:** λ_{max} (CH_3CN) = 638 nm (ε = 8090 L.mol^{-1}.cm^{-1}). **IR (CH_2Cl_2, cm^{-1}):** ν = 2965, 2878, 1585, 1510, 1470, 1338, 1258, 1170, 1127, 1055, 817, 779, 745. **HRMS (ESI+)** calculated for [M+]: 560.2544 ($C_{35}H_{34}N_3O_4^+$), Found 515.2543.

(*E*)-1,13-dimethoxy-5,9-dipropyl-6-(4-(trifluoromethyl)styryl)-5,9-dihydro-13bH-quinolino[2,3,4-kl]acridin-13b-ylium tetrafluoroborate (13c)

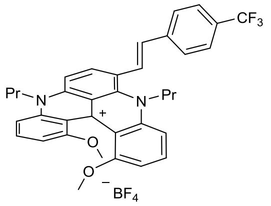

Prepared according to general procedure using *p*-CF_3-benzyl bromide (19.1 mg, 0.08 mmol). The reaction mixture was stirred for 4 h after addition of the aldehyde and NaH. Purification by selective precipitation with CH_2Cl_2 and pentane and flash chromatography provided the product as a light green solid (15.2 mg, 55%).

R_f = 0.3 (SiO_2, CH_2Cl_2/MeOH, 98:2). **M. P.:** 271-273 °C (decomposition). **^{1}H NMR (400 MHz, CD_2Cl_2):** δ = 8.39 (d, *J* = 9.0 Hz, 1H, CH), 7.98 (t, *J* = 9.0, 8.0 Hz, 1H, CH), 7.87 (t, *J* = 8.4 Hz, 1H, CH), 7.77 (d, *J* = 8.2 Hz, 2H, CH), 7.73 – 7.62 (m, 3H, CH), 7.51 – 7.40 (m, 3H, CH), 7.32 (d, *J* = 16.2 Hz, 1H, CH), 6.95 (d, *J* = 8.0 Hz, 1H, CH), 6.86 (d, *J* = 8.1 Hz, 1H, CH), 4.88 – 4.79 (m, 1H, CH), 4.78 – 4.66 (m, 1H, CH), 4.60 – 4.44 (m, 2H, CH), 3.80 (s, 3H, CH_3), 3.76 (s, 3H, CH_3), 2.35 – 2.10 (m, 2H, CH_2), 1.73 – 1.57 (m, 2H, CH_2), 1.28 (t, 3H, CH_3), 0.39 (t, *J* = 7.3 Hz, 3H, CH_3). **^{13}C{^{1}H} NMR (101 MHz, CD_2Cl_2):** δ = 160.2 (C), 159.7 (C), 144.8 (C), 142.8 (C), 142.2 (C), 141.0 (C), 139.8 (C), 138.3 (CH), 137.8 (C), 137.4 (CH), 137.0 (CH), 130.2 (CF_3), 129.4 (CH), 128.4 (CH), 127.4 (CH), 121.8 (C), 119.8 (C), 117.1 (C), 114.5 (C) 110.4 (CH), 108.0 (CH), 107.5 (CH), 104.2 (CH), 103.9 (CH), 57.4 (CH_2), 56.5 (OCH_3), 56.2 (OCH_3), 22.8 (CH_2), 21.1 (CH_2), 11.5 (CH_3), 10.9 (CH_3). **^{19}F NMR (282 MHz, CD_2Cl_2):** δ = -62.1, -152.5. **UV-Vis:** λ_{max} (CH_3CN) = 640 nm (ε = 8810 L.mol^{-1}.cm^{-1}). **IR (CH_2Cl_2, cm^{-1}):** ν = 2960, 2878, 1591, 1504, 1470, 1323,

1256, 1169, 1124, 1066, 817, 779, 750. **HRMS (ESI+)** calculated for [M+]: 583.2567 ($C_{36}H_{34}F_3N_2O_4^+$), Found 583.2586.

(*E*)-6-(4-aminostyryl)-1,13-dimethoxy-5,9-dipropyl-5,9-dihydro-13bH-quinolino[2,3,4-kl]acridin-13b-ylium tetrafluoroborate (13d)

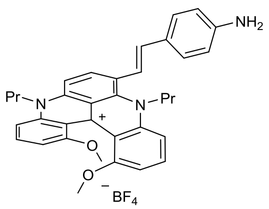

To a solution of **13b** (15 mg, 0.02 mmol) and CH_3COOH (20.0 µl, 0.35 mmol) in CH_2Cl_2 (20.0 µl, 1.2 M), was added Zn powder (8.0 mg, 0.12 mmol). The reaction mixture was stirred for 3 h at 25 °C. Purification by selective precipitation with CH_2Cl_2 and pentane and flash chromatography provided the product as a light green solid (14 mg, 98%).

R_f = 0.5 (SiO_2, CH_2Cl_2/MeOH, 98:2). **M. P.:** 290-292 °C (decomposition). **^{1}H NMR** (**400 MHz, CD_2Cl_2**)**:** δ = 8.32 (d, *J* = 9.0 Hz, 1H, CH), 7.95 (t, *J* = 8.9, 8.0 Hz, 1H, CH), 7.83 (t, *J* = 8.4 Hz, 1H, CH), 7.59 (d, *J* = 9.0 Hz, 1H, CH), 7.49 – 7.36 (m, 4H, CH), 7.16 (d, *J* = 16.1 Hz, 1H, CH), 7.05 (d, *J* = 16.2 Hz, 1H, CH), 6.92 (d, *J* = 8.0 Hz, 1H, CH), 6.83 (d, *J* = 8.1 Hz, 1H, CH), 6.78 – 6.67 (m, 2H, CH), 4.80 – 4.63 (m, 2H, CH), 4.62 – 4.43 (m, 2H, CH), 3.79 (s, 3H, CH_3), 3.75 (s, 3H, CH_3), 3.55 – 2.81 (m, 2H, NH_2), 2.31 – 2.08 (m, 2H, CH_2), 1.70 – 1.51 (m, 2H, CH_2), 1.28 (t, *J* = 7.3 Hz, 3H, CH_3), 0.38 (t, *J* = 7.3 Hz, 3H, CH_3). **^{13}C{^{1}H} NMR** (**101 MHz, CD_2Cl_2**)**:** δ = 160.2 (C), 159.7 (C), 148.2 (C), 145.3 (C), 142.9 (C), 142.2 (C), 138.9 (C), 137.9 (CH), 137.1 (CH), 137.0 (C), 136.8 (CH), 131.4 (CH), 128.6 (CH), 127.3 (C), 122.1 (C), 121.6 (CH), 121.5 (C), 117.1 (C), 115.5 (CH), 114.4 (C), 110.3 (CH), 108.0 (CH), 107.3 (CH), 104.0 (CH), 103.6 (CH), 56.5 (CH_2), 56.4 (OCH_3), 56.6 (OCH_3), 22.7 (CH_2), 21.0 (CH_2), 11.5 (CH_3), 10.9 (CH_3). **^{19}F NMR** (**282 MHz, CD_2Cl_2**)**:** δ = -151.5. **UV-Vis:** λ_{max} (CH_3CN) = 660 nm (ε = 5600 L.mol^{-1}.cm^{-1}). **IR (CH_2Cl_2, cm^{-1}):** ν = 3380, 2935, 2878, 1580, 1512, 1466, 1339, 1254, 1168, 1126, 1038, 814, 775, 730. **HRMS (ESI+)** calculated for [M+]: 530.2802 ($C_{35}H_{36}N_3O_2^+$), Found 530.2804.

(*E*)-6-(3,5-bis(trifluoromethyl)styryl)-1,13-dimethoxy-5,9-dipropyl-5,9-dihydro-13bH-quinolino[2,3,4-kl]acridin-13b-ylium tetrafluoroborate (13e)

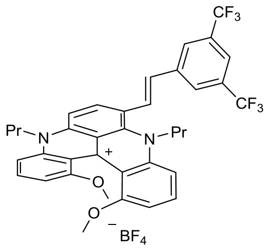

Prepared according to general procedure using *m*-di(CF_3)-benzyl chloride (21.0 mg, 0.08 mmol). The reaction mixture was stirred for 2 h after addition of the aldehyde and NaH. Purification by selective precipitation with CH_2Cl_2 and pentane and flash chromatography provided the product as a light green solid (22.7 mg, 65%).

R_f = 0.4 (SiO_2, CH_2Cl_2/MeOH, 98:2). **M. P.:** 223-225 °C (decomposition). **^{1}H NMR** (**400 MHz, CD_2Cl_2**)**:** δ = 8.38 (d, *J* = 9.1 Hz, 1H, CH), 8.07 (s, 2H, CH), 8.00 (t, *J* = 8.9, 8.0 Hz, 1H, CH), 7.92 – 7.84 (m, 2H, CH), 7.68 (d, *J* = 9.1 Hz, 1H, CH), 7.52 – 7.44 (m, 3H, CH), 7.35 (d, *J* = 16.2 Hz, 1H, CH), 6.96 (d, *J* = 8.0 Hz, 1H, CH), 6.87 (d, *J* = 8.1 Hz, 1H, CH), 4.89 – 4.79 (m, 1H, CH), 4.79 – 4.67 (m, 1H, CH), 4.62 – 4.40 (m, 2H, CH), 3.80 (s, 3H, CH_3), 3.76 (s, 3H, CH_3), 2.33 – 2.11 (m, 2H, CH_2), 1.72 – 1.58 (m, 2H, CH_2), 1.29 (t, *J* = 7.4 Hz, 3H, CH_3), 0.39 (t, *J* = 7.3 Hz, 3H, CH_3). **^{13}C{^{1}H} NMR** (**101 MHz,**

CD_2Cl_2): δ = 160.2 (C), 159.8 (C), 144.6 (C), 142.8 (C), 142.2 (C), 140.0 (C), 139.7 (C), 138.4 (CH), 138.1 (C), 137.6 (CH), 137.1 (CH), 132.7 (CF_3), 129.7 (CH), 127.9 (CH), 127.1 (CH), 125.3 (C), 122.6 (C), 121.7 (C), 119.2 (C), 117.1 (C), 114.5 (C), 110.4 (CH), 108.0 (CH), 107.7 (CH), 104.3 (CH), 104.0 (CH), 57.6 (CH_2), 56.5 (OCH_3), 56.2 (OCH_3), 22.8 (CH_2), 21.1 (CH_2), 11.5 (CH_3), 10.9 (CH_3). **^{19}F NMR** (**282 MHz, CD_2Cl_2**) δ = -62.6, -152.5. **UV-Vis:** λ_{max} (CH_3CN) = 636 nm (ε = 8750 L.mol^{-1}.cm^{-1}). **IR (CH_2Cl_2, cm^{-1}):** ν = 2967, 2881, 1585, 1502, 1468, 1376, 1277, 1169, 1125, 1039, 941, 894, 816, 778, 732. **HRMS (ESI+)** calculated for [M+]: 651.2441 ($C_{37}H_{33}F_6N_2O_2^+$), Found 651.2415.

(*E*)-6-(3,5-dimethoxystyryl)-1,13-dimethoxy-5,9-dipropyl-5,9-dihydro-13bH-quinolino[2,3,4-kl]acridin-13b-ylium tetrafluoroborate (13f)

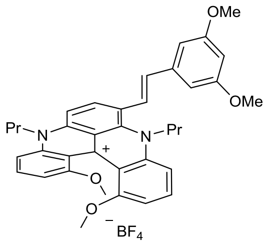

Prepared according to general procedure using *m*-di(OMe)-benzyl bromide (14.1 mg, 0.08 mmol). The reaction mixture was stirred for 2 h after addition of the aldehyde and NaH. Purification by selective precipitation with CH_2Cl_2 and pentane and flash chromatography provided the product as a light green solid (21.9 mg, 70%).

R_f = 0.5 (SiO_2, CH_2Cl_2/MeOH, 98:2). **M. P.:** 186-188 °C (decomposition). **^{1}H NMR** (**400 MHz, CD_2Cl_2**): δ = 8.35 (d, *J* = 9.0 Hz, 1H, CH), 7.97 (t, *J* = 8.0 Hz, 1H, CH), 7.86 (t, *J* = 8.4 Hz, 1H, CH), 7.64 (d, *J* = 9.0 Hz, 1H, CH), 7.49 – 7.40 (m, 2H, CH), 7.29 (d, *J* = 16.2 Hz, 1H, CH), 7.19 (d, *J* = 16.1 Hz, 1H, CH), 6.94 (d, *J* = 8.1 Hz, 1H, CH), 6.85 (d, *J* = 8.1 Hz, 1H, CH), 6.77 (s, 2H, CH), 6.47 (s, 1H, CH), 4.84 – 4.65 (m, 2H, CH), 4.59 – 4.47 (m, 2H, CH), 3.85 (s, 6H, CH_3), 3.80 (s, 3H, CH_3), 3.76 (s, 3H, CH_3), 2.31 – 2.10 (m, 2H, CH_2), 1.71 – 1.52 (m, 2H, CH_2), 1.29 (t, *J* = 7.4 Hz, 3H, CH_3), 0.38 (t, *J* = 7.3 Hz, 3H, CH_3). **^{13}C{^{1}H} NMR** (**101 MHz, CD_2Cl_2**): δ = 161.8 (C), 160.2 (C), 159.7 (C), 144.9 (C), 142.8 (C), 142.2 (C), 139.4 (C), 139.3 (C), 138.1 (CH), 137.5 (C), 137.4 (CH), 136.9 (CH), 131.1 (CH), 126.3 (CH), 121.8 (C), 120.3 (C), 117.0 (C), 114.4 (C), 110.4 (CH), 108.0 (CH), 107.5 (CH), 105.4 (CH), 104.1 (CH), 103.8 (CH), 100.7 (CH), 57.0 (CH_2), 56.4 (OCH_3), 56.2 (OCH_3), 56.0 (OCH_3) , 22.8 (CH_2), 21.1 (CH_2), 11.4 (CH_3), 10.9 (CH_3). **^{19}F NMR** (**282 MHz, CD_2Cl_2**): δ = -152.5. **UV-Vis:** λ_{max} (CH_3CN) = 644 nm (ε = 7960 L.mol^{-1}.cm^{-1}). **IR (CH_2Cl_2, cm^{-1}):** ν = 2938, 2879, 1582, 1500, 1463, 1320, 1260, 1169, 1152, 1040, 815, 777, 731. **HRMS (ESI+)** calculated for [M+]: 575.2904 ($C_{37}H_{39}N_2O_4^+$), Found 579.2886.

6-hydroxy-1,13-dimethoxy-5,9-dipropyl-5,9-dihydro-13bH-quinolino[2,3,4-kl]acridin-13b-ylium tetrafluoroborate (14)

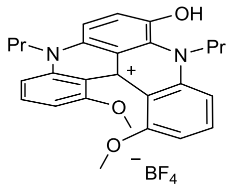

Compound **6** (400 mg, 0.76 mmol, 1 equiv) was dissolved in 5 mL of CH_2Cl_2. To this solution was added 3-chloroperbenzoic acid (653 mg, 3.79 mmol, 5 equiv) and the reaction was stirred for 2 h at 25 °C. The crude mixture was diluted in CH_2Cl_2 and washed twice with an aqueous saturated solution of $NaHCO_3$, an aqueous diluted solution of HBF_4, then dried over Na_2SO_4, filtered and finally evaporated. Several

purifications on neutral alumina (CH_2Cl_2/MeOH, 95:5) afforded the pure product as a green solid (306 mg, 78%).

R_f = 0.4 (Neutral alumina, CH_2Cl_2/MeOH, 95:5). **M. P.:** 154-156 °C (decomposition: 176-178 °C). **^{1}H NMR (CD_2Cl_2, 500 MHz):** δ = 11.63 (s, 1H, OH), 8.69 (d, *J* = 9.2 Hz, 1H, CH), 7.78 (t, *J* = 8.9 Hz, 1H, CH), 7.68 (t, *J* = 8.8 Hz, 1H, CH), 7.38 (d, *J* = 9.2 Hz, 1H, CH), 7.31 (dd, *J* = 8.9,1.0 Hz, 1H, CH), 7.25 (dd, *J* = 9.2, 1.0 Hz, 1H, CH), 6.73 (d, *J* = 8.1 Hz, 1H, CH), 6.65 (dd, *J* = 8.1, 1.0 Hz, 1H, CH), 5.08 – 5.02 (m, 1H, CH_2), 4.64 – 4.58 (m, 1H, CH_2), 4.55 – 4.48 (m,1H, CH_2), 4.36 – 4.23 (m, 1H, CH_2), 3.72 (s, 3H, OCH_3), 3.67 (s, 3H, OCH_3), 2.17 – 2.01 (m, 2H, CH_2), 1.94 – 1.76 (m, 2H, CH_2), 1.20 (t, *J* = 7.3 Hz, 3H, CH_3), 0.74 (t, *J* = 7.3 Hz, 3H, CH_3). **^{13}C{^{1}H} NMR (CD_2Cl_2, 125 MHz):** δ = 159.8 (C), 159.4 (C), 146.6 (C), 143.3 (C), 142.5 (C), 141.9 (C), 136.5 (CH), 136.2 (CH), 130.9 (C), 127.1 (C), 127.1 (CH), 123.2 (C), 115.8 (C), 113.4 (C), 109.1 (CH), 107.5 (CH), 106.0 (CH), 102.3 (CH), 102.2 (CH), 55.9 (OCH_3), 55.7 (OCH_3), 53.7 (CH_2), 52.0 (CH_2), 23.3 (CH_2), 20.4 (CH_2), 11.3 (CH_3), 11.2 (CH_3). **^{19}F NMR (CD_2Cl_2, 282 MHz):** δ = -150.7. **UV-Vis:** λ_{max} (CH_3CN) = 659 nm (ε = 7450 L.mol^{-1}.cm^{-1}). **IR (neat, cm^{-1}):** ν = 3367, 2940, 1604, 1557, 1500, 1459, 1365, 1304, 1257, 1163, 1127, 1046, 809, 770, 734, 717. **HRMS (ESI+)** calculated for $[M+H]^+$: 429.2173 ($C_{27}H_{29}N_2O_3^+$), Found 429.2159.

1,6,13-trimethoxy-5,9-dipropyl-5,9-dihydro-13bH-quinolino[2,3,4-kl]acridin-13b-ylium tetrafluoroborate (15)

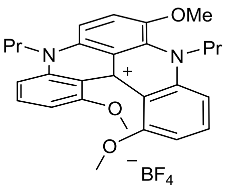

Compound **14** (30 mg, 0.06 mmol, 1 equiv) was dissolved in 2 mL of acetonitrile. To this solution was added K_2CO_3 (40 mg, 0.29 mmol, 5 equiv) and MeI (11 µL, 0.17 mmol, 3 equiv). The reaction was refluxed for 1 h then the crude mixture was diluted in CH_2Cl_2 and washed with an aqueous diluted solution of HBF_4, dried over Na_2SO_4, filtered and finally evaporated. Purification by silica column chromatography (CH_2Cl_2/MeOH, 95:5) afforded the pure product as a green solid (28 mg, 90%).

R_f = 0.45 (SiO_2, CH_2Cl_2/MeOH, 95:5). **M. P.:** 230-232 °C (decomposition). **^{1}H NMR (CD_2Cl_2/CD_3OD 1/1, 500 MHz):** δ = 7.98 (d, *J* = 9.3 Hz, 1H, CH), 7.90 (t, *J* = 8.8 Hz, 1H, CH), 7.81 (t, *J* = 8.8 Hz, 1H, CH), 7.63 (d, *J* = 9.3 Hz, 1H, CH), 7.45 (d, *J* = 8.7 Hz, 1H, CH), 7.42 (d, *J* = 8.9 Hz, 1H, CH), 6.88 (d, *J* = 7.8 Hz, 1H, CH), 6.82 (d, *J* = 8.0 Hz, 1H, CH), 4.85 – 4.81 (m, 1H, CH_2), 4.72 – 4.63 (m, 2H, CH_2), 4.49 – 4.43 (m, 1H, CH_2), 4.11 (s, 3H, OCH_3), 3.76 (s, 3H, OCH_3), 3.72 (s, 3H, OCH_3), 2.23 – 2.06 (m, 2H, CH_2), 2.89 – 1.78 (m, 2H, CH_2), 1.23 (t, *J* = 7.4 Hz, 3H, CH_3), 0.73 (t, *J* = 7.4 Hz, 3H, CH_3). **^{13}C{^{1}H} NMR (CD_2Cl_2/CD_3OD 1/1, 125 MHz):** δ = 160.4 (C), 160.0 (C), 146.6 (C), 143.9 (C), 143.1 (C), 143.0 (C), 138.1 (CH), 137.4 (CH), 133.0 (C), 129.5 (C), 122.8 (C), 122.3 (CH), 116.5 (C), 114.1 (C), 109.5 (CH), 108.1 (CH), 106.9 (CH), 103.5 (CH), 103.4 (CH), 57.8 (OCH_3), 56.2 (OCH_3), 56.0 (OCH_3), 55.2 (CH_2), 52.4 (CH_2), 23.5 (CH_2), 20.8 (CH_2), 11.36 (CH_3), 11.2 (CH_3). **^{19}F NMR (CD_2Cl_2, 282 MHz):** δ = -152.6. **UV-Vis:** λ_{max} (CH_3CN) = 653 nm (ε = 7750 L.mol^{-1}.cm^{-1}). **IR (neat, cm^{-1}):** ν = 3107, 2953, 1605, 1579, 1559, 1518, 1497, 1470, 1365, 1330,

1260, 1211, 1186, 1164, 1130, 1085, 1038, 995, 964, 903, 862, 808, 793, 775, 735, 714, 675, 626. **HRMS (ESI+)** calculated for [M+]: 443.2329 ($C_{28}H_{31}N_2O_3^+$), Found 443.2326.

1,13-dimethoxy-5,9-dipropyl-5,9-dihydroquinolino[2,3,4-kl]acridin-13b-ylium-6-carboxylate (16)

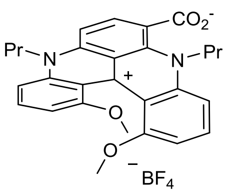

Compound **6** (500 mg, 0.95 mmol, 1 equiv) was dissolved in 20 mL of acetonitrile. To this solution was added separately NaH_2PO_4 (113 mg, 0.95 mmol, 1 equiv), 1 mL of H_2O_2 and $NaClO_2$ (171 mg, 1.89 mmol, 2 equiv). The reaction was stirred for 1 h at 60 °C, then concentrated under reduced pressure. The residue was diluted in CH_2Cl_2 and washed with aqueous $NaBF_4$ (0.2 M), water, dried over Na_2SO_4, filtered and finally evaporated. Purification by silicagel column chromatography (CH_2Cl_2/MeOH, 90:10) afforded the pure product as a green solid (390 mg, 76%).

R_f = 0.32 (SiO_2, CH_2Cl_2/MeOH, 90:10). **M. P.:** 179-181 °C (decomposition). **^{1}H NMR (CD_3OD, 500 MHz):** δ = 8.50 (d, *J* = 8.7 Hz, 1H, CH), 7.98 (t, *J* = 8.4 Hz, 1H, CH), 7.85 (t, *J* = 8.4 Hz, 1H, CH), 7.64 – 7.53 (m, 3H, CH), 7.05 (d, *J* = 8.0 Hz, 1H, CH), 6.94 (d, *J* = 8.0 Hz, 1H, CH), 5.00 – 4.97 (m, 1H, CH_2), 4.76 – 4.70 (m, 1H, CH_2), 4.53 – 4.48 (m, 1H, CH_2), 4.30 – 4.24 (m, 1H, CH_2), 3.79 (s, 3H, OCH_3), 3.73 (s, 3H, OCH_3), 2.15 – 2.07 (m, 2H, CH_2), 1.73 – 1.69 (m, 2H, CH_2), 1.20 (t, *J* = 7.3 Hz, 3H, CH_3), 0.42 (t, *J* = 7.3 Hz, 3H, CH_2). **^{13}C{^{1}H} NMR (CD_3OD, 125 MHz):** δ = 173.7 (C), 160.9 (C), 160.4 (C), 143.7 (C), 142.9 (C), 142.2 (C), 140.2 (C), 139.6 (C), 139.4 (CH), 138.6 (CH), 137.4 (CH), 121.6 (C), 121.5 (C), 116.9 (C), 114.4 (C), 111.2 (CH), 108.8 (CH), 106.4 (CH), 104.7 (CH), 104.3 (CH), 58.22 (CH_2), 56.5 (OCH_3), 56.2 (OCH_3), 52.7 (CH_2), 23.1 (CH_2), 21.2 (CH_2), 11.1 (CH_3), 10.8 (CH_3). **UV-Vis:** λ_{max} (CH_3CN) = 626 nm (ε = 10400 $L.mol^{-1}.cm^{-1}$). **IR (neat, cm^{-1}):** ν = 2954, 1583, 1495, 1468, 1390, 1329, 1262, 1240, 1165, 1127, 1047, 816, 768, 715, 644. **HRMS (ESI+)** calculated for $[M+H]^+$: 457.2122 ($C_{28}H_{29}N_2O_4^+$), Found 457.2120.

6-acetyl-1,13-dimethoxy-5,9-dipropyl-5,9-dihydro-13bH-quinolino[2,3,4-kl]acridin-13b-ylium tetrafluoroborate (17a)

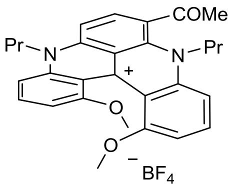

Compound **16** (20 mg, 0.04 mmol, 1 equiv) was dissolved in 3 mL of anhydrous CH_2Cl_2. To this solution was added $SOCl_2$ (3.2 µL, 0.05 mmol, 1.2 equiv) and the reaction was stirred for 10 min at 25 °C. Then the reaction mixture was cooled to -5 °C using an ice/salt bath and methylmagnesium iodide (3 M in Et_2O, 19 µL, 0.06 mmol, 1.5 equiv) was added and after 30 min of stirring at -5 °C, the mixture was hydrolysed by addition of 1 mL of HBF_4 1 M in H_2O. The reaction mixture was extracted with CH_2Cl_2, washed with $NaBF_4$ 0.2 M in H_2O. The organic phase was dried over Na_2SO_4, filtered and finally evaporated. Purification by silicagel column chromatography (CH_2Cl_2/MeOH, 97:3) afforded the pure product as a green solid (15 mg, 75%).

R_f = 0.46 (SiO_2, CH_2Cl_2/MeOH, 97:3). **M. P.:** 110-112 °C. **^{1}H NMR (CD_2Cl_2, 500 MHz)** : δ = 8.58 (d, *J* = 9.7 Hz, 1H, CH), 8.03 (t, *J* = 8.9 Hz, 1H, CH), 7.92 (t, *J* = 8.2 Hz, 1H, CH), 7.57 (d, *J* = 9.7 Hz, 1H, CH), 7.51 (d, *J* = 8.2 Hz, 1H, CH), 7.48 (d, *J* = 8.9 Hz, 1H, CH), 7.00 (d, *J* = 8.2 Hz, 1H, CH), 6.93 (d, *J* = 8.9 Hz, 1H, CH), 4.92 – 4.85 (m, 1H, CH_2), 4.78 – 4.70 (m, 1H, CH_2), 4.59 – 4.51 (m, 1H, CH_2), 3.80 (s, 3H, OCH_3), 3.77 (s, 3H, OCH_3), 3.69 – 3.58 (m, 1H, CH_2), 2.78 (s, 3H, CH_3), 2.27 – 2.14 (m, 2H, CH_2), 1.76 – 1.67 (m, 2H, CH_2), 1.28 (t, *J* = 7.4 Hz, 3H, CH_3), 0.40 (t, *J* = 7.4 Hz, 3H, CH_3). **$^{13}C\{^1H\}$ NMR (CD_2Cl_2, 125 MHz):** δ = 196.3 (C=O), 160.2 (C), 159.6 (C), 142.2 (C), 141.9 (C), 141.2 (C), 140.5 (C),139.0 (CH), 138.6 (CH),137.3 (CH), 129.6 (C), 120.6 (C), 120.6 (C), 116.7 (C), 114.1 (C), 110.6 (CH), 108.0 (CH), 105.9 (C), 104.8 (CH), 104.4 (CH), 60.1 (CH_2), 56.5 (OCH_3), 56.4 (OCH_3), 53.1 (CH_2), 28.8 (CH_3), 22.7 (CH_2), 21.0 (CH_2), 11.4 (CH_3), 10.8 (CH_3). **^{19}F NMR (CD_2Cl_2, 282 MHz):** δ = -150.7. **UV-Vis:** λ_{max} (CH_3CN) = 593 nm (ε = 9080 L.mol^{-1}.cm^{-1}). **IR (neat, cm^{-1}):** ν = 2928, 2854, 1674, 1580, 1504, 1467, 1405, 1390, 1345, 1268, 1236, 1171, 1123, 1041, 974, 895, 870, 818. **HRMS (ESI+)** calculated for [M+]: 455.2328 ($C_{29}H_{31}N_2O_3^+$), Found 455.2329.

6-benzoyl-1,13-dimethoxy-5,9-dipropyl-5,9-dihydro-13bH-quinolino[2,3,4-kl]acridin-13b-ylium tetrafluoroborate (17b)

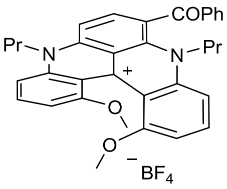

Compound **16** (20 mg, 0.04 mmol, 1 equiv) was dissolved in 3 mL of anhydrous CH_2Cl_2. To this solution was added $SOCl_2$ (3.2 µL, 0.05 mmol, 1.2 equiv) and the reaction was stirred for 10 min at 25 °C. Then the reaction mixture was cooled to -5 °C using an ice/salt bath and phenylmagnesium bromide (1.5 M in THF, 32 µL, 0.06 mmol, 1.5 equiv) was added and after 30 min of stirring at - 5 °C, the mixture was hydrolysed by addition of 1 mL of HBF_4 1 M in H_2O. The reaction mixture was extracted with CH_2Cl_2, washed with $NaBF_4$ 0.2 M in H_2O. The organic phase was dried over Na_2SO_4, filtered and finally evaporated. Purification by silicagel column chromatography (CH_2Cl_2/MeOH, 97:3) afforded the pure product as a green solid (15 mg, 67%).

R_f = 0.35 (SiO_2, CH_2Cl_2/MeOH, 97:3). **M. P.:** 130-132 °C. **^{1}H NMR (CD_2Cl_2, 500 MHz):** δ = 8.29 (d, *J* = 8.8 Hz, 1H, CH), 8.05 (t, *J* = 8.6 Hz, 1H, CH), 7.89 – 7.84 (m, 3H, CH), 7.71 (t, *J* = 6.8 Hz, 1H, CH), 7.57 – 7.51 (m, 4H, CH), 7.18 (d, *J* = 8.8 Hz, 1H, CH), 7.01 (d, *J* = 8.2 Hz, 1H, CH), 6.93 (d, *J* = 8.2 Hz, 1H, CH), 4.79-4.71 (m, 1H, CH_2), 4.58 – 4.49 (m, 1H, CH_2), 4.43 – 4.36 (m, 1H, CH_2), 3.81 (s, 3H, OCH_3), 3.78 (s, 3H, OCH_3), 3.78 – 3.72 (m, 1H, CH_2), 2.28 – 2.16 (m, 2H, CH_2), 1.77 – 1.69 (m, 1H, CH_2), 1.28 (t, *J* = 7.4 Hz, 3H, CH_3), 0.1 – 0.82 (m, 1H, CH_2), 0.39 (t, *J* = 7.4 Hz, 3H, CH_3). **$^{13}C\{^1H\}$ NMR (CD_2Cl_2, 125 MHz):** δ = 194.1 (C=O), 160.3 (C), 159.6 (C), 142.1 (C), 142.1 (C), 142.0 (C), 141.6 (C), 140.2 (C), 139.7 (CH), 138.8 (CH), 137.3 (CH), 137.0 (C), 134.5 (CH), 130.5 (2 CH), 129.6 (2 CH), 120.7 (C), 119.4 (C), 116.7 (C), 114.3 (C), 110.2 (CH), 108.0 (CH), 105.7 (CH),104.8 (CH), 104.5 (CH), 58.9 (NCH_2), 56.5 (OCH_3), 56.4 (OCH_3), 52.9 (NCH_2), 22.6 (CH_2), 20.9 (CH_2), 11.4 (CH_3), 10.8 (CH_3). **^{19}F NMR (CD_2Cl_2, 282 MHz):** δ = -150.6. **UV-Vis:** λ_{max} (CH_3CN) = 598 nm (ε = 9130 L.mol^{-1}.cm^{-1}). **IR (neat, cm^{-1}):** ν = 2960, 1650, 1587, 1508, 1469,

1347, 1273, 1172, 1128, 1055, 818. **HRMS (ESI+)** calculated for [M+]: 517.2486 ($C_{34}H_{33}N_2O_3^+$), Found 517.5201.

6-(ethoxycarbonyl)-1,13-dimethoxy-5,9-dipropyl-5,9-dihydro-13bH-quinolino[2,3,4-kl]acridin-13b-ylium tetrafluoroborate (17c)

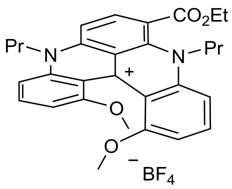

Compound **16** (100 mg, 0.18 mmol, 1 equiv) was dissolved in 2 mL of anhydrous CH_2Cl_2. To this solution was added $SOCl_2$ (40 µL, 0.56 mmol, 6 equiv) and the reaction was stirred for 10 min at 25 °C. Then freshly distilled ethanol (54 µL, 0.92 mmol, 5 equiv) was added and after 10 min of stirring at 25 °C the mixture was concentrated under reduced pressure. The residue was diluted in CH_2Cl_2 and washed with HBF_4 1 M in H_2O, dried over Na_2SO_4, filtered and finally evaporated. Purification by silicagel column chromatography (CH_2Cl_2/MeOH, 95:5) afforded the pure product as a shiny green solid (103 mg, 98%).

R_f = 0.65 (SiO_2, CH_2Cl_2/MeOH, 95:5). **M. P.:** 155-157 °C. **^{1}H NMR (CD_2Cl_2, 500 MHz):** δ = 8.62 (d, *J* = 9.1 Hz, 1H, CH), 8.02 (dd, *J* = 8.9, 8.8 Hz, 1H, CH), 7.90 (t, *J* = 8.3 Hz, 1H, CH), 7.51 (dd, *J* = 10.1, 9.1 Hz, 2H, CH), 7.44 (dd, *J* = 8.8, 0.9 Hz, 1H, CH), 6.99 (d, *J* = 8.1 Hz, 1H, CH), 6.91 (dd, J = 8.1, 0.9 Hz, 1H, CH), 4.92 – 4.87 (m, 1H, CH_2), 4.76 – 4.69 (m, 1H, CH_2), 4.52 – 4.47 (m, 3H, CH_2), 3.87 – 3.80 (m, 1H, CH_2), 3.80 (s, 3H, OCH_3), 3.76 (s, 3H, OCH_3), 2.22 – 2.16 (m, 2H, CH_2), 1.75 – 1.70 (m, 2H, CH_2), 1.46 (t, *J* = 7.0 Hz, 3H, CH_3), 1.26 (t, *J* = 7.4 Hz, 3H, CH_3), 0.40 (t, *J* = 7.4 Hz, 3H, CH_3). **$^{13}C\{^1H\}$ NMR (CD_2Cl_2, 125 MHz):** δ = 165.8 (C), 160.0 (C), 159.4 (C), 142.1 (C), 141.7 (C), 141.3 (C), 141.2 (C), 140.3 (C), 139.6 (CH), 128.5 (CH), 137.1 (CH), 120.5 (C), 116.4 (C), 112.8 (C), 110.2 (C), 107.8 (CH), 105.9 (CH), 104.6 (CH), 104.2 (CH), 62.4 (CH_2), 59.2 (CH_2), 56.23 (OCH_3), 56.2 (OCH_3), 52.9 (CH_2), 22.5 (CH_2), 20.8 (CH_2), 14.6 (CH_3), 11.2 (CH_3), 10.6 (CH_3). **^{19}F NMR (CD_2Cl_2, 282 MHz):** δ = -152.6. **UV-Vis:** λ_{max} (CH_3CN) = 592 nm (ε = 12200 L.mol^{-1}.cm^{-1}). **IR (neat, cm^{-1}):** ν = 3627, 3112, 2957, 1707, 1584, 1504, 1466, 1338, 1269, 1238, 1170, 1124, 1035, 816, 763, 718. **HRMS (ESI+)** calculated for [M+]: 485.2435 ($C_{30}H_{33}N_2O_4^+$), Found 485.2437.

1,13-dimethoxy-6-(phenoxycarbonyl)-5,9-dipropyl-5,9-dihydro-13bH-quinolino[2,3,4-kl]acridin-13b-ylium tetrafluoroborate (17d)

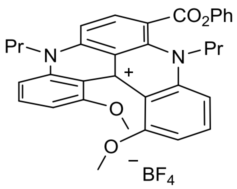

Compound **16** (50 mg, 0.09 mmol, 1 equiv) was dissolved in 1 mL of anhydrous CH_2Cl_2. To this solution was added $SOCl_2$ (20 µL, 0.28 mmol, 3 equiv) and the reaction was stirred for 10 min at 25 °C. Then phenol (129 mg, 1.38 mmol, 15 equiv) was added and after 2 h of stirring at 25 °C the mixture was quenched with water. The organic layer was extracted and washed with a 0.2 M aqueous diluted solution

of $NaBF_4$, dried over Na_2SO_4, filtered and finally evaporated. Purification by silicagel column chromatography (CH_2Cl_2/MeOH, 95:5) afforded the pure product as a green solid (50 mg, 88%).

R_f = 0.4 (SiO_2, CH_2Cl_2/MeOH, 95:5). **M. P.:** 128-140 °C. **^{1}H NMR (CD_2Cl_2, 500 MHz):** δ = 8.88 (d, *J* = 9.1 Hz, 1H, CH), 8.07 (t, *J* = 8.9 Hz, 1H, CH), 7.91 (t, *J* = 8.5 Hz, 1H, CH), 7.62 (d, *J* = 9.0 Hz, 1H, CH), 7.56 (d, *J* = 9.0 Hz, 1H, CH), 7.53 – 7.49 (m, 2H, CH), 7.44 (dd, *J* = 8.7, 0.8 Hz, 1H, CH), 7.39 – 7.34 (m, 1H, CH), 7.34 – 7.29 (m, 2H, CH), 7.04 (d, *J* = 8.1 Hz, 1H, CH), 6.94 (dd, *J* = 8.4, 0.8 Hz, 1H, CH), 5.02 – 4.97 (m, 1H, CH_2), 4.84 – 4.74 (m, 1H, CH_2), 4.63 – 4.56 (m, 1H, CH_2), 4.01 – 3.95 (m, 1H, CH_2), 3.83 (s, 3H, OCH_3), 3.78 (s, 3H, OCH_3), 2.32 – 2.18 (m, 2H, CH_2), 1.81 – 1.77 (m, 2H, CH_2), 1.30 (t, *J* = 7.4 Hz, 3H, CH_3), 0.46 (t, *J* = 7.4 Hz, 3H, CH_3). **^{13}C{^{1}H} NMR (CD_2Cl_2, 125 MHz):** δ = 164.1 (C), 160.1 (C), 159.4 (C), 151.1 (C), 142.1 (C), 142.0 (C), 141.7 (C), 141.2 (C), 140.9 (C), 140.0 (CH), 138.7 (CH), 137.3 (CH), 130.1 (CH), 126.8 (CH), 122.0 (CH), 120.5 (C), 116.5 (C), 114.1 (C), 111.1 (C), 110.4 (CH), 107.9 (CH), 106.3 (CH), 104.9 (CH), 104.4 (CH), 59.7 (CH_2), 56.4 (OCH_3), 56.2 (OCH_3), 53.0 (CH_2), 22.6 (CH_2), 20.9 (CH_2), 11.3 (CH_3), 10.7 (CH_3). **^{19}F NMR (CD_2Cl_2, 282 MHz):** δ = -152.5. **UV-Vis:** λ_{max} (CH_3CN) = 586 nm (ε = 11700 $L.mol^{-1}.cm^{-1}$). **IR (neat, cm^{-1}):** ν = 3631, 3550, 1724, 1583, 1504, 1469, 1410, 1390, 1336, 1269, 1235, 1188, 1165, 1119, 1036, 816, 786, 755, 691. **HRMS (ESI+)** calculated for [M+]: 533.2435 ($C_{34}H_{33}N_2O_4^+$), Found 533.2436.

6-((ethylthio)carbonyl)-1,13-dimethoxy-5,9-dipropyl-5,9-dihydro-13bH-quinolino[2,3,4-kl]acridin-13b-ylium tetrafluoroborate(17e)

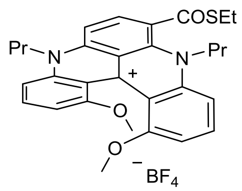

Compound **16** (100 mg, 0.18 mmol, 1 equiv) was dissolved in 1 mL of anhydrous CH_2Cl_2. To this solution was added $SOCl_2$ (20 µL, 0.28 mmol, 3 equiv) and the reaction was stirred for 10 min at 25 °C. Then ethanethiol (102 µL, 1.38 mmol, 15 equiv) was added and after 10 min of stirring at 25 °C the mixture was concentrated under reduced pressure. The residue was diluted in CH_2Cl_2 and washed with HBF_4 1 M in H_2O, dried over Na_2SO_4, filtered and finally evaporated. Purification by silicagel column chromatography (CH_2Cl_2/MeOH, 95:5) afforded the pure product as a green solid (41 mg, 76%).

R_f = 0.39 (SiO_2, CH_2Cl_2/MeOH, 95:5). **M. P.:** 158-160 °C. **^{1}H NMR (CD_2Cl_2, 500 MHz):** δ = 8.56 (d, *J* = 9.1 Hz, 1H, CH), 8.02 (t, *J* = 8.9 Hz, 1H, CH), 7.90 (t, *J* = 8.5 Hz, 1H, CH), 7.50 (dd, *J* = 9.2, 8.8 Hz, 2H, CH), 7.40 (d, *J* = 8.6 Hz, 1H, CH), 6.99 (d, *J* = 8.0 Hz, 1H, CH), 6.91 (d, J = 8.0 Hz, 1H, CH), 4.81 – 4.69 (m, 2H, CH_2), 4.54 – 4.51 (m, 1H, CH_2), 3.83 – 3.78 (m, 1H, CH_2), 3.79 (s, 3H, OCH_3), 3.75 (s, 3H, OCH_3), 3.22 – 3.15 (m, 2H, CH_2), 2.22 – 2.15 (m, 2H, CH_2), 1.76 – 1.71 (m, 2H, CH_2), 1.43 (t, *J* = 7.4 Hz, 3H, CH_3), 1.26 (t, *J* = 7.3 Hz, 3H, CH_3), 0.44 (t, *J* = 7.3 Hz, 3H, CH_3). **^{13}C{^{1}H} NMR (CD_2Cl_2, 125 MHz):** δ = 191.7 (C), 160.0 (C), 159.4 (C), 142.3 (C), 141.8 (C), 141.5 (C), 140.2 (C), 139.3 (C), 138.6 (CH), 138.0 (CH), 137.2 (CH), 120.5 (C), 120.2 (C), 116.5 (C), 114.0 (C), 110.1 (CH), 107.8 (CH), 105.6 (CH), 104.6 (CH), 104.3 (CH), 58.6 (CH_2), 56.3 (CH_3), 56.2 (CH_3), 52.8 (CH_2), 24.8 (CH_2), 22.6 (CH_2), 20.8 (CH_2), 15.0 (CH_3), 11.2

(CH_3), 10.6 (CH_3). **^{19}F NMR (CD_2Cl_2, 282 MHz):** δ = -152.6. **UV-Vis:** λ_{max} (CH_3CN) = 596 nm (ε = 11100 L.mol^{-1}.cm^{-1}). **IR (neat, cm^{-1}):** ν = 2954, 2876, 1650, 1582, 1504, 1468, 1389, 1342, 1265, 1219, 1171, 1039, 975, 880, 817, 782, 752, 721, 698, 670, 622. **HRMS (ESI+)** calculated for [M+]: 501.2207 ($C_{30}H_{33}N_2O_3S^+$), Found 501.2217.

1,13-dimethoxy-5,9-dipropyl-6-(propylcarbamoyl)-5,9-dihydro-13bH-quinolino[2,3,4-kl]acridin-13b-ylium tetrafluoroborate (17f)

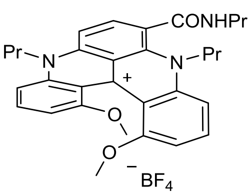

Compound **16** (50 mg, 0.09 mmol, 1 equiv) was dissolved in 2 mL of anhydrous CH_2Cl_2. To this solution was added $SOCl_2$ (20 µL, 0.28 mmol, 3 equiv) and the reaction was stirred for 10 min at 25 °C. Then propylamine (113 µL, 1.38 mmol, 15 equiv) was added at 0 °C and after 15 min of stirring at 25 °C the mixture was quenched with water. The organic layer was extracted and washed with HBF_4 1 M in H_2O, dried over Na_2SO_4, filtered and finally evaporated. Purification by silica column chromatography (CH_2Cl_2/MeOH, 95:5) afforded the pure product as a green solid (51 mg, 94%).

R_f = 0.57 (SiO_2, CH_2Cl_2/MeOH, 95:5). **M. P.:** 138-140 °C. **^{1}H NMR (CD_2Cl_2, 500 MHz):** δ = 8.26 (d, *J* = 8.8 Hz, 1H, CH), 7.94 (t, *J* = 8.9 Hz, 1H, CH), 7.84 (t, *J* = 8.6 Hz, 1H, CH), 7.44 (d, *J* = 8.9 Hz, 1H, CH), 7.41 (d, *J* = 8.9 Hz, 1H, CH), 7.37 (dd, *J* = 8.9, 0.8 Hz, 1H, CH), 7.19 (t, *J* = 5.7 Hz, 1H, NH), 6.90 (d, *J* = 8.0 Hz, 1H, CH), 6.84 (dd, *J* = 8.2, 0.8 Hz, 1H, CH), 4.77 – 4.72 (m, 1H, CH_2), 4.65 – 4.60 (m, 1H, CH_2), 4.42 – 4.36 (m, 1H, CH_2), 4.13 – 4.07 (m, 1H, CH_2), 3.76 (s, 3H, OCH_3), 3.74 (s, 3H, OCH_3), 2.22 – 2.09 (m, 2H, CH_2), 1.78 – 1.66 (m, 2H, CH_2), 1.22 (t, *J* = 7.4 Hz, 3H, CH_3), 1.03 (t, *J* = 7.3 Hz, 3H, CH_3), 0.50 (t, *J* = 7.3 Hz, 3H, CH_3). **^{13}C{^{1}H} NMR (CD_2Cl_2, 125 MHz):** δ = 167.6 (C), 160,0 (C), 159.3 (C), 142.7 (C), 139.2 (C), 138.9 (C), 137.9 (CH), 137.5 (CH), 136.7 (CH), 120.9 (C), 118.6 (C), 116.4 (C), 113.9 (C), 110.1 (CH), 107.7 (CH), 105.4 (CH), 103.9 (CH), 103.7 (CH), 56.6 (CH_2), 56.2 (OCH_3), 56.1 (OCH_3), 52.6 (CH_2), 42.4 (CH_2), 23.1 (CH_2), 22.5 (CH_2), 20.5 (CH_2), 11.7 (CH_3), 11.3 (CH_3), 10.7 (CH_3). **^{19}F NMR (CD_2Cl_2, 282 MHz):** δ = -152.0. **UV-Vis:** λ_{max} (CH_3CN) = 609 nm (ε = 11500 L.mol^{-1}.cm^{-1}). **IR (neat, cm^{-1}):** ν = 3367, 2943, 1643, 1587, 1500, 1468, 1388, 1284, 1261, 1243, 1170, 1127, 1170, 1127, 1036, 815, 756, 646. **HRMS (ESI+)** calculated for [M+]: 498.2751 ($C_{31}H_{36}N_3O_3^+$), Found 498.2752.

6-(diethylcarbamoyl)-1,13-dimethoxy-5,9-dipropyl-5,9-dihydro-13bH-quinolino[2,3,4-kl]acridin-13b-ylium tetrafluoroborate (17g)

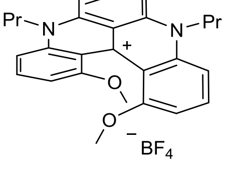

Compound **16** (50 mg, 0.09 mmol, 1 equiv) was dissolved in 2 mL of anhydrous CH_2Cl_2. To this solution was added $SOCl_2$ (20 µL, 0.28 mmol, 3 equiv) and the reaction was stirred for 10 min at 25 °C. Then diethylamine (143 µL, 1.38 mmol, 15 equiv) was added at 0 °C and after 15 min of stirring at 25 °C the mixture was

quenched with water. The organic layer was extracted and washed with HBF_4 1 M in H_2O, dried over Na_2SO_4, filtered and finally evaporated. Purification by silica column chromatography (CH_2Cl_2/MeOH, 95:5) afforded the pure product as a green solid (54 mg, 98%).

R_f = 0.35 (SiO_2, CH_2Cl_2/MeOH, 95:5). **M. P.:** 134-136 °C. **^{1}H NMR (DMSO-d_6, 368 K, 400 MHz):** δ = 8.10 – 8.00 (m, 2H, CH), 7.92 (dd, *J* = 8.0, 1.8 Hz, 1H, CH), 7.82 (d, *J* = 8.9 Hz, 1H, CH), 7.72 (d, *J* = 8.9 Hz, 1H, CH), 7.63 (d, *J* = 8.6 Hz, 1H, CH), 7.11 (d, *J* = 8.9 Hz, 1H, CH), 7.03 (d, *J* = 8.2 Hz, 1H, CH), 4.88 – 4.73 (m, 2H, CH_2), 4.72 – 4.60 (m, 1H, CH_2), 4.18 – 4.15 (m, 1H, CH_2), 3.77 (s, 3H, OCH_3), 3.73 (s, 3H, OCH_3), 3.62 (m, 2H, CH_2), 3.70 – 3.58 (m, 2H, CH_2), 3.40 – 3.14 (m, 2H, CH_2), 2.14 – 1.99 (m, 2H, CH_2), 1.77 – 1.57 (m, 2H, CH_2), 1.37 – 1.24 (m, 6H, CH_3), 1.17 (t, *J* = 7.3 Hz, CH_3), 0.57 (t, *J* = 7.3 Hz, 3H, CH_3). **^{13}C{^{1}H} NMR (DMSO-d_6, 343 K, 125 MHz):** δ = 167.7 (C), 158.8 (C), 158.2 (C), 141.8 (C), 141.2 (C), 141.0 (C), 137.6 (C), 137.4 (CH), 136.4 (CH), 135.7 (C), 119.9 (C), 114.7 (C), 112.7 (C), 109.2 (CH), 107.8 (CH), 107.7 (CH), 105.8 (C), 103.7 (CH), 103.4 (CH), 103.3 (CH), 55.8 (OCH_3), 55.5 (OCH_3), 54.5 (CH_2), 53.6 (CH_2), 50.8 (CH_2), 50.6 (CH_2), 21.3 (CH_2), 19.6 (CH_2), 11.9 (CH_3), 10.4 (2 CH_3), 10.1 (CH_3). **^{19}F NMR (CD_2Cl_2, 282 MHz):** δ = -152.6. **UV-Vis:** λ_{max} (CH_3CN) = 615 nm (ε = 10300 L.mol^{-1}.cm^{-1}). **IR (neat, cm^{-1}):** ν = 3619, 2943, 1587, 1501, 1464, 1430, 1385, 1346, 1319, 1281, 1262, 1170, 1122, 1037, 860, 815, 783, 757, 730, 643. **HRMS (ESI+)** calculated for [M+]: 512.2908 ($C_{32}H_{38}N_3O_3^+$), Found 512.2902.

3. ^1H, ^{13}C, ^{19}F NMR and HRMS analysis

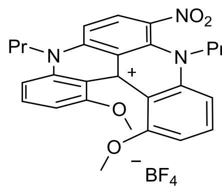

5

^1H NMR (400 MHz, CD_2Cl_2)

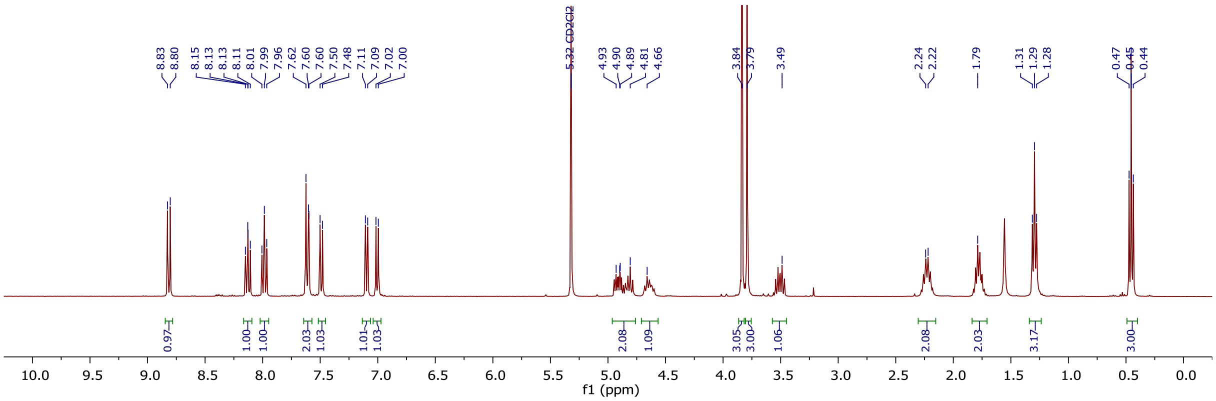

^{13}C NMR (101 MHz, CD_2Cl_2)

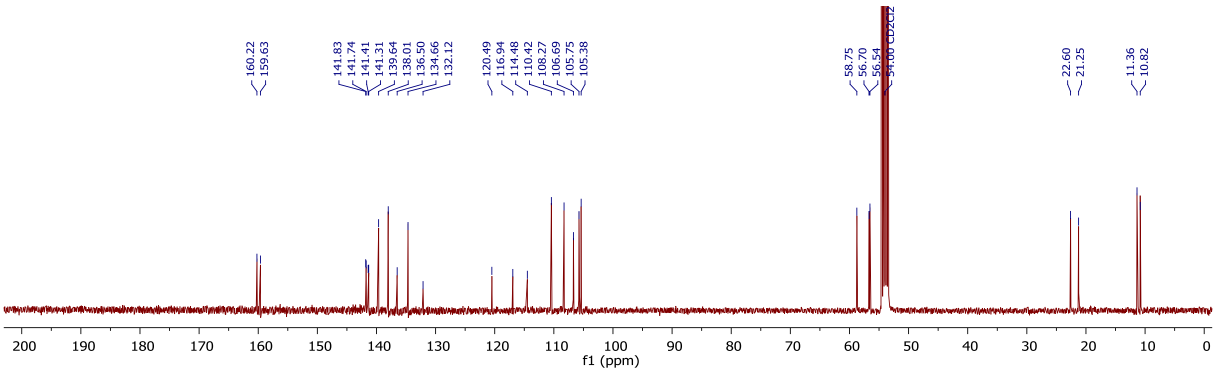

^{19}F NMR (282 MHz, CD_2Cl_2)

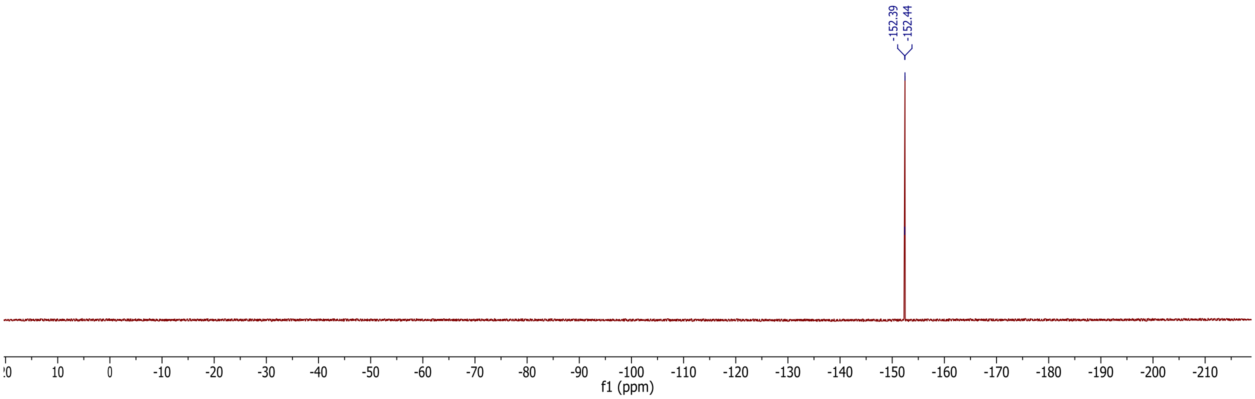

UNIVERSITY OF GENEVA

Faculty of Sciences

Sciences Mass Spectrometry

Submitter: HERNANDEZ-DELGADO	Date of reception: 03/12/15
Sample name: IH294	Date of certificate: 10/12/15
Sample number: 8163	Data filename: MS03GE-151209-ES-A001
Operator: Eliane Sandmeier	Instrument: QSTAR XL (AB/MDS Sciex)
Principal investigator: Dr. Sophie Michalet	Ionisation mode: ESI (positive mode)

Expected Formula	Observed *m/z* $[M]^+$	Expected *m/z* (amu)	Accuracy (ppm)
$C_{27}H_{28}N_3O_4$	458.2097	458.2074	4.9

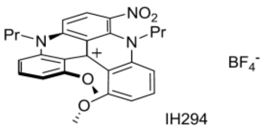

IH294

Chemical Formula: $C_{27}H_{28}N_3O_4^+$

Exact Mass: 458.21

Molecular Weight: 545.34

m/z: 458.21 (100.0%),

459.21 (30.8%), 460.21 (5.3%)

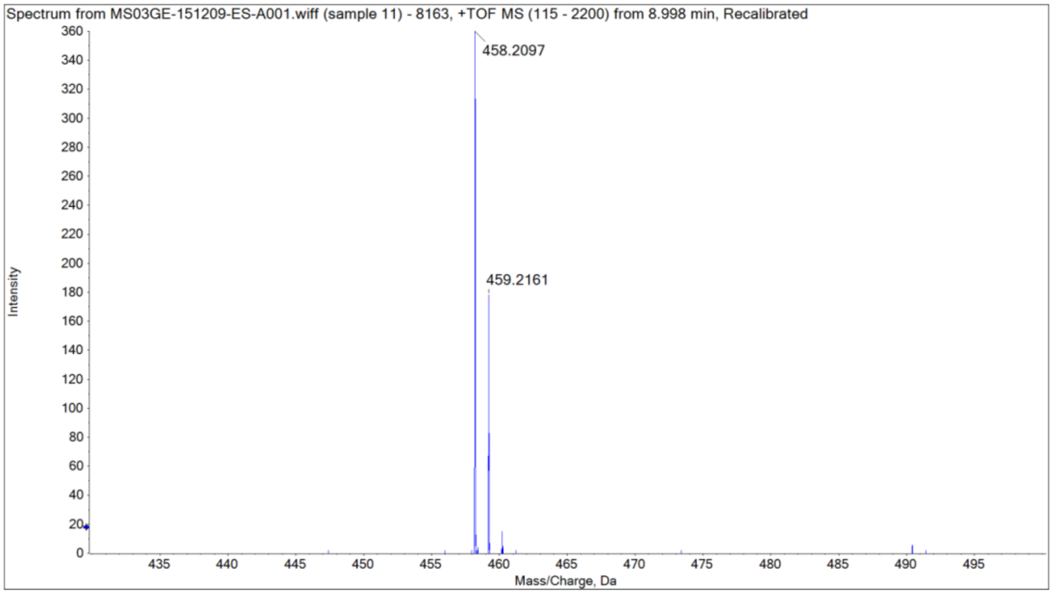

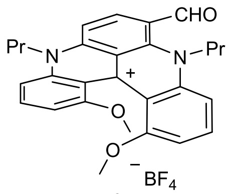

6

^{1}H NMR (400 MHz, CD_2Cl_2)

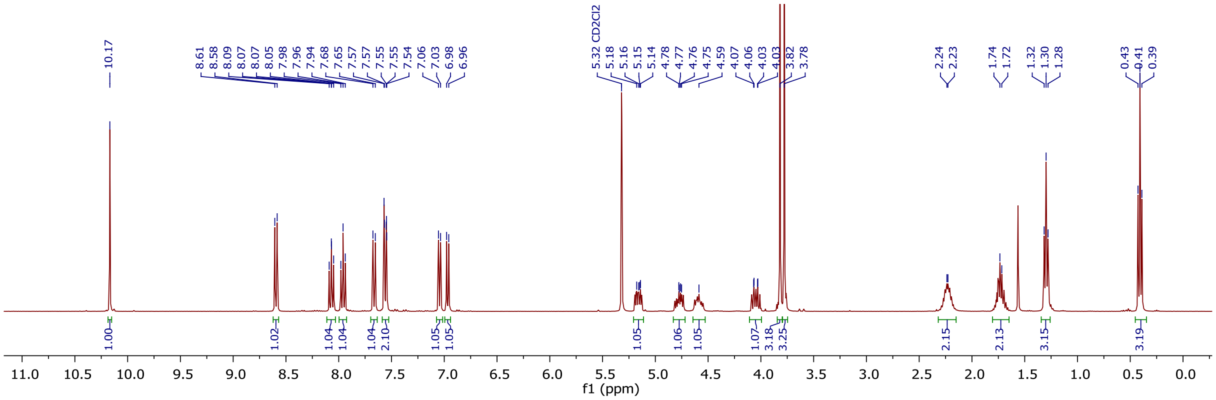

^{13}C NMR (101 MHz, CD_2Cl_2)

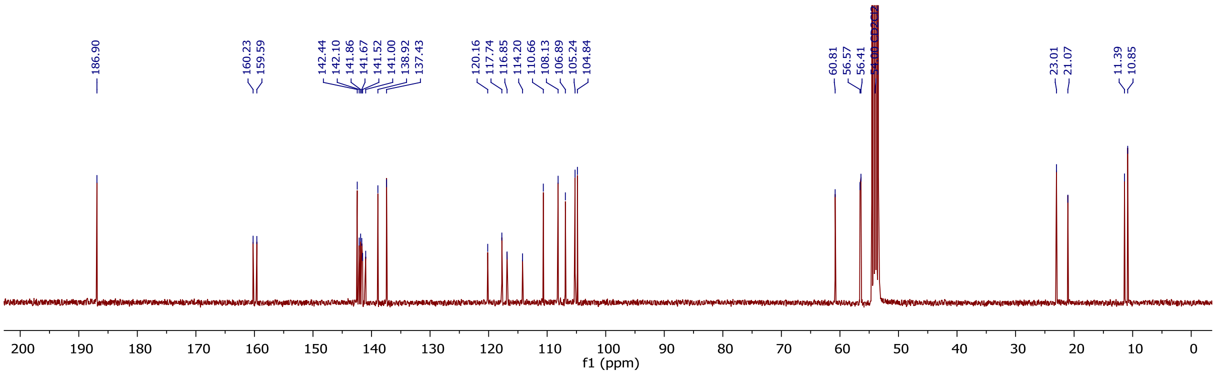

^{19}F NMR (282 MHz, CD_2Cl_2)

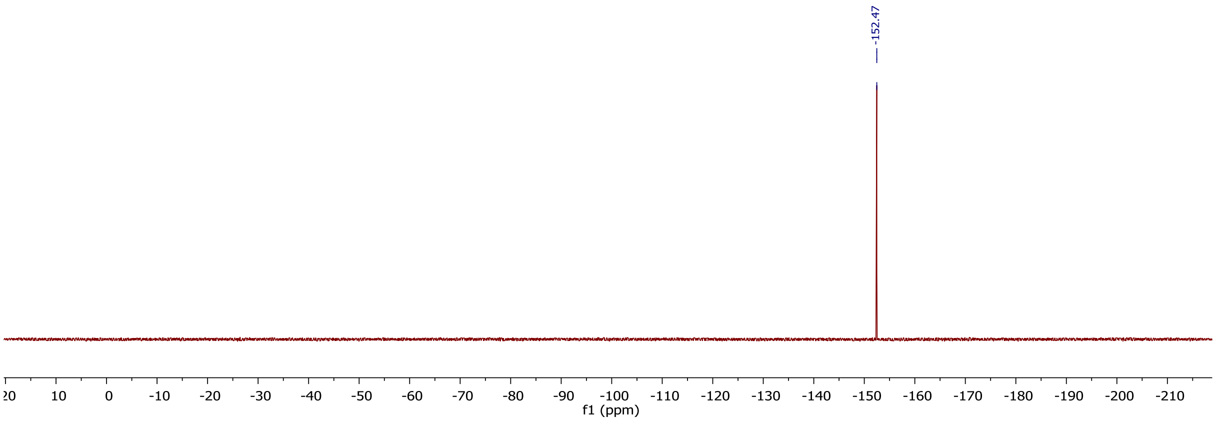

UNIVERSITY OF GENEVA

Faculty of Sciences

Sciences Mass Spectrometry

Submitter: HERNANDEZ-DELGADO	Date of reception: 03/12/15
Sample name: IH453	Date of certificate: 10/12/15
Sample number: 8165	Data filename: MS03GE-151209-ES-A001
Operator: Eliane Sandmeier	Instrument: QSTAR XL (AB/MDS Sciex)
Principal investigator: Dr. Sophie Michalet	Ionisation mode: ESI (positive mode)

Expected Formula	Observed *m/z* $[M]^+$	Expected *m/z* (amu)	Accuracy (ppm)
$C_{28}H_{29}N_2O_3$	441.2192	441.2173	4.3

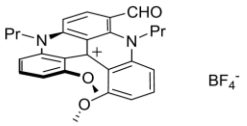

Chemical Formula: $C_{28}H_{29}N_2O_3^+$

IH453 Exact Mass: 441.22

Molecular Weight: 528.35

m/z: 441.22 (100.0%), 442.22 (30.7%), 443.22 (5.3%)

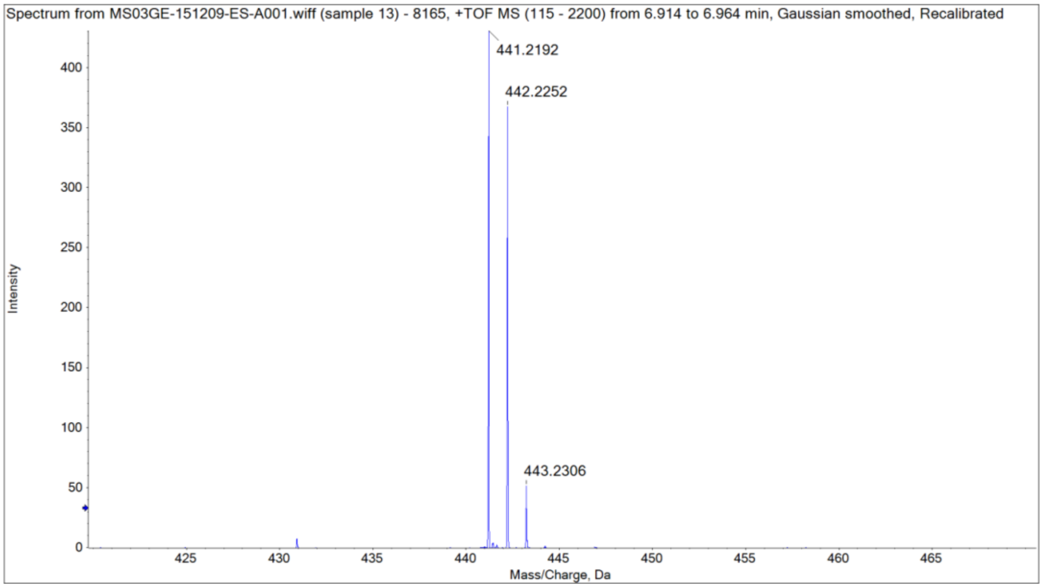

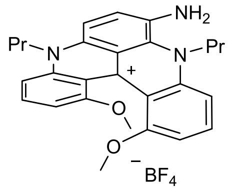

7

^{1}H NMR (400 MHz, CD_2Cl_2)

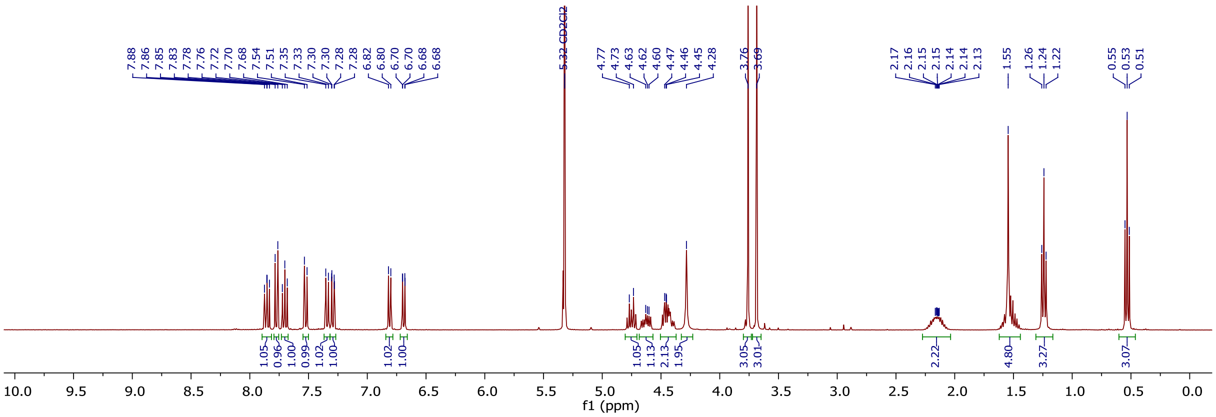

^{13}C NMR (101 MHz, CD_2Cl_2)

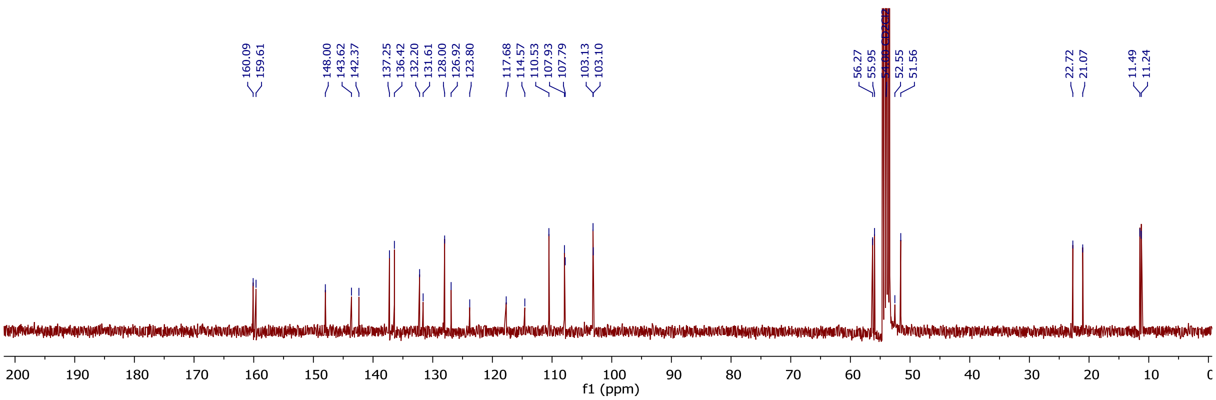

^{19}F NMR (282 MHz, CD_2Cl_2)

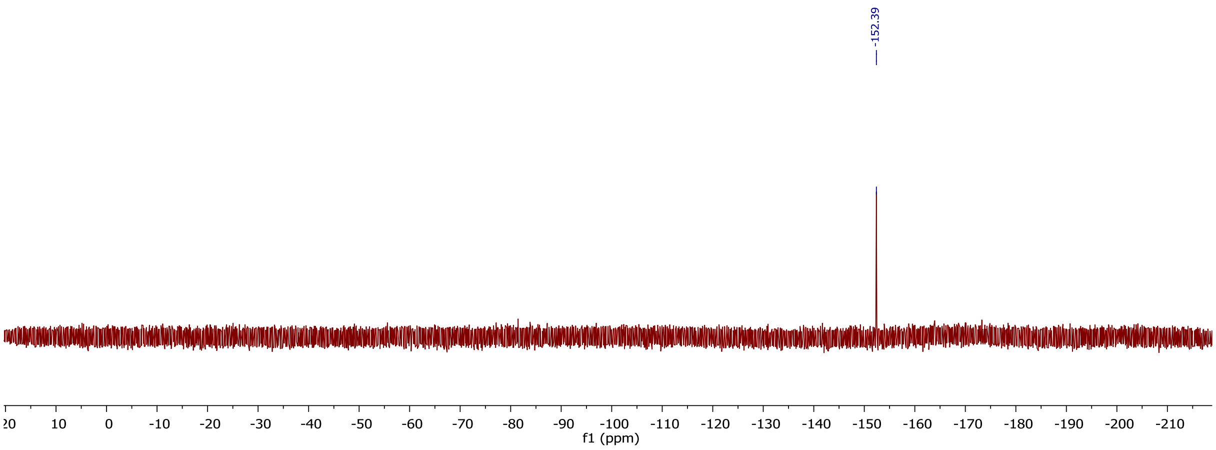

UNIVERSITY OF GENEVA

Faculty of Sciences

Sciences Mass Spectrometry

Submitter: HERNANDEZ DELGADO	Date of reception: 03/12/15
Sample name: IH212	Date of certificate: 09/12/15
Sample number: 8160	Data filename: MS03GE-151208-HT-A001
Operator: Harry Théraulaz	Instrument: QSTAR XL (AB/MDS Sciex)
Principal investigator: Dr. Sophie Michalet	Ionisation mode: ESI (positive mode)

Expected Formula	Observed *m/z* $[M]^+$	Expected *m/z* (amu)	Accuracy (ppm)
$C_{27}H_{30}N_3O_2$	428.2325	428.2333	-1.8

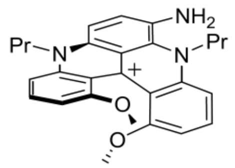

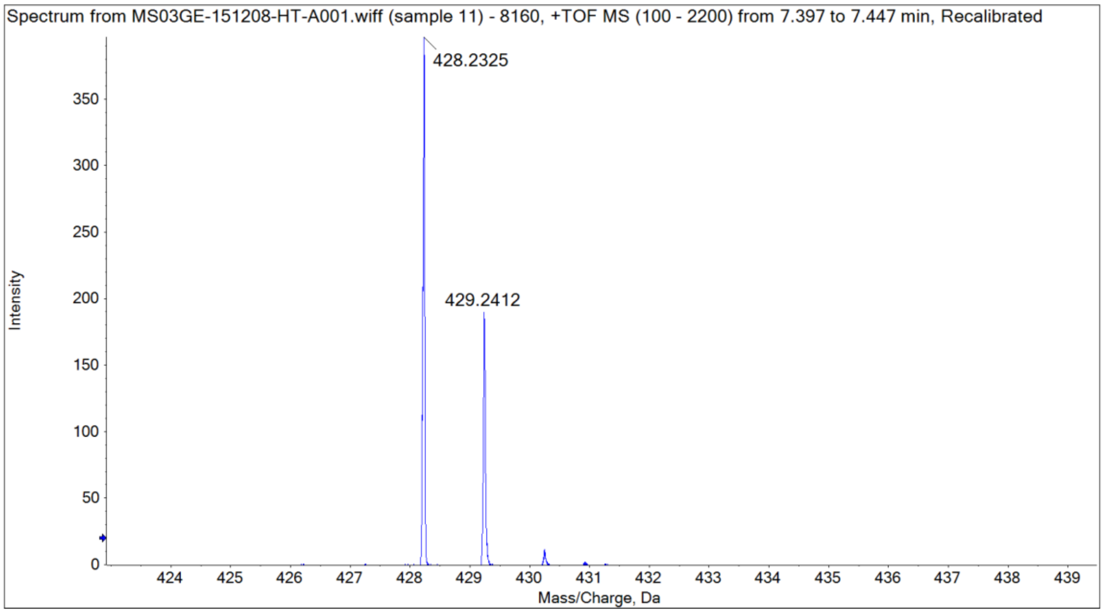

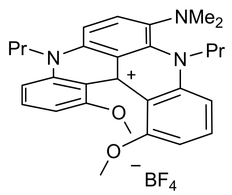

8

^{1}H NMR (400 MHz, CD_2Cl_2)

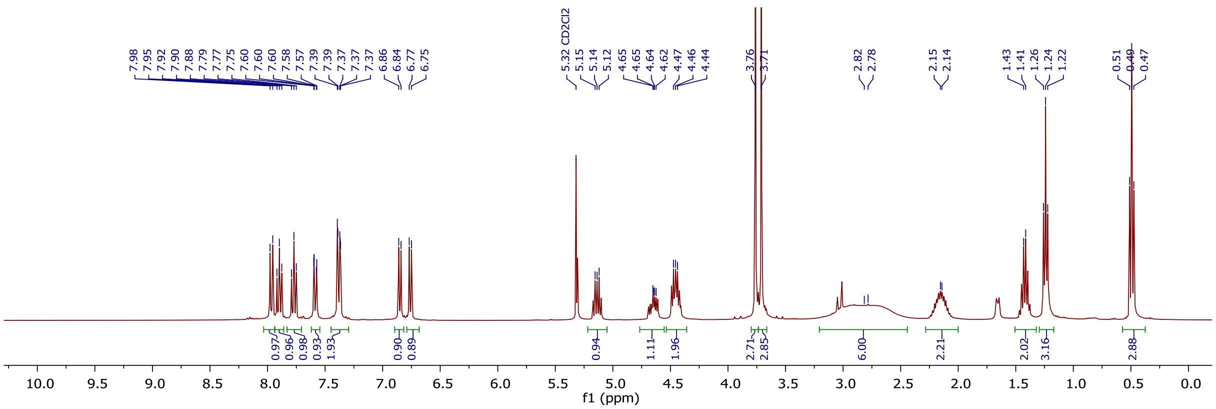

^{13}C NMR (101 MHz, CD_2Cl_2)

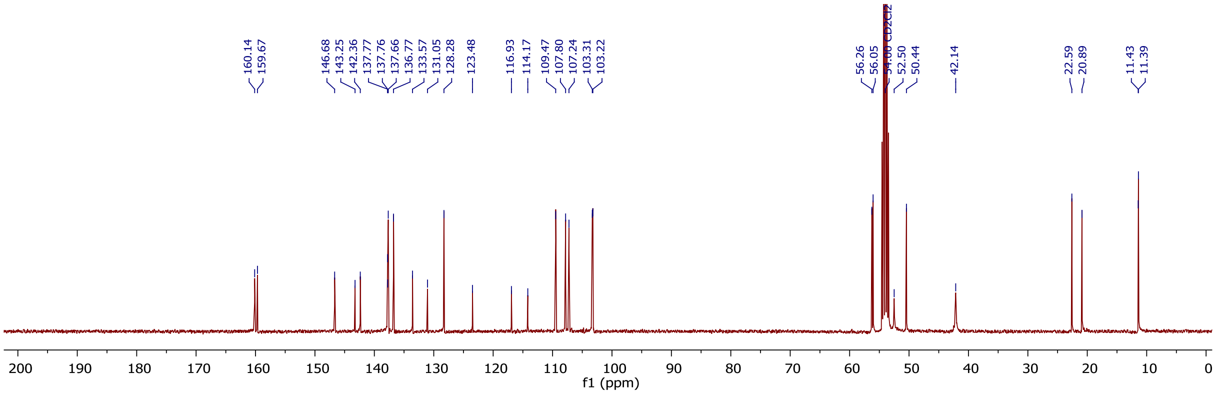

^{19}F NMR (282 MHz, CD_2Cl_2)

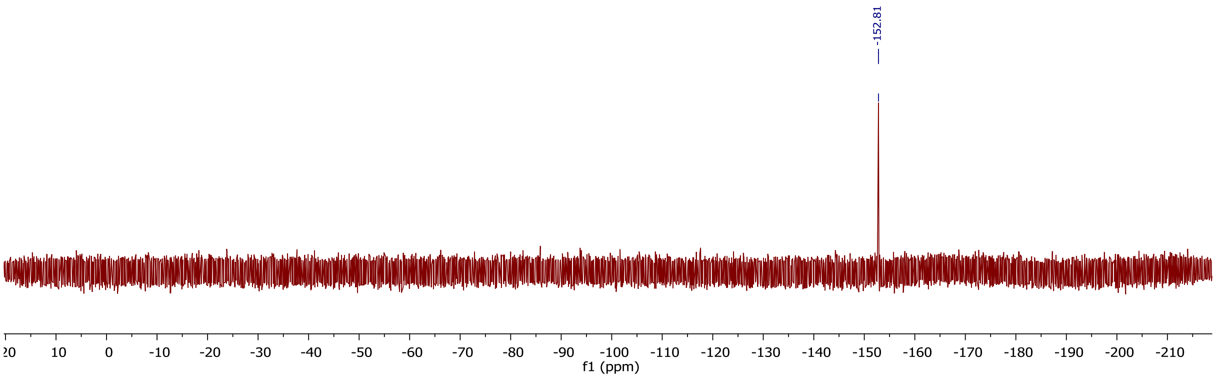

UNIVERSITY OF GENEVA

Faculty of Sciences

Sciences Mass Spectrometry

UNIVERSITÉ DE GENÈVE

SMS MS

Submitter:	HERNANDEZ DELGADO	Date of reception:	03/12/15
Sample name:	IH192	Date of certificate:	09/12/15
Sample number:	8155	Data filename:	MS03GE-151208-HT-A001
Operator:	Harry Théraulaz	Instrument:	QSTAR XL (AB/MDS Sciex)
Principal investigator:	Dr. Sophie Michalet	Ionisation mode:	ESI (positive mode)

Expected Formula	Observed *m/z* $[M]^+$	Expected *m/z* (amu)	Accuracy (ppm)
$C_{29}H_{34}N_3O_2$	456.2645	456.2645	-0.2

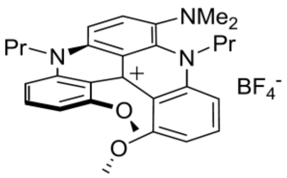

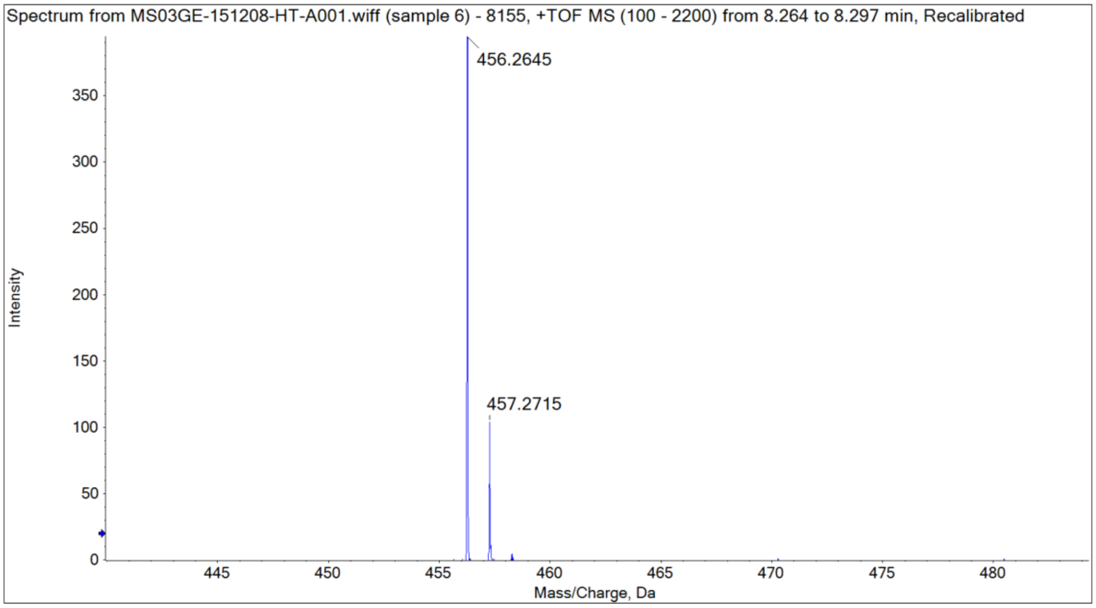

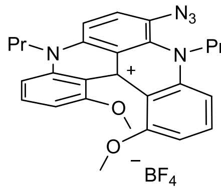

9

^{1}H NMR (400 MHz, CD_2Cl_2)

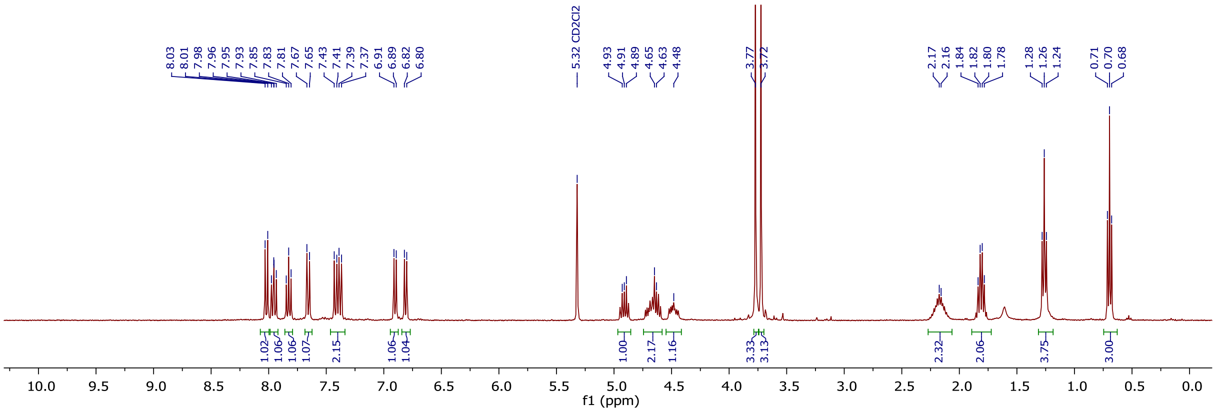

^{13}C NMR (101 MHz, CD_2Cl_2)

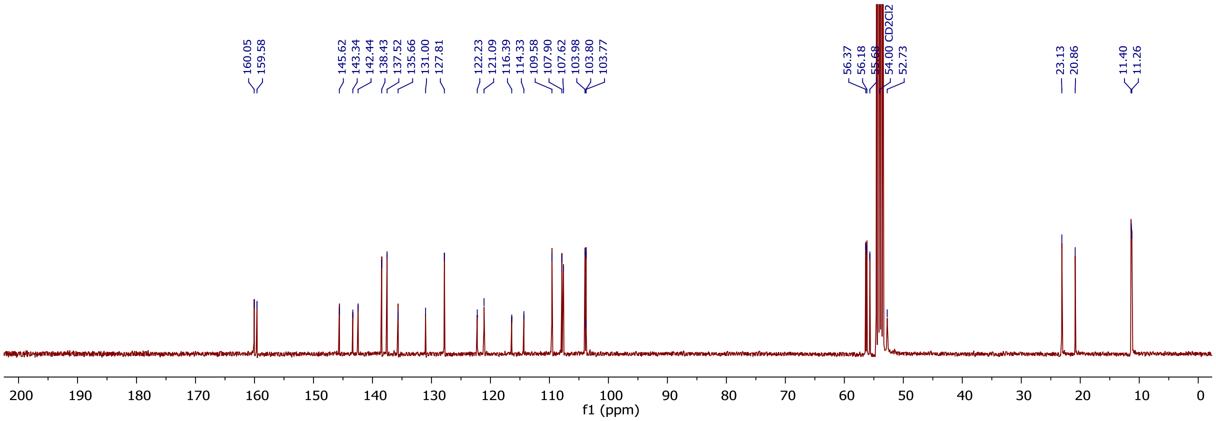

^{19}F NMR (282 MHz, CD_2Cl_2)

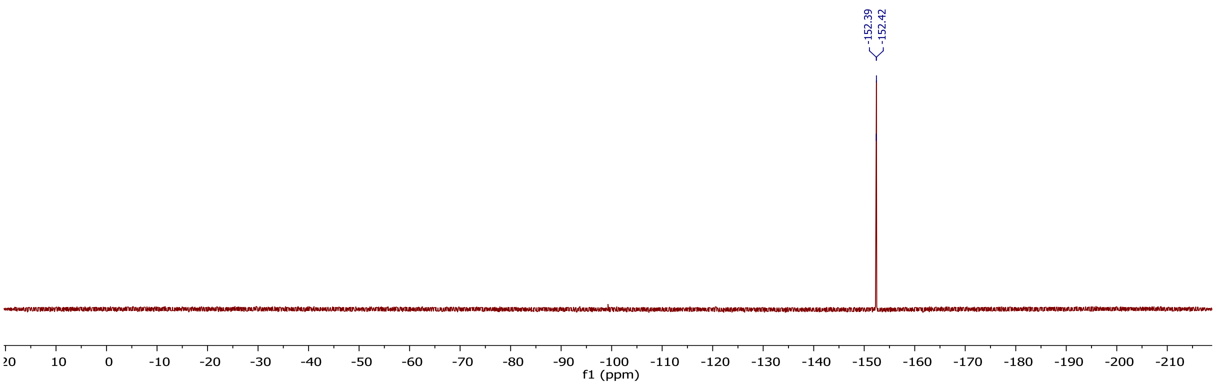

UNIVERSITY OF GENEVA

UNIVERSITÉ DE GENÈVE

Faculty of Sciences

Sciences Mass Spectrometry

Submitter: HERNANDEZ-DELGADO	Date of reception: 03/12/15
Sample name: IH278	Date of certificate: 10/12/15
Sample number: 8169	Data filename: MS03GE-151209-ES-A001
Operator: Eliane Sandmeier	Instrument: QSTAR XL (AB/MDS Sciex)
Principal investigator: Dr. Sophie Michalet	Ionisation mode: ESI (positive mode)

Expected Formula	Observed *m/z* $[M-N_2]^+$	Expected *m/z* (amu)	Accuracy (ppm)
$C_{27}H_{28}N_3O_2$	426.2188	426.2176	2.9

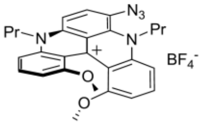

IH278

Chemical Formula: $C_{27}H_{28}N_5O_2^+$
Exact Mass: 454.22
Molecular Weight: 541.36
m/z: 454.22 (100.0%), 455.23 (29.6%), 456.23 (4.6%), 455.22 (1.8%)

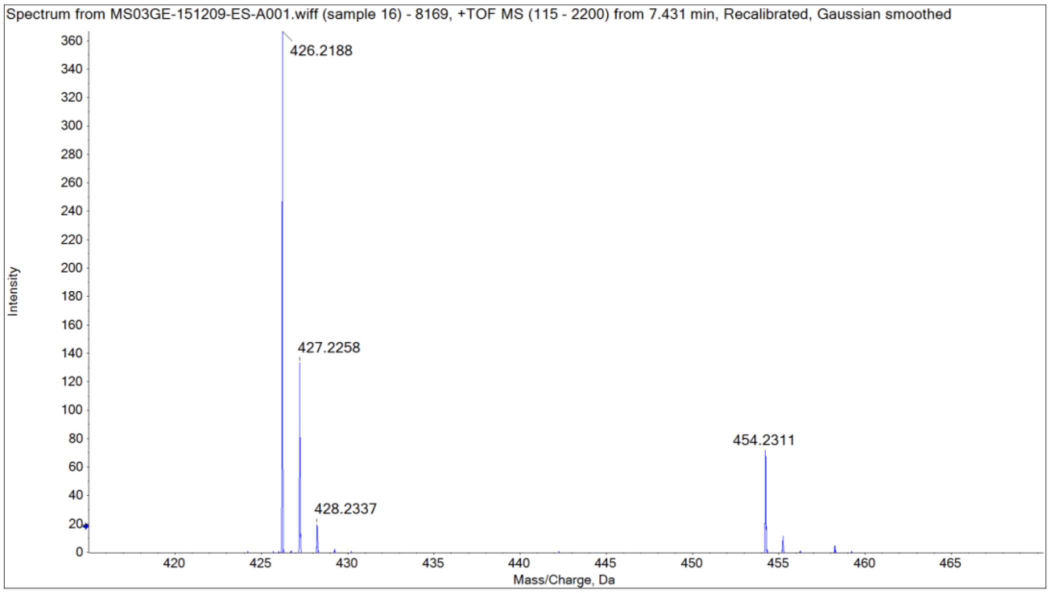

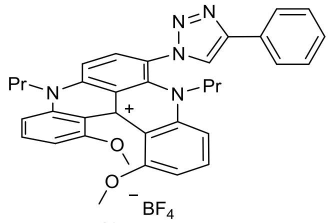

10a

^{1}H NMR (400 MHz, CD_2Cl_2)

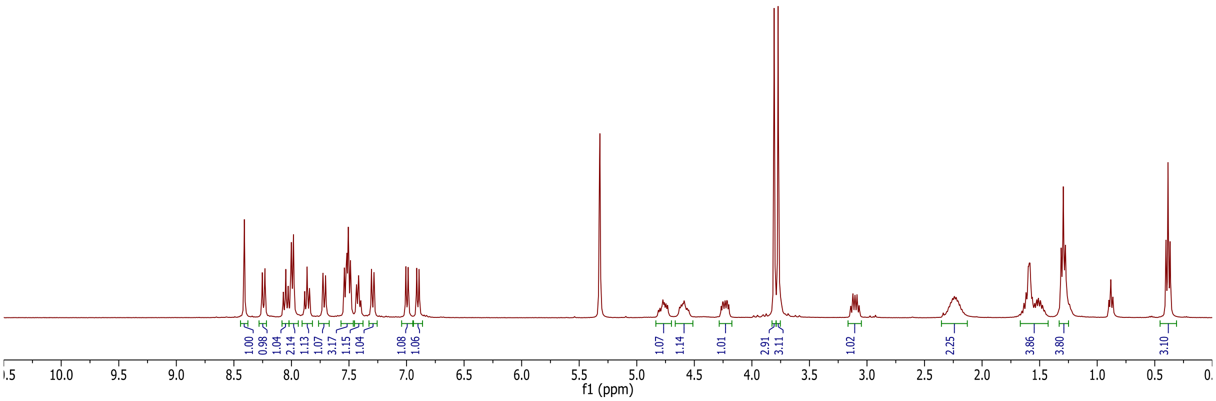

^{13}C NMR (101 MHz, CD_2Cl_2)

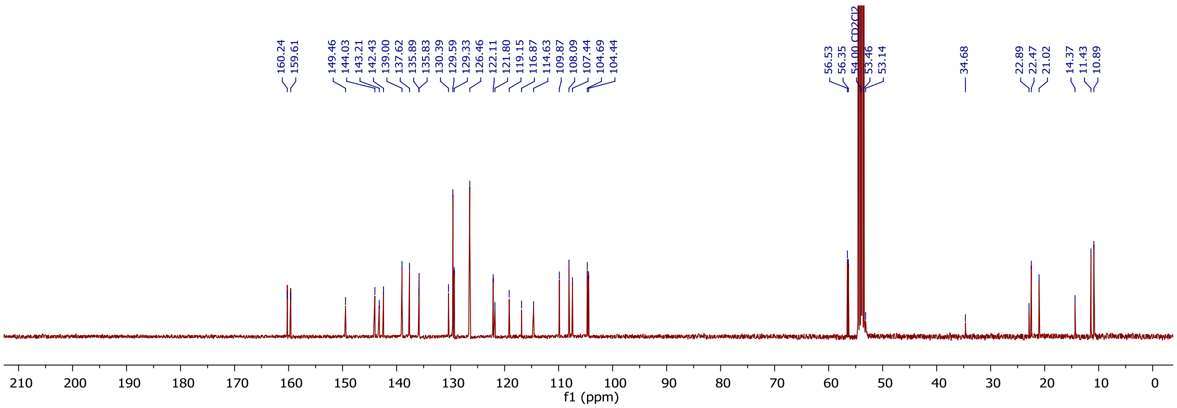

^{19}F NMR (282 MHz, CD_2Cl_2)

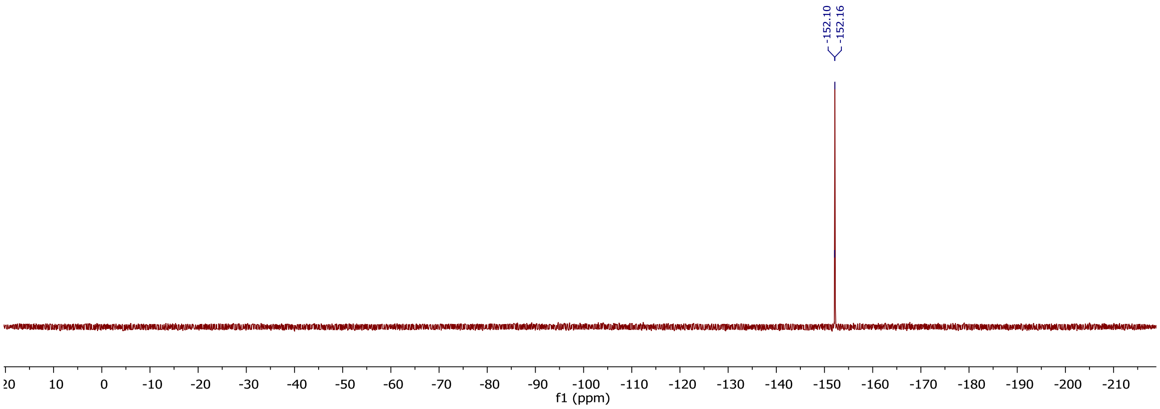

UNIVERSITY OF GENEVA

Faculty of Sciences

Sciences Mass Spectrometry

Submitter:	HERNANDEZ DELGADO	Date of reception:	09/12/15
Sample name:	IH275	Date of certificate:	17/12/15
Sample number:	8193	Data filename:	MS03GE-151216-HT-A001
Operator:	Harry Théraulaz	Instrument:	QSTAR XL (AB/MDS Sciex)
Principal investigator:	Dr. Sophie Michalet	Ionisation mode:	ESI (positive mode)

Expected Formula	Observed *m/z* $[M]^+$	Expected *m/z* (amu)	Accuracy (ppm)
$C_{35}H_{34}N_5O_2$	556.2681	556.2707	-4.6

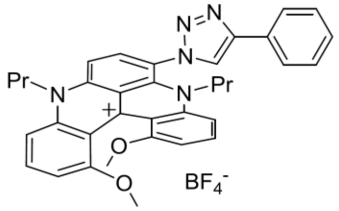

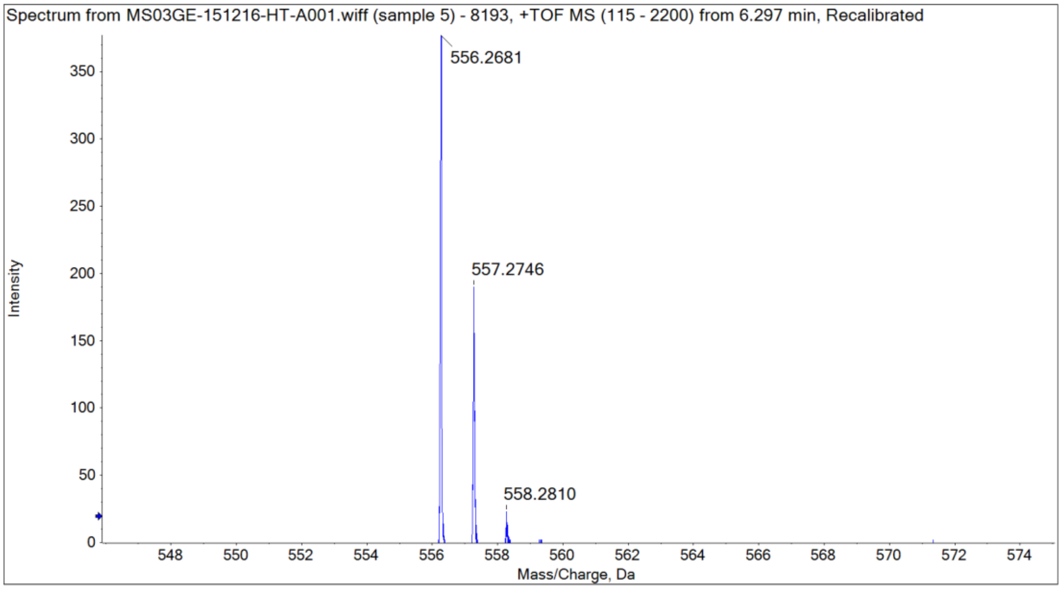

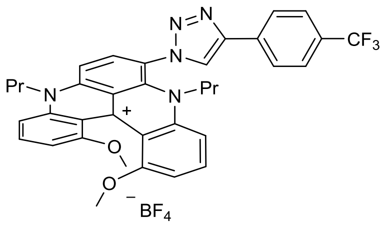

10b

^{1}H NMR (400 MHz, CD_2Cl_2)

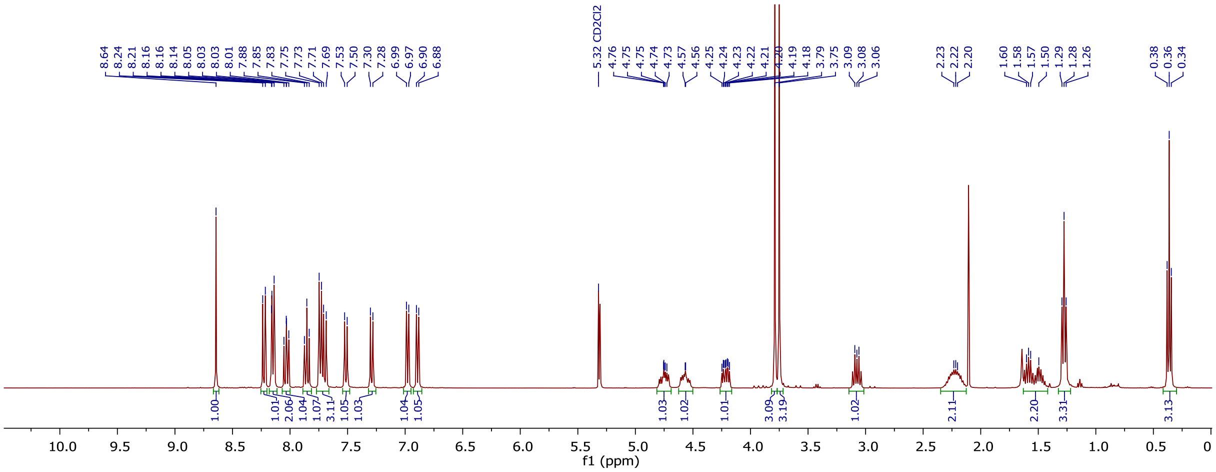

^{13}C NMR (101 MHz, CD_2Cl_2)

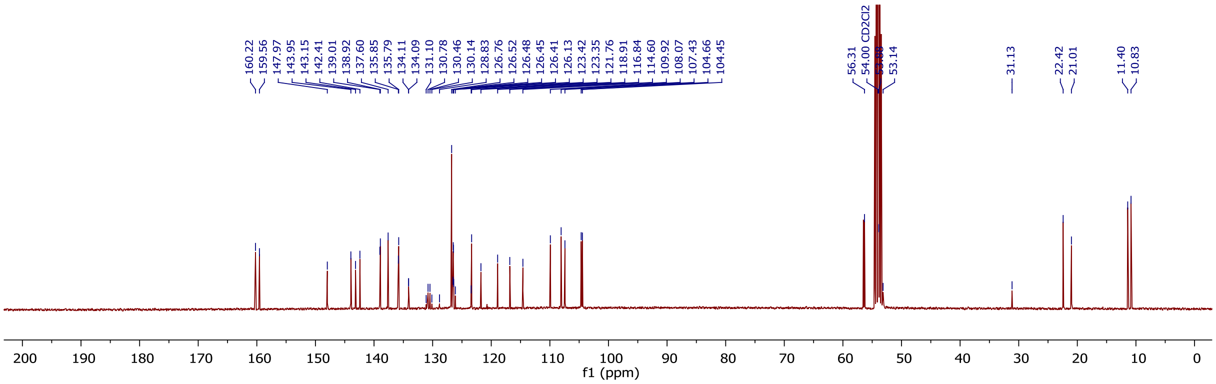

^{19}F NMR (282 MHz, CD_2Cl_2)

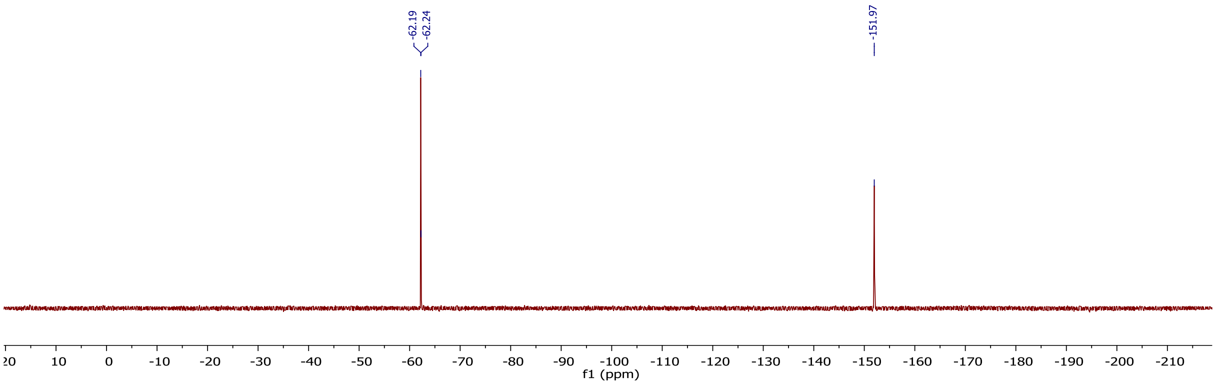

UNIVERSITY OF GENEVA

Faculty of Sciences

Sciences Mass Spectrometry

UNIVERSITÉ DE GENÈVE

Submitter: HERNANDEZ-DELGADO	Date of reception: 03/12/15
Sample name: IH280	Date of certificate: 10/12/15
Sample number: 8161	Data filename: MS03GE-151209-ES-A001
Operator: Eliane Sandmeier	Instrument: QSTAR XL (AB/MDS Sciex)
Principal investigator: Dr. Sophie Michalet	Ionisation mode: ESI (positive mode)

Expected Formula	Observed *m/z* $[M]^+$	Expected *m/z* (amu)	Accuracy (ppm)
$C_{36}H_{33}F_3N_5O_2$	624.2573	624.2581	-1.2

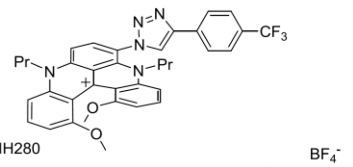

IH280

Chemical Formula: $C_{36}H_{33}F_3N_5O_2^+$
Exact Mass: 624.26
Molecular Weight: 711.49
m/z: 624.26 (100.0%), 625.26 (41.2%), 626.27 (7.5%), 626.26 (1.1%), 627.27 (1.1%)

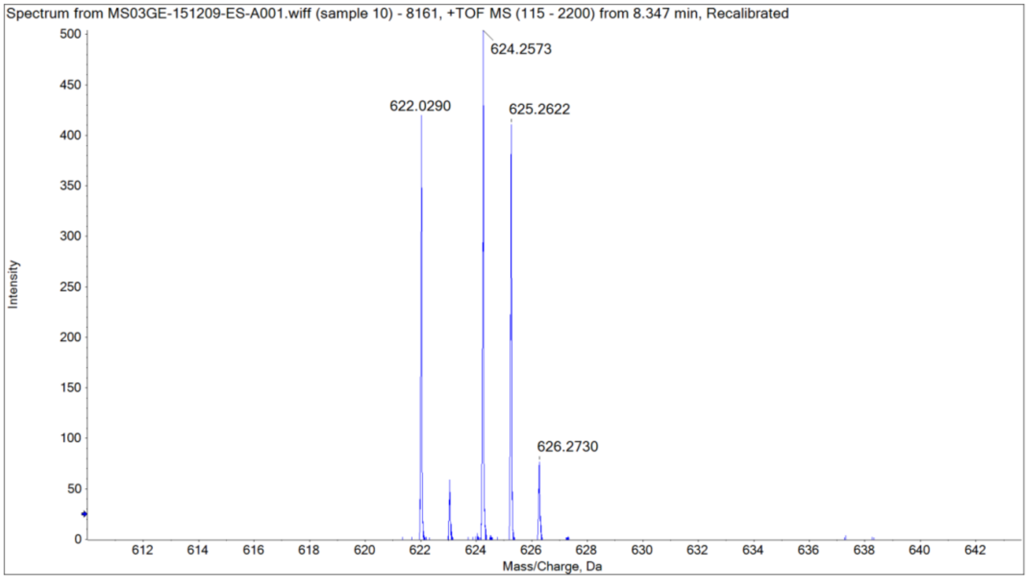

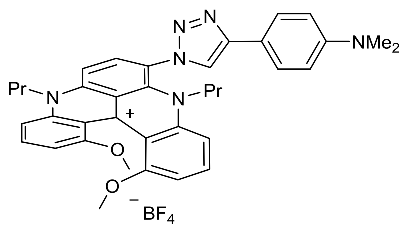

10c

^{1}H NMR (400 MHz, CD_2Cl_2)

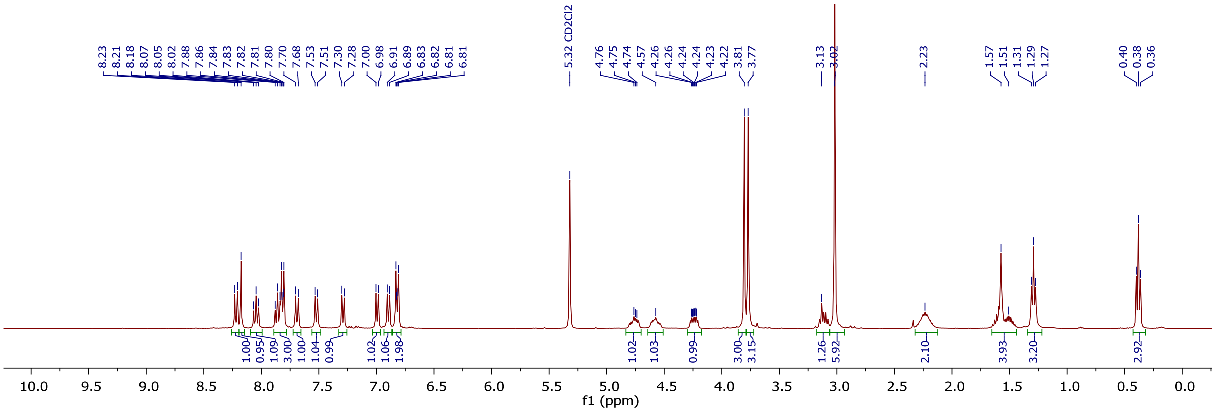

^{13}C NMR (101 MHz, CD_2Cl_2)

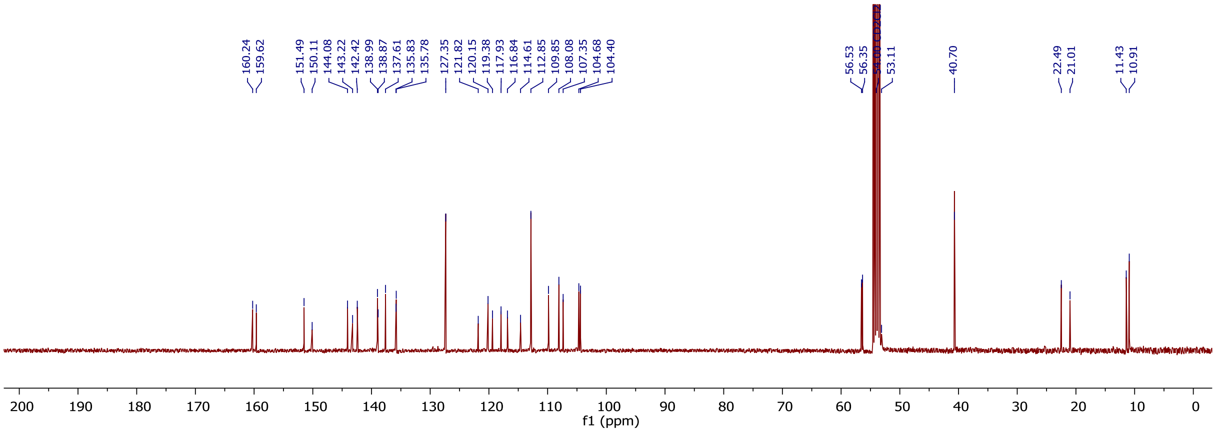

^{19}F NMR (282 MHz, CD_2Cl_2)

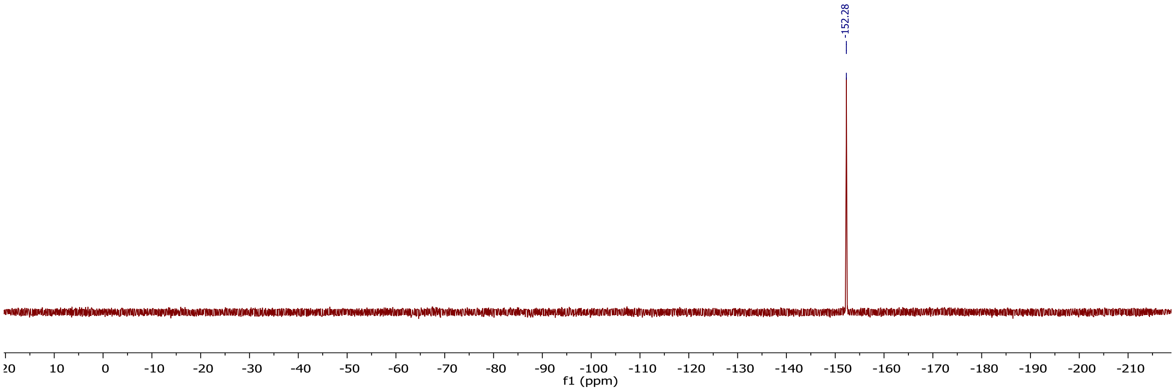

UNIVERSITY OF GENEVA

Faculty of Sciences

Sciences Mass Spectrometry

Submitter: HERNANDEZ-DELGADO	Date of reception: 03/12/15
Sample name: IH281	Date of certificate: 10/12/15
Sample number: 8162	Data filename: MS03GE-151209-ES-A001
Operator: Eliane Sandmeier	Instrument: QSTAR XL (AB/MDS Sciex)
Principal investigator: Dr. Sophie Michalet	Ionisation mode: ESI (positive mode)

Expected Formula	Observed *m/z* $[M]^+$	Expected *m/z* (amu)	Accuracy (ppm)
$C_{37}H_{39}N_6O_2$	599.3155	599.3129	4.4

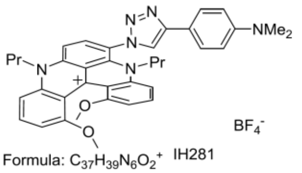

Chemical Formula: $C_{37}H_{39}N_6O_2^+$ IH281
Exact Mass: 599.31

Molecular Weight: 686.56
m/z: 599.31 (100.0%), 600.32 (40.5%),
601.32 (8.4%), 600.31 (2.2%), 602.32 (1.3%)

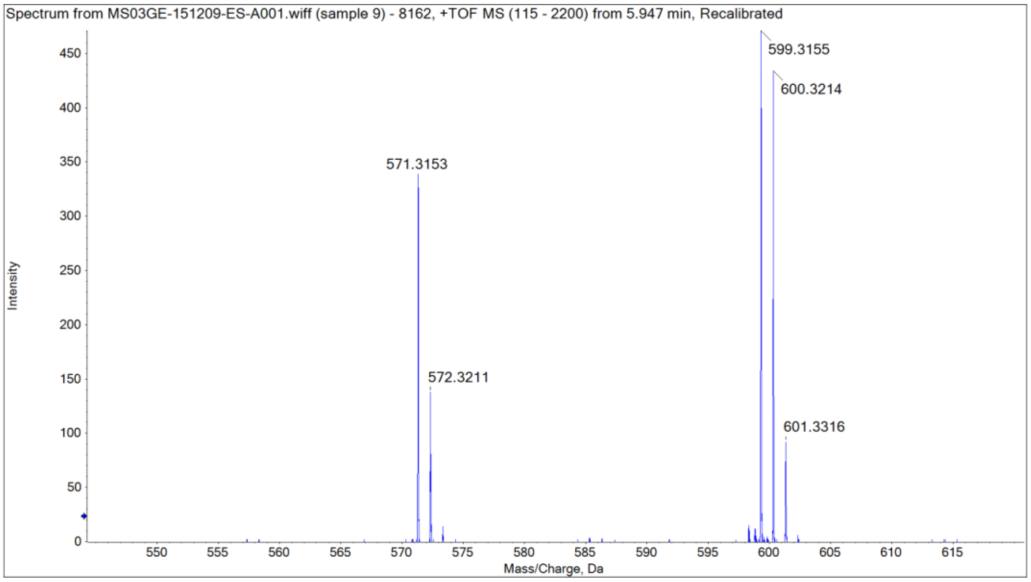

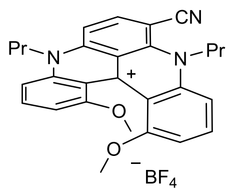

11

^{1}H NMR (400 MHz, CD_2Cl_2)

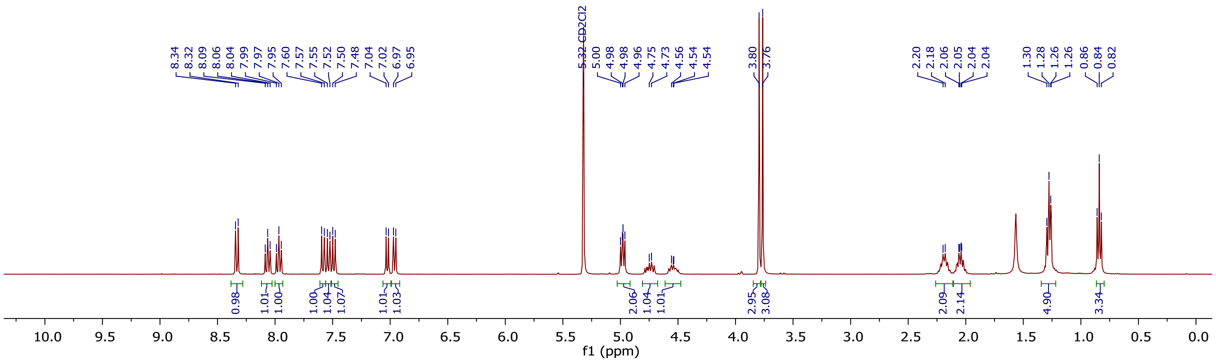

^{13}C NMR (101 MHz, CD_2Cl_2)

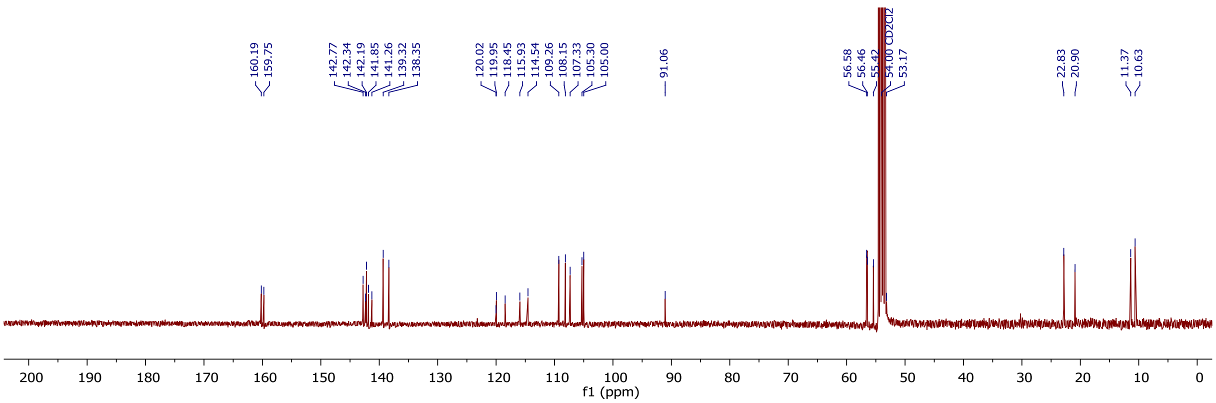

^{19}F NMR (282 MHz, CD_2Cl_2)

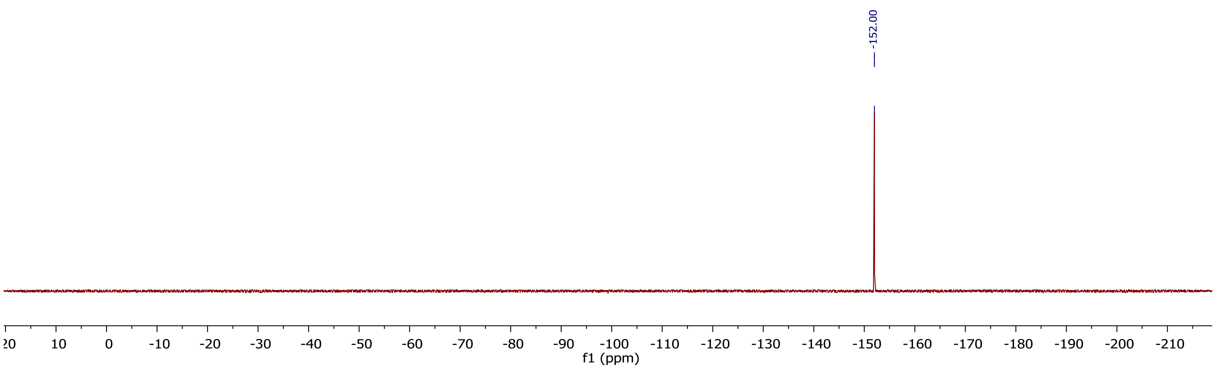

UNIVERSITY OF GENEVA

Faculty of Sciences

Sciences Mass Spectrometry

Submitter:	HERNANDEZ-DELGADO	Date of reception:	03/12/15
Sample name:	IH464	Date of certificate:	10/12/15
Sample number:	8166	Data filename:	MS03GE-151209-ES-A001
Operator:	Eliane Sandmeier	Instrument:	QSTAR XL (AB/MDS Sciex)
Principal investigator:	Dr. Sophie Michalet	Ionisation mode:	ESI (positive mode)

Expected Formula	Observed *m/z* $[M]^+$	Expected *m/z* (amu)	Accuracy (ppm)
$C_{28}H_{28}N_3O_2$	438.2170	438.2176	-1.3

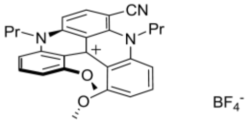

Chemical Formula: $C_{28}H_{28}N_3O_2^+$
Exact Mass: 438.22

IH464
Molecular Weight: 525.35
m/z: 438.22 (100.0%), 439.22 (31.8%), 440.22 (5.2%)

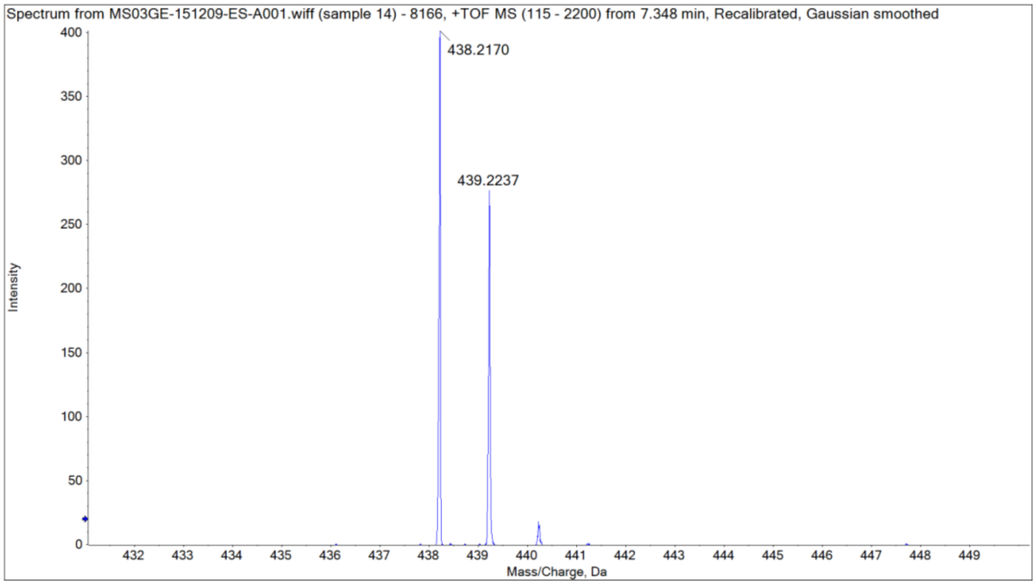

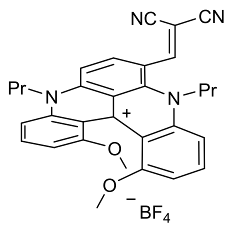

12

^{1}H NMR (400 MHz, CD_2Cl_2)

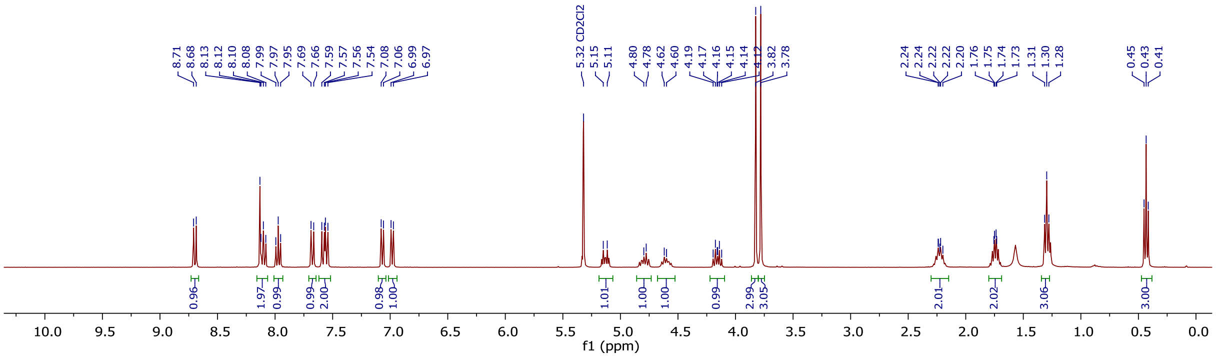

^{13}C NMR (101 MHz, CD_2Cl_2)

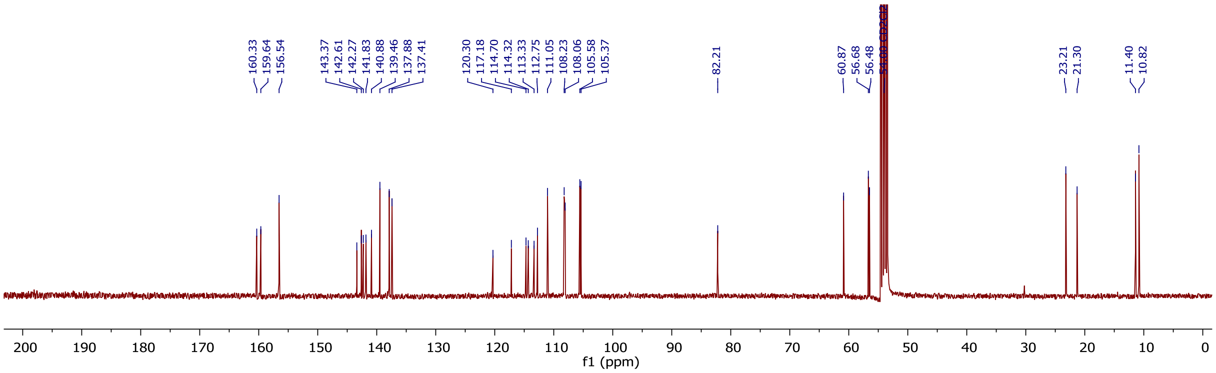

^{19}F NMR (282 MHz, CD_2Cl_2)

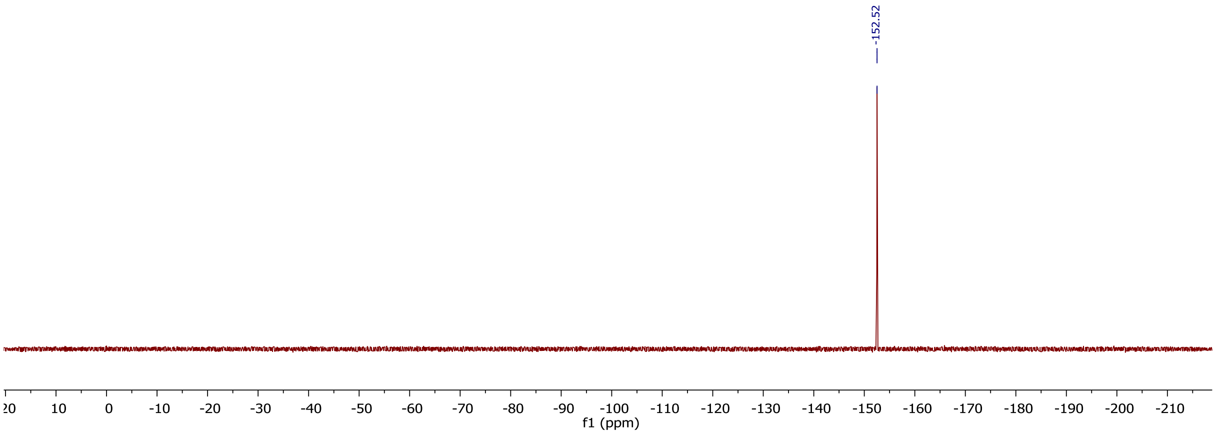

UNIVERSITY OF GENEVA

Faculty of Sciences

Sciences Mass Spectrometry

Submitter: HERNANDEZ DELGADO	Date of reception: 09/12/15
Sample name: IH339	Date of certificate: 17/12/15
Sample number: 8194	Data filename: MS03GE-151216-HT-A001
Operator: Harry Théraulaz	Instrument: QSTAR XL (AB/MDS Sciex)
Principal investigator: Dr. Sophie Michalet	Ionisation mode: ESI (positive mode)

Expected Formula	Observed *m/z* $[M]^+$	Expected *m/z* (amu)	Accuracy (ppm)
$C_{31}H_{29}N_4O_2$	489.2277	489.2285	-1.6

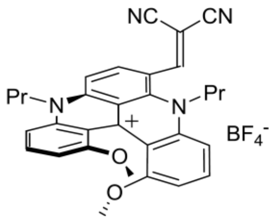

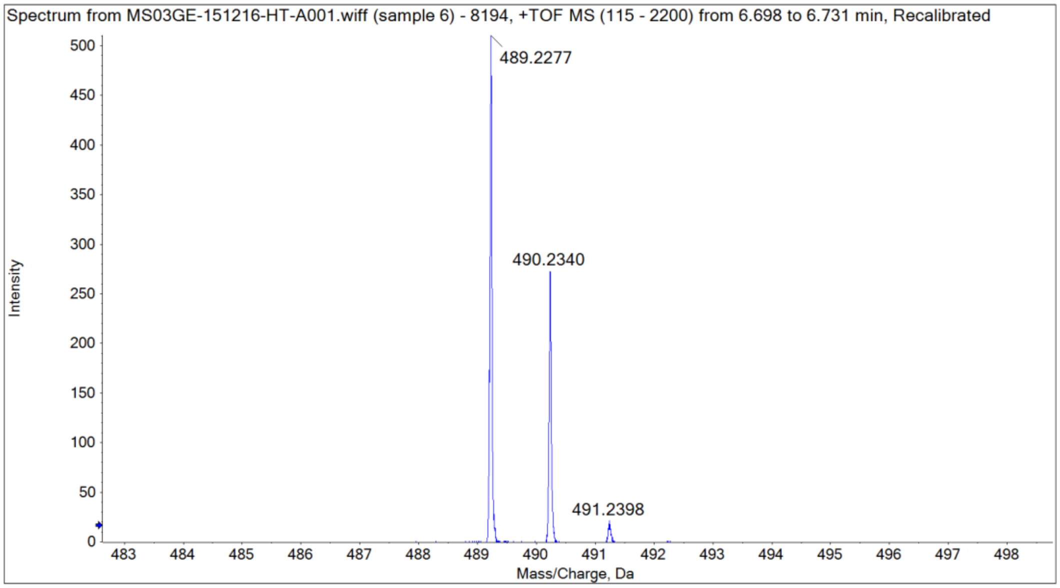

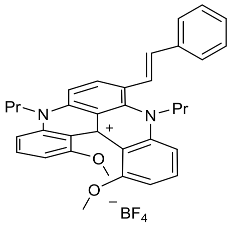

13a

^{1}H NMR (400 MHz, CD_2Cl_2)

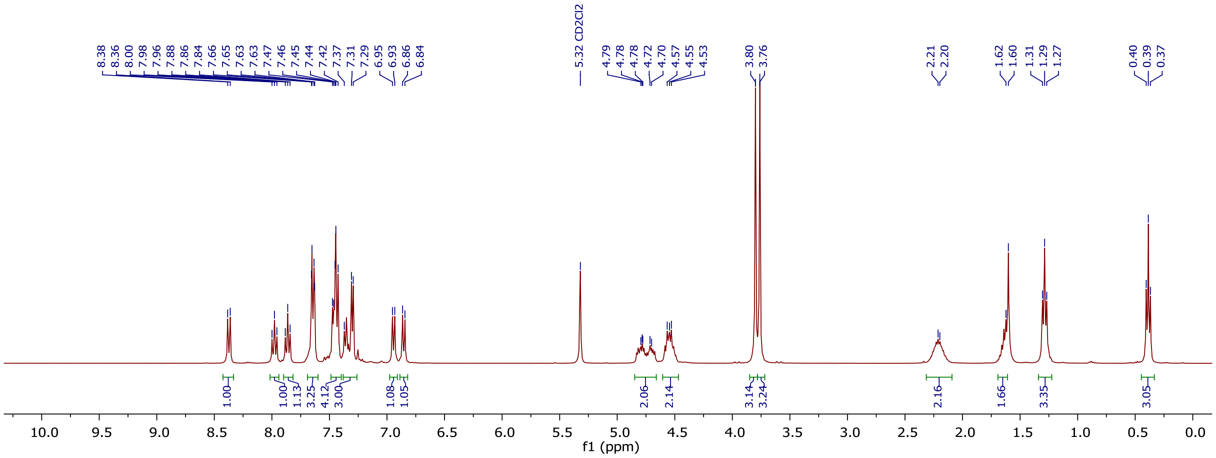

^{13}C NMR (101 MHz, CD_2Cl_2)

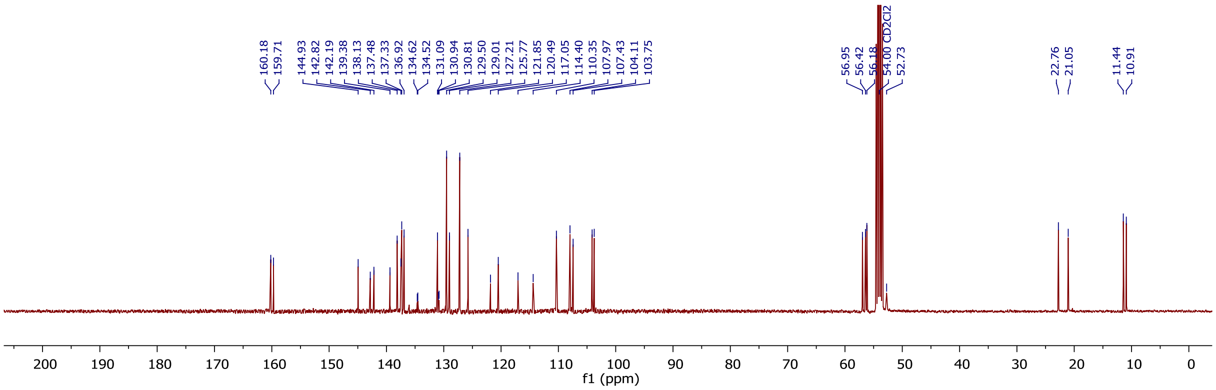

^{19}F NMR (282 MHz, CD_2Cl_2)

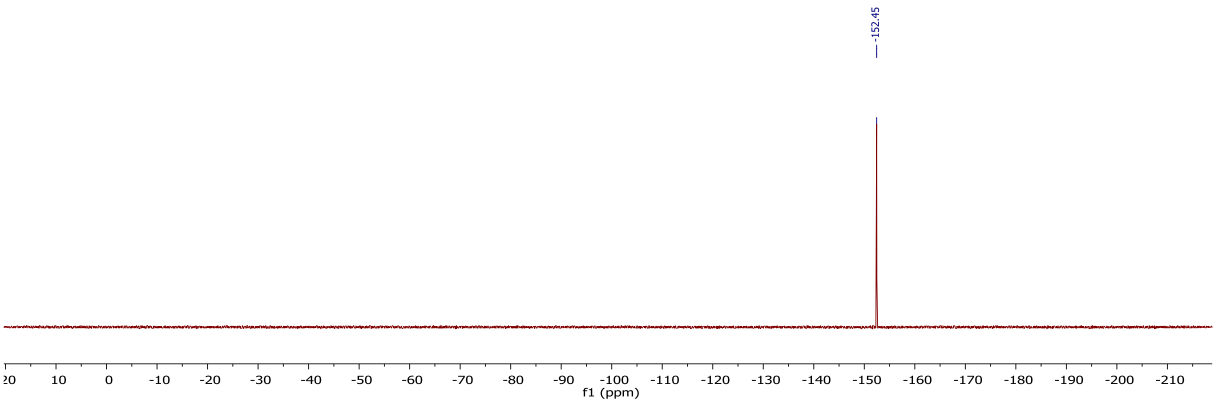

UNIVERSITY OF GENEVA

Faculty of Sciences

Sciences Mass Spectrometry

Submitter:	HERNANDEZ-DELGADO	Date of reception:	03/12/15
Sample name:	IH447	Date of certificate:	10/12/15
Sample number:	8164	Data filename:	MS03GE-151209-ES-A001
Operator:	Eliane Sandmeier	Instrument:	QSTAR XL (AB/MDS Sciex)
Principal investigator:	Dr. Sophie Michalet	Ionisation mode:	ESI (positive mode)

Expected Formula	Observed *m/z* $[M]^+$	Expected *m/z* (amu)	Accuracy (ppm)
$C_{35}H_{35}N_2O_2$	515.2693	515.2693	-0.1

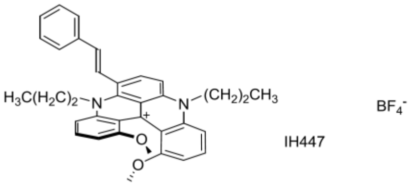

IH447

Chemical Formula: $C_{35}H_{35}N_2O_2^+$
Exact Mass: 515.27
Molecular Weight: 602.48
m/z: 515.27 (100.0%), 516.27 (38.7%), 517.28 (7.1%), 518.28 (1.0%)

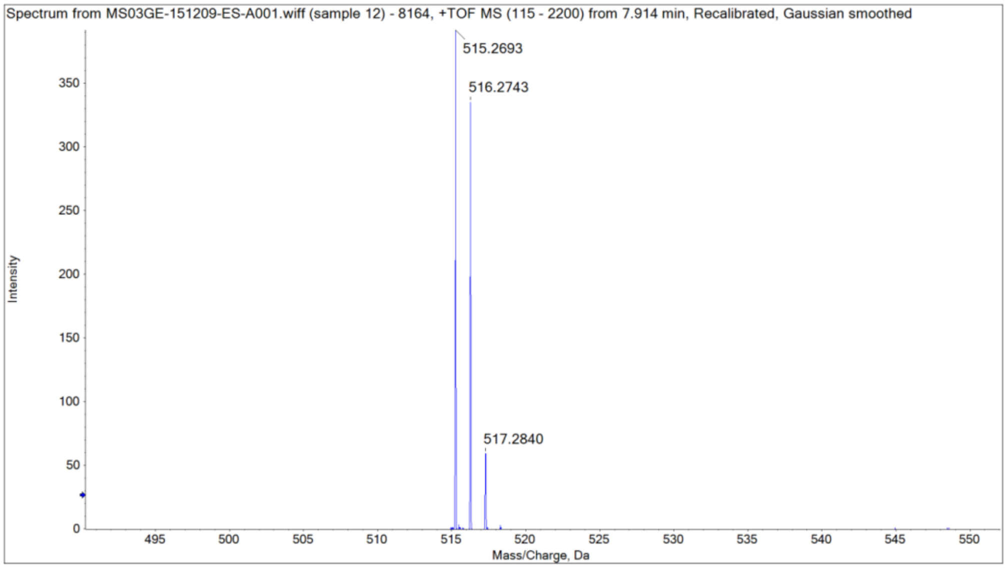

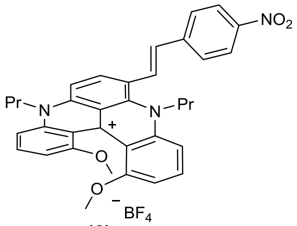

13b

¹H NMR (400 MHz, CD_2Cl_2)

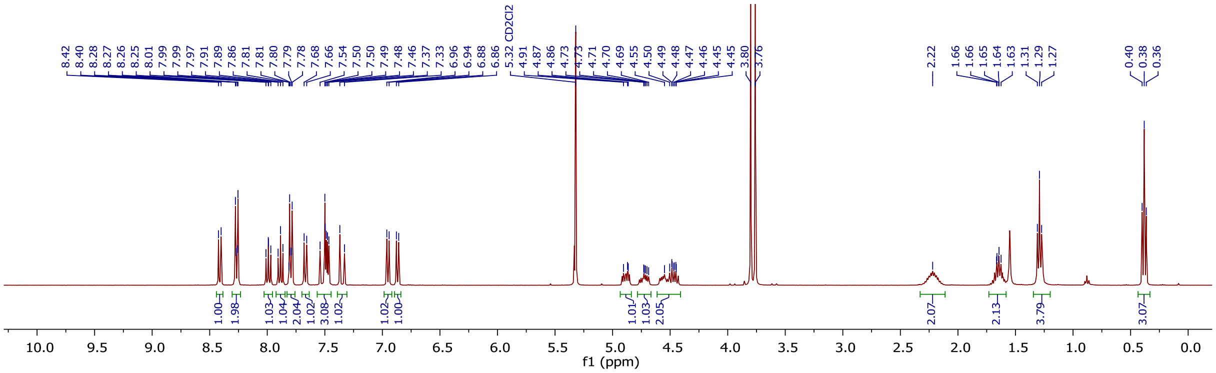

¹³C NMR (101 MHz, CD_2Cl_2)

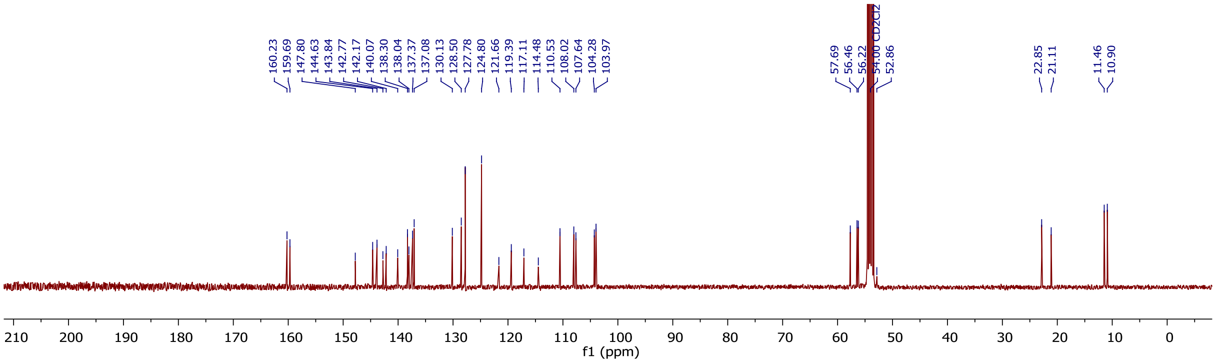

¹⁹F NMR (282 MHz, CD_2Cl_2)

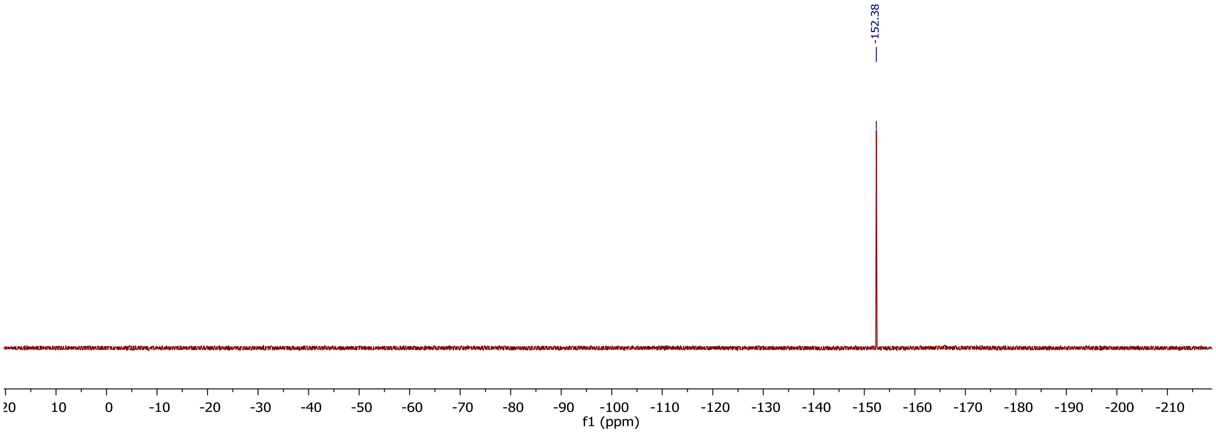

UNIVERSITY OF GENEVA

Faculty of Sciences

Sciences Mass Spectrometry

UNIVERSITÉ DE GENÈVE

SMS MS

Submitter:	HERNANDEZ DELGADO	Date of reception:	03/12/15
Sample name:	IH195	Date of certificate:	09/12/15
Sample number:	8156	Data filename:	MS03GE-151208-HT-A001
Operator:	Harry Théraulaz	Instrument:	QSTAR XL (AB/MDS Sciex)
Principal investigator:	Dr. Sophie Michalet	Ionisation mode:	ESI (positive mode)

Expected Formula	Observed *m/z* $[M]^+$	Expected *m/z* (amu)	Accuracy (ppm)
$C_{35}H_{34}N_3O_4$	560.2543	560.2544	-0.1

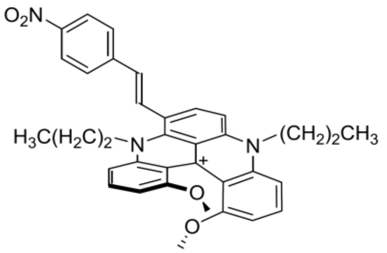

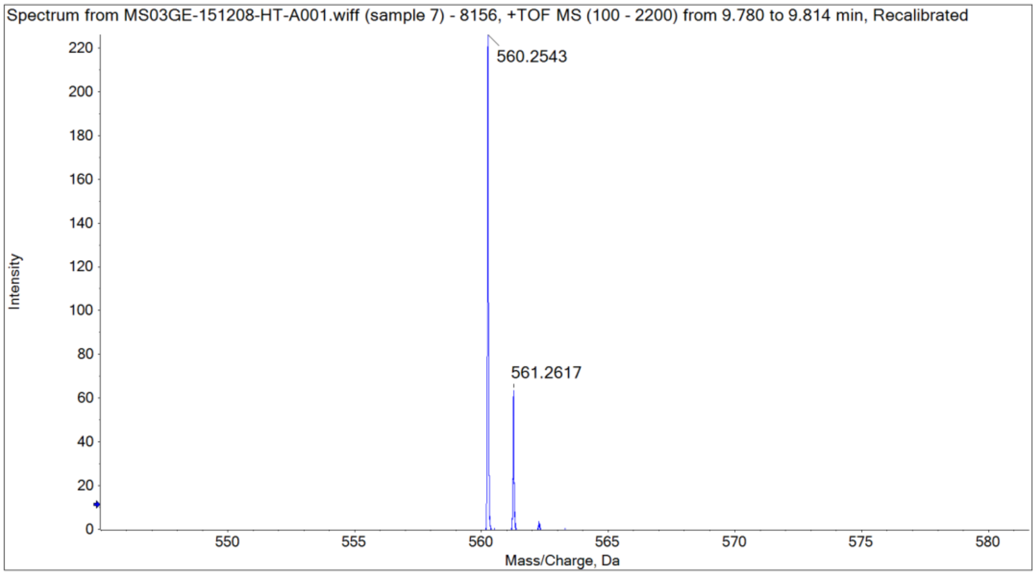

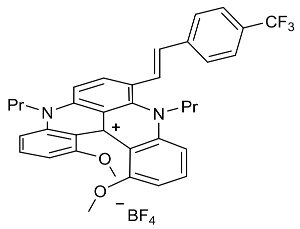

13c

^{1}H NMR (400 MHz, CD_2Cl_2)

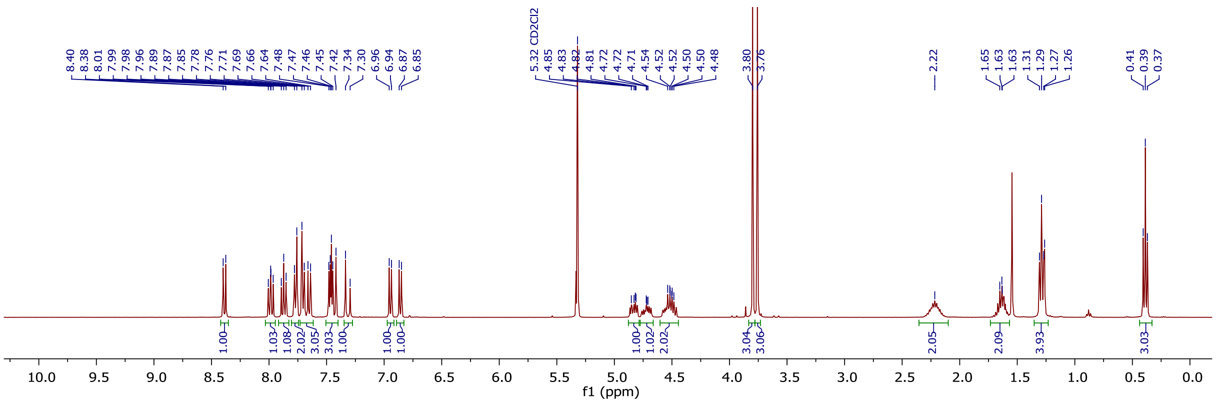

^{13}C NMR (101 MHz, CD_2Cl_2)

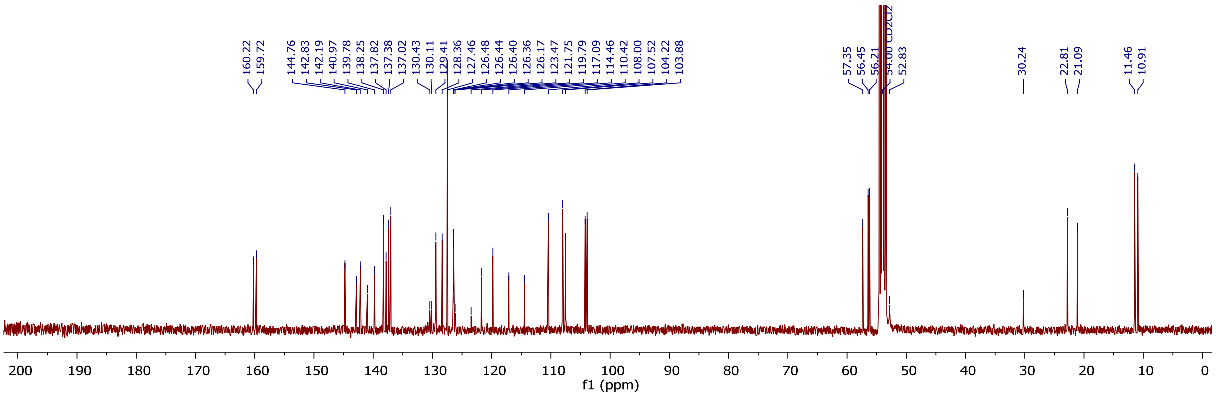

^{19}F NMR (282 MHz, CD_2Cl_2)

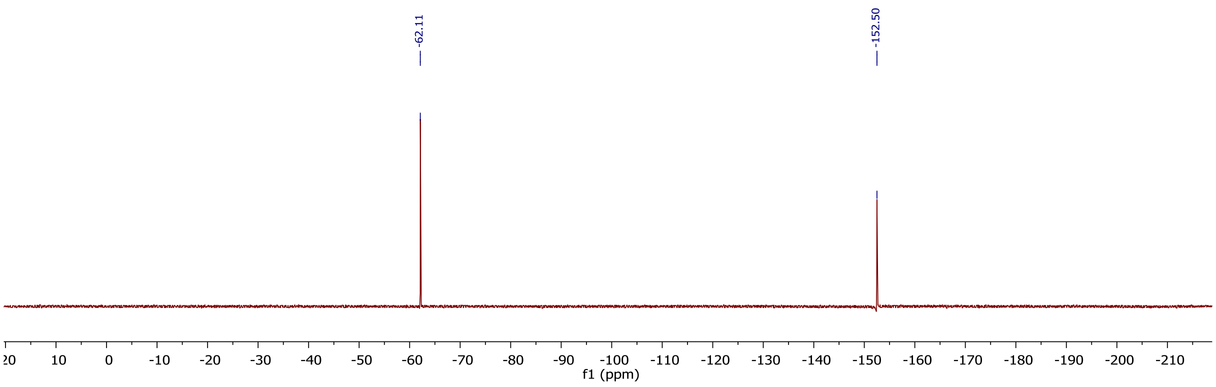

UNIVERSITY OF GENEVA

Faculty of Sciences

Sciences Mass Spectrometry

Submitter: HERNANDEZ DELGADO	Date of reception: 03/12/15
Sample name: IH198	Date of certificate: 09/12/15
Sample number: 8159	Data filename: MS03GE-151208-HT-A001
Operator: Harry Théraulaz	Instrument: QSTAR XL (AB/MDS Sciex)
Principal investigator: Dr. Sophie Michalet	Ionisation mode: ESI (positive mode)

Expected Formula	Observed *m/z* $[M]^+$	Expected *m/z* (amu)	Accuracy (ppm)
$C_{36}H_{34}F_3N_2O_2$	583.2586	583.2567	3.3

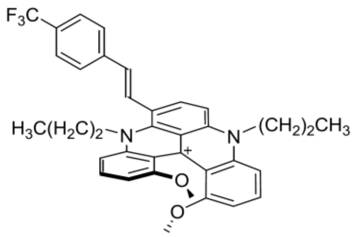

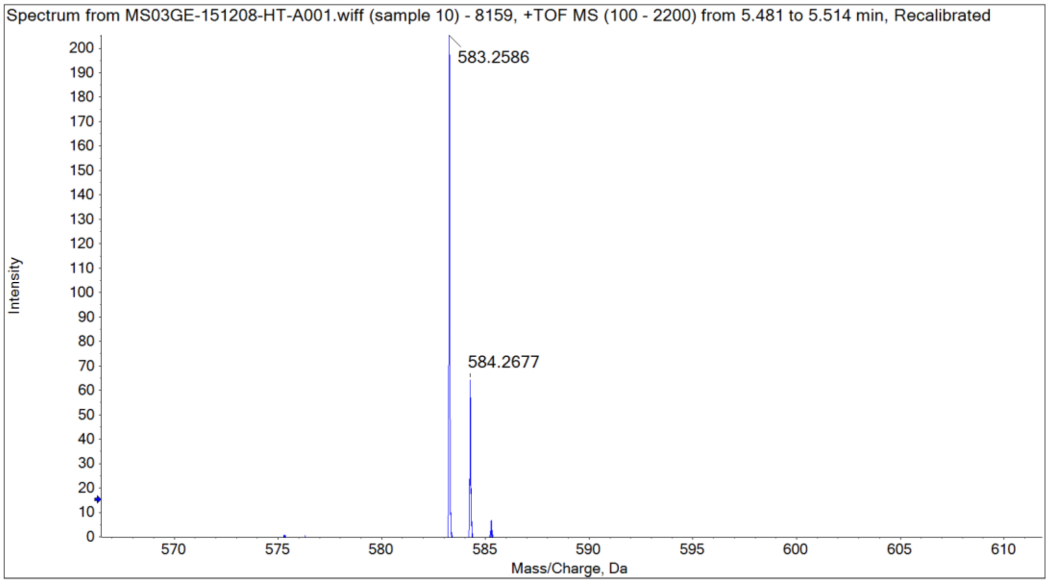

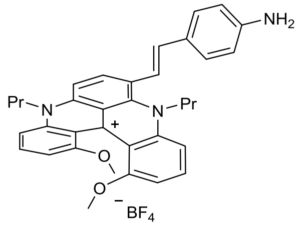

13d

^{1}H NMR (400 MHz, CD_2Cl_2)

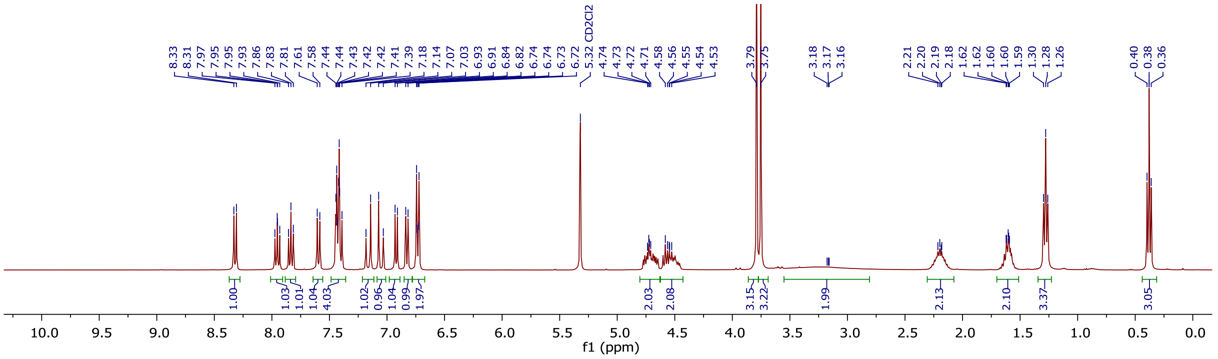

^{13}C NMR (101 MHz, CD_2Cl_2)

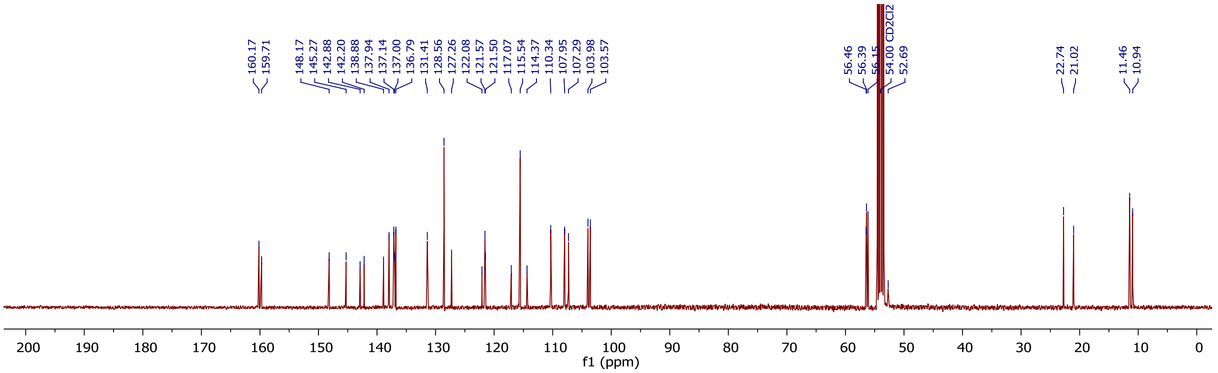

^{19}F NMR (282 MHz, CD_2Cl_2)

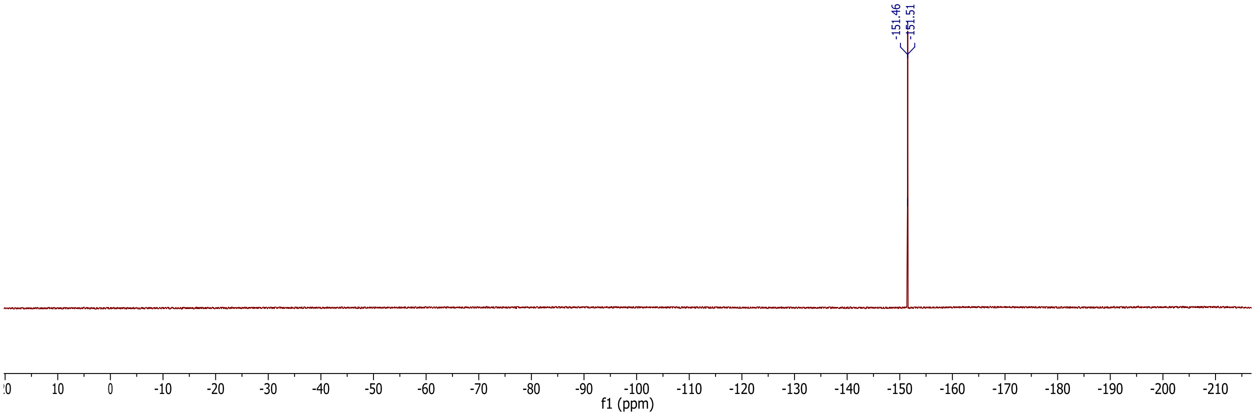

UNIVERSITY OF GENEVA

Faculty of Sciences

Sciences Mass Spectrometry

UNIVERSITÉ DE GENÈVE

Submitter:	HERNANDEZ DELGADO	Date of reception:	09/12/15
Sample name:	IH463	Date of certificate:	17/12/15
Sample number:	8195	Data filename:	MS03GE-151216-HT-A001
Operator:	Harry Théraulaz	Instrument:	QSTAR XL (AB/MDS Sciex)
Principal investigator:	Dr. Sophie Michalet	Ionisation mode:	ESI (positive mode)

Expected Formula	Observed *m/z* $[M]^+$	Expected *m/z* (amu)	Accuracy (ppm)
$C_{35}H_{36}N_3O_2$	530.2804	530.2802	0.3

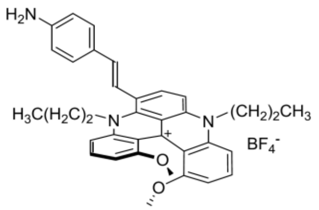

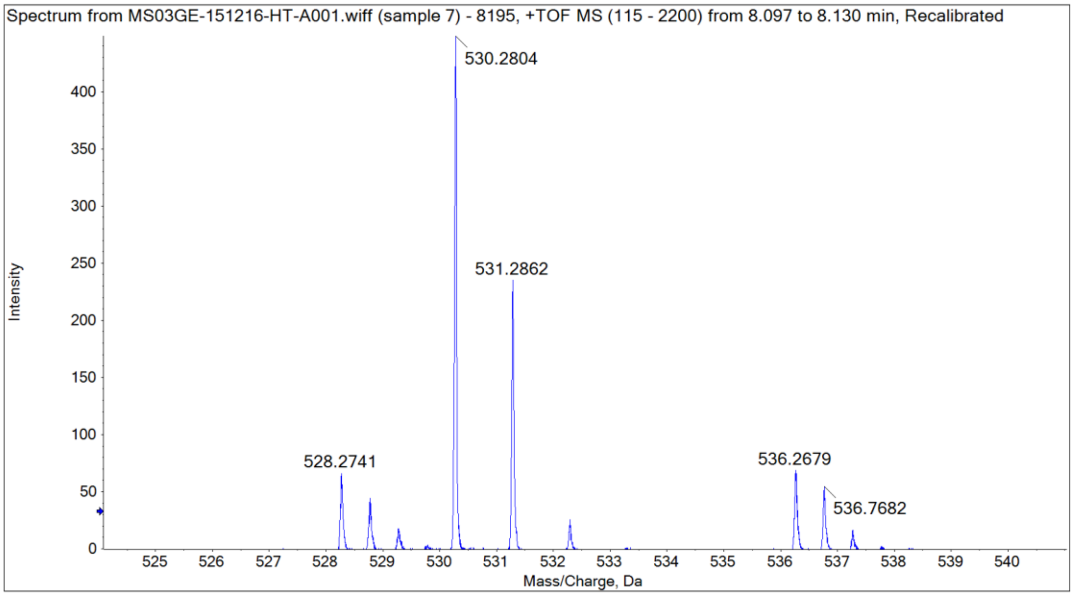

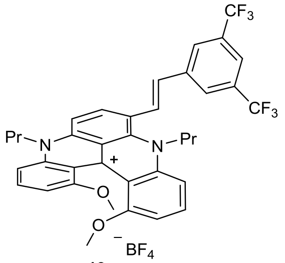

13e

^{1}**H NMR** (400 MHz, CD_2Cl_2)

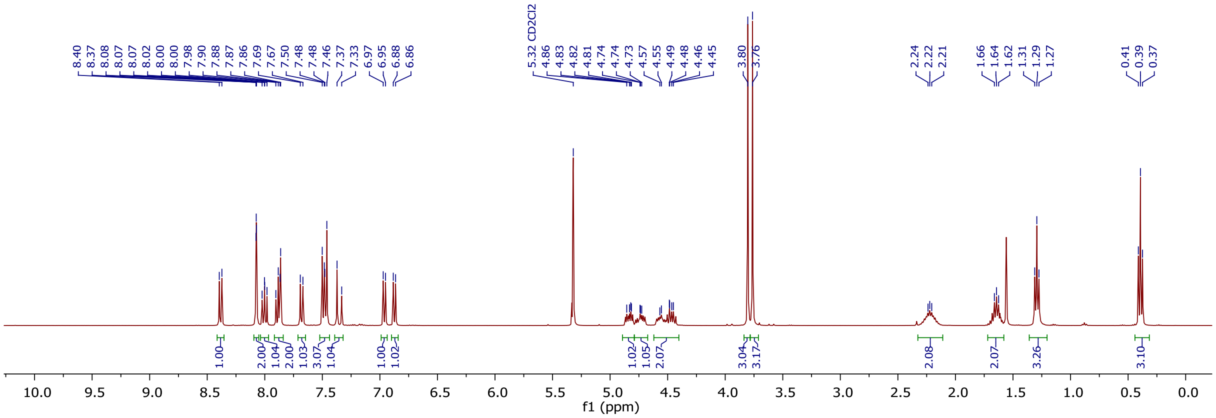

^{13}**C NMR** (101 MHz, CD_2Cl_2)

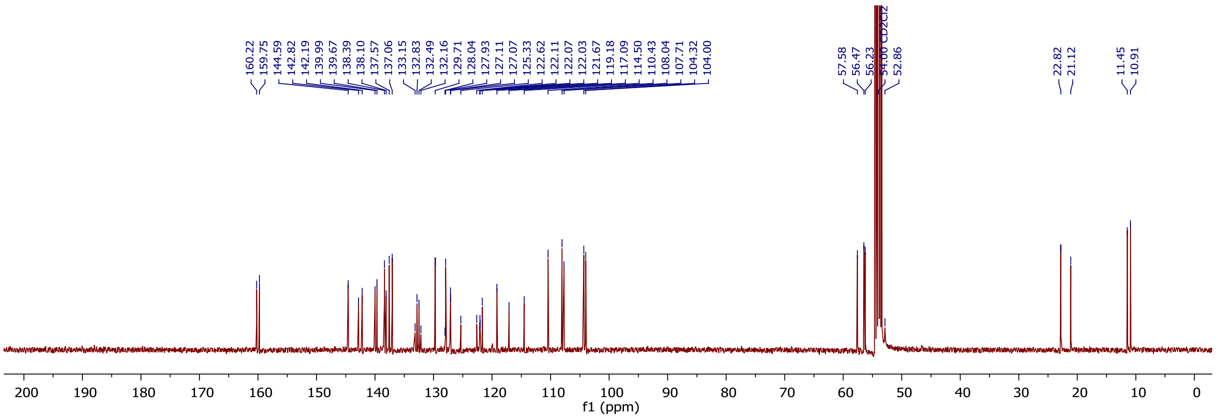

^{19}**F NMR** (282 MHz, CD_2Cl_2)

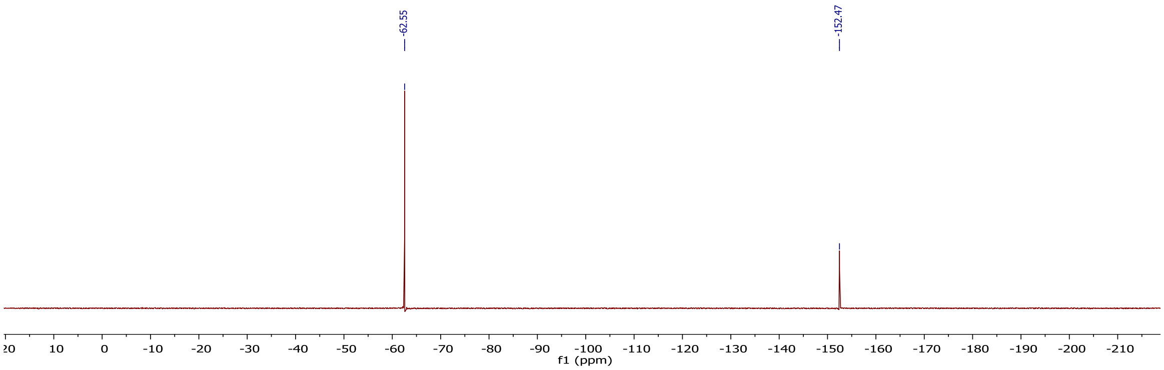

UNIVERSITY OF GENEVA

Faculty of Sciences

Sciences Mass Spectrometry

UNIVERSITÉ DE GENÈVE

Submitter:	HERNANDEZ DELGADO	Date of reception:	03/12/15
Sample name:	IH196	Date of certificate:	09/12/15
Sample number:	8157	Data filename:	MS03GE-151208-HT-A001
Operator:	Harry Théraulaz	Instrument:	QSTAR XL (AB/MDS Sciex)
Principal investigator:	Dr. Sophie Michalet	Ionisation mode:	ESI (positive mode)

Expected Formula	Observed *m/z* $[M]^+$	Expected *m/z* (amu)	Accuracy (ppm)
$C_{37}H_{33}F_6N_2O_2$	651.2415	651.2441	-3.9

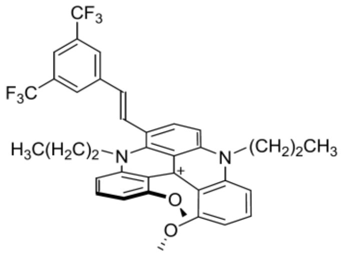

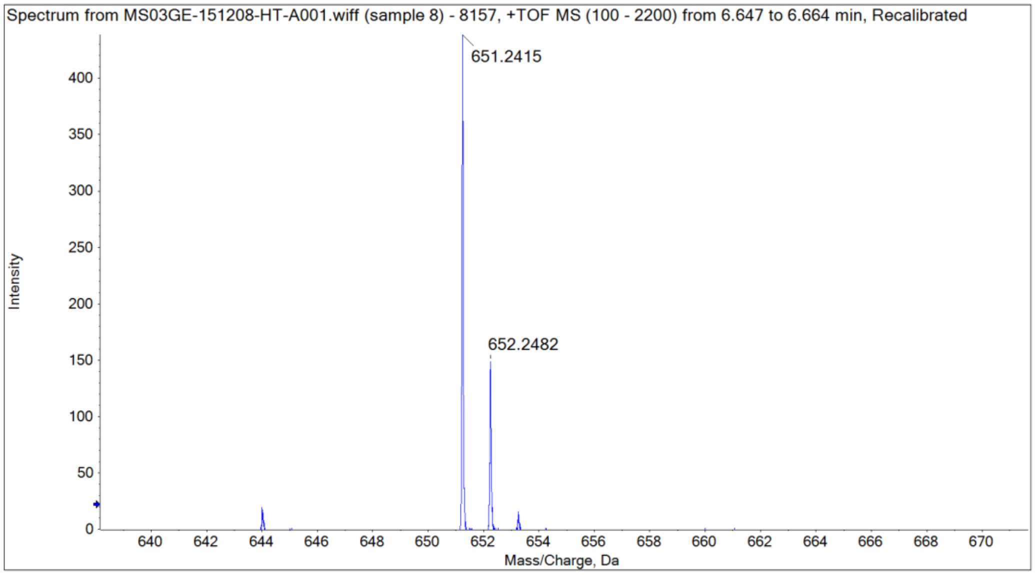

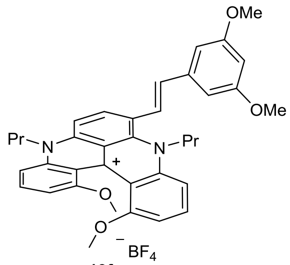

13f

¹H NMR (400 MHz, CD_2Cl_2)

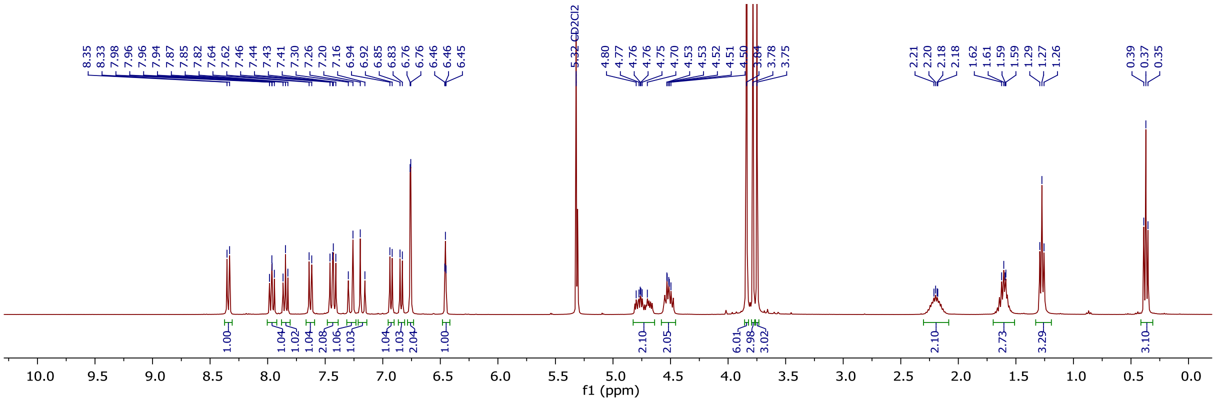

¹³C NMR (101 MHz, CD_2Cl_2)

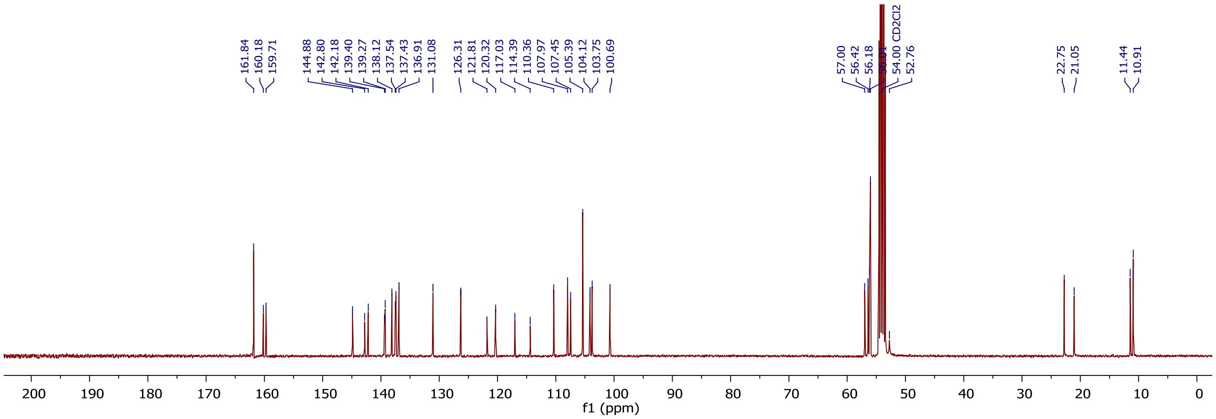

¹⁹F NMR (282 MHz, CD_2Cl_2)

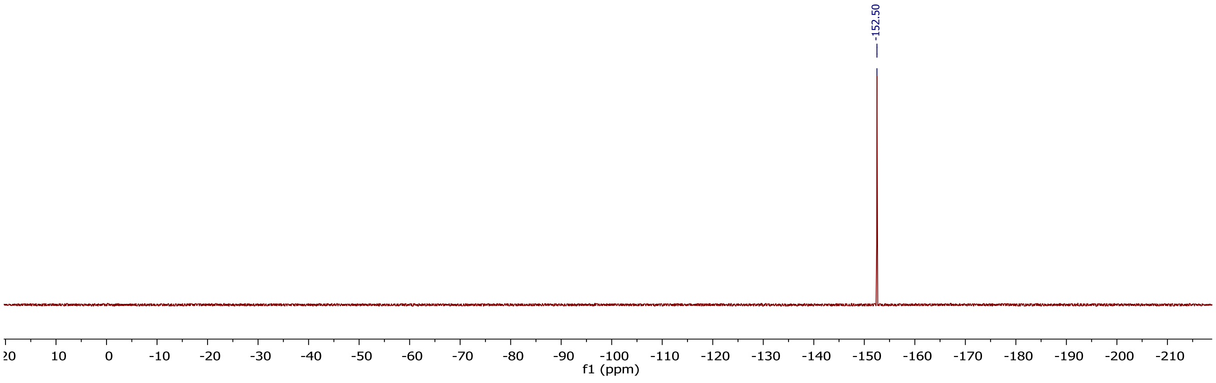

UNIVERSITY OF GENEVA

Faculty of Sciences

Sciences Mass Spectrometry

Submitter:	HERNANDEZ DELGADO	Date of reception:	03/12/15
Sample name:	IH197	Date of certificate:	09/12/15
Sample number:	8158	Data filename:	MS03GE-151208-HT-A001
Operator:	Harry Théraulaz	Instrument:	QSTAR XL (AB/MDS Sciex)
Principal investigator:	Dr. Sophie Michalet	Ionisation mode:	ESI (positive mode)

Expected Formula	Observed *m/z* $[M]^+$	Expected *m/z* (amu)	Accuracy (ppm)
$C_{37}H_{39}N_2O_4$	575.2886	575.2904	-3.2

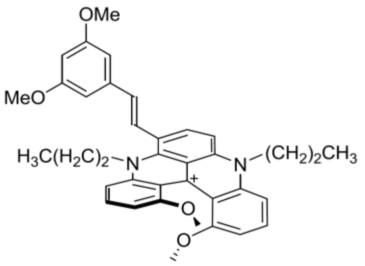

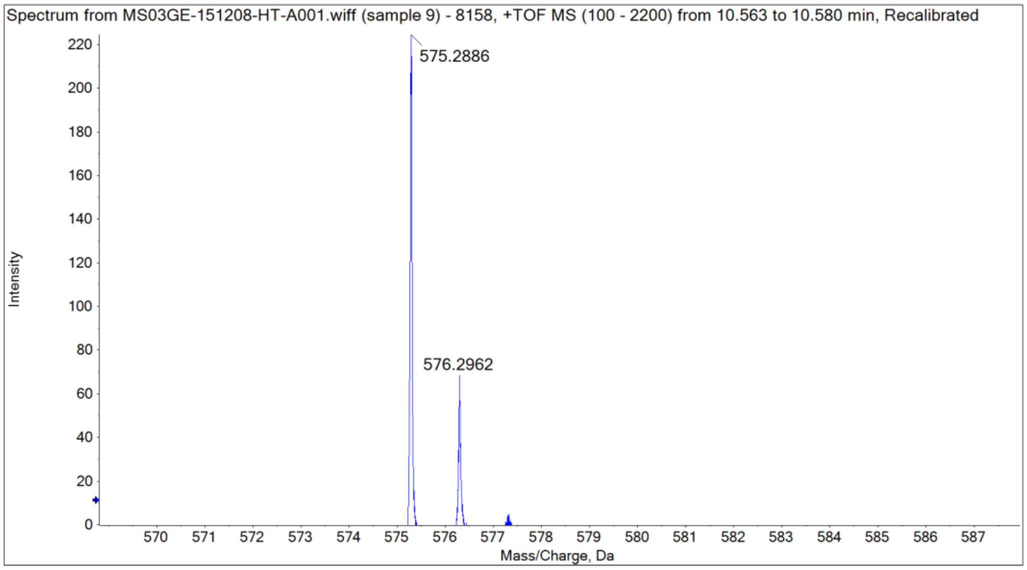

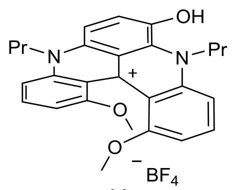

14

^{1}H NMR (500 MHz, CD_2Cl_2)

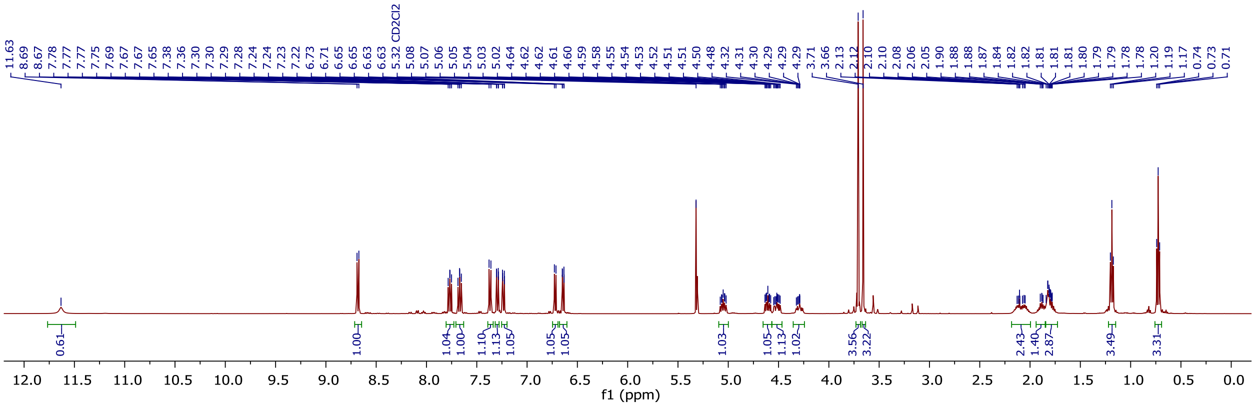

^{13}C NMR (125 MHz, CD_2Cl_2)

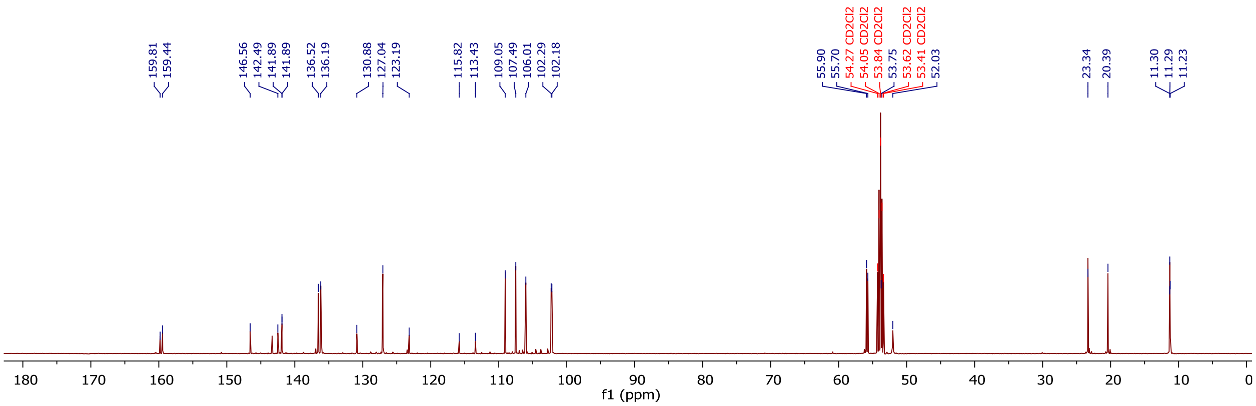

^{19}F NMR (282 MHz, CD_2Cl_2)

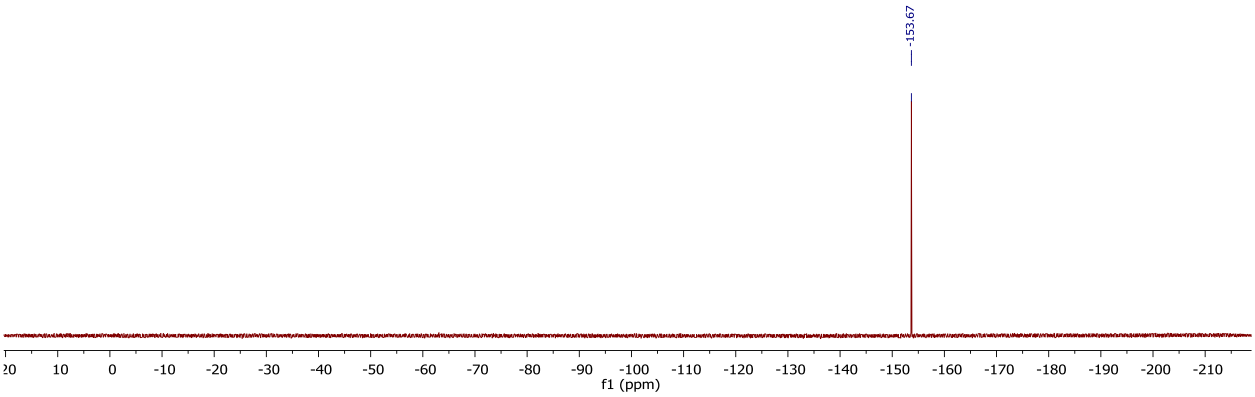

UNIVERSITY OF GENEVA

Faculty of Sciences

Sciences Mass Spectrometry

UNIVERSITÉ DE GENÈVE

SMS MS

Submitter: Simon Pascal	Date of reception: 29/05/15
Sample name: SPA094	Date of certificate: 10/06/15
Sample number: 7904	Data filename: SMS10GE-150608-ES-A002
Operator: Julien Meyer	Instrument: QSTAR Pulsar (AB/MDS Sciex)
Principal investigator: Dr. Sophie Michalet	Ionisation mode: ESI (positive mode)

Expected Formula	Observed *m/z* $[M+H]^+$	Expected *m/z* (amu)	Accuracy (ppm)
$C_{27}H_{29}N_2O_3^+$	429.2159	429.2173	-3.1

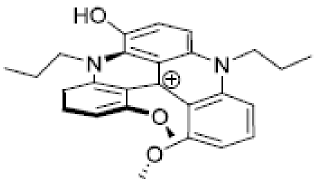

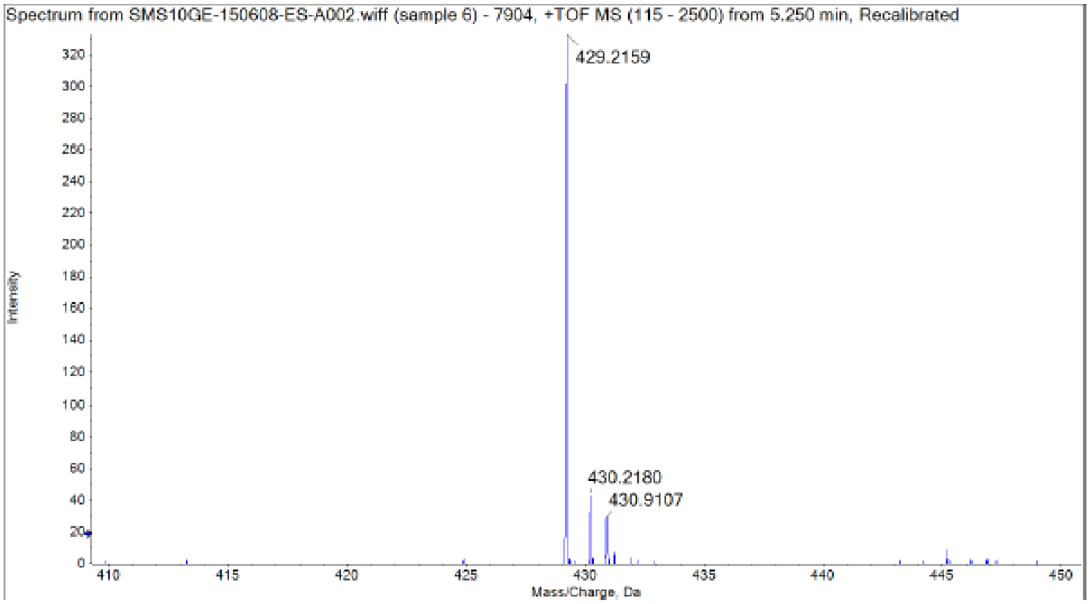

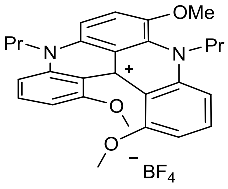

15

^{1}H NMR (500 MHz, CD_2Cl_2/CD_3OD, 1:1)

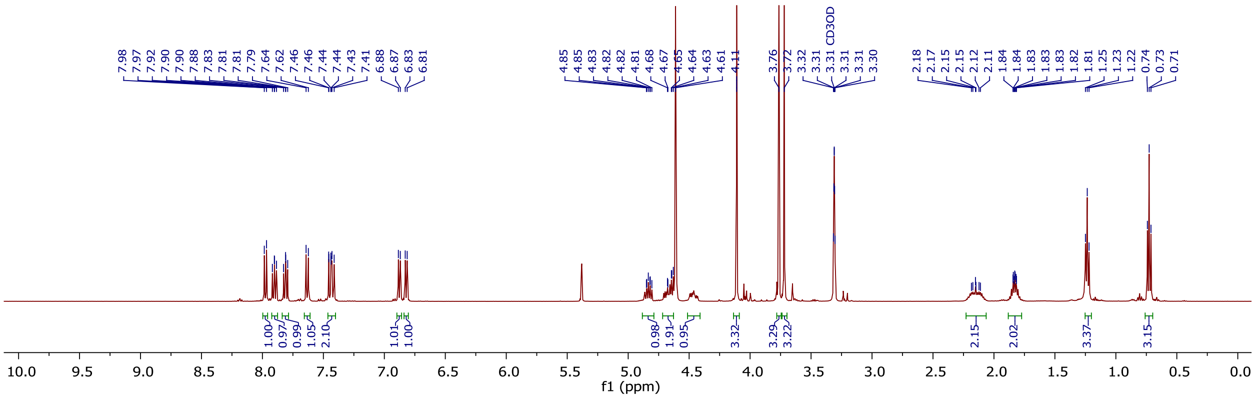

^{13}C NMR (125 MHz, CD_2Cl_2/CD_3OD, 1:1)

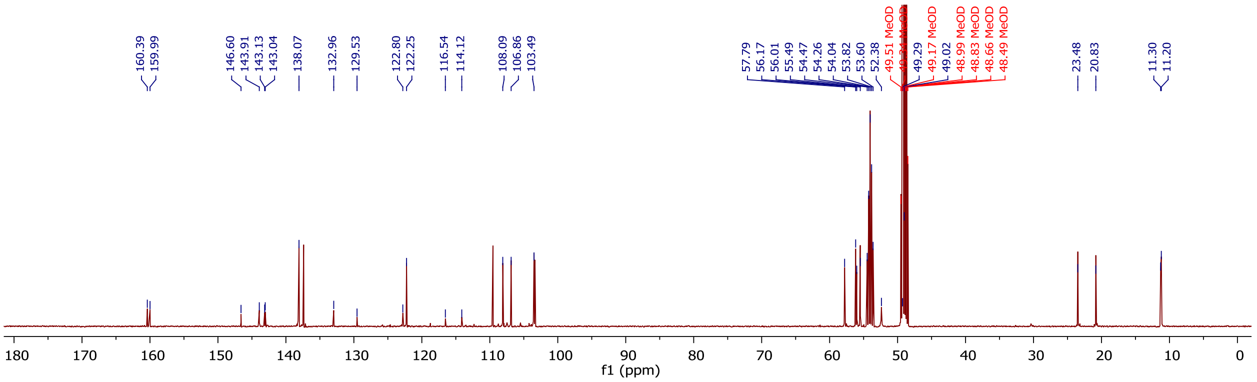

^{19}F NMR (282 MHz, CD_2Cl_2/CD_3OD, 1:1)

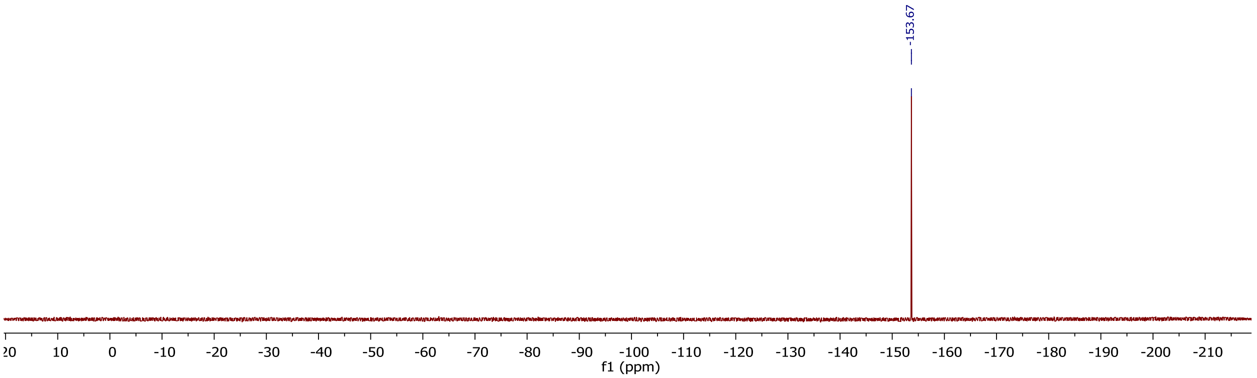

UNIVERSITY OF GENEVA

Faculty of Sciences

Sciences Mass Spectrometry

Submitter:	PASCAL	Date of reception:	12/06/15
Sample name:	SPA099	Date of certificate:	18/06/15
Sample number:	7926	Data filename:	SMS10GE-150617-ES-A003
Operator:	Eliane Sandmeier	Instrument:	QSTAR Pulsar (AB/MDS Sciex)
Principal investigator:	Dr. Sophie Michalet	Ionisation mode:	ESI (positive mode)

Expected Formula	Observed *m/z* $[M]^+$	Expected *m/z* (amu)	Accuracy (ppm)
$C_{28}H_{31}N_2O_3$	443.2326	443.2329	-0.6

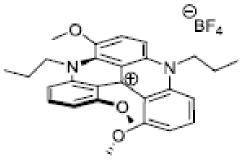

SPA099

Chemical Formula: $C_{28}H_{31}N_2O_3^+$

Exact Mass: 443.2329

Molecular Weight: 443.5568

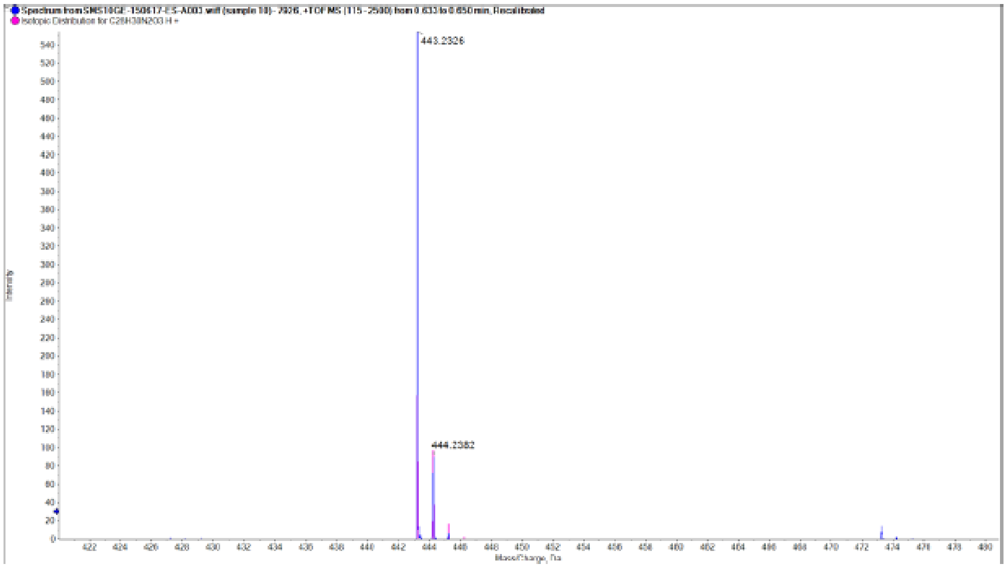

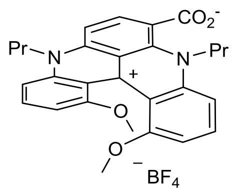

16

^{1}H NMR (500 MHz, CD_3OD)

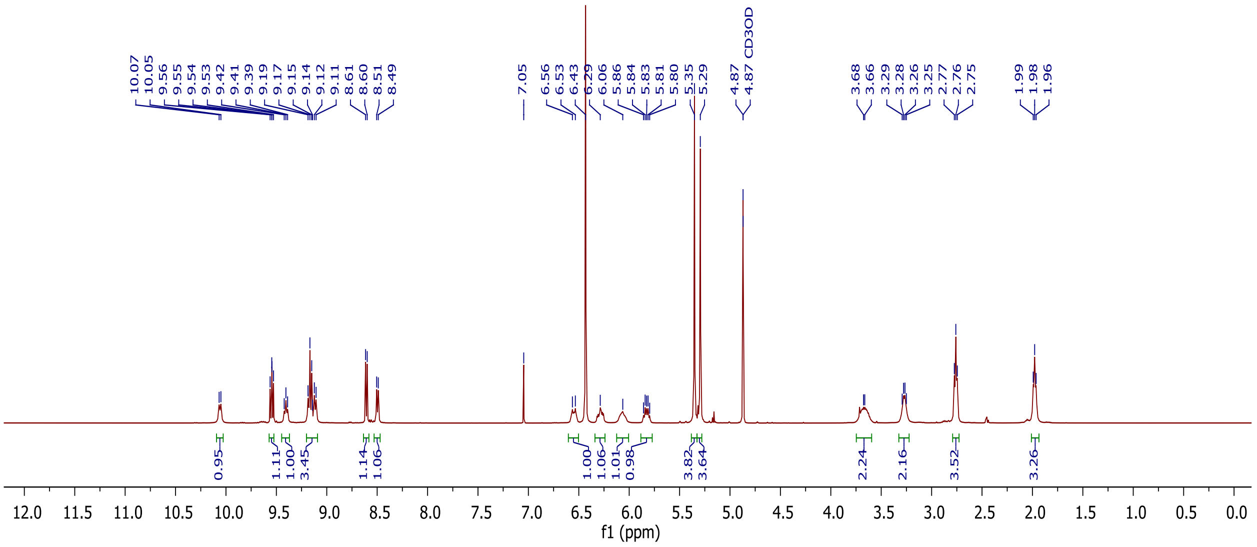

^{13}C NMR (125 MHz, CD_3OD)

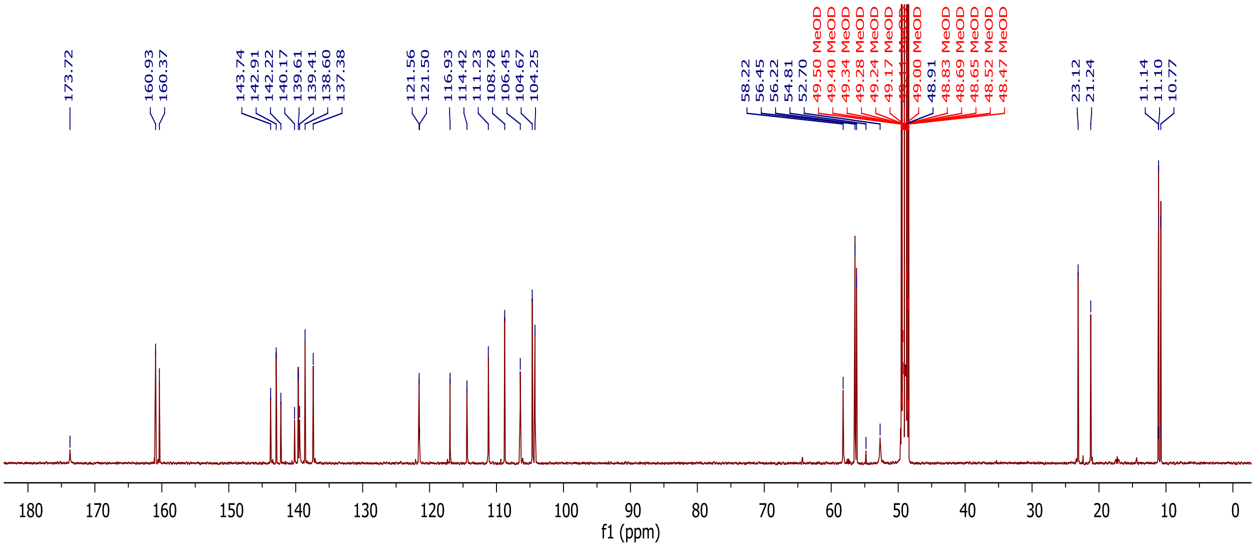

UNIVERSITY OF GENEVA

Faculty of Sciences

Sciences Mass Spectrometry

Submitter:	PASCAL	Date of reception:	13/02/15
Sample name:	SPA036	Date of certificate:	17/02/15
Sample number:	7693	Data filename:	SMS10GE-150216-ES-A002
Operator:	Eliane Sandmeier	Instrument:	QSTAR Pulsar (AB/MDS Sciex)
Principal investigator:	Dr. Sophie Michalet	Ionisation mode:	ESI (positive mode)

Expected Formula	Observed *m/z* $[M]^+$	Expected *m/z* (amu)	Accuracy (ppm)
$C_{28}H_{29}N_2O_4$	457.2120	457.2122	-0.4

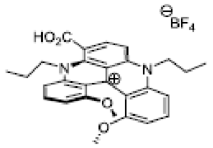

SPA036
Chemical Formula: $C_{28}H_{29}N_2O_4^+$
Exact Mass: 457,2122
Molecular Weight: 457,5403

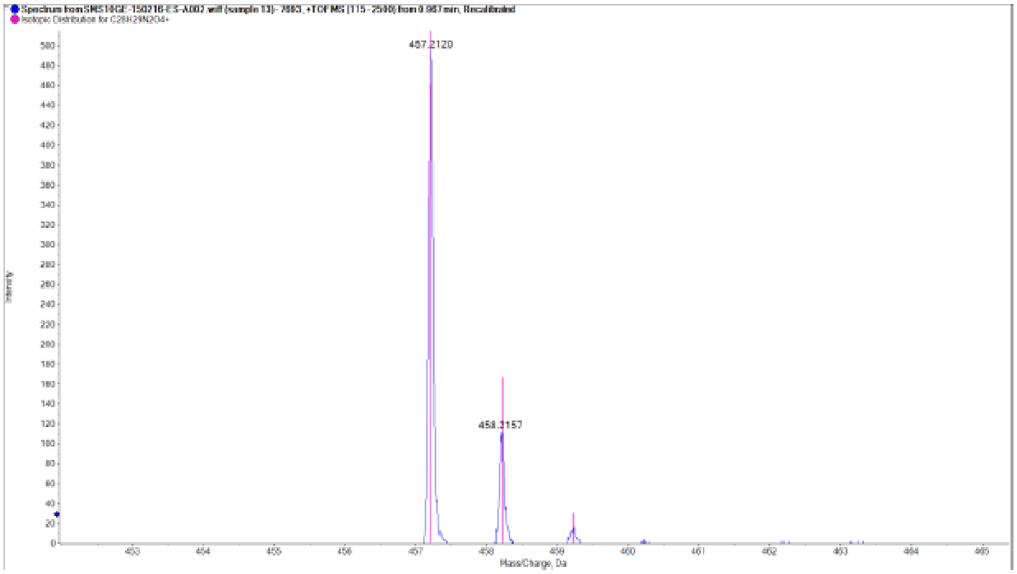

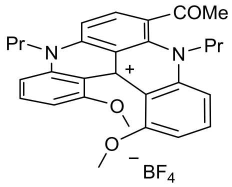

17a

^{1}H NMR (500 MHz, CD_2Cl_2)

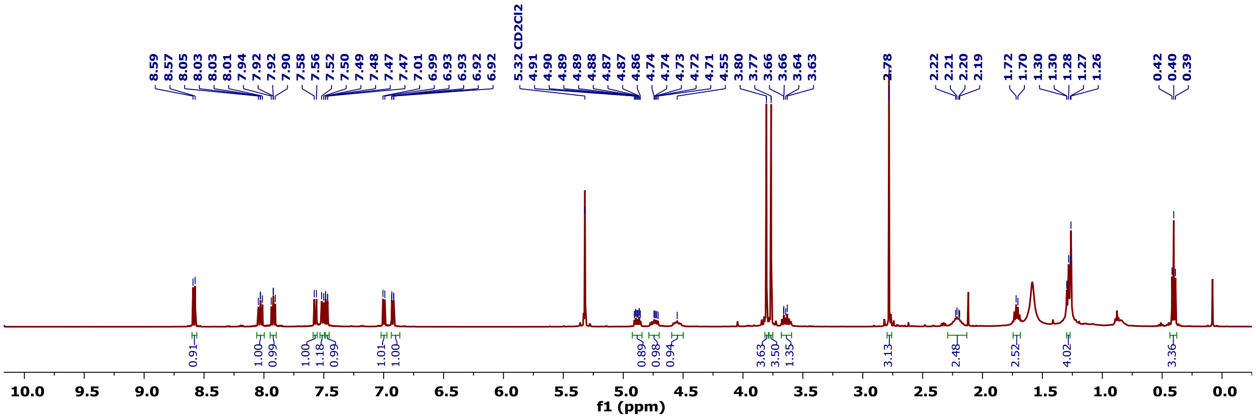

^{13}C NMR (125 MHz, CD_2Cl_2)

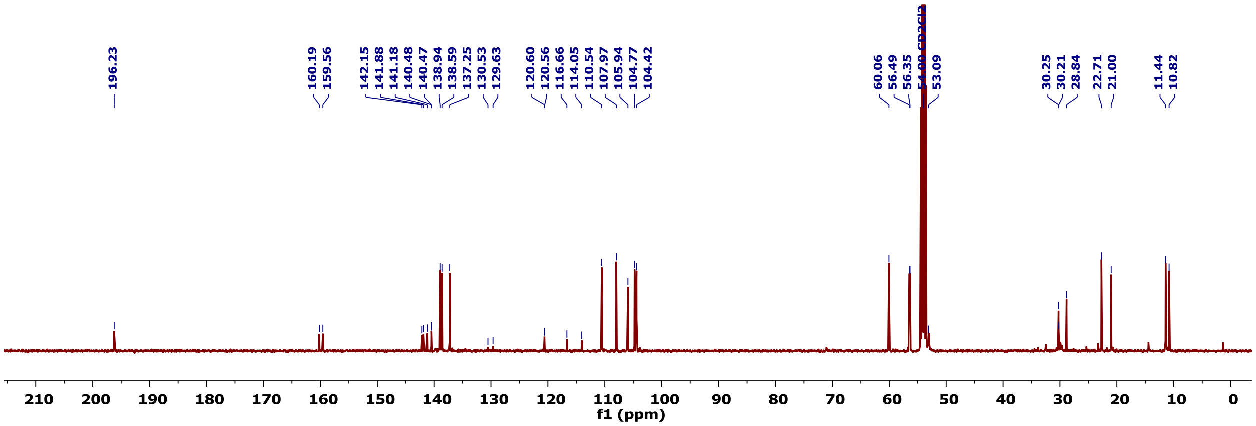

^{19}F NMR (282 MHz, CD_2Cl_2)

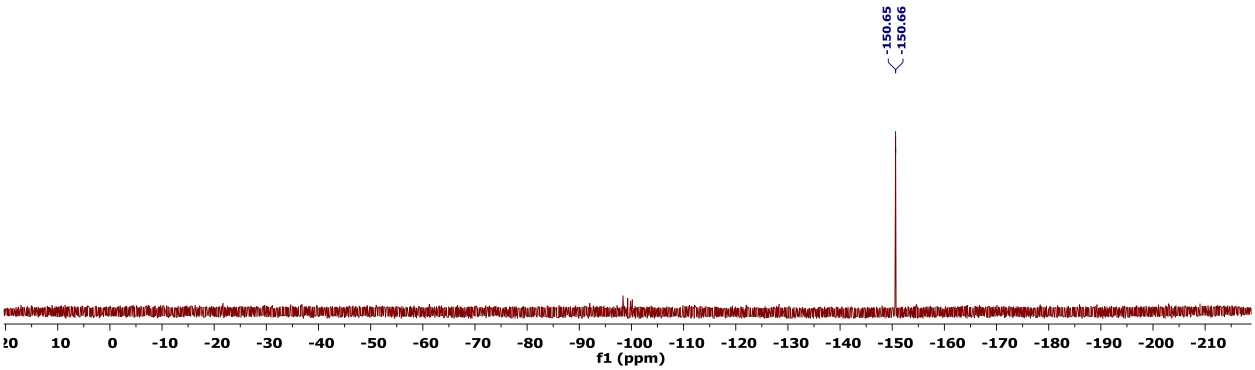

UNIVERSITY OF GENEVA

Faculty of Sciences

Sciences Mass Spectrometry

Submitter:	Romain Duwald	Date of reception:	07/09/15
Sample name:	DuR263	Date of certificate:	21/09/15
Sample number:	8046	Data filename:	SMS10GE-150918-JM-A001
Operator:	Julien Meyer	Instrument:	QSTAR Pulsar (AB/MDS Sciex)
Principal investigator:	Dr. Sophie Michalet	Ionisation mode:	ESI (positive mode)

Expected Formula	Observed *m/z* $[M]^+$	Expected *m/z* (amu)	Accuracy (ppm)
$C_{29}H_{31}O_3N_2$	455.2329	455.2328	-0.2

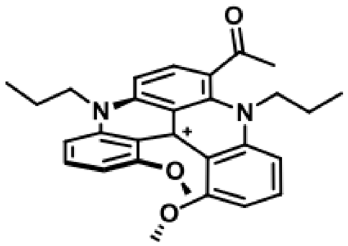

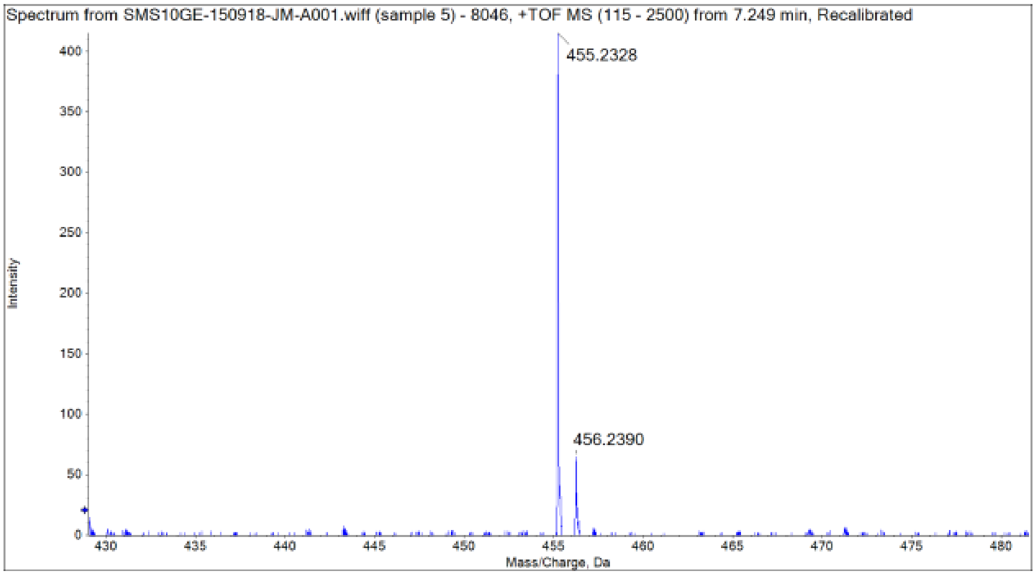

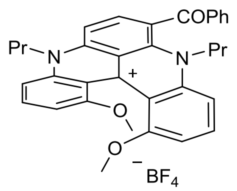

17b

^{1}H NMR (500 MHz, CD_2Cl_2)

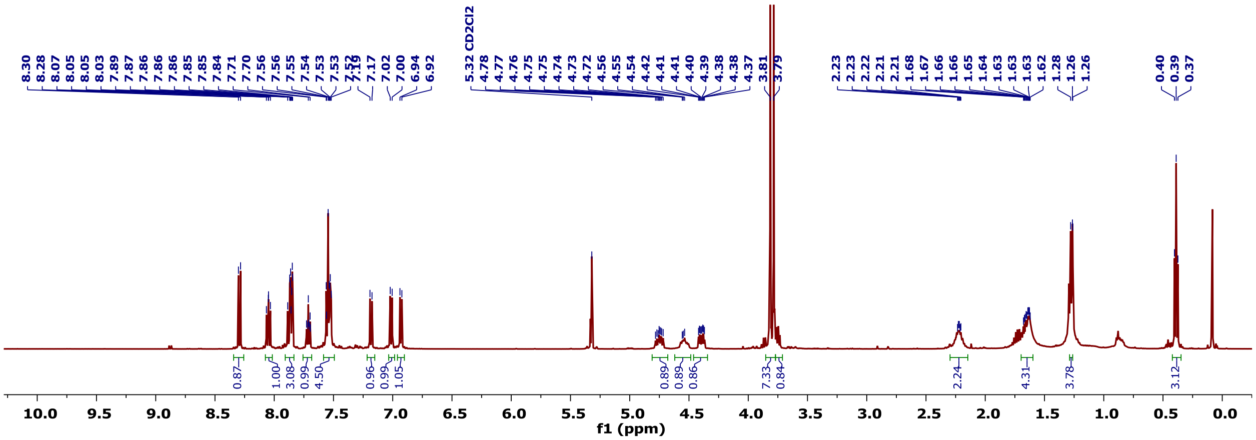

^{13}C NMR (125 MHz, CD_2Cl_2)

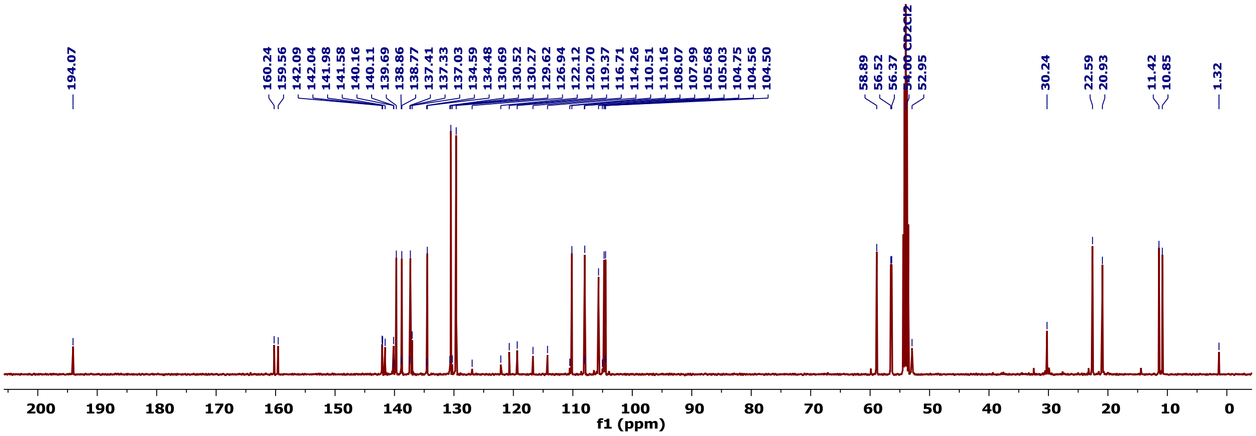

^{19}F NMR (282 MHz, CD_2Cl_2)

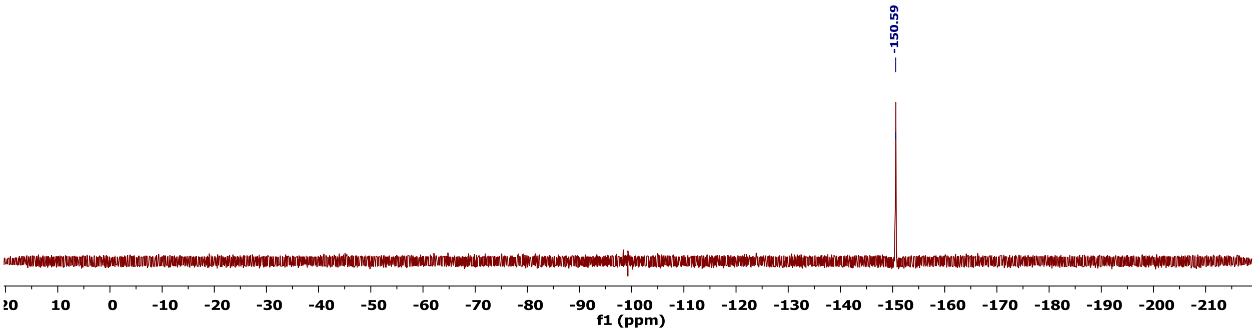

UNIVERSITY OF GENEVA

Faculty of Sciences

Sciences Mass Spectrometry

Submitter: Romain Duwald	Date of reception: 07/09/15
Sample name: DuR260	Date of certificate: 22/09/15
Sample number: 8045	Data filename: SMS10GE-150916-HT-A001
Operator: Harry Théraulaz	Instrument: QSTAR Pulsar (AB/MDS Sciex)
Principal investigator: Dr. Sophie Michalet	Ionisation mode: ESI (positive mode)

Expected Formula	Observed *m/z* $[M]^+$	Expected *m/z* (amu)	Accuracy (ppm)
$C_{34}H_{33}N_2O_3$	517.2501	517.2486	2.9

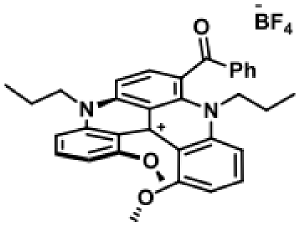

Spectrum from SMS10GE-150916-HT-A001.wiff (sample 4) - 8045, +TOF MS (115 - 2500) from 0.967 to 1.000 min, Recalibrated

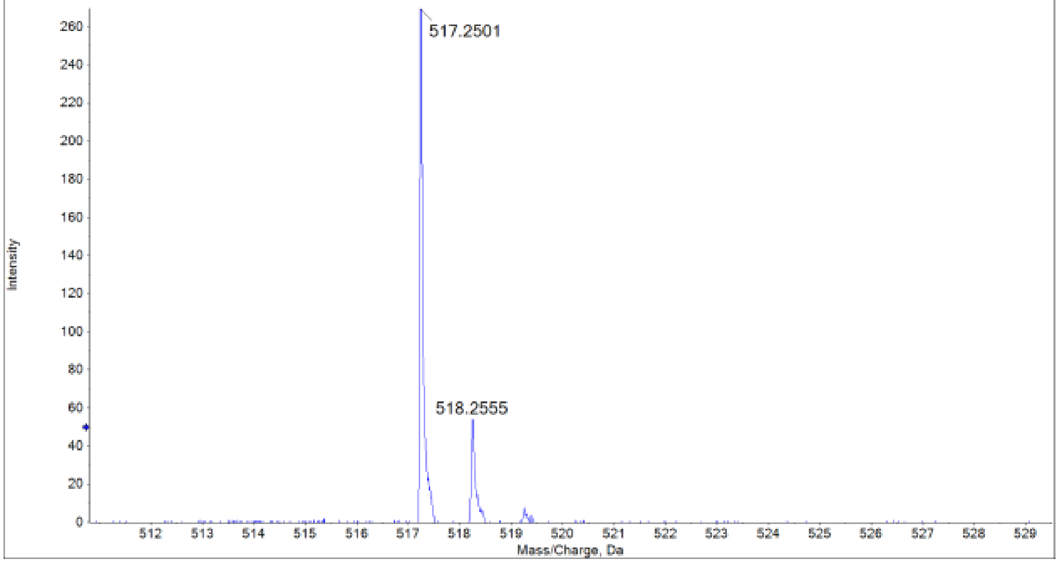

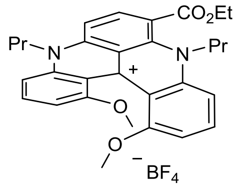

17c

¹H NMR (500 MHz, CD_2Cl_2)

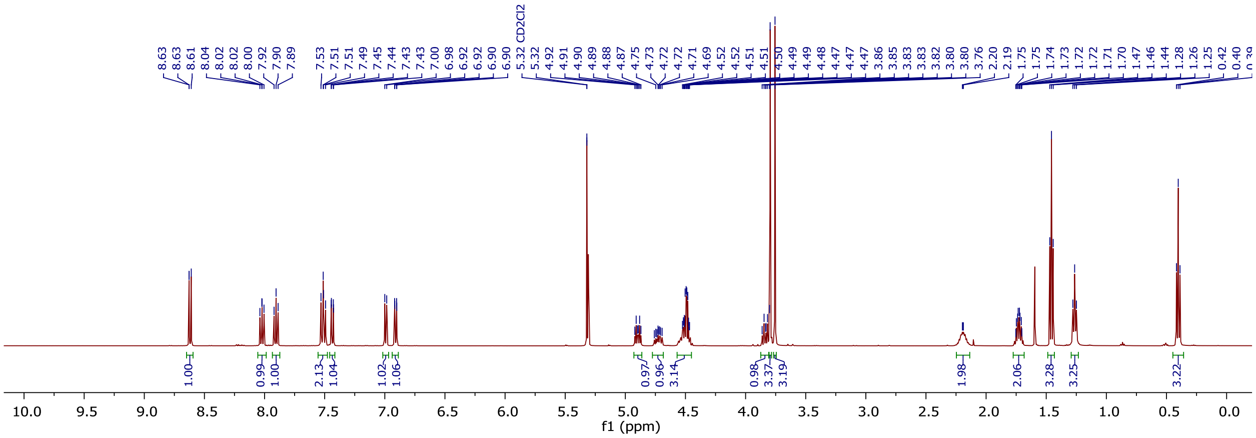

¹³C NMR (125 MHz, CD_2Cl_2)

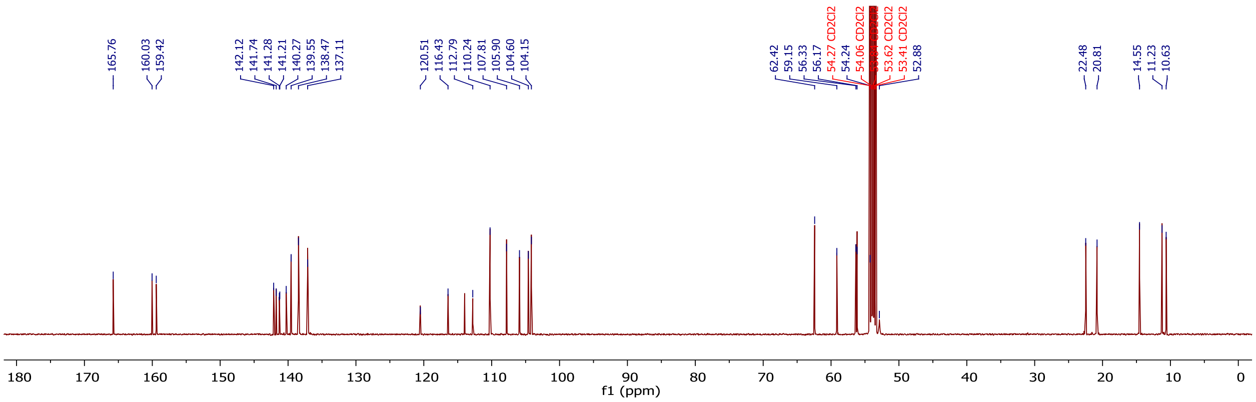

¹⁹F NMR (282 MHz, CD_2Cl_2)

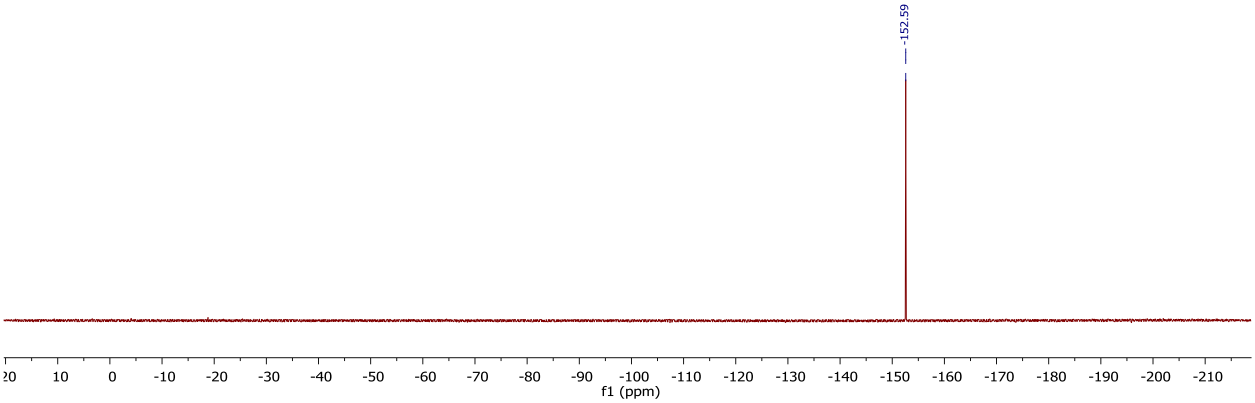

UNIVERSITY OF GENEVA

Faculty of Sciences

Sciences Mass Spectrometry

Submitter: PASCAL	Date of reception: 13/02/15
Sample name: SPA041	Date of certificate: 17/02/15
Sample number: 7692	Data filename: SMS10GE-150216-ES-A002
Operator: Eliane Sandmeier	Instrument: QSTAR Pulsar (AB/MDS Sciex)
Principal investigator: Dr. Sophie Michalet	Ionisation mode: ESI (positive mode)

Expected Formula	Observed *m/z* $[M]^+$	Expected *m/z* (amu)	Accuracy (ppm)
$C_{30}H_{33}N_2O_4$	485.2437	485.2435	0.5

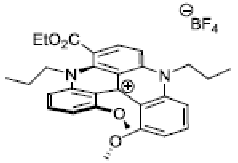

SPA041

Chemical Formula: $C_{30}H_{33}N_2O_4^+$

Exact Mass: 485,2435

Molecular Weight: 485,5935

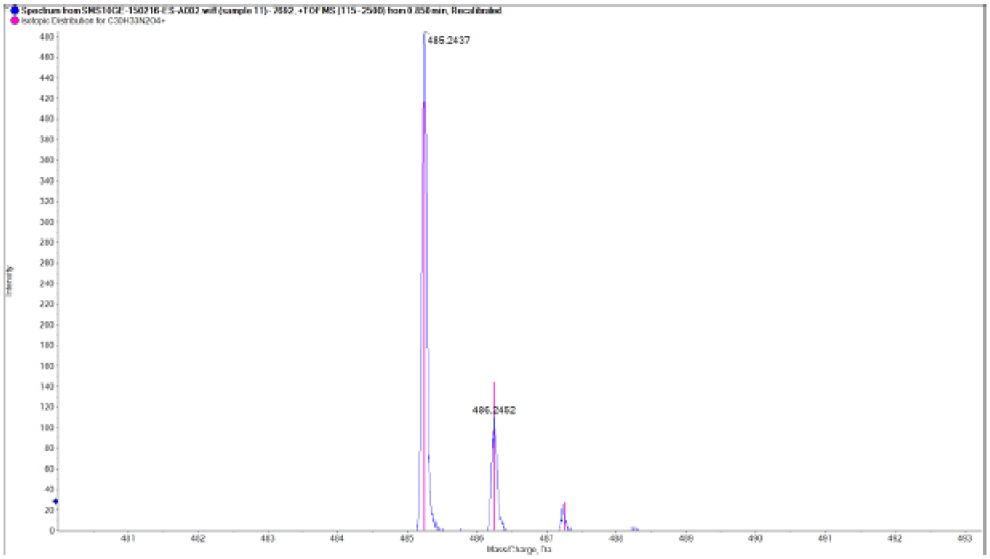

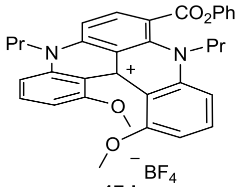

17d

^{1}H NMR (500 MHz, CD_2Cl_2)

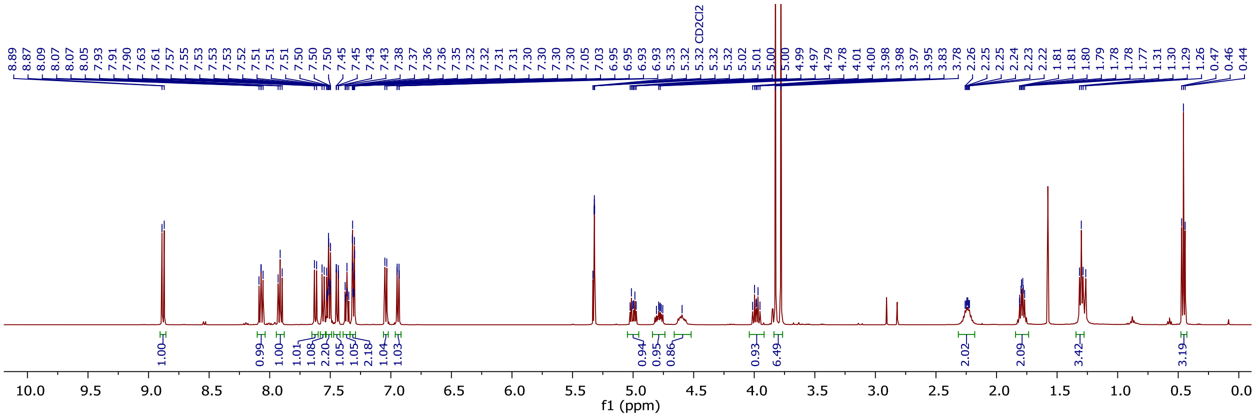

^{13}C NMR (125 MHz, CD_2Cl_2)

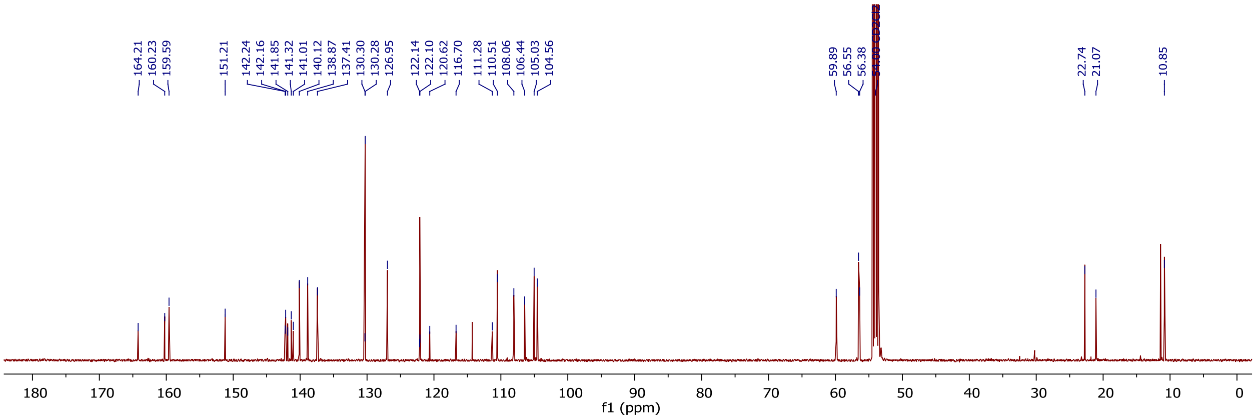

^{19}F NMR (282 MHz, CD_2Cl_2)

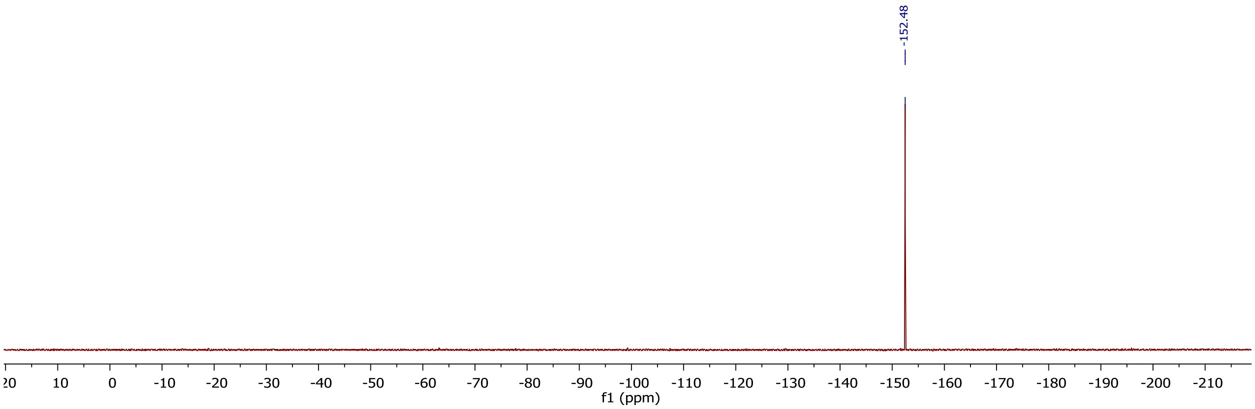

UNIVERSITY OF GENEVA

Faculty of Sciences

Sciences Mass Spectrometry

UNIVERSITÉ DE GENÈVE

SMS MS

Submitter: PASCAL	Date of reception: 24/03/15
Sample name: SPA045	Date of certificate: 08/04/15
Sample number: 7752	Data filename: SMS10GE-150407-ES-A001
Operator: Eliane Sandmeier	Instrument: QSTAR Pulsar (AB/MDS Sciex)
Principal investigator: Dr. Sophie Michalet	Ionisation mode: ESI (positive mode)

Expected Formula	Observed *m/z* $[M]^+$	Expected *m/z* (amu)	Accuracy (ppm)
$C_{34}H_{33}N_2O_4$	533.2436	533.2435	0.2

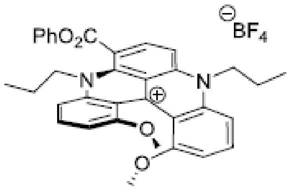

SPA045

Chemical Formula: $C_{34}H_{33}N_2O_4^+$

Exact Mass: 533.2435

Molecular Weight: 533.6363

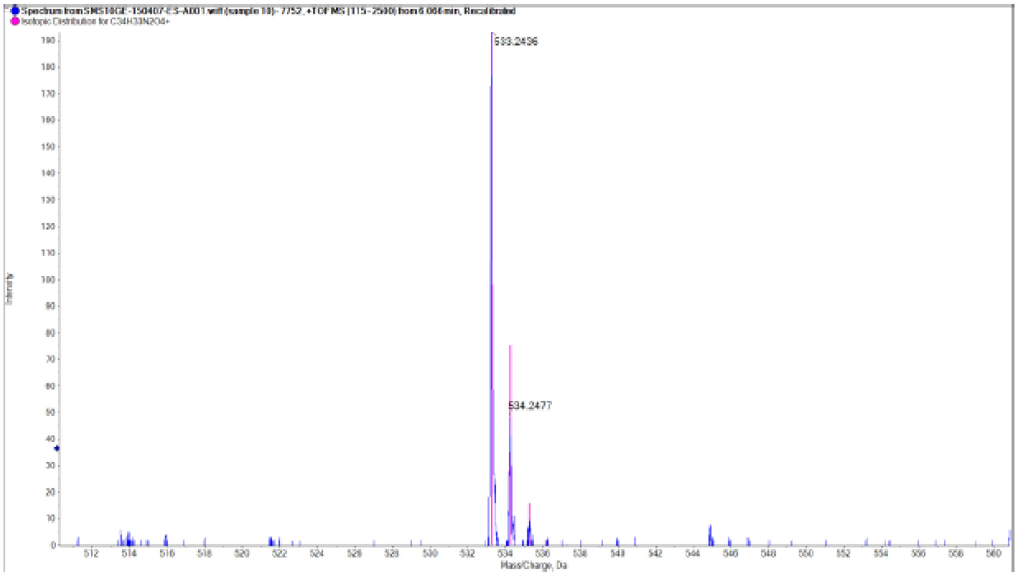

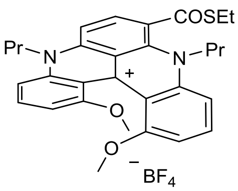

17e

^{1}H NMR (500 MHz, CD_2Cl_2)

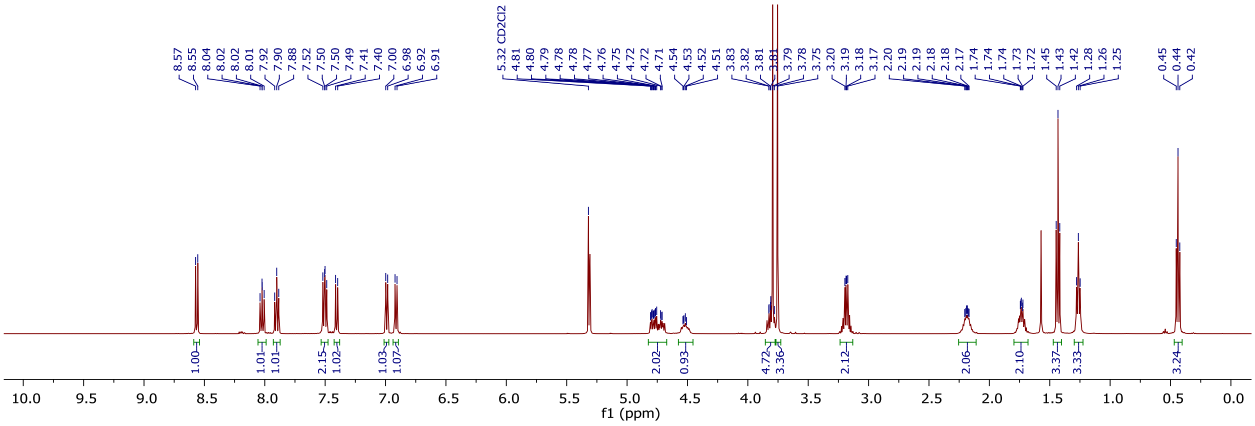

^{13}C NMR (125 MHz, CD_2Cl_2)

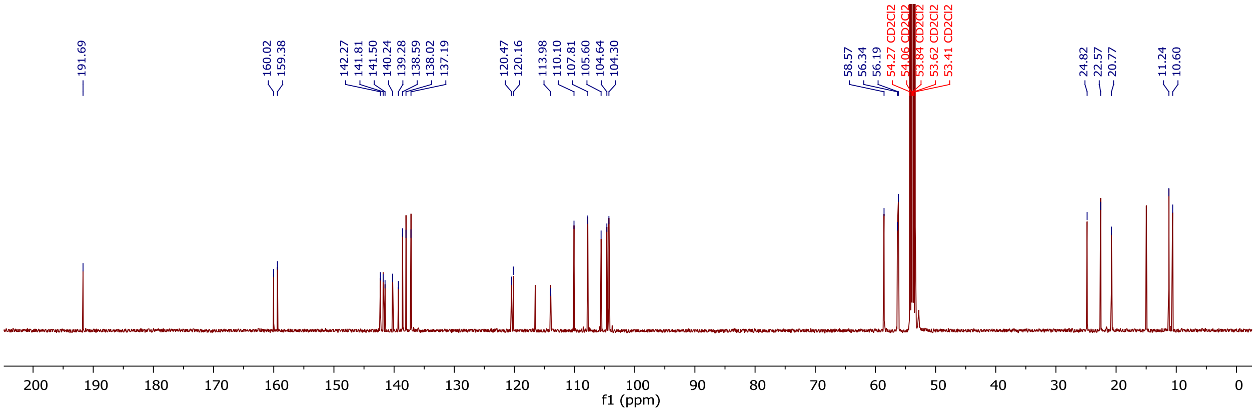

^{19}F NMR (282 MHz, CD_2Cl_2)

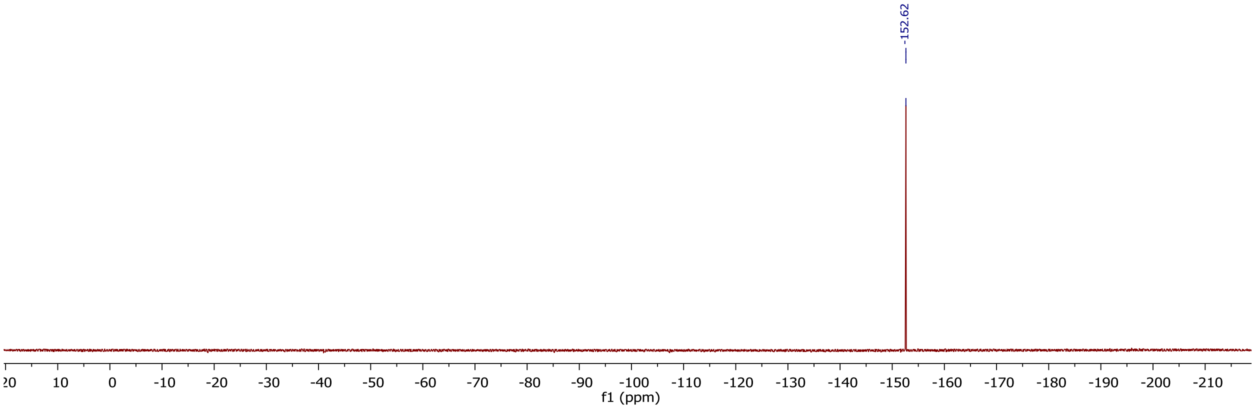

UNIVERSITY OF GENEVA

Faculty of Sciences

Sciences Mass Spectrometry

Submitter: PASCAL	Date of reception: 26/03/15
Sample name: SPA065	Date of certificate: 02/04/15
Sample number: 7759	Data filename: SMS03GE-150401-ES-A002
Operator: Eliane Sandmeier	Instrument: QSTAR Pulsar (AB/MDS Sciex)
Principal investigator: Dr. Sophie Michalet	Ionisation mode: ESI (positive mode)

Expected Formula	Observed *m/z* $[M]^+$	Expected *m/z* (amu)	Accuracy (ppm)
$C_{30}H_{33}N_2O_3S$	501.2217	501.2207	2.2

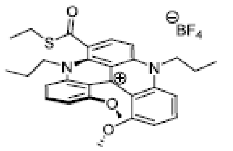

SPA065

Chemical Formula: $C_{30}H_{33}N_2O_3S^+$

Exact Mass: 501.2206

Molecular Weight: 501.6591

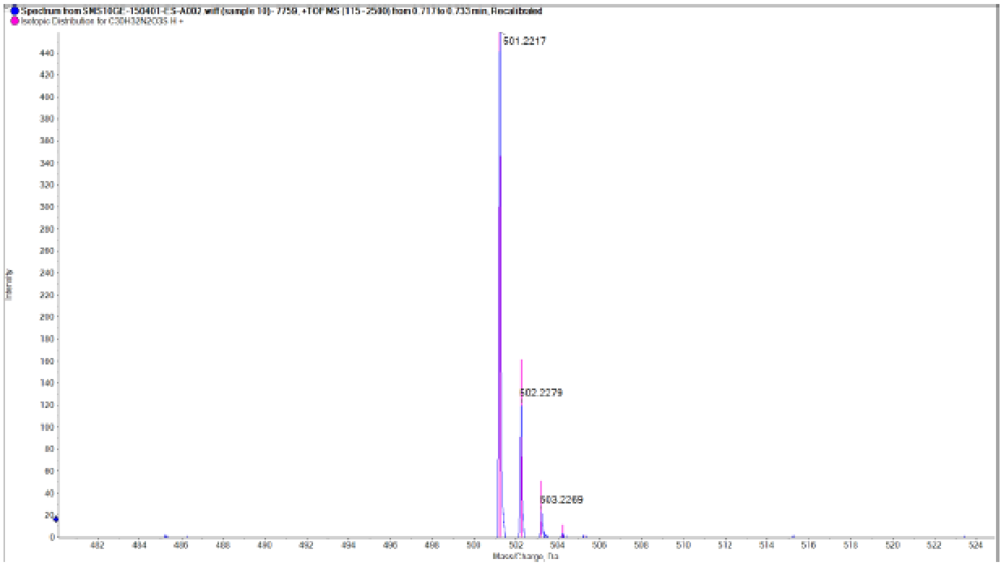

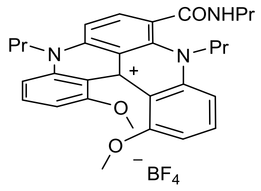

17f

^{1}H NMR (500 MHz, CD_2Cl_2)

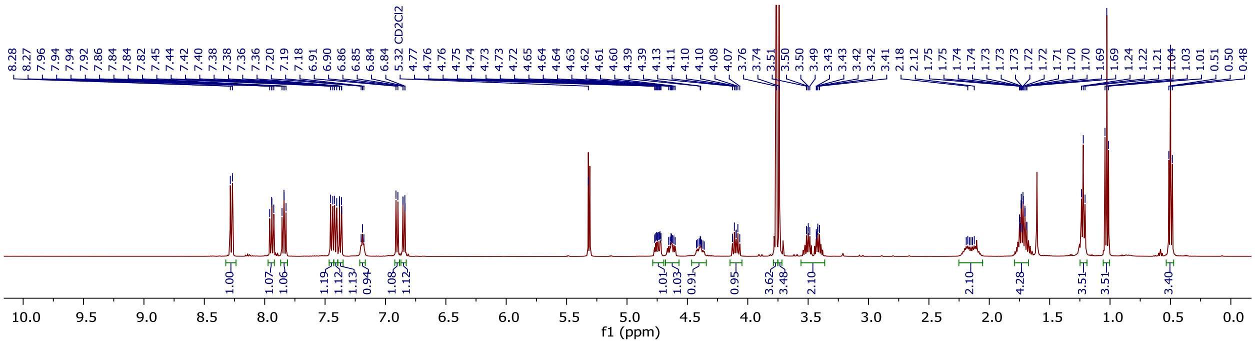

^{13}C NMR (125 MHz, CD_2Cl_2)

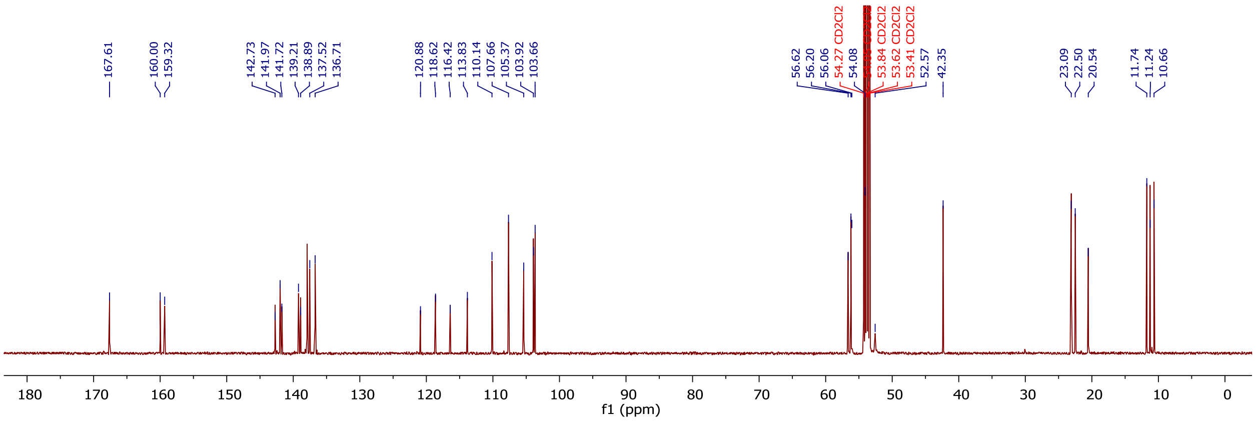

^{19}F NMR (282 MHz, CD_2Cl_2)

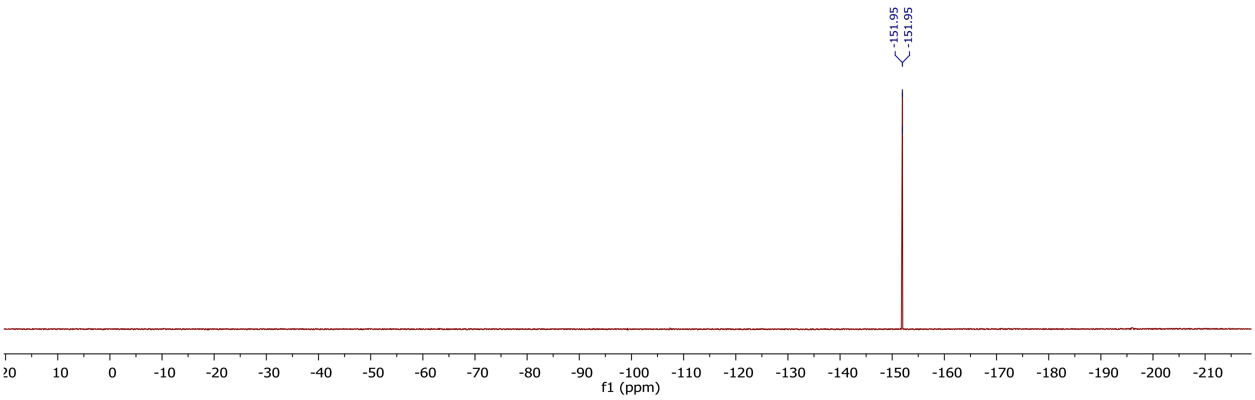

UNIVERSITY OF GENEVA

Faculty of Sciences

Sciences Mass Spectrometry

Submitter: PASCAL	Date of reception: 24/03/15
Sample name: SPA062	Date of certificate: 01/04/15
Sample number: 7755	Data filename: SMS03GE-150331-ES-A001
Operator: Eliane Sandmeier	Instrument: QSTAR Pulsar (AB/MDS Sciex)
Principal investigator: Dr. Sophie Michalet	Ionisation mode: ESI (positive mode)

Expected Formula	Observed *m/z* $[M]^+$	Expected *m/z* (amu)	Accuracy (ppm)
$C_{31}H_{36}N_3O_3$	498.2752	498.2751	0.2

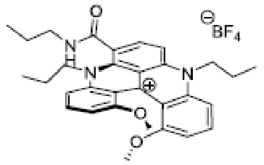

SPA062

Chemical Formula: $C_{31}H_{36}N_3O_3^+$

Exact Mass: 498.2751

Molecular Weight: 498.6353

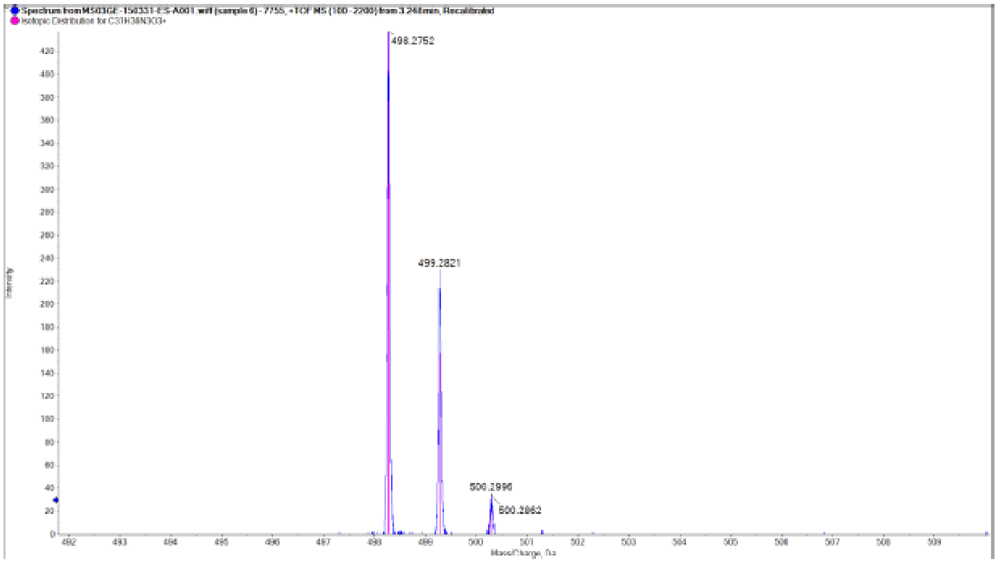

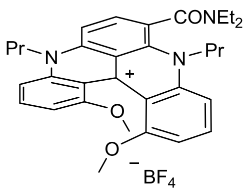

17g

^{1}H NMR (DMSO-d$_6$, 368 K, 400 MHz)

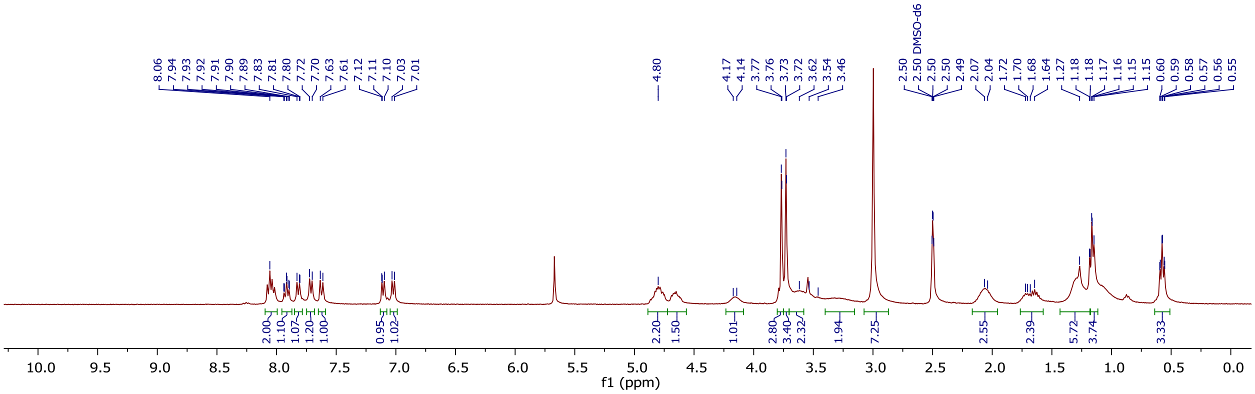

^{13}C NMR (DMSO-d$_6$, 343 K, 125 MHz)

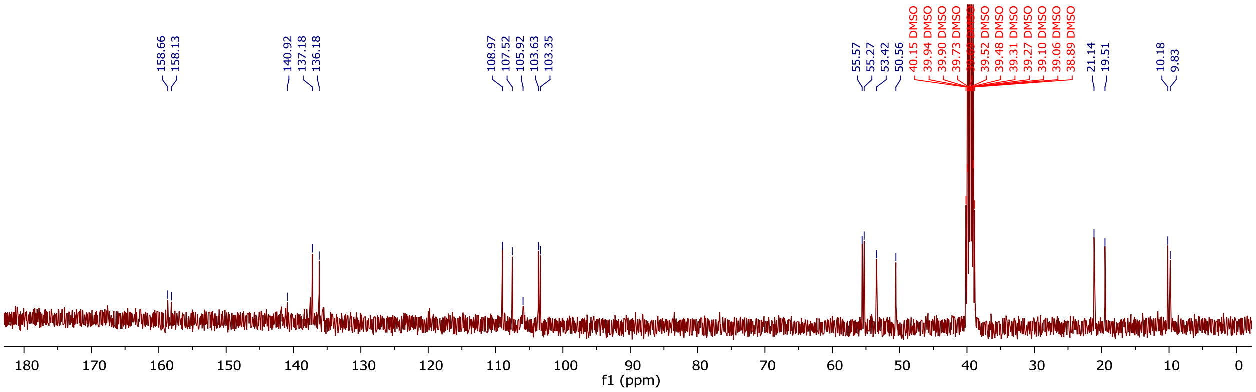

^{19}F NMR (282 MHz, DMSO-d$_6$)

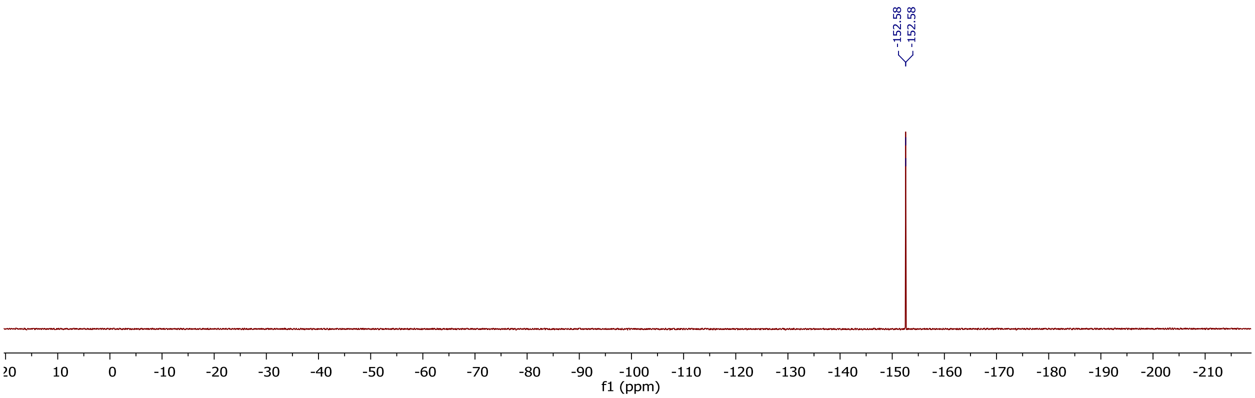

^{1}H NMR (DMSO-d$_6$, variable temperature, 400 MHz)

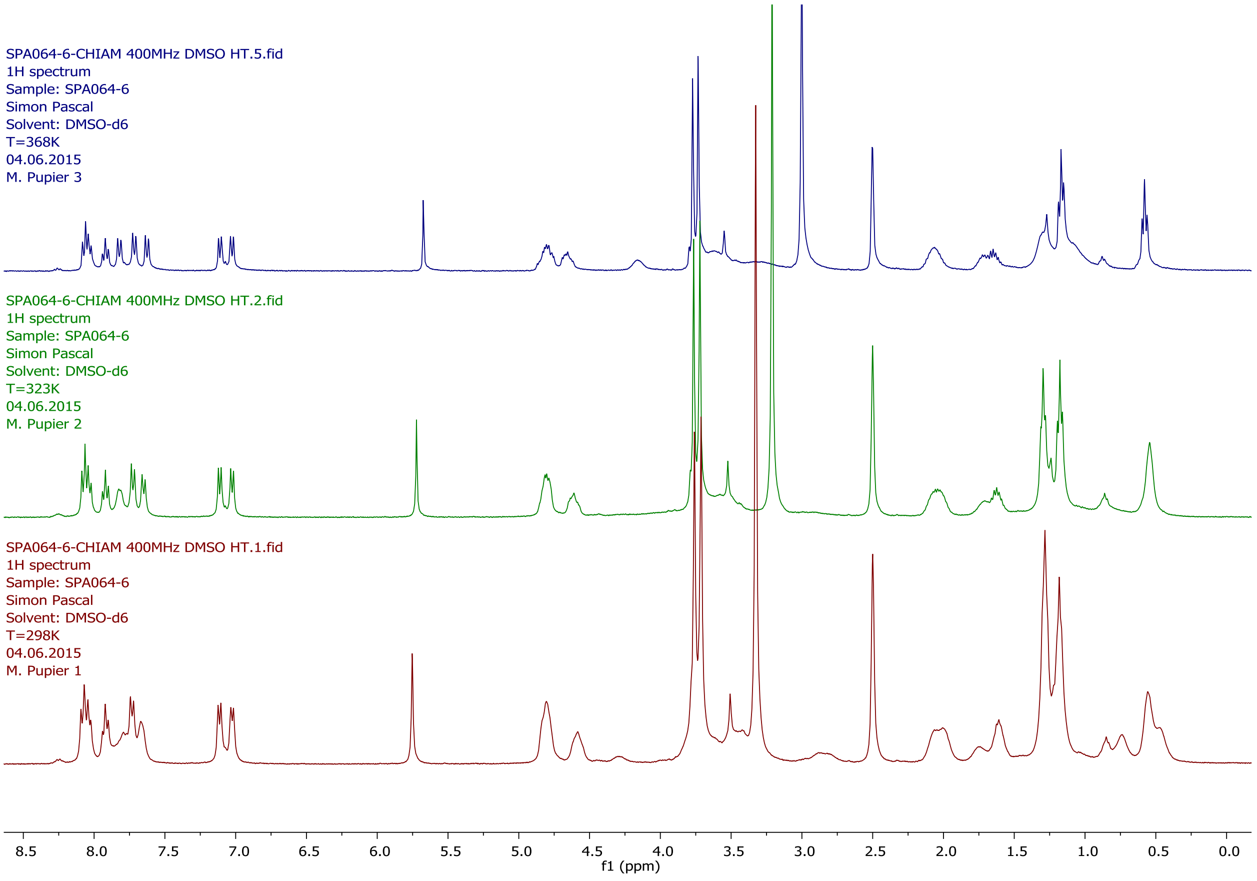

4. X-ray diffraction

Details for the refinement for each structure can be found below with, for each structure, a representation of the cation and of the content of the asymmetric units shown as displacement ellipsoids drawn at 50 percent probability. For disordered structures, comments on the modelling of the disorder are included in this table. More detailed information can be found in the deposited cif files which can be obtained free of charge at the CCDC. For all structure, a view of the packing is included showing that the molecules are forming stacks along the small unit-cell axis. The distances between adjacent molecules is between 3.8 and 4.2 Å. However, adjacent molecules are most of the time shifted, not allowing good π- π overlap.

Table S 1. Relevant crystallographic data.

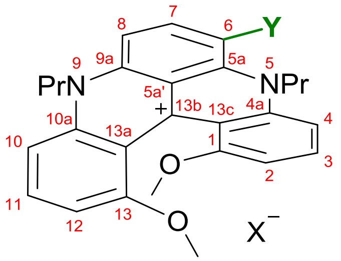

Molecule	Functional group (Y)	Helical pitch / Å	Helical angle / °	$\varphi_{C(13)-C(13a)-C(13b)-C(13c)}$ / °	$\varphi_{C(13a)-C(13b)-C(13c)-C(1)}$ / °
**1**	H	3.19	41.1	27.4	27.4
**5**	NO_2	3.22	41.2	25.8	27.7
**6**	CHO	3.26	45.9	28.2	29.1
**7**	NH_2	3.19	39.8	25.4	27.7
**14**	OH	3.18	39.3	23.2	29.5
**15**	OMe	3.18	39.2	25.2	27.0
**17c**	CO_2Et	3.24	42.0	26.1	27.7
**17e**	COSEt	3.25	42.0	26.8	26.9
**17f**	CONHPr	3.22	42.4	24.8	30.7
**17g**	$CONEt_2$	3.23	43.4	26.9	28.6

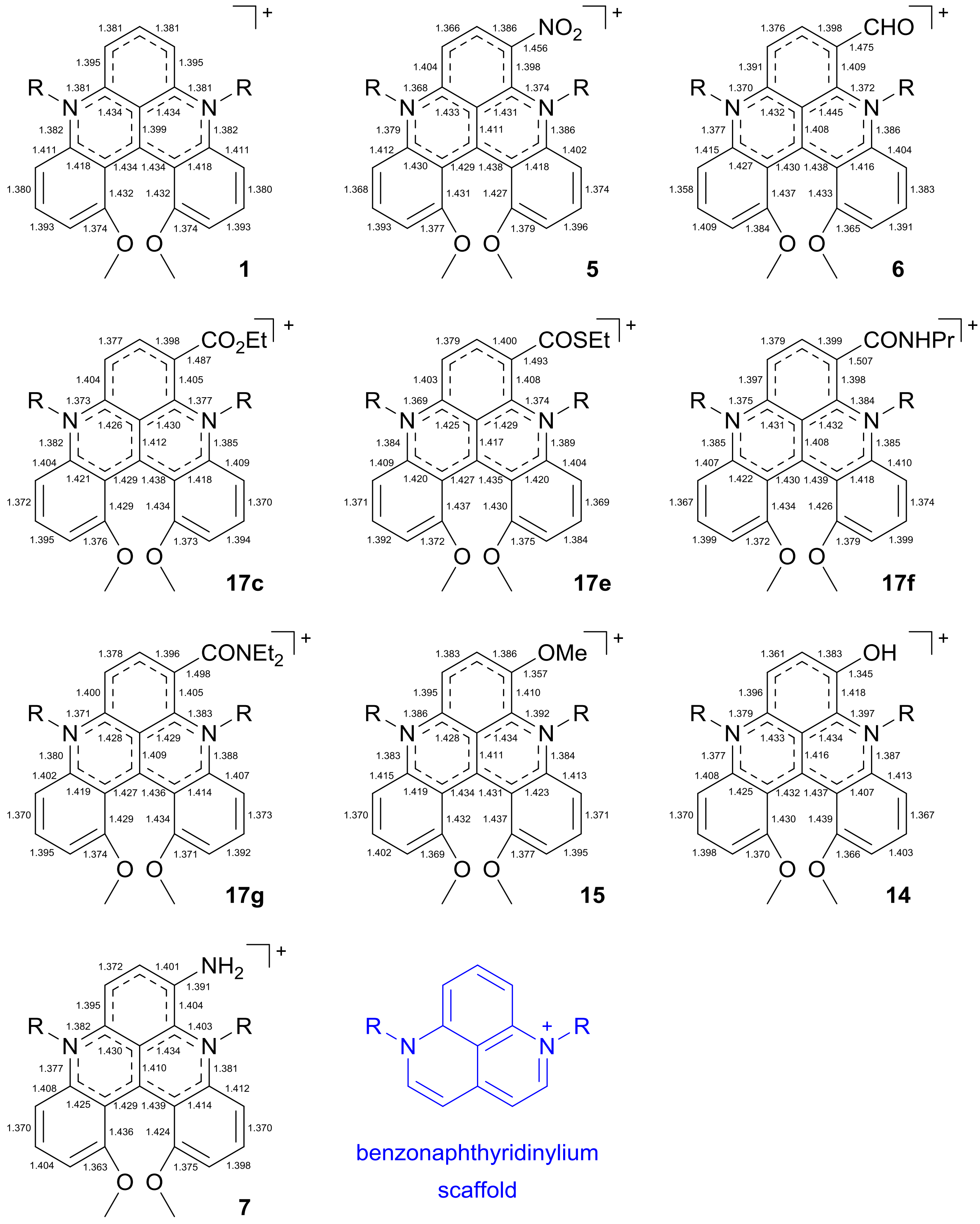

Figure S 1. Bond lengths (Å) extracted from X-ray analysis.

Compound 1 (Y=H)

CCDC	1443641
Formula	$C_{27}H_{29}BF_4N_2O_2$
$D_{calc.}$/ g cm^{-3}	1.342
μ/mm^{-1}	0.878
Formula Weight	500.33
Colour	brown
Shape	prism
Max Size/mm	0.57
Mid Size/mm	0.16
Min Size/mm	0.08
T/K	180(2)
Crystal System	monoclinic
Space Group	I2/a
a/Å	10.0250(5)
b/Å	19.7722(8)
c/Å	12.5456(6)
α/°	90.00
β/°	95.411(4)
γ/°	90.00
V/Å^3	2475.66(19)
Z	4
Z'	0.5
Θ_{min}/°	4.19
Θ_{max}/°	73.63
Measured Refl.	9790
Independent Refl.	2475
Reflections Used	2079
R_{int}	0.0286
Parameters	174
Restraints	0
Largest Peak	0.494
Deepest Hole	-0.267
GooF	1.067
wR_2 (all data)	0.1905
wR_2	0.1794
R_1 (all data)	0.0705
R_1	0.0621

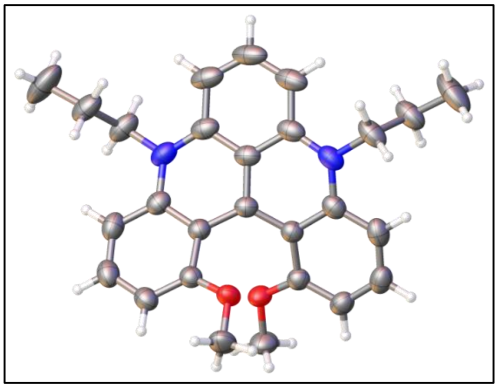

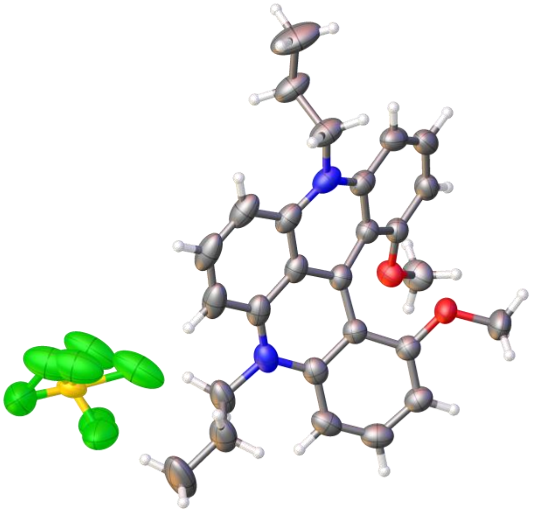

View of the asymmetric unit.(Displacement parameters are drawn at 50 percent probability.)

View along the a axis.

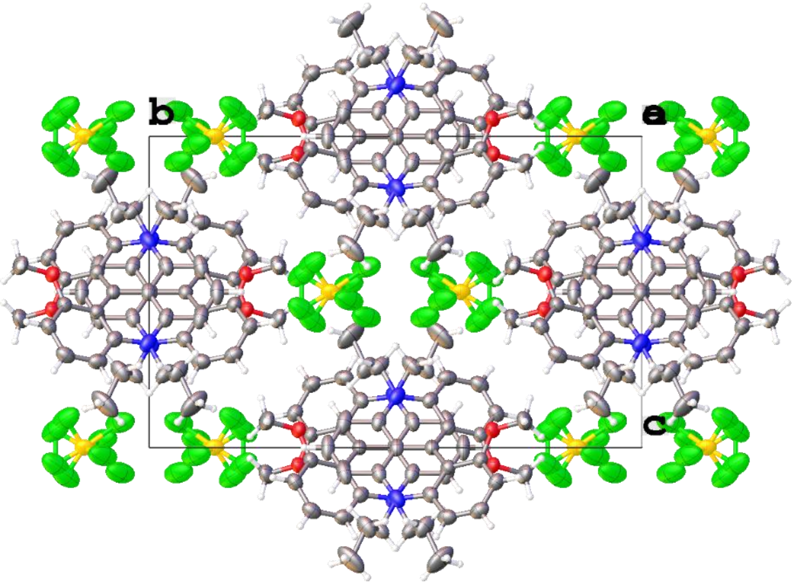

Compound 5 (Y=NO_2)

CCDC	1470066
Formula	$C_{27}H_{29}N_5O_{10}$
$D_{calc.}$/ g cm^{-3}	1.420
μ/mm^{-1}	0.930
Formula Weight	500.33
Colour	orange
Shape	block
Max Size/mm	0.4757
Mid Size/mm	0.3328
Min Size/mm	0.1608
T/K	180(2)
Crystal System	triclinic
Space Group	P-1
a/Å	11.8159(7)
b/Å	14.3381(7)
c/Å	17.9959(9)
$\alpha/^\circ$	78.648(4)
$\beta/^\circ$	84.294(4)
$\gamma/^\circ$	66.016(5)
V/Å^3	2730.4(3)
Z	4
Z'	2
$\Theta_{min}/^\circ$	6.85
$\Theta_{max}/^\circ$	147.69
Measured Refl.	42101
Independent Refl.	10854
Reflections Used	10854
R_{int}	0.0260
Parameters	773
Restraints	2
Largest Peak	0.53
Deepest Hole	-0.31
GooF	1.052
wR_2 (all data)	0.1227
wR_2	0.1164
R_1 (all data)	0.0466
R_1	0.0407

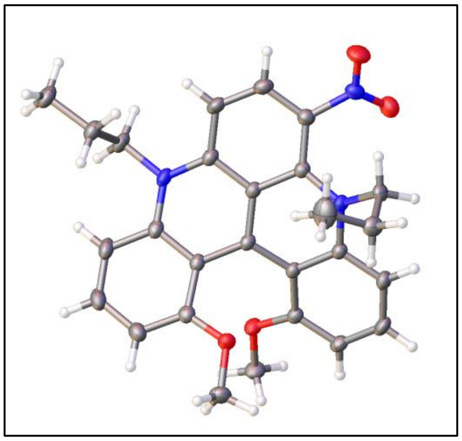

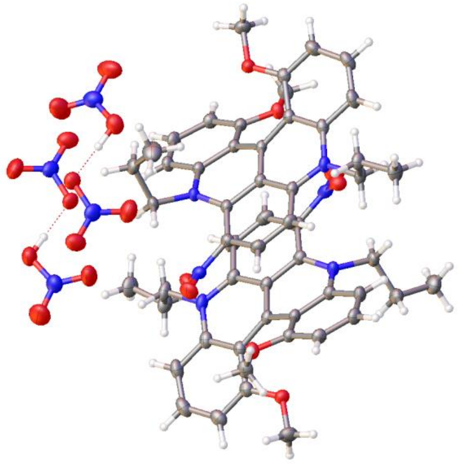

View of the asymmetric unit. (Displacement parameters are drawn at 50 percent probability.)

View along the [-1,1,-1] direction

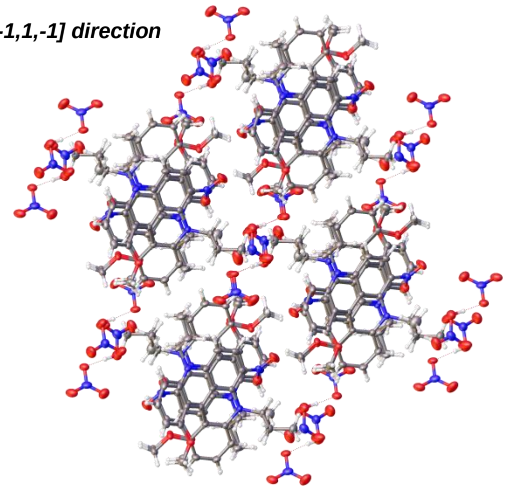

There are two cations in the asymmetric unit that are almost superimposable apart from the positions of the atom C30. H atoms on HNO3 groups were placed using difference Fourier map and geometrical considerations. They were refined using DFIX restraints.

Compound 6 (Y=CHO)

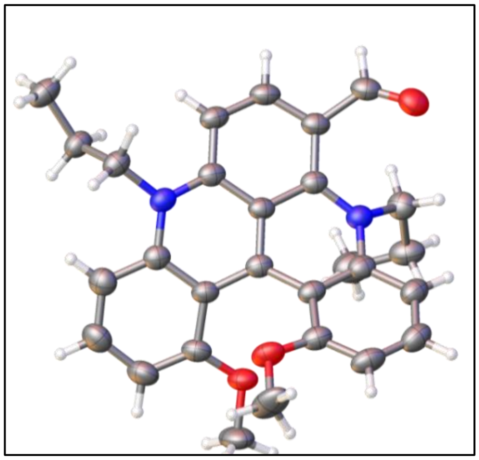

CCDC	1443633
Formula	$C_{28}H_{29}BF_4N_2O_3$
$D_{calc.}$/ g cm^{-3}	1.384
μ/mm^{-1}	0.919
Formula Weight	528.34
Colour	dark red
Shape	plate
Max Size/mm	0.43
Mid Size/mm	0.13
Min Size/mm	0.07
T/K	180.00(14)
Crystal System	orthorhombic
Space Group	Pn2$_1$a
a/Å	22.69881(19)
b/Å	21.08434(17)
c/Å	10.59460(9)
α/°	90
β/°	90
γ/°	90
V/Å^3	5070.46(7)
Z	8
Z'	2
Θ_{min}/°	3.895
Θ_{max}/°	73.413
Measured Refl.	38343
Independent Refl.	9318
Reflections Used	8670
R_{int}	0.0313
Parameters	713
Restraints	84
Largest Peak	0.686
Deepest Hole	-0.403
GooF	1.033
wR_2 (all data)	0.1533
wR_2	0.1480
R_1 (all data)	0.0546
R_1	0.0510

View of the asymmetric unit.
(Displacement parameters are drawn at 50 percent probability)

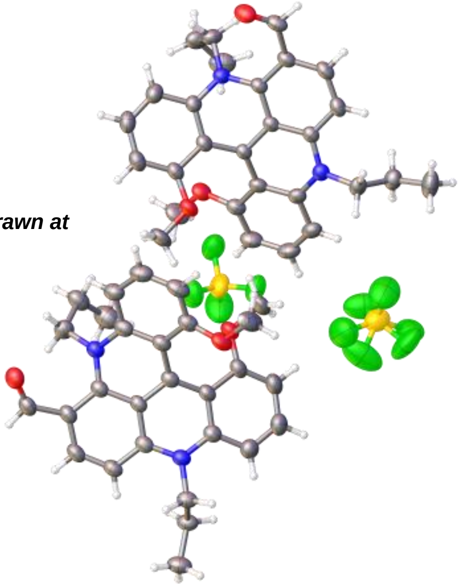

View along the a axis.

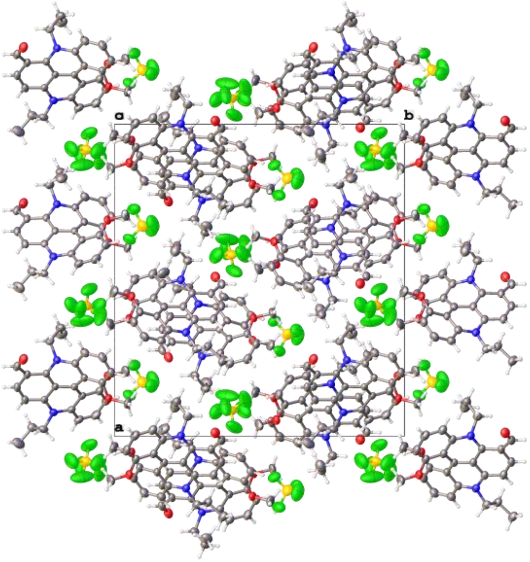

Compound 7 (Y=NH_2)

CCDC	1443632
Formula	$C_{27}H_{30}BF_4N_3O_2$
$D_{calc.}$/ g cm^{-3}	1.412
μ/mm^{-1}	0.926
Formula Weight	515.35
Colour	green
Shape	plate
Max Size/mm	0.12
Mid Size/mm	0.11
Min Size/mm	0.01
T/K	180.00(14)
Crystal System	triclinic
Space Group	P-1
a/Å	7.8922(4)
b/Å	12.0779(7)
c/Å	13.8862(7)
α/°	67.020(5)
β/°	84.303(4)
γ/°	85.759(4)
V/Å^3	1211.69(12)
Z	2
Z'	1
Θ_{min}/°	3.467
Θ_{max}/°	73.395
Measured Refl.	15168
Independent Refl.	4747
Reflections Used	3681
R_{int}	0.0305
Parameters	346
Restraints	0
Largest Peak	0.226
Deepest Hole	-0.258
GooF	0.960
wR_2 (all data)	0.0977
wR_2	0.0904
R_1 (all data)	0.0566

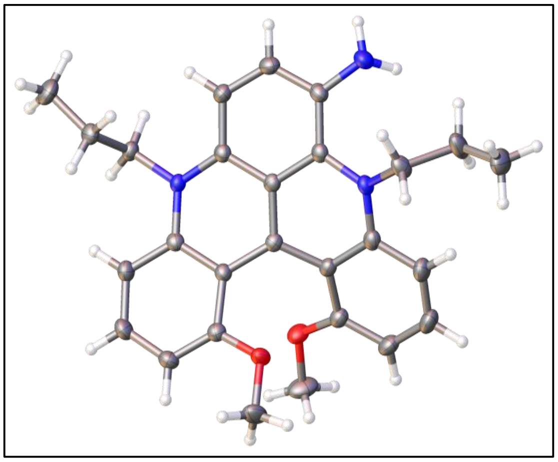

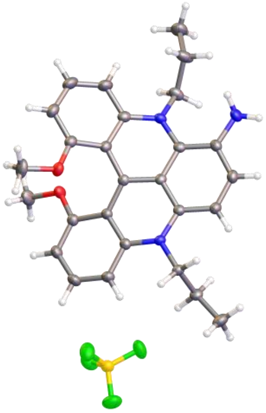

View of the asymmetric unit. Displacement parameters are drawn at 50 percent probability.

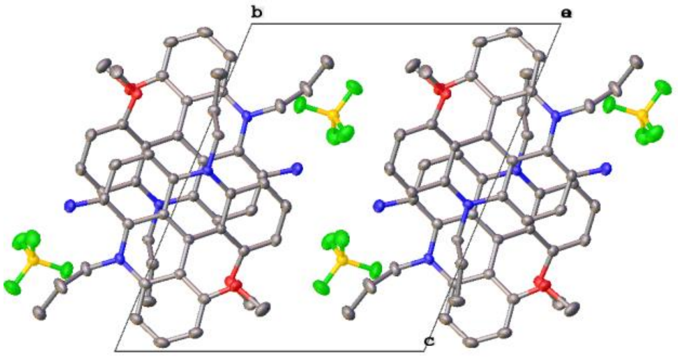

Packing of the molecules : The molecules forms stacks along the b axis.

Compound 14 (Y=OH)

CCDC	1443635
Formula	$C_{27}H_{29}ClN_2O_3$
$D_{calc.}$/ g cm^{-3}	1.382
μ/mm^{-1}	1.780
Formula Weight	464.97
Colour	colourless
Shape	needle
Max Size/mm	0.34
Mid Size/mm	0.05
Min Size/mm	0.03
T/K	180.00(14)
Crystal System	monoclinic
Space Group	$P2_1/n$
a/Å	7.9972(4)
b/Å	20.6791(7)
c/Å	13.5143(4)
$\alpha/^\circ$	90
$\beta/^\circ$	90.901(3)
$\gamma/^\circ$	90
V/Å^3	2234.66(15)
Z	4
Z'	1
$\Theta_{min}/^\circ$	3.908
$\Theta_{max}/^\circ$	73.599
Measured Refl.	16344
Independent Refl.	4437
Reflections Used	3331
R_{int}	0.0509
Parameters	305
Restraints	1
Largest Peak	0.428
Deepest Hole	-0.410
GooF	1.046
wR_2 (all data)	0.1636
wR_2	0.1508
R_1 (all data)	0.0733
R_1	0.0545

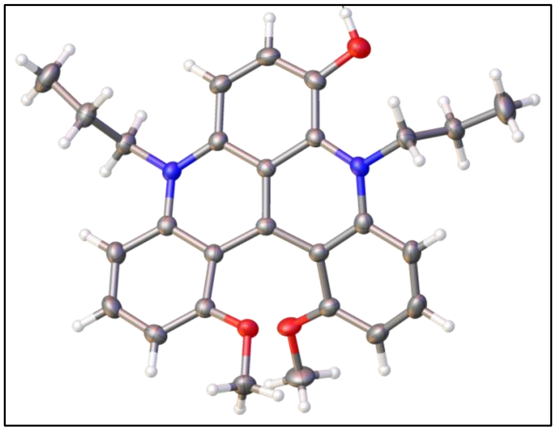

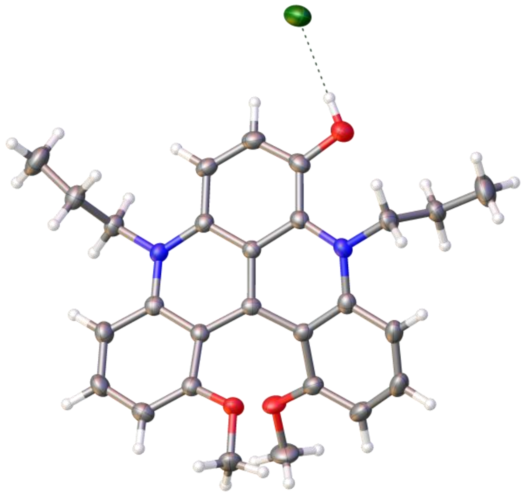

View of the asymmetric unit. Displacement parameters are drawn at 50 percent probability

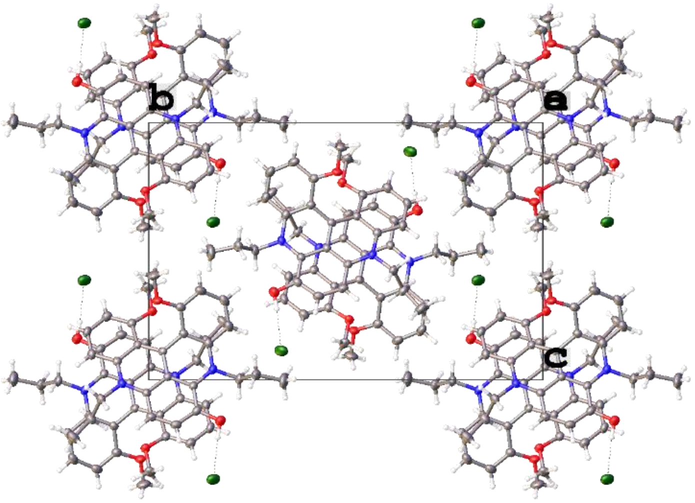

View along the a axis.

Compound 15 (Y=OMe)

CCDC	1443640
Formula	$C_{28}H_{31}BF_4N_2O_3$
$D_{calc.}$/ g cm^{-3}	1.392
μ/mm^{-1}	0.922
Formula Weight	530.36
Colour	red
Shape	plate
Max Size/mm	0.52
Mid Size/mm	0.15
Min Size/mm	0.03
T/K	180.00(14)
Crystal System	triclinic
Space Group	P-1
a/Å	7.8446(4)
b/Å	12.7594(7)
c/Å	13.7549(8)
$\alpha/^{\circ}$	69.935(5)
$\beta/^{\circ}$	78.524(5)
$\gamma/^{\circ}$	82.802(5)
V/Å^3	1264.90(13)
Z	2
Z'	1
$\Theta_{min}/^{\circ}$	3.470
$\Theta_{max}/^{\circ}$	73.474
Measured Refl.	19658
Independent Refl.	5013
Reflections Used	3876
R_{int}	0.0527
Parameters	348
Restraints	0
Largest Peak	0.296
Deepest Hole	-0.276
GooF	1.026
wR_2 (all data)	0.1349
wR_2	0.1223
R_1 (all data)	0.0615
R_1	0.0468

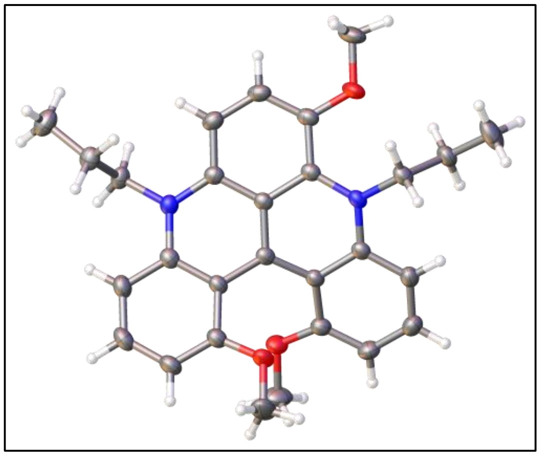

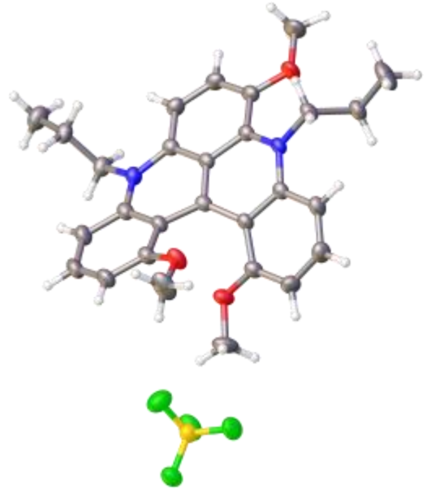

View of the asymmetric unit. Displacement parameters are drawn at 50 percent probability.

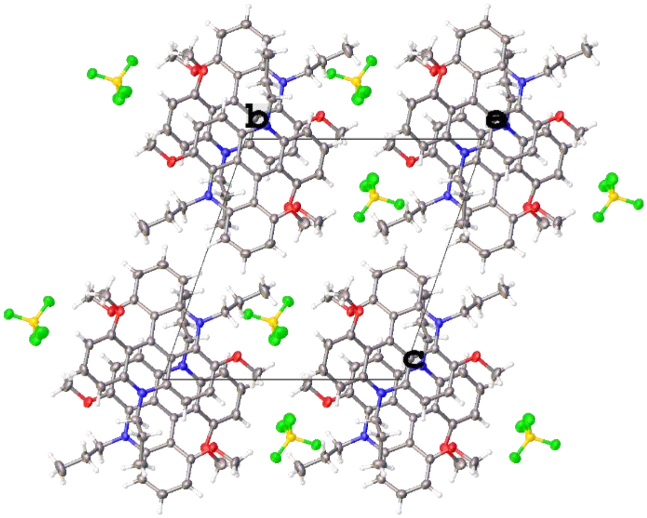

View along the a axis.

Compound 17c (Y=CO_2Et)

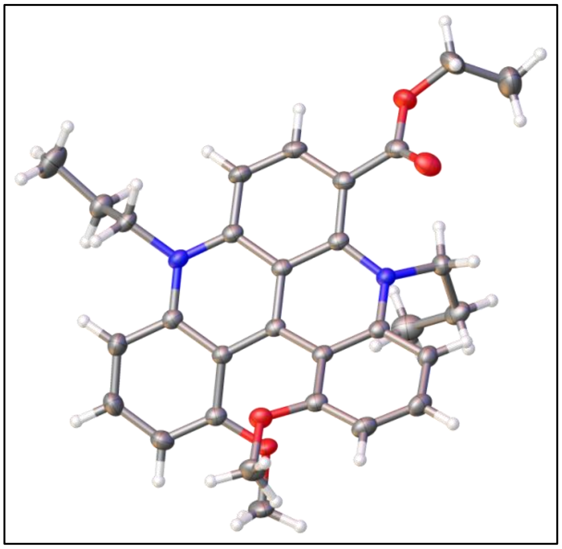

CCDC	1443636
Formula	$C_{31}H_{35}BCl_2F_4N_2O_4$
$D_{calc.}$/ g cm^{-3}	1.391
μ/mm^{-1}	2.407
Formula Weight	657.32
Colour	red
Shape	plate
Max Size/mm	0.30
Mid Size/mm	0.19
Min Size/mm	0.04
T/K	180.00(14)
Crystal System	triclinic
Space Group	P-1
a/Å	10.9867(6)
b/Å	12.0208(6)
c/Å	13.2458(8)
α/°	82.687(5)
β/°	66.161(6)
γ/°	79.273(5)
V/Å^3	1569.51(17)
Z	2
Z'	1
Θ_{min}/°	3.654
Θ_{max}/°	73.622
Measured Refl.	24305
Independent Refl.	6249
Reflections Used	5628
R_{int}	0.0278
Parameters	402
Restraints	0
Largest Peak	0.682
Deepest Hole	-0.627
GooF	1.067
wR_2 (all data)	0.1362
wR_2	0.1320
R_1 (all data)	0.0545

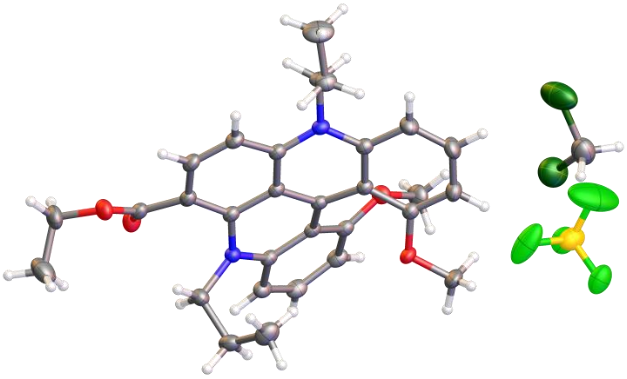

View of the asymmetric unit. Displacement parameters are drawn at 50 percent probability

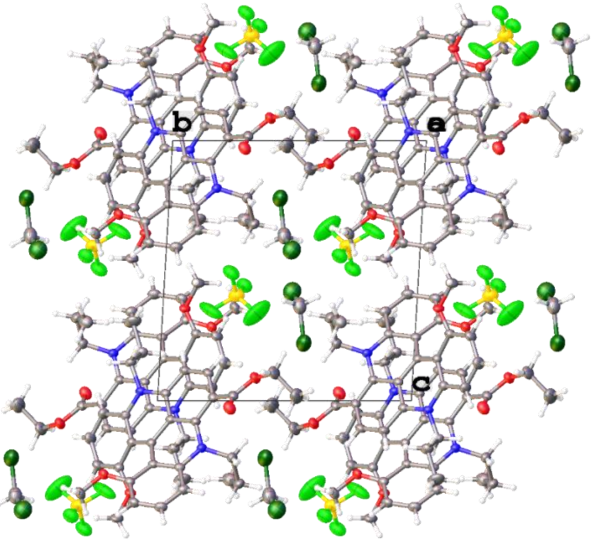

View along the a axis.

Compound 17e (Y=COSEt)

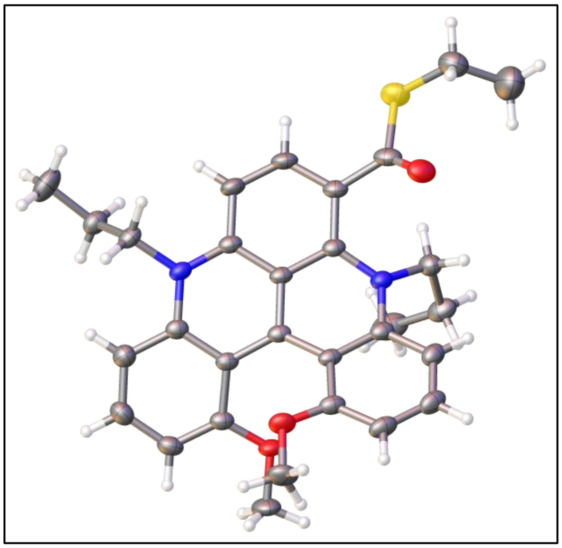

CCDC	1443639
Formula	$C_{31}H_{35}BCl_2F_4N_2O_3S$
$D_{calc.}$/ g cm^{-3}	1.407
μ/mm^{-1}	2.964
Formula Weight	673.38
Colour	red
Shape	plate
Max Size/mm	0.44
Mid Size/mm	0.24
Min Size/mm	0.11
T/K	180(2)
Crystal System	triclinic
Space Group	P-1
a/Å	11.1714(6)
b/Å	11.8849(6)
c/Å	13.3323(7)
α/°	84.422(4)
β/°	65.830(5)
γ/°	79.905(4)
V/Å^3	1589.31(16)
Z	2
Z'	1
Θ_{min}/°	3.635
Θ_{max}/°	73.778
Measured Refl.	24488
Independent Refl.	6301
Reflections Used	5791
R_{int}	0.0233
Parameters	402
Restraints	0
Largest Peak	0.835
Deepest Hole	-1.018
GooF	1.066
wR_2 (all data)	0.1786
wR_2	0.1750
R_1 (all data)	0.0643
R_1	0.0610

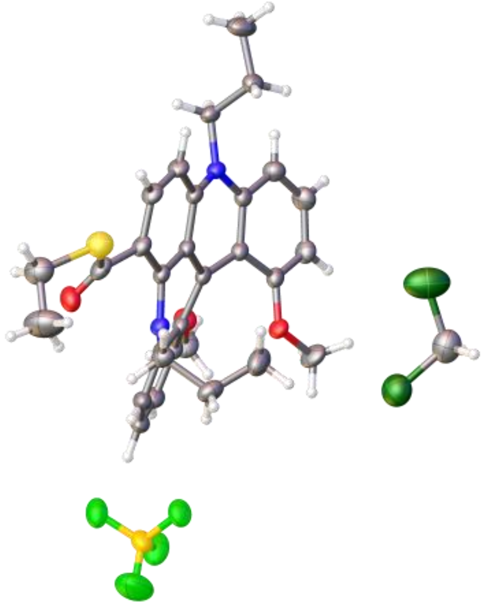

View of the asymmetric unit. Displacement parameters are drawn at 50 percent probability

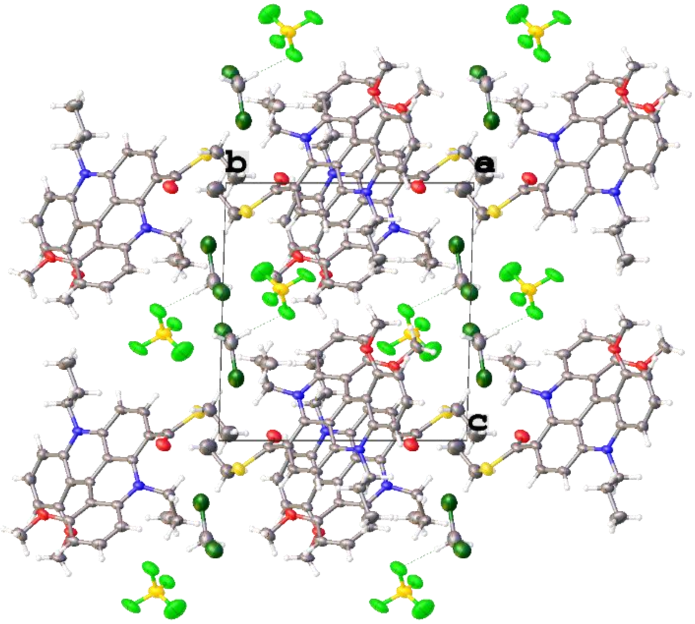

View along the a axis.

Compound 17f (Y=CONHPr)

CCDC	1443637
Formula	$C_{31}H_{36}BF_4N_3O_3$
$D_{calc.}$/ g cm^{-3}	1.340
μ/mm^{-1}	0.865
Formula Weight	585.44
Colour	red
Shape	plate
Max Size/mm	0.31
Mid Size/mm	0.18
Min Size/mm	0.03
T/K	179.95(10)
Crystal System	triclinic
Space Group	P-1
a/Å	8.8012(3)
b/Å	13.0835(4)
c/Å	14.2973(4)
α/°	108.555(3)
β/°	106.512(3)
γ/°	97.631(3)
V/Å^3	1450.56(8)
Z	2
Z'	1
Θ_{min}/°	3.477
Θ_{max}/°	73.803
Measured Refl.	22881
Independent Refl.	5761
Reflections Used	5022
R_{int}	0.0256
Parameters	420
Restraints	127
Largest Peak	0.323
Deepest Hole	-0.419
GooF	1.063
wR_2 (all data)	0.1179
wR_2	0.1129
R_1 (all data)	0.0480
R_1	0.0421

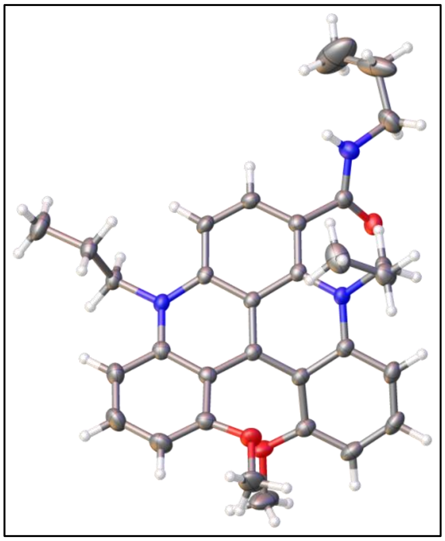

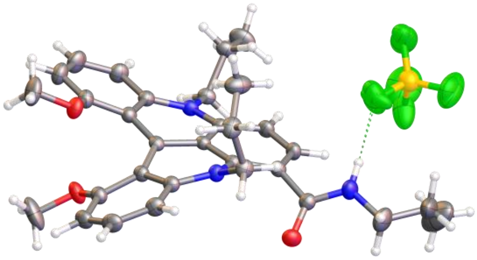

View of the asymmetric unit. Displacement parameters are drawn at 50 percent probability

The BF_4^- anion is disordered and was refined using restraints on 1-2 and 1-3 distances. Anisotropic displacement parameters were also restrained.

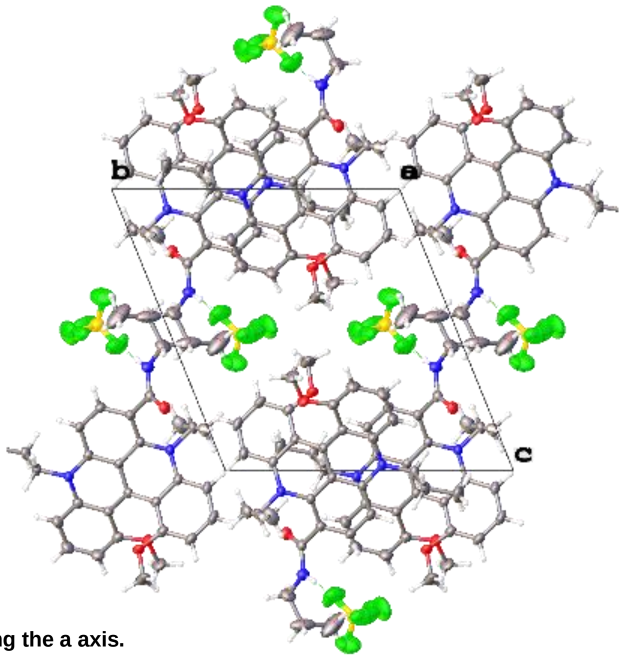

View along the a axis.

Compound 17c (Y=CONEt$_2$)

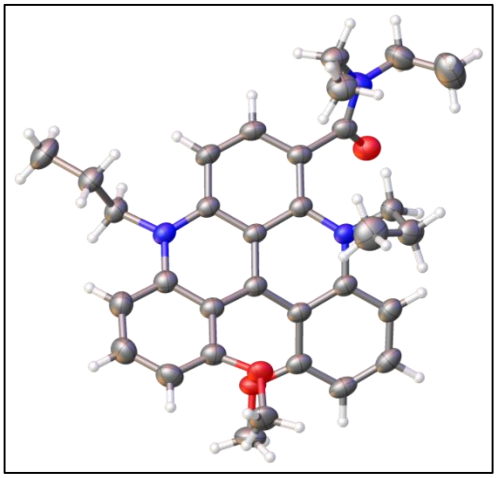

CCDC	1443638
Formula	$C_{35.5}H_{42}BF_4N_3O_3$
$D_{calc.}$/ g cm^{-3}	1.287
μ/mm^{-1}	0.803
Formula Weight	645.53
Colour	red
Shape	plate
Max Size/mm	0.53
Mid Size/mm	0.42
Min Size/mm	0.07
T/K	180.00(14)
Crystal System	triclinic
Space Group	P-1
a/Å	11.0563(3)
b/Å	13.2880(4)
c/Å	14.0408(5)
α/°	115.683(3)
β/°	92.594(3)
γ/°	112.243(3)
V/Å^3	1665.63(11)
Z	2
Z'	1
Θ_{min}/°	4.116
Θ_{max}/°	73.407
Measured Refl.	25669
Independent Refl.	6598
Reflections Used	5743
R_{int}	0.0282
Parameters	531
Restraints	257
Largest Peak	0.512
Deepest Hole	-0.327
GooF	1.049
wR_2 (all data)	0.1748
wR_2	0.1675
R_1 (all data)	0.0631
R_1	0.0574

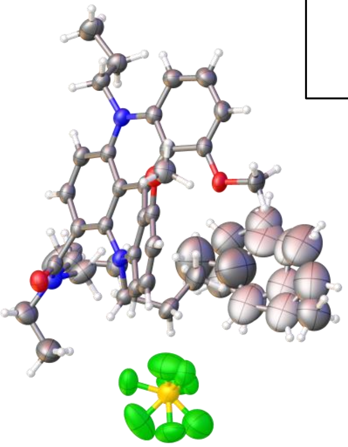

View of the asymmetric unit. Displacement parameters are drawn at 50 percent probability

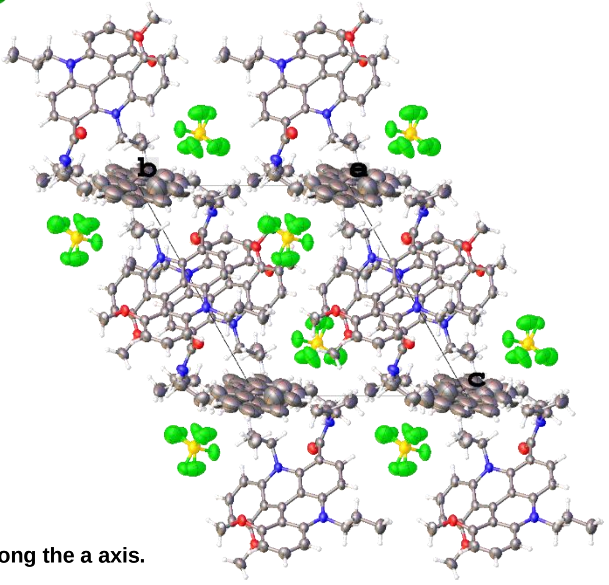

View along the a axis.

The BF_4^- ion is slightly disordered and was refined with 2 components (occupancy 0.8 and 0.2, respectively). SADI restraints were applied on 1,2 and 1,3 distances. RIGU restraints were applied on anisotropic displacement parameters. There is one ethyl chain that is disordered. It was refined using two positions (occupancy 0.567 and 0.433). Restraints were applied on distances and anisotropic displacement parameters. Finally, there is a very disordered toluene molecule which was modelled using two rigid bodies with occupancy 0.25 each. The anisotropic displacement parameters are large but the stoichiometry is well-consistent with the SQUEEZE/BYPASS procedure where the hole at the position of the toluene molecule (0.5 0 0 on the symmetry center) contained 50 electrons. Using the "squeezed" data gave slightly better agreement factor for the refinement but the model with the solvent molecule was preferred.

5. ^{1}H-NOESY experiment of compound 6

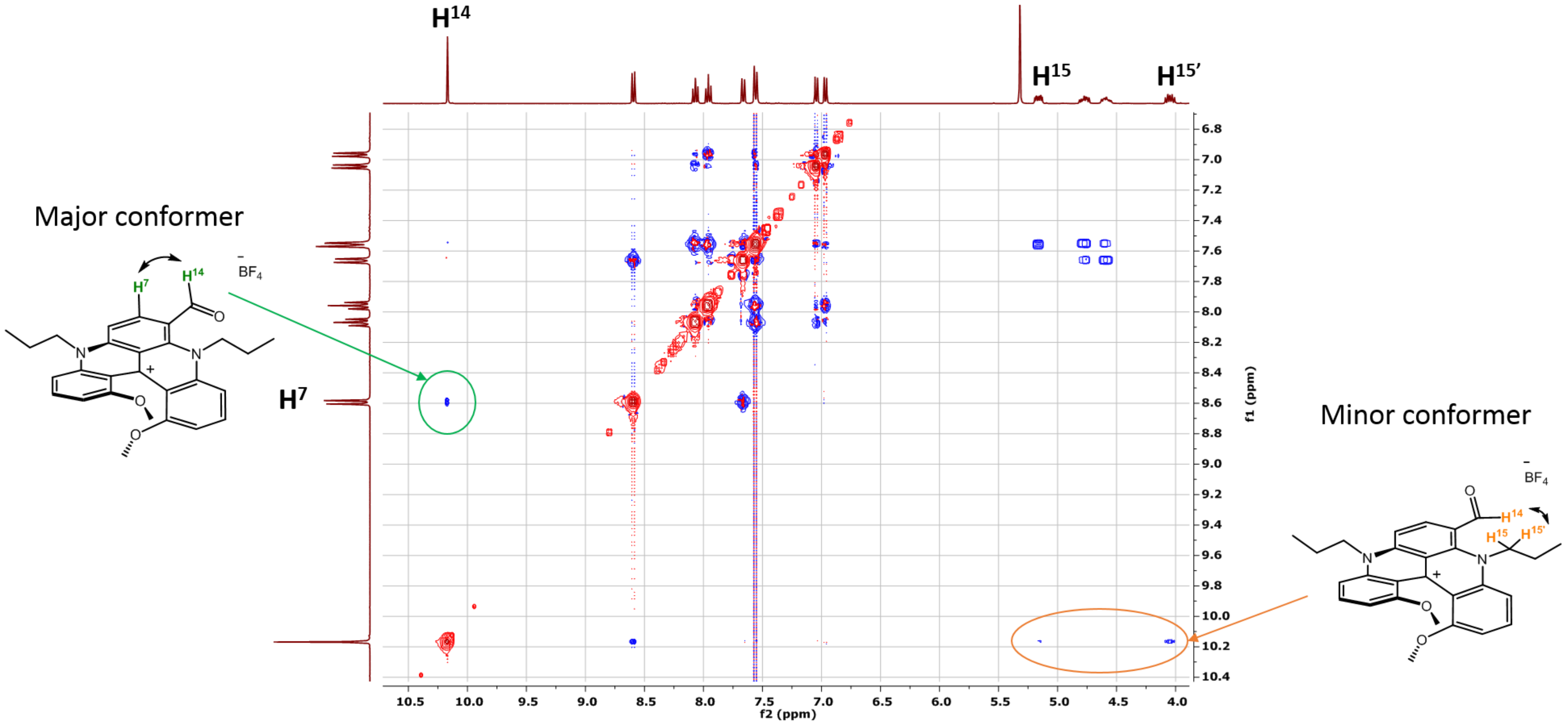

Figure S 2. ^{1}H-NOESY map of compound **6**, CD_2Cl_2, 400 MHz, 298 K. The ^{1}H-NOESY experiment shows two different correlation spots. The major conformer presents H^{14} in front of H^{7} (circled in green on the map) and the minor conformer presents H^{14} in front of H^{15} and $H^{15'}$ (circled in orange on the map

6. Additional cyclic voltammetry data

Table S 2. Anodic and cathodic peak potentials (E_{pa}, E_{pc}) and half-wave potentials ($E_{1/2}$) values (mV) measured by CV for [4]helicenes (10^{-3} M) in acetonitrile ([TBA][PF_6] 10^{-1} M) at a Pt electrode (Ø = 3 mm, v = 0.1 $V.s^{-1}$), E vs Ag/Ag^+. Red_n and Ox_n represent the n successive reduction and oxidation processes, respectively.

Molecule	Functional group (Y)	**Red_3**	**Red_2**			**Red_1**			**Ox_1**			**Ox_2**			**Fc/Fc^+**		
		E_{pc}	E_{pa}	E_{pc}	$E_{1/2}$	E_{pa}	E_{pc}	$E_{1/2}$	E_{pa}	E_{pc}	$E_{1/2}$	E_{pa}	E_{pc}	$E_{1/2}$	E_{pa}	E_{pc}	$E_{1/2}$
**1**	H	-	-	-	-	-1109	-1178	-1144	1002	929	966	-	-	-	114	50	82
**5**	NO_2	-	-1626	-1765	-1696	-854	-922	-888	1417	-	-	-	-	-	113	51	82
**6**	CHO	-1892	-1126	-1243	-1185	-942	-1009	-976	1257	1172	1215	-	-	-	116	51	84
**7**	NH_2	-	-	-	-	-1071	-1148	-1110	487	403	445	915	-	-	134	49	92
**8**	NMe_2	-	-	-	-	-1065	-1136	-1101	463	400	432	819	707	763	129	51	90
**9**	N_3	-	-	-1780	-	-0.972	-1036	-1004	897	820	859	1401	-	-	126	54	90
**15**	OMe	-	-	-	-	-1042	-1107	-1075	764	695	730	1380	-	-	122	57	90
**16**	CO_2^-	-	-	-	-	-1158	-1240	-1199	831	-	-	1137	1047	1092	120	51	86
**17c**	CO_2Et	-	-	-	-	-972	-1041	-1007	1211	1126	1169	-	-	-	118	51	85
**17f**	CONHPr	-	-1727	-1856	-1792	-1001	-1070	-1036	1127	1042	1085	-	-	-	118	51	85

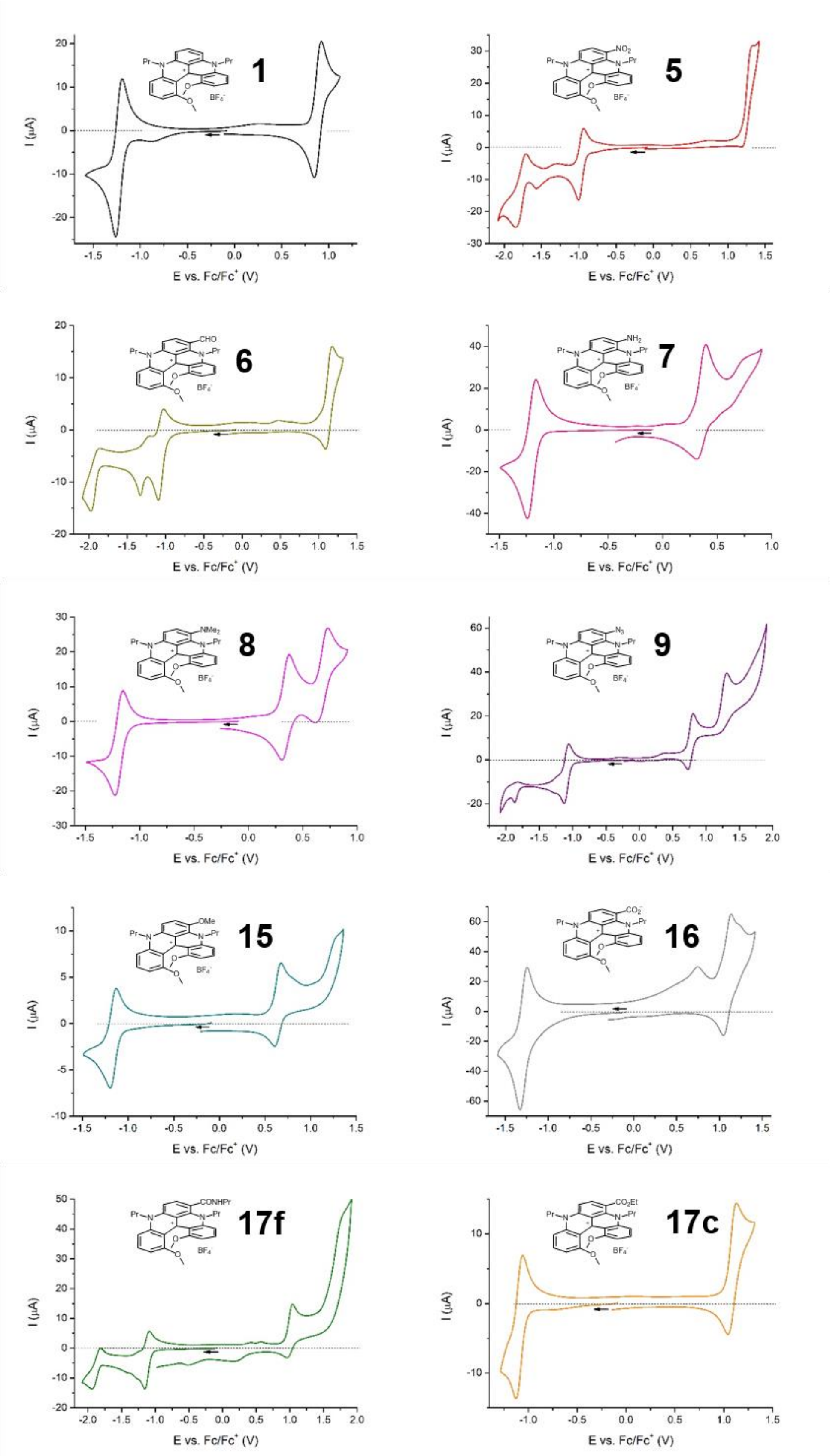

Figure S 3. Voltammetric curves of acetonitrile ([TBA][PF_6] 10^{-1} M) solutions of [4]helicenes (10^{-3} M) recorded at a Pt working electrode (v = 0.1 $V.s^{-1}$).

7. Additional optical data

1. Solvatochromism

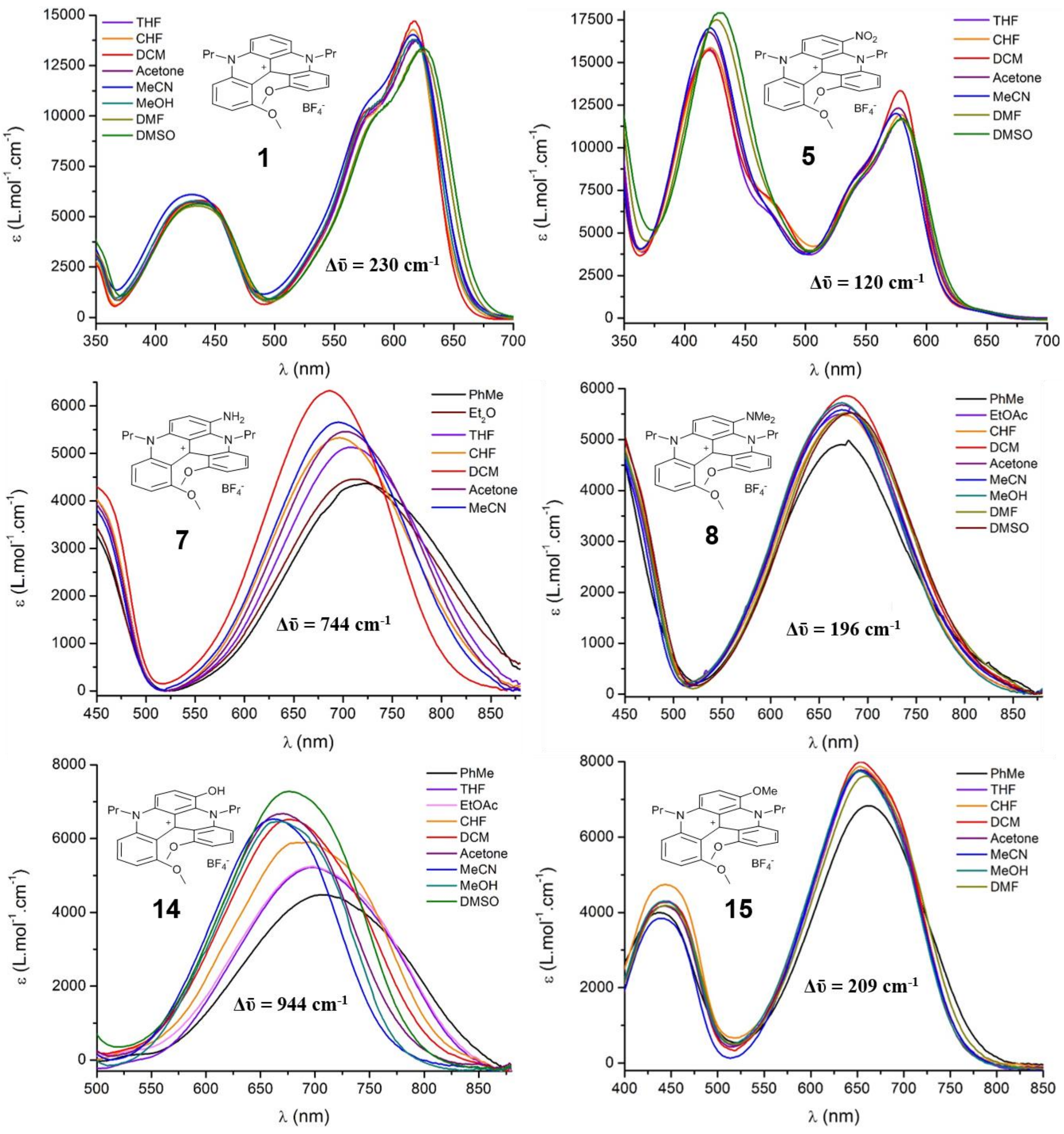

Figure S 4. Absorption solvatochromism (10^{-5} M) of compounds **1** (top left), **5** (top right), **7** (middle left), **8** (middle right), **14** (bottom left) and **15** (bottom right).

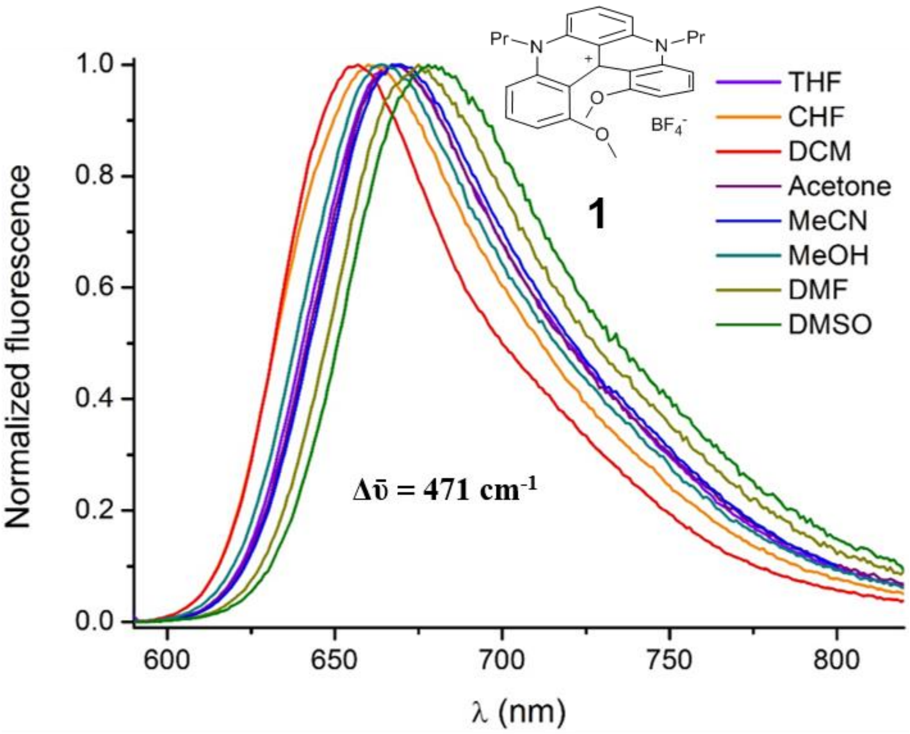

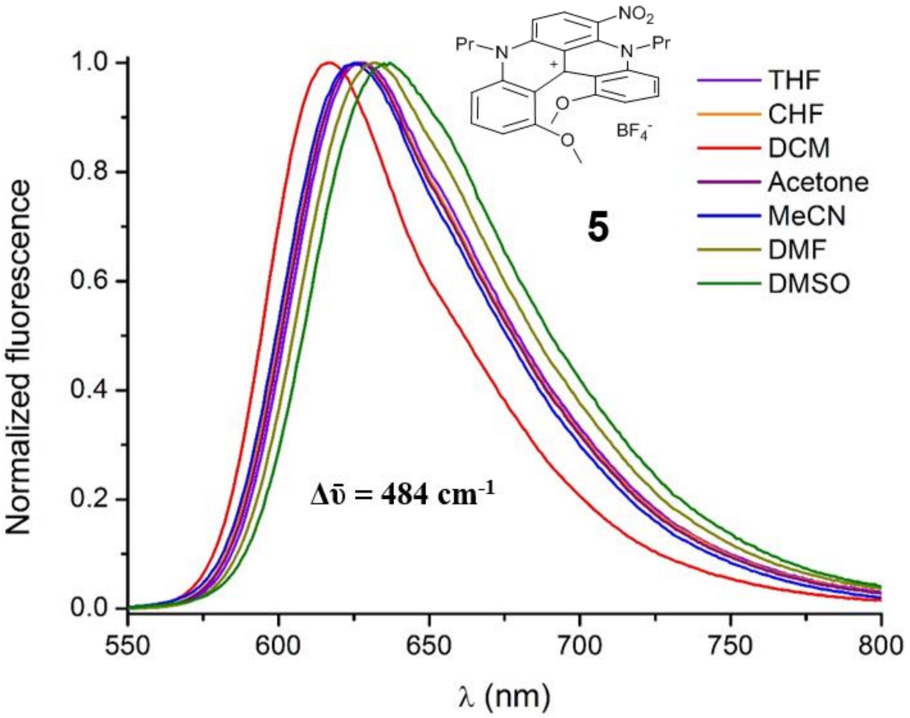

Figure S 5. Emission solvatochromism of compounds **1** (top) and **5** (bottom).

2. Fluorescence

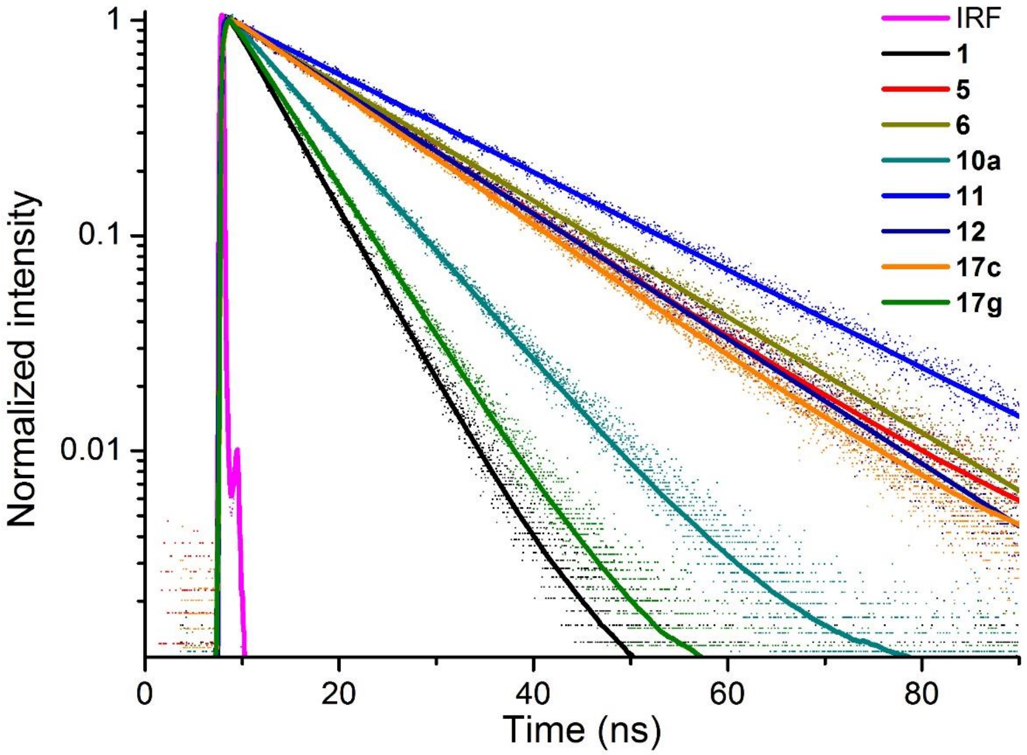

Figure S 6. Intensity normalized fluorescence time profiles and best single exponential fit of selected [4]helicene dyes in acetonitrile.

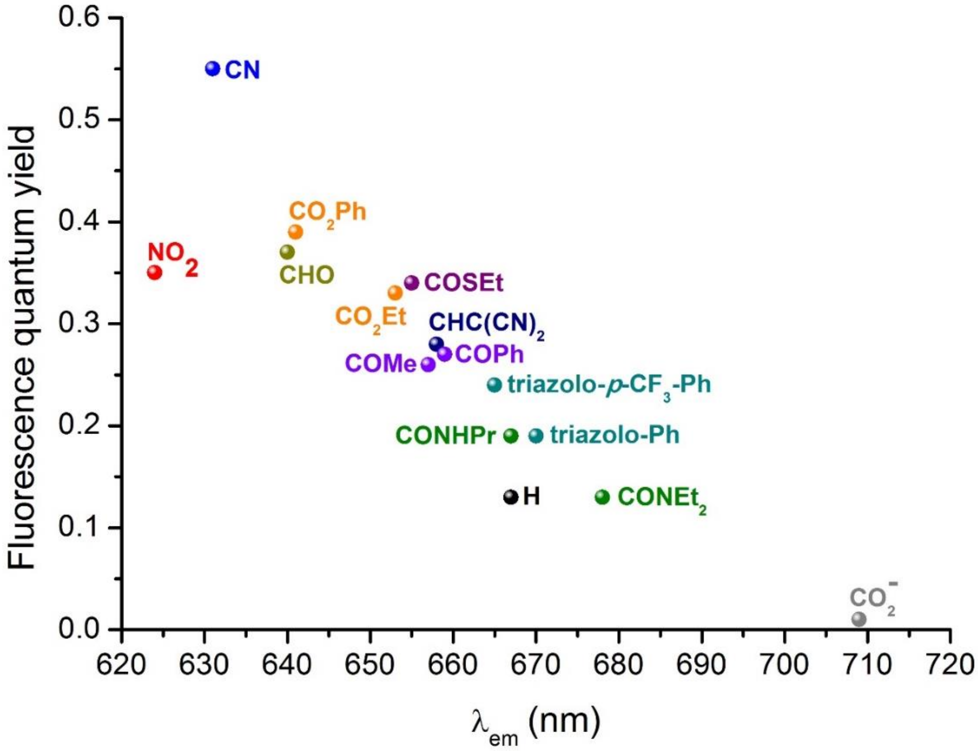

Figure S 7. Plot of fluorescence quantum yield depending on the emission maxima in acetonitrile.

3. ECD and OR data

Cationic [4]helicenes are dyes that absorb light efficiently in most of the visible region, leading to high errors during OR measurements. Thus very dilute solutions and restricted wavelengths were required to measure the specific optical rotations with Hg lamp.

1,13-dimethoxy-5,9-dipropyl-5,9-dihydro-13bH-quinolino[2,3,4-kl]acridin-13b-ylium tetrafluoroborate (1)

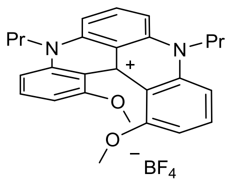

1

M enantiomer: **CD** (CH_3CN, $1.16.10^{-5}$ M, 20°C) λ (Δε) 640 (-2.2), 456 (7.2), 403 (-1.8), 350 (-27.8), 307 (23.4), 281 (-63.7). **OR** $[\alpha]_{365}$ = **–** 13600 (CH_3CN, c = $5.80.10^{-6}$ $g.mL^{-1}$, 20°C).

P enantiomer: **CD** (CH_3CN, $1.18.10^{-5}$ M, 20°C) λ (Δε) 648 (1.3), 455 (-6.4), 407 (2.2), 351 (31.7), 306 (-27.0), 281 (70.6).**OR** $[\alpha]_{365}$ = + 13900 (CH_3CN, c = $5.88.10^{-6}$ $g.mL^{-1}$, 20°C).

1,13-dimethoxy-6-nitro-5,9-dipropyl-5,9-dihydro-13bH-quinolino[2,3,4-kl]acridin-13b-ylium tetrafluoroborate (5)

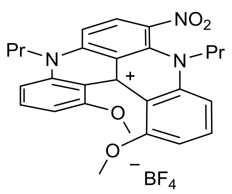

5

M enantiomer: **CD** (CH_3CN, $1.09.10^{-5}$ M, 20°C) λ (Δε) 579 (-5.6), 476 (6.3), 412 (-5.3), 340 (-31.1), 308 (-44.1), 287 (35.0). **OR** $[\alpha]_{365}$ = **–** 7600 (CH_3CN, c = $5.92.10^{-6}$ $g.mL^{-1}$, 20°C).

P enantiomer: **CD** (CH_3CN, $1.17.10^{-5}$ M, 20°C) λ (Δε) 582 (5.7), 473 (-7.8), 413 (4.5), 340 (32.7), 308 (47.0), 287 (-40.9). **OR** $[\alpha]_{365}$ = + 7100 (CH_3CN, c = $6.36.10^{-6}$ $g.mL^{-1}$, 20°C).

6-formyl-1,13-dimethoxy-5,9-dipropyl-5,9-dihydro-13bH-quinolino[2,3,4-kl]acridin-13b-ylium tetrafluoroborate (6)

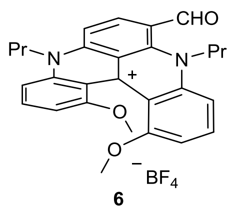

6

M enantiomer: **CD** (CH_3CN, $1.05.10^{-5}$ M, 20°C) λ (Δε) 594 (-4.6), 464 (8.9), 410 (-1.7), 380 (11.2), 337 (-10.9), 305 (-51.5), 263 (-7.1). **OR** $[\alpha]_{365}$ = **–** 9800 (CH_3CN, c = $5.52.10^{-6}$ $g.mL^{-1}$, 20°C).

P enantiomer: **CD** (CH_3CN, $1.11.10^{-5}$ M, 20°C) λ (Δε) 592 (3.9), 463 (-8.3), 412 (4.1), 381 (-11.9), 337 (14.3), 305 (63.5), 264 (10.2). **OR** $[\alpha]_{365}$ = + 11700 (CH_3CN, c = $5.88.10^{-6}$ $g.mL^{-1}$, 20°C).

6-amino-1,13-dimethoxy-5,9-dipropyl-5,9-dihydro-13bH-quinolino[2,3,4-kl]acridin-13b-ylium tetrafluoroborate (7)

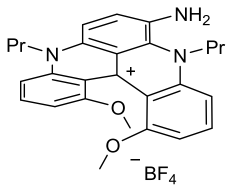

7

M enantiomer: **CD** (CH_3CN, $1.13.10^{-5}$ M, 20°C) λ (Δε) 723 (-3.8), 459 (10.3), 408 (-1.3), 363 (-14.3), 340 (-18.3), 313 (20.7), 283 (-37.9), 262 (-45.4). **OR** $[\alpha]_{365}$ = **–** 4100 (CH_3CN, c = $5.80.10^{-6}$ $g.mL^{-1}$, 20°C).

P enantiomer: **CD** (CH_3CN, $1.15.10^{-5}$ M, 20°C) λ (Δε) 726 (4.3), 462 (-11.0), 409 (1.1), 363 (14.4), 340 (18.0), 313 (-21.9), 283 (38.3), 262 (47.3). **OR** $[\alpha]_{365}$ = + 4600 (CH_3CN, c = $5.92.10^{-6}$ $g.mL^{-1}$, 20°C).

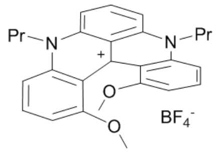

1

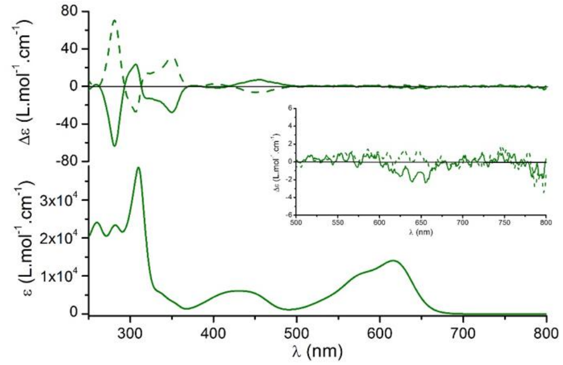

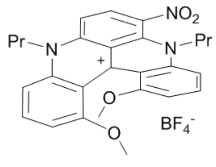

5

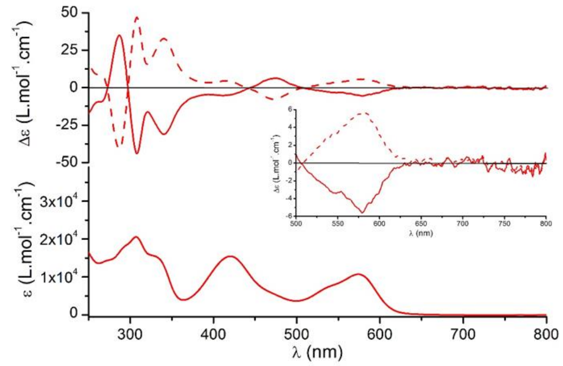

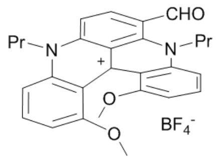

6

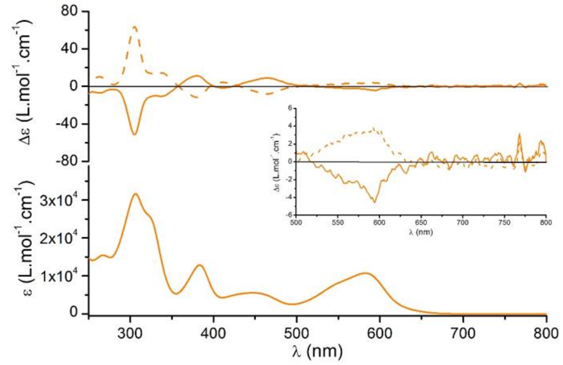

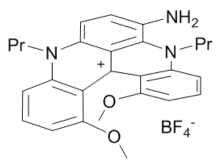

7

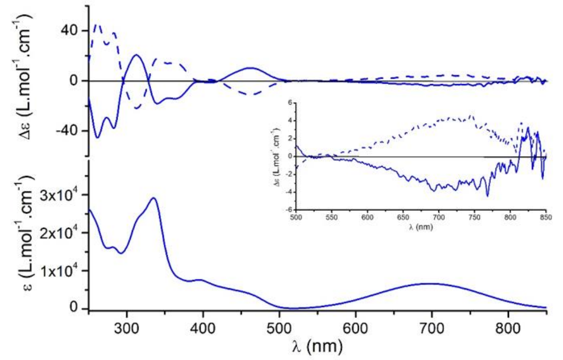

Figure S 8. Absorption versus ECD spectra of (*M*) (plain lines) and (*P*)-helices (dashed lines) for compounds **1** (green), **5** (red), **6** (orange) and **7** (blue) in acetonitrile (10^{-5} M) at 293 K. Insets: zoom of ECD in the Vis-NIR range.

8. References

(1) Laursen, B. W.; Krebs, F. C. *Angew. Chem. Int. Ed.* **2000**, *39*, 3432.

(2) Herse, C.; Bas, D.; Krebs, F. C.; Bürgi, T.; Weber, J.; Wesolowski, T.; Laursen, B. W.; Lacour, J. *Angew. Chem. Int. Ed.* **2003**, *42*, 3162.

(3) CrysalisPro Software system, Agilent Technologies UK Ltd., Oxford, UK.

(4) Sheldrick, G. *Acta Crystallogr. A* **2008**, *64*, 112.

(5) Burla, M. C.; Caliandro, R.; Camalli, M.; Carrozzini, B.; Cascarano, G. L.; De Caro, L.; Giacovazzo, C.; Polidori, G.; Spagna, R. *J. Appl. Crystallogr.* **2005**, *38*, 381.

(6) Palatinus, L.; Chapuis, G. *J. Appl. Crystallogr.* **2007**, *40*, 786.

(7) Dolomanov, O. V.; Bourhis, L. J.; Gildea, R. J.; Howard, J. A. K.; Puschmann, H. *J. Appl. Crystallogr.* **2009**, *42*, 339.

(8) Muller, P.-A.; Högemann, C.; Allonas, X.; Jacques, P.; Vauthey, E. *Chem. Phys. Lett.* **2000**, *326*, 321.

(9) Furstenberg, A.; Vauthey, E. *Photochem. Photobiol. Sci.* **2005**, *4*, 260.

(10) Brunet, E.; Jiménez, L.; de Victoria-Rodriguez, M.; Luu, V.; Muller, G.; Juanes, O.; Rodríguez-Ubis, J. C. *Microporous Mesoporous Mater.* **2013**, *169*, 222, and references therein.

(11) Dekkers, H. P. J. M.; Moraal, P. F.; Timper, J. M.; Riehl, J. P. *Appl. Spectrosc.* **1985**, *39*, 818.
